# Evaluation of Bifunctional, PSMA-Targeted Triazamacrocycle-Picolinates Compatible with the ^18^F/^44^Sc/^177^Lu Isotope Triad

**DOI:** 10.1021/acs.jmedchem.5c02286

**Published:** 2025-12-08

**Authors:** Owen M. Glaser, Hannah Goerlach, Megan V. Salek, Eduardo Aluicio-Sarduy, Mallory J. Gork, Dariusz Śmiłowicz, Edith Amason, Jason C. Mixdorf, Todd E. Barnhart, Jonathan W. Engle, Eszter Boros

**Affiliations:** † Department of Chemistry, 5228 University of Wisconsin-Madison, 1101 University Avenue, Madison, Wisconsin 53705, United States; ‡ Department of Medical Physics, University of Wisconsin-Madison, 1111 Highland Avenue, Madison, Wisconsin 53705, United States; § Department of Radiology, University of Wisconsin-Madison, Madison, Wisconsin 53705, United States

## Abstract

The development of matched diagnostic and therapeutic radiopharmaceuticals—theranostic pairs—has emerged as a promising strategy to advance personalized nuclear medicine. However, many current systems rely on chemically distinct elements such as the ^68^Ga^3+^/^177^Lu^3+^ pair, leading to inconsistencies in the pharmacokinetics. Here, we evaluate bifunctional chelator platforms derived of triazamacrocycle picolinates, capable of stably incorporating three clinically relevant isotopes ^18^F^–^, ^44^Sc^3+^, and ^177^Lu^3+^. **mpatcn** supported the formation of [^18^F]­[ScF], [^44^Sc]­[Sc], and [^177^Lu]­[Lu] complexes when conjugated to a PSMA-targeting peptide (**picaga-Met-hex-KuE**). **picaga-Met-hex-KuE** displayed quantitative radiochemical yields and >95% formulation stability after 2 h. Biodistribution and metabolite analysis confirmed PSMA-targeting, minimal off-target uptake, and renal clearance for all **picaga-Met-hex-KuE** systems. Additionally, we demonstrate that a cartridge-based purification method formulates [^18^F]­[ScF­(**picaga-Met-hex-KuE**)] in >95% radiochemical purity with nondecay corrected yields of 32% in under 110 min. These results establish **picaga-Met-hex-KuE** as a lead scaffold for the ^18^F/^44^Sc/^177^Lu triad, enabling single-kit radiopharmaceutical preparation for theranostic applications.

## Graphical abstract

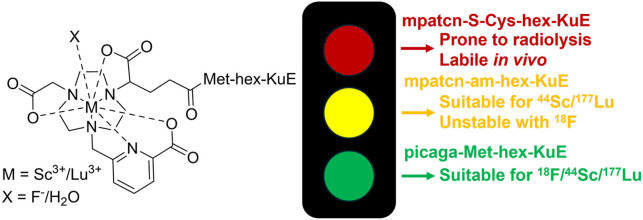

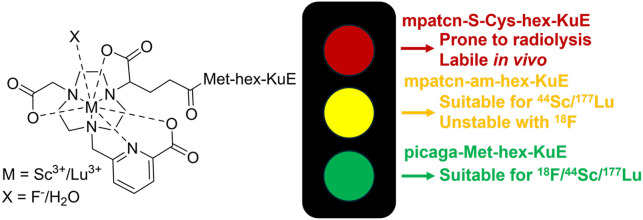

## Introduction

Personalized nuclear medicine with theranostic radiopharmaceuticals has emerged as a promising strategy to improve patient outcomes in the clinic.^1–4^ Theranostic pairs employ a diagnostic radionuclide, enabling noninvasive imaging and diagnosis through Positron Emission Tomography (PET) or Single Photon Emission Computed Tomography (SPECT) imaging, followed by radiotherapeutic treatment with beta- or alpha-emitting radionuclide.^5–7^ An ideal theranostic pair would employ radionuclides of the same element; however, only few elements have radionuclides that are suitable for both imaging and therapeutic purposes and can be readily produced at the clinical scale.^8^ While the theranostic pair of ^68^Ga^3+^ (*E*_β+avg_ = 0.89 MeV; *t*_1/2_ = 68 min) and ^177^Lu^3+^ (*E*_β–avg_ = 134 keV, *t*_1/2_ = 159.6 h) has shown to lead to improvement in patient outcomes; the differences in the coordination chemistry (Figure 1)^9^ of these metals result in differences in the pharmacokinetic profiles that preclude reliable dosimetry calculations.^10–16^

**Figure 1:**
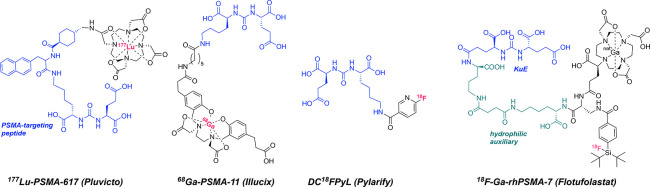
Structures of clinically employed, FDA-approved PSMA-targeted radiopharmaceuticals.

Due to differences in the coordination chemistry of metal radionuclides, many clinically employed radiochemical labeling strategies are not compatible with more than 1–2 radionuclides of interest. ^18^F^–^ (*E*_β+avg_ = 0.25 MeV, *t*_1/2_ = 110 min) remains the most extensively used PET nuclide despite scalability concerns due to favorable imaging properties.^17^

Radiofluorination approaches often involve C–F bond formations that can require large scale, multistep radiosynthesis in organic solvents, followed by radiochromatographic purification.^18^ The development of Al–^18^F and Si–^18^F radiochemistry^19,20^ is a step toward alleviating this issue, but these radiofluorination strategies are not readily interchangeable with therapeutic radionuclides. A recently FDA-approved compound Ga-rhPSMA-7 (Flotufolastat, Figure 1)^21^ provides multiple, distinct sites for radiolabeling with ^18^F^–^, ^68^Ga^3+^, or ^177^Lu^3+^ on a single molecule.^22^ However, implementation of radiopharmaceutical synthesis requires different chemical precursors depending on the nuclide incorporated and, due to the structure’s significant steric and lipophilic bulk, enhanced hepatic uptake is observed in vivo.^23^ New chemical tools are needed to incorporate clinically relevant radionuclides interchangeably with the disease homing targeting vector of choice.

Previously, we have demonstrated the ability of the fluoride anion to displace the inner sphere water in Sc^3+^ complexes and form a ternary Sc–F complex.^24,25^ The radiochemical species is readily formed by a two-step reaction, first forming the Sc–^18^F bond followed by chelation to form the corresponding [^18^F]­[ScF­(L)] complexes. Prior work has shown the ability of these ligands to generate structural homology between Sc^3+^ and Lu^3+^ complexes, providing chemical compatibility with three radionuclides of clinical interest, forming the ^18^F/^44^Sc/^177^Lu isotope triad.^26^ Following a thorough structure–activity–relationship campaign, we determined that the 7-coordiante ligands, **mpatcn** and **mpatcn-am-Bz**, were most ideally suited to stabilize the three radionuclides of interest in vivo (Figure 2).^24,27,28^

**Figure 2:**
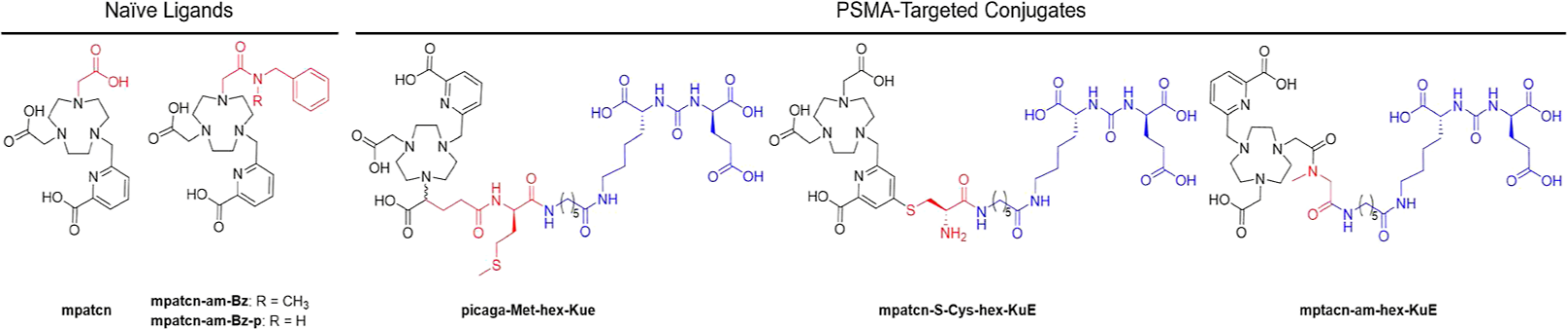
Structures of ligands discussed in this work. Structural differences are denoted in red, and the PSMA targeting peptide **hex-KuE** is denoted in blue.

Optimized bioconjugation and radiochemical labeling strategies that can be readily advanced toward clinical translation remain to be developed. In this work, we establish Sc–F speciation of our first generation leads and develop the synthesis of three bifunctional constructs appended to a cancer-targeting vector. Radiochemical labeling studies and experiments in a murine xenograft model shed light on the impact of functionalization on the radiolabeling, stability, and pharmacokinetic profile of the corresponding [^18^F]­[ScF­(L)] and [^44^Sc]­[Sc­(L)] PET imaging complexes and [^177^Lu]­[Lu­(L)] therapeutic complexes. These optimized systems are compatible with the current “gold standard” isotopes of ^18^F and ^177^Lu^3+^ and will be compatible with Sc^3+^ isotopes of interest, ^44^Sc^3+^ (*E*_β+avg_ = 632 keV, *t*_1/2_ = 4.04 h) and ^47^Sc^3+^ (*E*_β–avg_ = 162 keV, *t*_1/2_ = 3.35 days), as production capabilities are increased. The longer half-life of ^44^Sc^3+^ can enable imaging time points later than those of ^18^F, providing enhanced flexibility to clinicians. As the global demand for ^18^F increases, ^44^Sc^3+^ may provide an alternative in cases where ^18^F is not available. Finally, preliminary human trials have shown the ability for ^44^Sc^3+^ produced using a ^44^Ti-generator to enable imaging in locations where infrastructure for cyclotrons is not feasilble.^29^

## Results and Discussion

### Speciation and Optimization of Radiofluorination

To establish optimal parameters for the formation of ScF ternary complexes, we determined the pH-dependent speciation of ScF complexes with **H**_**3**_ **mpatcn** and **H**_**2**_ **mpatcn-am-Bz** using ^1^H and ^45^Sc NMR spectroscopy in accordance with previous methods established for the formation of lanthanide-fluoride complexes (Supporting Information, Section S3).^30^ Figure 3 provides pH-dependent speciation diagrams of both ligand systems and reveals that the 8-coordinate [ScF­(L)] species is readily stabilized for both systems between pH 3 and 8 while remaining in direct competition with a 7-coordinate, [Sc­(L)] species. Above pH 10, the [Sc­(L)­OH] species becomes most favorable, effectively outcompeting the F^–^ ligand at increasing concentration. Low pH conditions led to the presence of protonated species [Sc­(**mpatcn**)­H]^+^, which competes with inner sphere fluorination. The relative ratio of [ScF­(L)] to [Sc­(L)] species is 4:1 for **mpatcn** between pH 3 and 8, whereas **mpatcn-am-Bz** shows the inverse, 1:4 ratio in favor of the 7-coordinate [Sc­(**mpatcn-am-Bz**)]^+^ species. For both ligands, no statistically significant differences for pH dependence were observed, with the % of [ScF­(L)] species remaining constant between pH 3 and 8 (Table 1). This is in good correlation with radiochemical labeling experiments that produced comparable yields for **mpatcn** at pH 4.8 and 7.4, respectively (Table S11).

**Figure 3:**
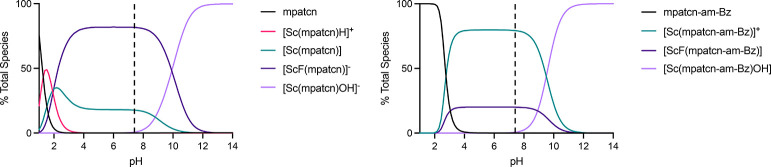
Data fit of [ScF­(**mpatcn**)]^−^. (Left) [ScF­(**mpatcn-am-Bz**)]. (Right) speciation in HySS2009. Modeling concentrations are set to 0.5 mM and 0.01 mM, respectively, corresponding to the concentration at which spectra were measured. Dashed line indicates physiological pH (7.4).

**Table 1:** Tabulated logß_MLX_ Constants for **mpatcn** and **mpatcn-am-Bz** as Determined by HypNMR2008

ligand	logß_MLH_	logß_ML_	logß_MLF_	logß_MLOH_
**mpatcn**	24.9	23	27.7	14.2
**mpatcn-am-Bz**	N/A	23.2	27.8	13.7

Prior investigation of the Sc^3+^ and Lu^3+^ speciation of **mpatcn** and **mpatcn-am-Bz** indicated that neutral ScF complexes may exhibit enhanced radiolabeling and in vivo inertness.^27^ An additional model ligand system, **mpatcn-am-p**, was synthesized (Supporting Information in Section S1 and Figure S2) to favor the formation of an amide-coordinated, charge-equilibrated species. While the corresponding, nonradioactive ScF complex readily formed using ^nat^F^–^, formation of the [^18^F]­[ScF­(**mpatcn-am-Bz-p**)] complex was not observed, and thus, **mpatcn-am-p** was not further investigated.

While speciation studies informed us of the pH range to stabilize the desirable [ScF­(L)] complex, we additionally hypothesized that metal impurities could reduce achievable Sc^18^F yields. Prior work by Goldberg and co-workers had shown that Al^3+^ can readily form Al^18^F bonds under conditions similar to what is employed for Sc^18^F chemistry.^31–35^ We conducted inductively coupled plasma-optical emission spectroscopy (ICP-OES) measurements and identified significant Al^3+^ contamination (1.9 ppm) in labeling solutions, possibly by leaching from scintillation vial lids. To further remove trace metal impurities, all glassware was soaked in 5% HNO_3_ for 24 h prior to Sc^18^F labeling studies. Labeling with **mpatcn** using this sample preparation strategy improved previously established radiochemical yields (RCY) for all of the investigated constructs (Table 2).

**Table 2:** Tabulated % RCY without and with HNO_3_ Washing

ligand	% RCY^28^	HNO_3_ rinse % RCY
**mpatcn**	33%	57%
**mpatcn-am-Bz**	37%	46%

### Computational Studies

Our previous structure–activity relationship (SAR) study indicated that steric bulk of the coordinating ligand controls [ScF­(L)] formation by modulation of ternary ligand access to the metal center.^27,36^ To probe this, we conducted DFT structure optimization^37^ of [Sc­(L)­OH_2_] complexes and determined the angular restriction of the aqua ligand relative to the chelate within each complex, defined by the smallest O_aqua_–Sc–O_ligand_ angle (Figure 4A). A plot of O_aqua_–Sc–O_ligand_ against the experimentally obtained radiochemical yield (RCY) for Sc^18^F formation is shown in Figure 4, including data obtained from previous studies.^25,27^ The observed trend indicates that ligands with large ternary coordination sites (>70°) produce a lower RCY. As the O_aqua_–Sc–O_ligand_ is restricted, RCY’s increase, with maximum values <70°. However, ligands with O_aqua_–Sc–O_ligand_ angle <55° efficiently exclude the binding of inner-sphere ligands, identifying a “goldilocks zone” for the O_aqua_–Sc–O_ligand_ of 55–70°, which readily accommodates the F^–^ anion and disfavors rapid displacement by H_2_O. To confirm this experimentally, we selected AAZTA, a 7-coordinate, mesocyclic ligand, previously validated as a suitable chelator for ^44^Sc and ^177^Lu isotopes.^38^ The corresponding crystal structure reveals an O_aqua_–Sc–O_ligand_ angle of 74.5°.^39^ Indeed, experiments to form the [^nat/18^F]­[ScF­(AAZTA)]^2–^ complexes were unsuccessful, and other chelators with >70° did not achieve yields above 15%.

**Figure 4:**
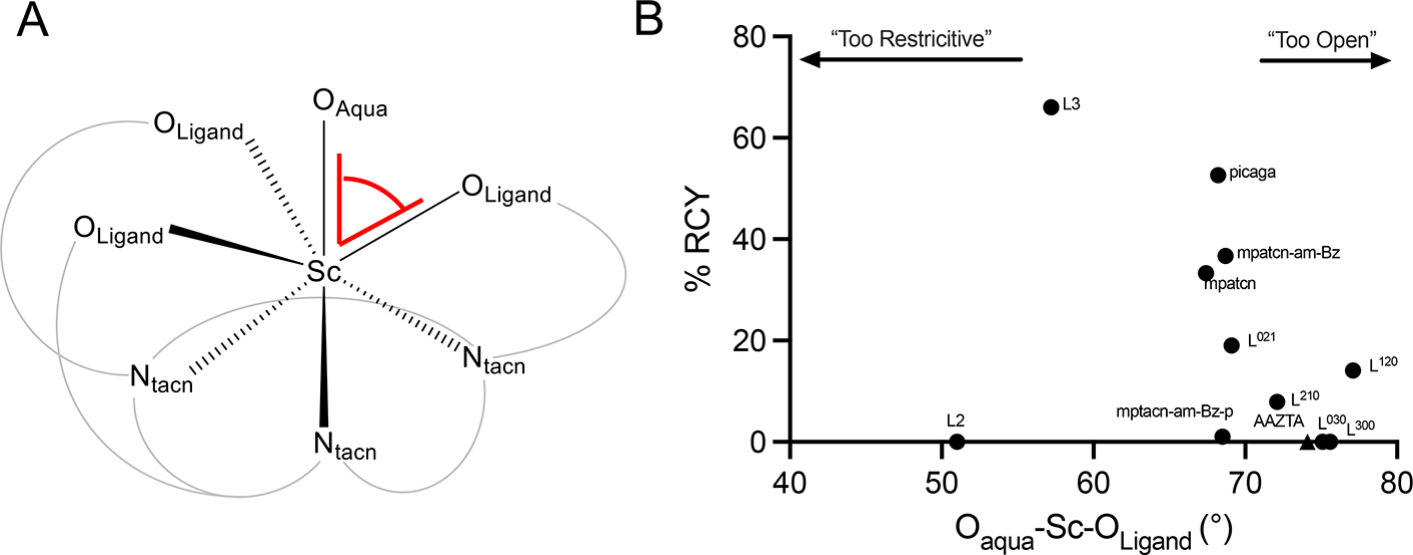
(A) Representative Sc complex structure denoting the O_aqua_–Sc–O_ligand_ bond angle. Gray arcs are used to denote the larger ligand scaffold. (B) O_aqua_–Sc–O_ligand_ bond angle plotted against % RCY from radiofluorination studies. AAZTA is denoted as ▲ for clarity.

### Bifunctional Chelator Synthesis

Three distinct functionalization strategies were chosen to probe if **mpatcn-am-Bz** and **mpatcn** bifunctionals could retain compatibility with the ^18^F/^44^Sc/^177^Lu triad. Functionalization with a **hexyl-KuE** peptide, targeting the prostate specific membrane antigen was utilized as a proof-of-concept targeting vector strategy.^40^

For **mpatcn-am-Bz**, we pursued the amidation of **mpatcn-am** through a *N*-(2-hydroxyacetyl)-*N*-methylglycine arm, which can be readily linked to the peptidic targeting vector **hex-KuE** via the free carboxylic acid.

For **mpatcn**, we probed two distinct conjugation strategies: (1) previous work from our lab has demonstrated that nitro-picolinate can be linked to the cysteine-functionalized peptide **Cys-KuE** by formation of thio-ether bonds.^41,42^ The resulting chelate would retain the coordinative environment of **mpatcn** closely, with only minute changes to the electronic properties of the picolinate, and was also compatible with selective conjugation without the need for orthogonal protection of chelator and peptide side-chains. (2) We have previously established that a glutarate functionalization strategy of one of the carboxylic acid arms represents an in vivo compatible bioconjugation strategy.^24,28^ To directly compare the influence of a thio-ether on the radiolytic stability of these constructs, we linked the validated picaga scaffold by amidation to the **Met-hex-KuE** peptide, incorporating the thio-ether containing the amino acid methionine.

For both **picaga-Met-hex-KuE** and **mpatcn-am-hex-KuE**, solid-phase synthesis was employed, with **hex-KuE** and **Met-hex-KuE** synthesized and coupled to orthogonally protected chelator precursors **11** and **20** (Scheme 1) on the resin. Global deprotection released the crude bifunctional chelate, which was isolated with >98% purity following reverse phase chromatography.

**Scheme 1:**
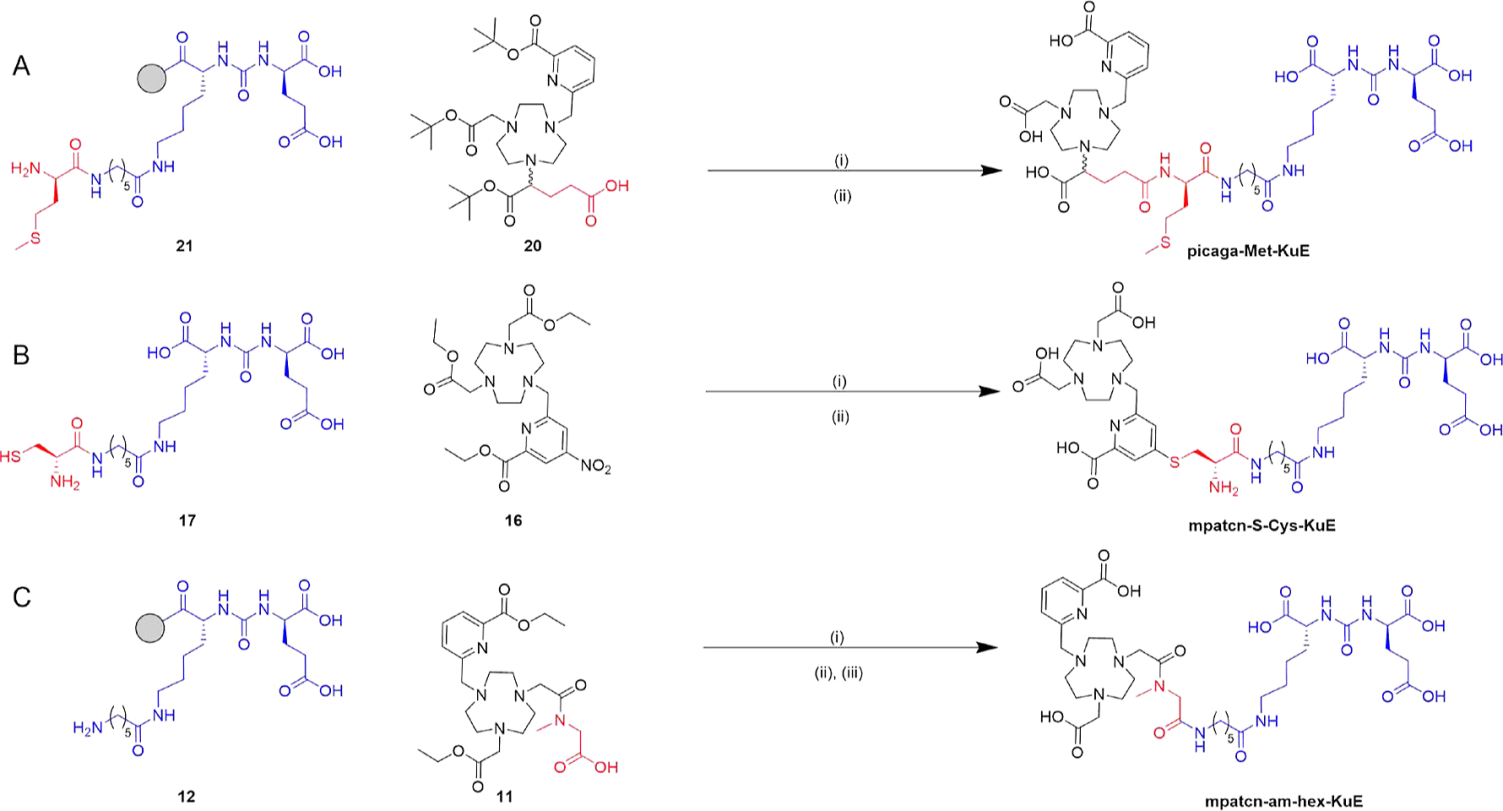
Abbreviated Synthesis of the Three PSMA Targeted Conjugates Discussed in This Work^a^

a Grey indicates Rink Amide resin used for SPPS, red indicates the linker moiety, and blue indicates the KuE PSMA targeting vector. (A) (i) PyBOP, DIPEA, DMF, (ii) TFA:DCM, (B) (i) TCEP, NH_4_HCO_3_:DMF, (ii) 1.0 M KOH, and (C) (i) PyBOP, DIPEA, DMF, (ii) TFA:DCM, (iii) 1.0 M KOH.

While the **Cys-hex-KuE** peptide was also synthesized on the solid phase, deprotection of the Mmt thiol protecting group was conducted in concert with the release of the peptide from the resin. The subsequent conjugation to the chelator precursor **16** was achieved in solution (Scheme 1), followed by isolation of the target conjugate with preparative reverse phase chromatography. While **picaga-Met-hex-KuE** and **mpatcn-am-hex-KuE** could be readily stored as dry powders at room temperature, **mpatcn-S-Cys-hex-KuE** required storage at −80 °C to prevent degradation under ambient conditions.

### Radiolabeling and Formulation Stability

Following synthesis, radiochemical labeling studies were performed to determine the % RCY and formulation stability for each conjugate (Scheme 2, Table 3). Following previously established radiochemical labeling approaches, radiofluorination was achieved by the preformation of [^18^F]­[ScF]^2+^ species, which was subsequently mixed with the ligand at 100 nmol mCi^–1^ molar activity and incubated at 80 °C for 30 min at pH 4.8 (0.25 M NH_4_OAc buffer).^25,27^ Radiochemical yield was then determined via radioHPLC, and the desired radiofluorinated complex was isolated chromatographically and formulated for injection into phosphate buffered saline (PBS).

**Scheme 2:**
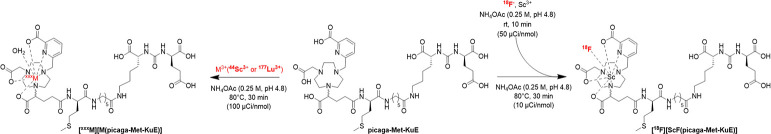
Example Reaction Scheme and Conditions for Radiochemical Labeling with ^18^F, ^44^Sc, ^177^Lu Isotopes^a^

a Relevant radionuclides are denoted in red.

**Table 3:** Tabulated % RCY and Formulation Stability at *t* = 0, 1, 2 h of the Three Conjugates with Sc^18^F, ^44^Sc, and Formulation Stability at *t* = 0, 2, 4 h with ^177^Lu

	Sc^18^F (0.01 mCi/nmol)				^44^Sc^3+^ (0.1 mCi/nmol)				^177^Lu^3+^ (0.1 mCi/nmol)			
ligand	% RCY	0 h	1 h	2 h	% RCY	0 h	1 h	2 h	% RCY	0 h	2 h	4 h
**mpatcn-am-hex-KuE**	60%	79%	n.d.	40%	71%	99%	n.d.	99%	95%	96%	95%	95%
**picaga-Met-hex-KuE**	53%	97%	97%	55%	99%	99%	97%	90%	98%	99%	99%	99%
**mpatcn-S-Cys-hex-KuE**	0%	N/A	N/A	N/A	96%	98%	n.d.	98%	89%	98%	88%	81%

The **picaga-Met-hex-KuE** and **mpatcn-am-hex-KuE** conjugates readily formed the corresponding Sc^18^F complexes. [^18^F]­[ScF­(**picaga-Met-hex-KuE**)] retained integrity throughout purification and formulation, whereas [^18^F]­[ScF­(**mpatcn-am-hex-KuE**)] degraded during purification, with only 79% of the complex remaining intact upon formulation. [^18^F]­[ScF­(**mpatcn-S-Cys-hex-KuE**)] did not form the radiofluorinated Sc^18^F complex.

Radiolabeling with the radiometal ions ^44^Sc and ^177^Lu was performed at 10 nmol mCi^–1^ molar activity and incubated at 80 °C for 30 min at pH 4.8 (0.25 M NH_4_OAc buffer).^27^ [^44^Sc]­[Sc­(**picaga-Met-hex-KuE**)] and [^177^Lu]­[Lu­(**picaga-Met-hex-KuE**)] formed with a >95% RCY, with subsequent formulation in PBS without additional purification. In contrast, while [^44^Sc]­[Sc­(**mpatcn-S-Cys-hex-KuE**)] displayed >95% RCY with ^44^Sc, the corresponding [^177^Lu]­[Lu­(**mpatcn-S-Cys-hex-KuE**)] formation was not quantitative with an 89% RCY, requiring chromatographic purification prior to formulation. Conversely, [^44^Sc]­[Sc­(**mpatcn-am-hex-KuE**)] only formed with a 71% RCY, whereas [^177^Lu]­[Lu­(**mpatcn-am-hex-KuE**)] produced a >95% yield. All radiometalated conjugates, once achieving sufficient radiochemical purity, readily formulated in PBS, with greater than 95% of the complex being intact at *t* = 0 h, and ≥90% formulation stability at *t* = 2 h.

### Targeted Nuclear Imaging and Biodistribution

To evaluate the in vivo performance of our radiolabeled constructs, we employed a murine xenograft tumor model with a PSMA-expressing PCP-3-PIP xenograft on the right shoulder and a contralateral PSMA-negative PC-3-flu xenograft on the left shoulder. Following injection, a static, PET/CT (^18^F, ^44^Sc) or SPECT/CT (^177^Lu) image was acquired at 90 min p.i., followed by sacking and ex vivo biodistribution measurements at 2 h post injection (p.i.).

For ^18^F, the formulation stability was highly predictive of in vivo stability with [^18^F]­[ScF­(**mpatcn-am-hex-KuE**)] showing defluorination, indicated by the high bone uptake, no significant PSMA + tumor uptake (Table S8) and affirmed by radioHPLC analysis of urine metabolites, showing only 16% of the complex intact 2 h p.i (Figure S106). In contrast, [^18^F]­[ScF­(**picaga-Met-hex-KuE**)] demonstrated minimal off target uptake in free F^–^ target organs such as bone.^43,44^ Ex vivo biodistribution studies confirmed PET/CT findings, with [^18^F]­[ScF­(**picaga-Met-hex-KuE**)] displaying 4.20 ± 0.92% ID/g in the PSMA + tumor and low kidney activity at 1.36 ± 0.58% ID/g (Figure 5). Urine metabolite analysis 2 h p.i. further confirms the enhanced stability of [^18^F]­[ScF­(**picaga-Met-hex-KuE**)], with 56 ± 31% of the labeled complex intact in the urine (Figure S114). We hypothesize that defluorination of [^18^F]­[ScF­(**picaga-Met-hex-KuE**)] likely occurs after renal clearance in the bladder and not in the blood due to low off target uptake and a favorable pharmacokinetic profile.

**Figure 5:**
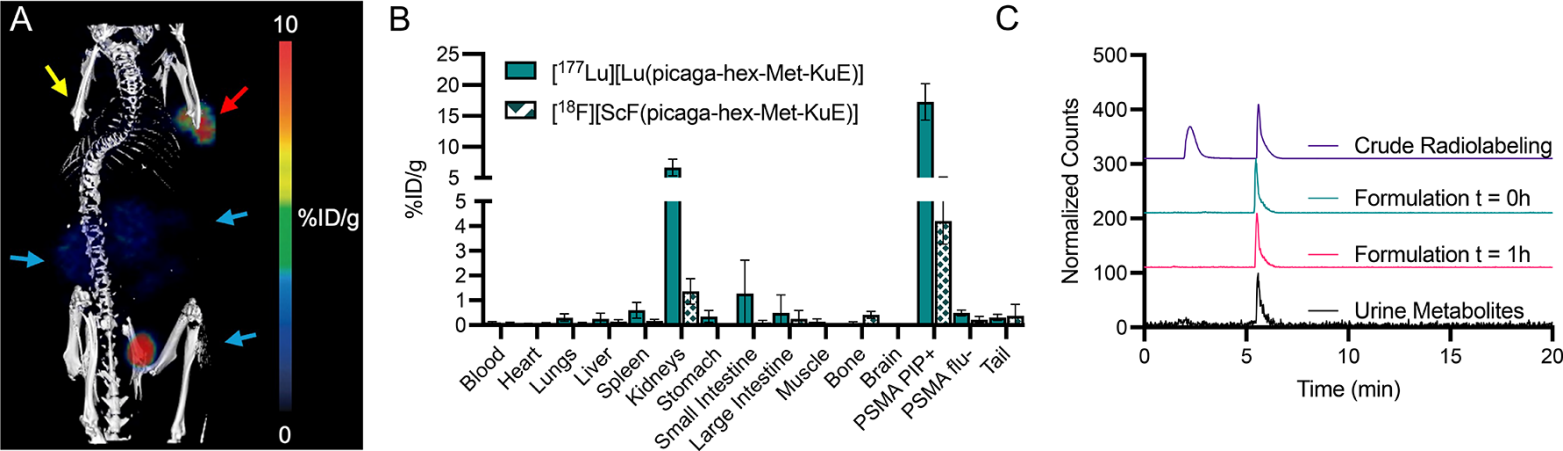
(A) Representative PET/CT of [^18^F]­[ScF­(**picaga-Met-hex-KuE**)] 90 min p.i. The yellow arrow indicates the PSMA flu – tumor, the red arrow indicates the PSMA PIP + tumor, and the blue arrows indicate the renal clearance pathway (kidneys and bladder). (B) Ex vivo biodistribution performed 2 h p.i. (C) Radio-HPLC traces of the crude radiolabeling, formulation at *t* = 0 and 1 h, and urine metabolites collected 2 h p.i.

For ^44^Sc^3+^, all three conjugates demonstrated high PSMA + tumor uptake and renal clearance, as indicated by kidney activity. Notably, [^44^Sc]­[Sc­(**picaga-Met-hex-KuE**)] displayed diminished kidney activity (4.99 ± 0.78% ID/g) 2 h p.i. as opposed to 17.25 ± 4.66 and 54.87 ± 12.71% ID/g for [^44^Sc]­[Sc­(**mpatcn-S-Cys-hex-KuE**)] and [^44^Sc]­[Sc­(**mpatcn-am-hex-KuE**)] respectively (Figure 6). Urine metabolite analysis highlights the increased stability of [^44^Sc]­[Sc­(**picaga-Met-hex-KuE**)] with 93.6 ± 2.07% intact in the urine, whereas [^44^Sc]­[Sc­(**mpatcn-S-Cys-hex-KuE**)] and [^44^Sc]­[Sc­(**mpatcn-am-hex-KuE**)] were only 28% and 39% intact, respectively (Table S9).

**Figure 6:**
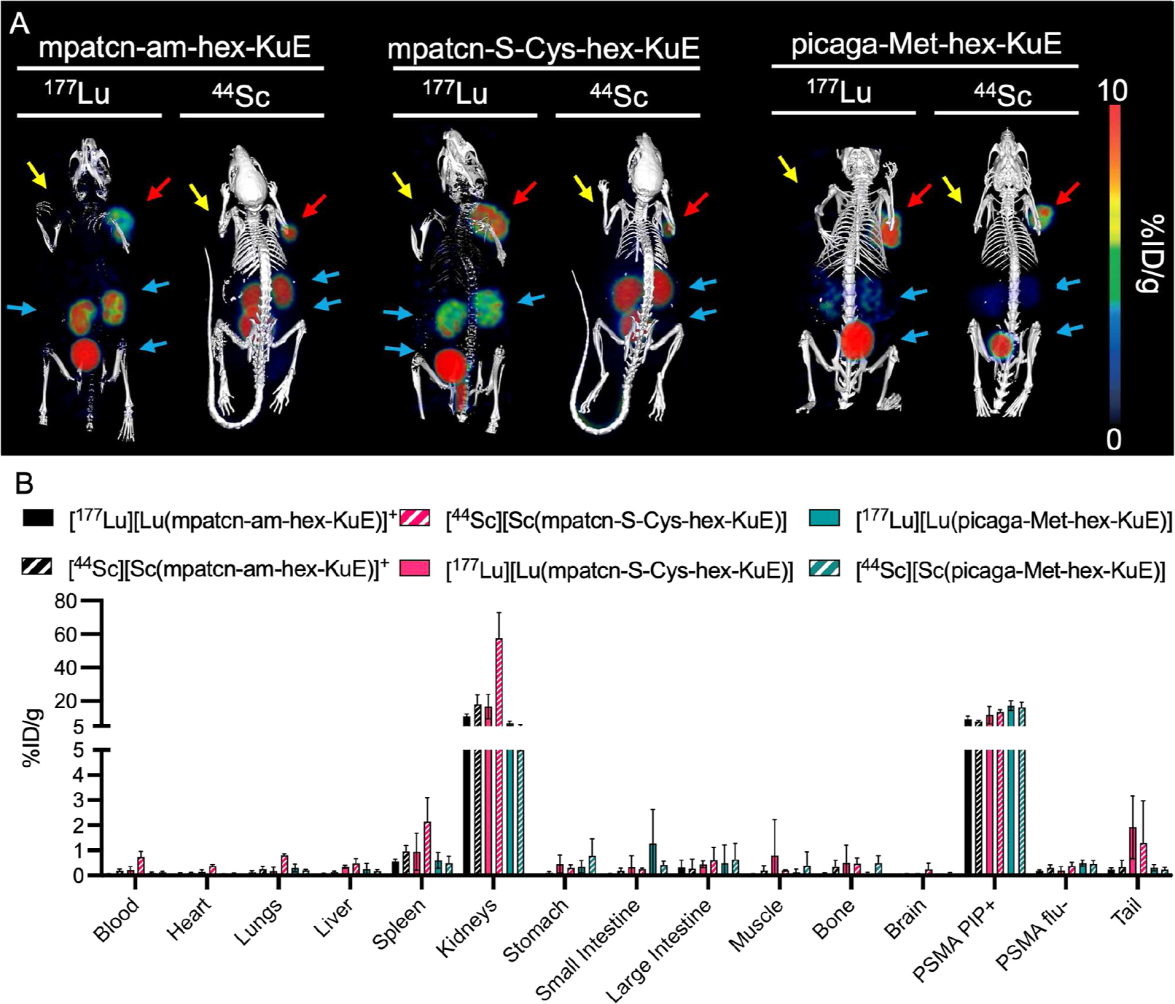
(A) Representative SPECT/CT and PET/CT of all three conjugates with ^177^Lu^3+^ and ^44^Sc^3+^, respectively, performed at 90 min p.i. The yellow arrow indicates the PSMA flu – tumor, the red arrow indicates the PSMA PIP + tumor, blue arrows indicate the renal clearance pathway (kidneys and bladder). (B) Ex vivo biodisitrubution performed 2 h p.i.

^177^Lu labeled conjugates compared well to their ^44^Sc counterparts, displaying statistically significant PSMA + tumor uptake and renal clearance. [^177^Lu]­[Lu­(**picaga-Met-hex-KuE**)] displays low kidney activity, with 6.66 ± 1.34% ID/g 2 h p.i (Figure 6). Despite displaying a similar pharmacokinetic profile to the other conjugates, [^177^Lu]­[Lu­(**mpatcn-S-Cys-hex-KuE**)] showed significantly more degradation as indicated by 68% intact in urine metabolites, whereas [^177^Lu]­[Lu­(**picaga-Met-hex-KuE**)] and [^177^Lu]­[Lu­(**mpatcn-am-hex-KuE**)] were 98% and 96% intact, respectively. Additionally, [^177^Lu]­[Lu­(**mpatcn-S-Cys-hex-KuE**)] was observed to have an enhanced bone uptake (0.50 ± 0.72% ID/g) when compared to the other conjugates, indicating possible dechelation of ^177^Lu, which typically deposits in the bone.^45^ When compared to the clinically employed ^177^Lu^3+^ therapy agent Pluvicto, [^177^Lu]­[Lu­(**picaga-Met-hex-KuE**)] performs well, displaying a higher tumor uptake 2 h p.i (Table S11). Additionally, [^44^Sc]­[Sc­(**picaga-Met-hex-KuE**)] and [^177^Lu]­[Lu­(**picaga-Met-hex-KuE**)] display a well matched biodistriubtion profile with tumor to blood ratios of 135 ± 41 and 172 ± 75.1 2 h p.i., for ^44^Sc^3+^ and ^177^Lu^3+^ respecitively. In comparison, the clinically employed Illucix and Pluvicto displays more divergent tumor to blood ratios of 500 ± 70.2 and 633 ± 54.5 2 h p.i., for ^68^Ga^3+^ and ^177^Lu^3+^ respectively.^9^ [^18^F]­[ScF­(**picaga-Met-hex-KuE**)] displays a tumor to blood ratio of 60 ± 36 while the clinically employed Pylarify displays a tumor to blood ratio 1220 ± 431 of 2 h p.i.^46^ While comparative imaging of Pylarify and [^18^F]­[ScF­(**picaga-Met-hex-KuE**)] in PCP-3-PIP and PC-3-flu models demonstrate the enhanced tumor uptake of Pylarify, [^18^F]­[ScF­(**picaga-Met-hex-KuE**)] displays reduced off-target uptake with a lower % ID/g present in the kidneys and liver (Figure S100, Tables S8 and S11).

While the biodistribution profiles of [^18^F]­[ScF­(**picaga-Met-hex-KuE**)] and [^177^Lu]­[Lu­(**picaga-Met-hex-KuE**)] are similar, quantitative differences in kidney and targeted tumor uptake can be seen in Figure 5. ^177^Lu^3+^ radiolabeling is performed at a molar activity 10× greater than Sc^18^F, 100 μCi/nmol and 10 μCi/nmol, respectively. Following purification, the Sc^18^F complex has a decay corrected molar activity of 33.3 μCi/nmol, still significantly lower than the ^177^Lu^3+^ labeled counterpart. Luurtsema and co-worker’s have previously reported the impact of molar activity on biodistrubution profiles of [^18^F]­[**FDOPA**].^47^ Additionally, while ScF and Lu^3+^ may share similar coordinative preferences, the ScF complex has a mononegative overall charge, while the Lu^3+^ complex is neutral. Hambley and co-workers have previously shown the impact of complex charge on the biodistibution profile of Pt^4+^ complexes.^48^ We hypothesize that these factors play a significant role in observed, minor differences in biodistribution of the **picaga-Met-hex-KuE** system, when combined with different radiochemical labeling strategies; however, dynamic imaging studies were not conducted and were outside the scope of the present SAR study.

### Binding Affinity to PSMA

To probe the impact of complex charge and chelate structure on target binding affinity, the IC_50_ of [^18^F]­[ScF­(**picaga-Met-hex-KuE**)] and [^177^Lu]­[Lu­(**picaga-Met-hex-KuE**)] was determined. This was performed through a radioactive displacement assay by challenging constant amounts of Pylarify with a varying concentration of the nonradioactive ScF and Lu^3+^ complexes. Our experiments revealed a *K*_i_ of 12.4 ± 0.5 and 12.1 ± 0.8 nM for the ScF and Lu^3+^ complexes, respectively (Figure 7). These values are consistent with previously reported values of **picaga-KuE** conjugates.^28^ The similarity between *K*_i_ values indicated that complex charge plays a minor role in differences between in vivo uptake suggesting that the significantly lower specific activity of [^18^F]­[ScF­(**picaga-Met-hex-KuE**)] may be a larger contributor to the deceased uptake.

**Figure 7:**
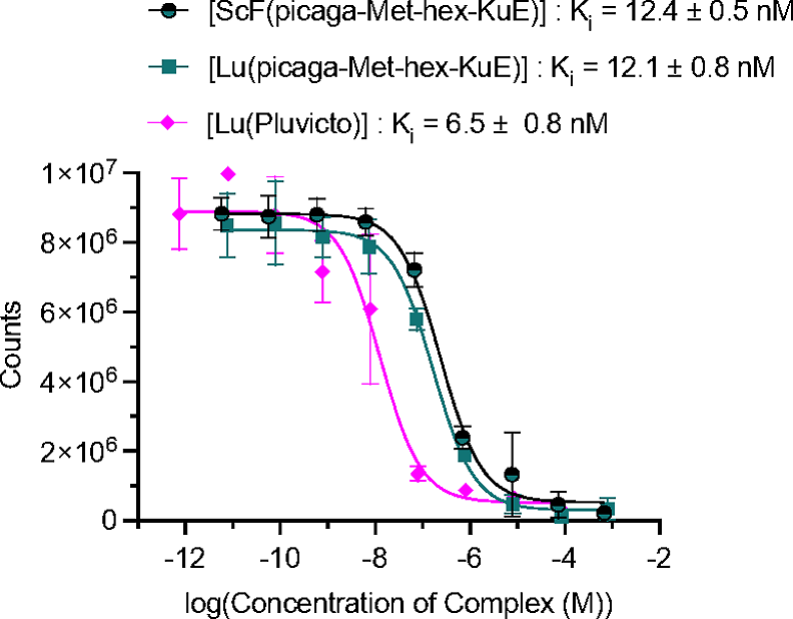
Displacement assay curves obtained with concentration dependent challenge of DCFPyL with [ScF­(**picaga-Met-hex-KuE**)], [Lu­(**picaga-Met-hex-KuE**)], and Pluvicto.

### Radiolytic Degradation Studies

In vivo experiments evidenced that [^44^Sc]­[Sc­(**mpatcn-S-Cys-hex-KuE**)] and [^177^Lu]­[Lu­(**mpatcn-S-Cys-hex-KuE**)] had diminished stability when compared with those of the other investigated conjugates. As the presence of a picolyl-adjacent thioether may predispose the construct to radiolytic degradation, we probed the effect of radioprotectants on the formulation stability of both thioether-containing derivatives, [^177^Lu]­[Lu­(**mpatcn-S-Cys-hex-KuE**)] and [^177^Lu]­[Lu­(**picaga-Met-hex-KuE**)].^49^ We observed that in the presence of ascorbate, both radiolabeled complexes remain intact >99% at *t* = 8 h. In the absence of ascorbate, [^177^Lu]­[Lu­(**mpatcn-S-Cys-hex-KuE**)] was only 75% intact at *t* = 8 h (Figure 8), whereas [^177^Lu]­[Lu­(**picaga-Met-hex-KuE**)] remained >98% intact in solution without the presence of ascorbate (Figure 8). These experiments support the role of radiolysis in destabilizing [^177^Lu]­[Lu­(**mpatcn-S-Cys-hex-KuE**)].

**Figure 8:**
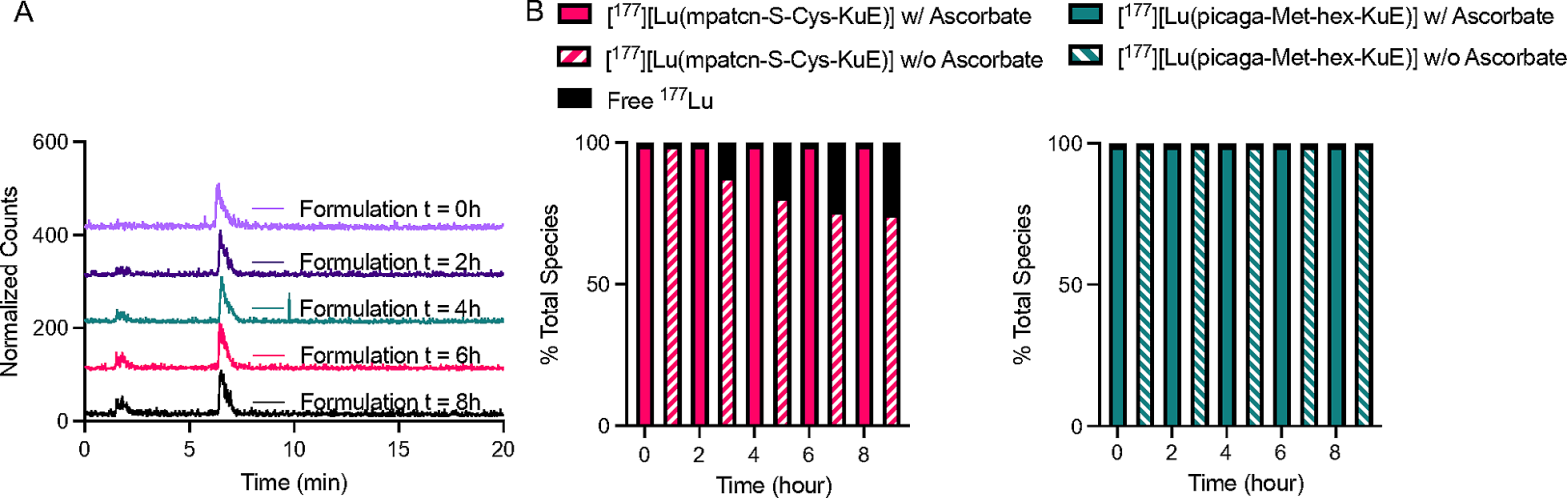
(A) Normalized radio-HPLC traces of [^177^Lu]­[Lu­(**mpatcn-S-Cys-hex-KuE**)] formulated in PBS without ascorbate. (B) Integrated values of intact complex and free activity present in the [^177^Lu]­[Lu­(**mpatcn-S-Cys-hex-KuE**)] (left) and [^177^Lu]­[Lu­(**picaga-Met-hex-KuE**)] (right) formulations with and without ascorbate.

### Cartridge Purification

While **picaga-Met-hex-KuE** serves as a versatile platform for ScF/Sc/Lu chemistry, the nonquantitative formation of [^18^F]­[ScF­(**picaga-Met-hex-KuE**)] complexes, necessitating chromatographic purification, poses a barrier to clinical translation. This strategy requires an additional 2 h, resulting in 50% decay of ^18^F, in addition to further losses incurred due to the fractional elution of the product, ultimately producing <26% nondecay corrected, ready-to-inject dose. In a clinical setting, this results in reduction of patient doses.^17,50,51^ To overcome this, we investigated a manual solid-phase separation employing commercially available QMA cartridge systems. Following aqueous priming, the crude radiolabeling solution (200 μL, 385 μCi) was loaded onto the column. Two milliliters of a 50 mM Kryptofix solution were used to elute nonchelated ^18^F.^52^ Fractional elution with NH_4_OAc (0.25 M pH 4.8) of the product fraction produced the desired product with >95% purity and 32% nondecay corrected yield after 45 min. All other fractions contained a low radiochemical purity (<95%) and a degraded product with a later Rt than the desired [^18^F]­[ScF­(**picaga-Met-hex-KuE**)] complex (Figure 9). The solid phase extraction strategy can be readily adapted and performed in <0.5 half-lives. Future work will focus on automation of the radiosynthesis using commercial, clinical cassette systems.^53,54^

**Figure 9:**
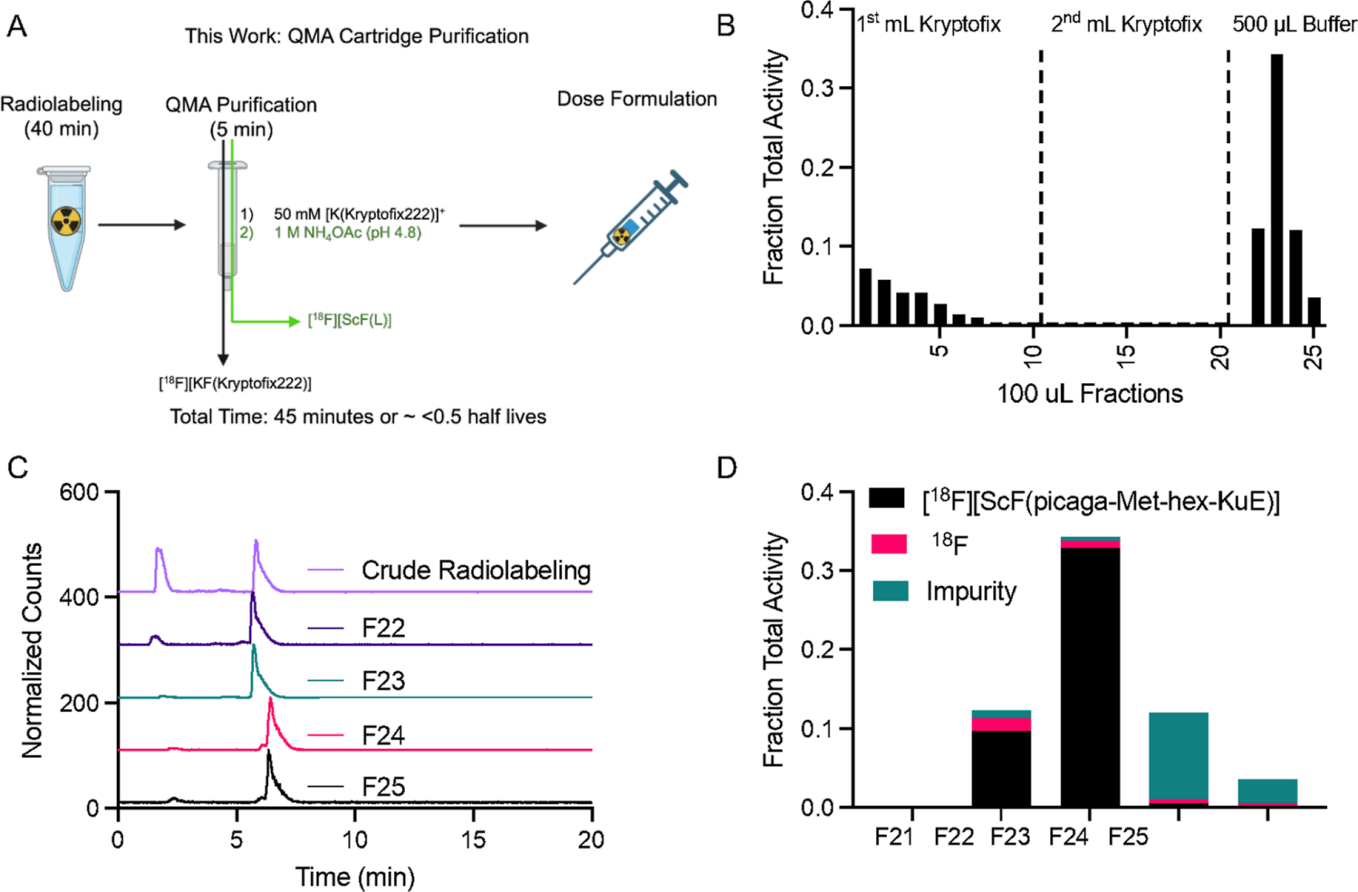
(A) Schematic diagram depicting QMA cartridge purification of [^18^F]­[ScF­(**picaga-Met-hex-KuE**)] using a QMA cartridge, kryptofix2.2.2., and NH_4_OAc buffer. (B) Elution profile of the cartridge system, broken down into 100 μL aliquots. (C) RadioHPLC traces of relevant fractions containing the desired purified [^18^F]­[ScF­(**picaga-Met-hex-KuE**)] complex. (D) Integrations of radioactive species present in relevant elution fractions.

## Conclusions

Current theranostic pairing systems lack properly matched pharmacokinetic profiles or lack a single chelator platform that enables a single kit for the formulation of both diagnostic and therapeutic systems. We conducted comprehensive solution and in silico studies that identified the parameters for chelator compatibility with the theranostic ^18^F/^44^Sc/^177^Lu triad. Subsequent evaluation of three distinct bifunctional chelator scaffolds linked to the PSMA-targeting peptide KuE identify **picaga-Met-hex-KuE** as the lead bifunctional system for all three nuclides, whereas the amide-linked conjugate **mpatcn-am-hex-KuE** is well suited for ^44^Sc^3+^ and ^177^Lu^3+^. A third derivative, **mpatcn-S-Cys-hex-KuE**, demonstrated moderate performance in vivo and showed enhanced radiolytic sensitivity.

The favorable in vivo performance of [^18^F]­[ScF­(**picaga-Met-hex-KuE**)] paired with its compatibility with a cartridge-based purification strategy motivates the future clinical translation of [^18^F]­[ScF­(**picaga-Met-hex-KuE**)] and [^177^Lu]­[Lu­(**picaga-Met-hex-KuE**)] as a single kit theranostic pair. Employing the pair as a single kit reduces the cost associated with synthesizing multiple compounds tailored to each isotope. Additionally, when employed as a triad, the kit can enhance physician flexibility and allow them to tailor treatment to patient needs. As ^4*x*^Sc^3+^ isotopes become more readily available and clinically validated, **picaga-Met-hex-KuE** is also readily compatible with the ^44^Sc/^47^Sc theranostic pair.^55^

## Experimental Section

### Equipment and Chemicals

All starting materials were purchased from Acros Organics, Alfa Aesar, Sigma-Aldrich, or TCI America and used without further purification. Unless otherwise noted, all solvents and reagents were used according to the manufacturer’s recommendations and stored in appropriate conditions.

### NMR Spectra

All proton ^1^H nuclear magnetic resonance spectra were recorded on 400 or 500 MHz Bruker or 600 MHz Advance III Bruker spectrometers. All carbon ^13^C {^1^H} nuclear magnetic resonance spectra were recorded on a 101 or 125 MHz Bruker or 175 MHz Advance III Bruker NMR spectrometer. Spectra were collected at 25 °C and processed using a MestReNova. Chemical shifts are expressed in parts per million (ppm, δ scale) and are referenced to residual CDCl_3_ (^1^H: δ 7.26 ppm, ^13^C: δ 77.1 ppm), CD_3_OD (^1^H: δ 3.31 ppm, ^13^C: δ 49.0 ppm), and (CD_3_)_2_SO (^1^H: δ 2.50 ppm, ^13^C: δ 39.5 ppm).^1^ Data is presented as follows: chemical shift, multiplicity (s = singlet, d = doublet, t = triplet, q = quartet, m = multiplet, and bs = broad singlet), integration, and coupling constant in hertz (Hz).

### Mass Spectrometry

Low-resolution electrospray ionization (ESI) mass spectrometry and high-resolution (ESI) mass spectrometry was carried out at the UW-Madison Chemistry Paul Bender Chemical Instrumentation Center Facility, with a Thermo Q ExactiveTM Plus (Electrospray Ionization-Quadrupole-Ion Trap) mass spectrometer.

### UV–Vis Spectra

Data was collected with a NanoDrop 1C instrument (AZY1706045), and spectra were recorded from 190 to 850 nm in a quartz cuvette with 1 cm path length.

### High-Performance Liquid Chromatography

Analytical HPLC analysis was carried out using a Shimadzu HPLC-20AR equipped with an autoinjector, binary gradient pump, Phenomenex Luna 5 μm C18(2) column (150 mm × 3 mm), and UV–vis detector. RadioHPLC was carried out using an Agilent 1220 Infinity II LC system equipped with a binary gradient pump, autoinjector, a Phenomenex Luna 5 μm C18(2) column (150 mm × 3 mm), UV–vis detector, and a LabLogic radio detector.

### LCMS

Low-resolution liquid chromatography-mass spectrometry (LC-MS) was carried out on a Phenomenex Luna 5 μm C18 column (150 mm × 3 mm, 100 Å, AXIA packed) at a flow rate of 0.8 mL/min using a single quadrupole Agilent 1200 Infinity II LC/MSD system equipped with a binary gradient pump, UV–vis detector, automatic injector, and an atmospheric pressure electrospray ionization (API-ES) source. UV absorption was recorded at 254 nm, and both positive and negative mass spectra were collected. Purity of all intermediate and final products, including radiochemical species, was determined using analytical HPLC. All conjugates and complexes were ≥95% pure.

### Inductively Coupled Plasma-Optical Emission Spectroscopy (ICP-OES)

Metal ion concentrations were determined with an Agilent 5110 ICP-OES. A 10-point standard calibration curve with respect to scandium or lutetium was used, and a line of best fit was found with *R* ^2^ of 0.999.

### HPLC Methods

#### Method A (Analytical)

Binary solvent system (A: water + 0.1% TFA; B: MeCN + 0.1% TFA); gradient (0–2 min: 5% B; 2–14 min: 5–95% B; 14–16 min: 95% B; 16–16.5 min: 95–5% B; 16.5–20 min, 5% B); flow rate: 0.8 mL/min; column: Phenomenex Luna C18 column (5 μm, 150 mm × 3 mm, 100 Å, AXIA packed).

#### Method B (Analytical)

Binary solvent system (A: 10 mM ammonium formate pH 7.4; B: MeCN); gradient (0–2 min: 0% B; 2–14 min: 0–90% B; 14–16 min: 90% B; 16–16.5 min: 90–0% B; 16.5–20 min: 0% B); flow rate: 1.0 mL/min; temperature: 50 °C; column: Restek Ultra AQ C18 column (5 μm, 250 mm × 3 mm).

#### Method C (Analytical)

Binary solvent system (A: water + 0.1% TFA; B: MeCN + 0.1% TFA); gradient (0–2 min: 0% B; 2–14 min: 0–90% B; 14–16 min: 90% B; 16–16.5 min: 90–0% B; 16.5–20 min: 0% B); flow rate: 1.0 mL/min; temperature: 50 °C; column: Restek Ultra AQ C18 column (5 μm, 250 mm × 3 mm).

#### Method D (LCMS)

Binary solvent system (A: water + 0.1% FA; B: MeCN + 0.1% FA); gradient (0–3 min: 5% B; 3–10 min: 5–95% B; 10–12 min: 95% B; 12–12.5 min: 95–5% B; 12.5–16 min: 5% B); flow rate: 0.8 mL/min; column: Phenomenex Luna C18 column (5 μm, 150 mm × 3 mm, 100 Å, AXIA packed).

#### Method E (Semi-preparative)

Binary solvent system (A: 0.1% TFA in water; B: 0.1% TFA in MeCN); gradient (0–5 min: 5% B; 5–24 min: 5–95% B; 24–27 min: 95% B; 27–27.5 min: 95–5% B; 27.5–30 min: 5% B); flow rate: 10 mL/min; column: Phenomenex Luna C18 column (10 μm, 250 × 10 mm, 100 Å, AXIA packed).

### Naïve Ligand Synthesis

#### **mpatcn-am-Bz-p** (**8**)

**7** (5.5 mg, 73.2 μmol, 1 equiv) was dissolved in 1 mL of 0.1 M KOH and allowed to stir for 3 h. Following this, excess solvents were removed to give the crude product in quantitative yield. ^1^H NMR (500 MHz, MeOD): δ 7.94 (d, *J* = 7.7 Hz, 1H), 7.80 (t, *J* = 7.7 Hz, 1H), 7.40–7.21 (m, 9H), 4.45 (d, *J* = 3.1 Hz, 1H), 4.36 (s, 2H), 3.83 (s, 2H), 3.18 (s, 2H), 3.09 (s, 2H), 2.69 (t, *J* = 5.3 Hz, 2H), 2.66–2.62 (m, 3H), 2.61–2.57 (m, 4H), 2.47 (s, 2H), 2.36 (s, 2H). ^13^C NMR (126 MHz, MeOD): δ 177.89, 171.83, 169.02, 162.08, 161.81, 161.54, 161.26, 158.09, 138.50, 137.42, 128.19, 128.15, 128.13, 127.39, 127.16, 127.12, 126.88, 126.82, 122.26, 120.37, 118.04, 115.71, 113.38, 70.12, 62.17, 61.85, 52.96, 48.11, 48.06, 47.94, 47.77, 47.73, 47.61, 47.60, 47.56, 47.43, 47.33, 47.26, 47.09, 42.69, 42.12. ESI-HR-MS: Calcd for C_24_H_31_N_5_O_5_ [M – H]^−^, *m*/*z* 468.2252; found, 468.2252.

### Conjugate Synthesis

#### **mpatcn-am-hex-KuE** (**14**)

**13** (3.0 mg, 3.4 μmol, 1 equiv) was dissolved in 1 mL of 1 M KOH and allowed to stir overnight. Excess solvent was removed, and the crude product was purified by semipreparative reverse-phase HPLC (Method E). Pure fractions were pooled and lyophilized to yield a white powder (1.0 mg, 1 μmol, 29% yield). ^1^H NMR (500 MHz, MeOD): δ 7.99 (t, *J* = 7.3 Hz, 1H), 7.89 (td, *J* = 7.8, 5.0 Hz, 1H), 7.77 (dd, *J* = 15.2, 7.6 Hz, 1H), 4.46 (s, 3H), 4.36 (q, *J* = 7.1 Hz, 2H), 4.23 (s, 1H), 4.19 (s, 1H), 4.10–4.00 (m, 3H), 3.96 (t, *J* = 7.1 Hz, 4H), 3.41 (s, 2H), 3.19–3.08 (m, 2H), 2.93 (d, *J* = 7.8 Hz, 6H), 2.85 (s, 1H), 2.26 (dt, *J* = 11.3, 5.9 Hz, 2H), 2.08 (q, *J* = 7.0 Hz, 3H), 2.00 (s, 1H), 1.90–1.79 (m, 1H), 1.70 (s, 2H), 1.51 (dt, *J* = 14.6, 6.9 Hz, 5H), 1.42 (s, 5H), 1.32 (t, *J* = 7.1 Hz, 4H), 1.24–1.11 (m, 14H), 0.80 (t, *J* = 6.8 Hz, 1H). ESI-HR-MS: Calcd for C_38_H_59_N_9_O_14_ [M + H]^+^, *m*/*z* 866.4254; found, 866.4255.

#### **mpatcn-S-Cys-hex-KuE** (**19**)

**9** (2.0 mg, 2.2 μmol, 1 equiv) was dissolved in 1 mL of 1 M KOH and allowed to stir at room temperature overnight. Excess solvent was removed, and the crude product was purified by semipreparative reverse-phase HPLC (Method E). Pure fractions were pooled and lyophilized to yield a white powder (0.22 mg, 250 nmol, 11% yield). Due to rapid degradation, ^1^H NMR of the pure final product was unable to be collected. ESI-LR-MS: Calcd for C_38_H_57_N_9_O_15_S [M + H]^+^, *m*/*z* 914.4; found, 914.2.

#### **picaga-Met-hex-KuE** (**22**)

**20** (24.6 mg, 39.6 μmol, 1 equiv) was dissolved in 1 mL DMF. Following this, PyBOP (61.9 mg, 119 μmol, 3 equiv) and DIPEA (34.5 μL, 198 μmol, 5 equiv) were added, and the solution was allowed to stir for 10 min. The solution was loaded onto a syringe containing **21** (101 mg, 0.0594 mmol, 1.5 equiv) on resin. The syringe was shaken overnight, before excess solvent was removed. The syringe was then loaded with TFA:TIS:H_2_O (95:2.5:2.5) and shaken for 5 h before the solution was eluted off the syringe. Excess solvent was removed, and the crude product was purified by semipreparative reverse-phase HPLC (Method E). Pure fractions were pooled and lyophilized to yield a white powder (2 mg, 2 μmol, 5% yield). ^1^H NMR (600 MHz, D_2_O): δ 8.34 (d, *J* = 0.7 Hz, 2H), 7.94 (dd, *J* = 22.2, 7.6 Hz, 1H), 4.70 (s, 3H), 4.16 (d, *J* = 12.8 Hz, 2H), 3.95 (ddt, *J* = 16.4, 7.8, 4.4 Hz, 2H), 3.73–3.63 (m, 2H), 3.25 (d, *J* = 0.7 Hz, 2H), 3.06 (td, *J* = 6.9, 4.3 Hz, 5H), 3.02 (s, 3H), 2.90 (s, 2H), 2.73 (d, *J* = 6.3 Hz, 2H), 2.34 (t, *J* = 8.6 Hz, 3H), 2.29–2.22 (m, 2H), 1.79 (s, 1H), 1.47 (dd, *J* = 6.2, 3.1 Hz, 2H), 1.45 (s, 4H), 1.43 (s, 4H), 1.42 (s, 3H), 1.39 (s, 3H), 1.26 (p, *J* = 7.6 Hz, 3H), 1.19–1.12 (m, 3H). ^13^C NMR (151 MHz, D_2_O): δ 215.37, 170.75, 62.40, 55.05, 39.09, 30.19, 27.96, 25.42, 25.00, 24.30, 22.41, 17.65. ESI-HR-MS: Calcd for C_43_H_67_N_9_O_16_S [M – H]^−^, *m*/*z* 996.4426; found, 996.4336.

### Radioisotope Production

^18^F and ^44^ScCl_3_ were received from the University of Wisconsin–Madison Cyclotron group (produced on a GE PETtrace cyclotron). ^177^LuCl_3_ was received from Shine Medical Technology in 0.04 M HCl.

### General ^18^F Radiolabeling Procedure

Radiolabeling was conducted following a literature procedure.^24^ To an aqueous solution of ammonium acetate (50 μL, 1 M, pH 4.8) was added an aliquot of unprocessed [^18^F]­F^–^ stock (110–143 μL, ∼1 mCi) followed by an aliquot of a ScCl_3_·6H_2_O stock solution (2–20 μL, 20 nmol) of known concentration as determined by ICP-OES or MP-AES. Following incubation at room temperature for 10 min, an aliquot of a ligand stock solution (5–20 μL, 100 nmol), of known concentration as determined UV–vis spectroscopy, was added. Total reaction volume = 200 μL. All radiolabeling experiments were performed at 0.01 mCi/nmol. The mixtures were incubated at 80 °C for 30 min prior to radioHPLC analysis (Method B). The same procedure was used for animal studies. Following purification of the labeled compounds for animal studies, injection solutions were prepared with a decay corrected specific activity of 0.03 mCi/nmol for [^18^F]­[ScF­(**picaga-Met-hex-KuE**)]. Due to formulation instability, an exact specific activity of [^18^F]­[ScF­(**mpatcn-am-hex-KuE**)] at the time of injection was not able to be determined.

### General ^44^Sc/^177^Lu Radiolabeling Procedure

Radiolabeling was conducted following a literature procedure.^24^ To an aqueous solution of ammonium acetate (10 μL, 1 M, pH 4.8) was added a ligand stock solution (16–54 μL, 10 nmol), of known concentration as determined by UV–vis spectroscopy, followed by an aliquot of the [^44^Sc]­ScCl_3_ or [^177^Lu]­LuCl_3_ stock (36–74 μL, ∼1 mCi). Total reaction volume = 200 μL. All radiolabeling experiments were performed at a specific activity of 0.1 mCi/nmol. The mixtures were incubated at 80 °C for 30 min prior to radio-HPLC analysis (^45^Sc: Method C; ^177^Lu: Method C). The same procedure was used for animal studies. For animal studies, injection doses were prepared with a specific activity of 0.1 mCi/nmol.

### PET/CT Imaging

Positron emission tomography/computed tomography (PET/CT) imaging was performed using either a Siemens Inveon Hybrid MicroPET/CT Scanner (Siemens Medical Solutions USA, Inc., Knoxville, TN) or a Mediso nanoScan microPET/CT Scanner (Mediso, Budapest, Hungary). Mice were anesthetized with 4% isoflurane gas, and anesthesia was maintained during scans at 2% isoflurane in oxygen. CT scans were acquired prior to PET scans for anatomical coregistration as well as attenuation correction. CT scan parameters were as follows: 220 rotation degrees, 120 rotation steps, binning factor of 4, exposure time of 250 ms, X-ray energy of 80 kVp, 1 mA current, and 105 μm resolution. PET scans were acquired with 40 million coincidence events per mouse, an energy window of 350–650 keV, and a timing window of 3.432 ns. Quantification of PET/CT images was performed in an Inveon Research Workstation, and data are expressed as percent injected dose per gram of tissue (% ID/g).

### SPECT/CT Imaging

Mice were inducted with 4% isoflurane gas anesthesia, placed on a heated bed with 2% maintenance, and subsequently scanned on an MILabs U-SPECT6CTUHR microsingle photon emission computed tomography/computed tomography (μSPECT/CT) system (MILabs; Houten, The Netherlands). SPECT data were acquired using the General Purpose Rat/Mouse collimator (GP-RM; 1.5 mm pinholes) with 4 frames in spiral scan mode using normal steps for a total of 40 min of effective acquisition time. SPECT data were reconstructed based on the 114 and 208 keV photopeaks with a 20% window of the photopeak and background subtraction using the automatic triple energy windows (8.3 and 4.6 keV 114 keV photopeaks, respectively) into one static image with 0.4 mm isotropic voxels, SROSEM reconstruction with 128 subsets and 5 iterations, and a 1.4 mm full width half max (fwhm) smoothing filter.

### Ethical Use of Animals

All animal experiments were conducted with the approval of the University of Wisconsin–Madison Institutional Animal Care and Use Committee (IACUC). All studies were conducted in accordance with the relevant guidelines and regulations, approved under protocol number M00678 and conducted at the UW-Madison School of Medicine and Public Health (Small Animal Imaging and Radiotherapy Facility, SAIRF).

## Supplementary Material





## Notes

Detailed synthesis procedures, characterization data, cleavage experiments, mechanism studies, images, and related biodistribution analysis (PDF)
Molecular formula strings: SMILES (CSV)

## Abbreviations

Ac: acetate
AcN: acetonitrile
API-ES: atmospheric pressure ionization-electrospray
Ar: aromatic
Ar­(Bz): aromatic benzyl
Ar­(Pa): aromatic picolinic acid
Asc: ascorbate
Bz: benzyl
CV: column volume
DCM: dichloromethane
DFT: density functional theory
DIPEA: *N*,*N*-diisopropylethylamine
DMAP: 4-dimethylaminopyridine
DMEM: Dulbecco’s modified Eagle medium
DMF: dimethylformamide
DMSO: dimethyl sulfoxide
ESI: electrospray ionization
EtOAc: ethyl acetate
FBS: fetal bovine serum
HEPES: 2-[4-(2-hydroxyethyl)­piperazin-1-yl]­ethanesulfonic acid
HPLC: high performance liquid chromatography
HR: high resolution
Hz: hertz
ICP-OES: inductively coupled plasma-optical emission spectroscopy
IS: internal standard
LC: liquid chromatography
LR: low resolution
MeCN: acetonitrile
MeOH: methanol
MS: mass spectrometry
NMR: nuclear magnetic resonance
PBS: phosphate buffered saline
PVDF: polyvinylidene fluoride
QTAIM: quantum theory of atoms in molecules
RCY: radiochemical yield
RMSD: root mean square deviation
RT: room temperature
Rt: retention time
SD: standard deviation
TACN: triazacyclononane
TEA: triethylamine
TFA: trifluoroacetic acid
THF: tetrahydrofuran
TLC: thin layer chromatography
TOF: time of flight.

## References

[1] Sgouros G., Bodei L., McDevitt M. R., Nedrow J. R. (2020). Radiopharmaceutical
therapy in cancer: clinical advances and challenges. Nat. Rev. Drug Discovery.

[2] Delbeke D., Segall G. M. (2011). Status of and Trends in Nuclear Medicine
in the United
States. J. Nucl. Med..

[3] Dondi M., Kashyap R., Paez D., Pascual T., Zaknun J., Bastos F. M., Pynda Y. (2011). Trends in
Nuclear Medicine in Developing
Countries. J. Nucl. Med..

[4] Garin E., Tselikas L., Guiu B., Chalaye J., Edeline J., de Baere T., Assenat E., Tacher V., Robert C., Terroir-Cassou-Mounat M. (2021). Personalised versus
standard dosimetry approach of selective internal radiation therapy
in patients with locally advanced hepatocellular carcinoma (DOSISPHERE-01):
a randomised, multicentre, open-label phase 2 trial. Lancet Gastroenterol. Hepatol..

[5] Baidoo K. E., Yong K., Brechbiel M. W. (2013). Molecular
Pathways: Targeted α-Particle
Radiation Therapy. Clin. Cancer Res..

[6] Hennrich U., Kopka K. (2019). Lutathera®: The
First FDA- and EMA-Approved Radiopharmaceutical
for Peptide Receptor Radionuclide Therapy. Pharmaceuticals.

[7] Kostelnik T. I., Orvig C. (2019). Radioactive Main Group and Rare Earth Metals for Imaging and Therapy. Chem. Rev..

[8] Wang Y., Chen D., dos Santos Augusto R., Liang J., Qin Z., Liu J., Liu Z. (2022). Production
Review of Accelerator-Based Medical Isotopes. Molecules.

[9] Umbricht C. A., Benešová M., Schmid R. M., Türler A., Schibli R., van der
Meulen N. P., Müller C. (2017). 44Sc-PSMA-617
for radiotheragnostics in tandem with 177Lu-PSMA-617—preclinical
investigations in comparison with 68Ga-PSMA-11 and 68Ga-PSMA-617. EJNMMI Res..

[10] Burkett B. J., Bartlett D. J., McGarrah P. W., Lewis A. R., Johnson D. R., Berberoğlu K., Pandey M. K., Packard A. T., Halfdanarson T. R., Hruska C. B. (2023). A Review of Theranostics: Perspectives on Emerging
Approaches and Clinical Advancements. Radiol.
Imaging Cancer.

[11] Hennrich U., Benešová M. (2020). [68Ga]­Ga-DOTA-TOC:
The First FDA-Approved
68Ga-Radiopharmaceutical for PET Imaging. Pharmaceuticals.

[12] Hennrich U., Eder M. (2021). [68Ga]­Ga-PSMA-11: The First FDA-Approved 68Ga-Radiopharmaceutical
for PET Imaging of Prostate Cancer. Pharmaceuticals.

[13] Miller C., Rousseau J., Ramogida C. F., Celler A., Rahmim A., Uribe C. F. (2022). Implications of physics, chemistry and biology for
dosimetry calculations using theranostic pairs. Theranostics.

[14] Eberlein U., Cremonesi M., Lassmann M. (2017). Individualized Dosimetry for Theranostics:
Necessary, Nice to Have, or Counterproductive?. J. Nucl. Med..

[15] Currie G. M., Rohren E. M. (2025). Sharpening the Blade of Precision
Theranostics. Semin. Nucl. Med..

[16] Currie G. (2025). Molecular
theranostics: principles, challenges and controversies. J. Med. Rad. Sci..

[17] Jacobson O., Kiesewetter D. O., Chen X. (2015). Fluorine-18 Radiochemistry, Labeling
Strategies and Synthetic Routes. Bioconjugate
Chem..

[18] Luu T. G., Kim H.-K. (2023). Recent progress
on radiofluorination using metals:
strategies for generation of C–18F bonds. Org. Chem. Front..

[19] Bernard-Gauthier V., Bailey J. J., Liu Z., Wängler B. r., Wängler C., Jurkschat K., Perrin D. M., Schirrmacher R. (2016). From unorthodox
to established: The current status of 18F-trifluoroborate-and 18F-SiFA-based
radiopharmaceuticals in PET nuclear imaging. Bioconjugate Chem..

[20] Liu T., Liu C., Xu X., Liu F., Guo X., Li N., Wang X., Yang J., Yang X., Zhu H. (2019). Preclinical Evaluation
and Pilot Clinical Study of Al(18)­F-PSMA-BCH
for Prostate Cancer PET Imaging. J. Nucl. Med..

[21] Heo Y.-A. (2023). Flotufolastat
F 18: Diagnostic first approval. Mol. Diagn.
Ther..

[22] Lepage M. L., Kuo H. T., Roxin A. ´., Huh S., Zhang Z., Kandasamy R., Merkens H., Kumlin J. O., Limoges A., Zeisler S. K. (2020). Toward ^18^F-Labeled Theranostics:
A Single Agent that Can Be Labeled with ^18^F, ^64^Cu, or ^177^Lu. ChemBioChem.

[23] Wängler C., Waser B., Alke A., Iovkova L., Buchholz H.-G., Niedermoser S., Jurkschat K., Fottner C., Bartenstein P., Schirrmacher R. (2010). One-step 18F-labeling of carbohydrate-conjugated
octreotate-derivatives containing a silicon-fluoride-acceptor (SiFA):
in vitro and in vivo evaluation as tumor imaging agents for positron
emission tomography (PET). Bioconjugate Chem..

[24] Whetter J. N., Vaughn B. A., Koller A. J., Boros E. (2022). An Unusual Pair: Facile
Formation and In Vivo Validation of Robust Sc–18F Ternary Complexes
for Molecular Imaging. Angew. Chem., Int. Ed..

[25] Whetter J. N., Śmiłowicz D., Becker K. V., Aluicio-Sarduy E., Kelderman C. A. A., Koller A. J., Glaser O. M., Marlin A., Ahn S. H., Kretowicz M. N. (2024). Phosphonate-Based Aza-Macrocycle
Ligands for Low-Temperature, Stable Chelation of Medicinally Relevant
Rare Earth Radiometals and Radiofluorination. J. Am. Chem. Soc..

[26] Vaughn B. A., Koller A. J., Chen Z., Ahn S. H., Loveless C. S., Cingoranelli S. J., Yang Y., Cirri A., Johnson C. J., Lapi S. E. (2021). Homologous Structural, Chemical, and Biological Behavior
of Sc and Lu Complexes of the Picaga Bifunctional Chelator: Toward
Development of Matched Theranostic Pairs for Radiopharmaceutical Applications. Bioconjugate Chem..

[27] Kelderman C. A. A., Glaser O. M., Whetter J. N., Aluicio-Sarduy E., Mixdorf J. C., Sanders K. M., Guzei I. A., Barnhart T. E., Engle J. W., Boros E. (2024). Charting the coordinative
landscape
of the ^18^F–Sc/^44^Sc/^177^Lu triad
with the tri-aza-cyclononane (tacn) scaffold. Chem. Sci..

[28] Vaughn B.
A., Ahn S. H., Aluicio-Sarduy E., Devaraj J., Olson A. P., Engle J., Boros E. (2020). Chelation with a twist: a bifunctional
chelator to enable room temperature radiolabeling and targeted PET
imaging with scandium-44. Chem. Sci..

[29] Eppard E., de la Fuente A., Benešová M., Khawar A., Bundschuh R. A., Gärtner F. C., Kreppel B., Kopka K., Essler M., Rösch F. (2017). Clinical Translation and First In-Human
Use of [44Sc]­Sc-PSMA-617 for PET Imaging of Metastasized Castrate-Resistant
Prostate Cancer. Theranostics.

[30] Tripier R., Platas-Iglesias C., Boos A., Morfin J.-F., Charbonnière L. (2010). Towards Fluoride
Sensing with Positively Charged Lanthanide Complexes. Eur. J. Inorg. Chem..

[31] McBride W. J., Sharkey R. M., Karacay H., D’Souza C. A., Rossi E. A., Laverman P., Chang C. H., Boerman O. C., Goldenberg D. M. (2009). A novel method of 18F radiolabeling for PET. J. Nucl. Med..

[32] Archibald S. J., Allott L. (2021). The aluminium-[(18)­F]­fluoride revolution:
simple radiochemistry
with a big impact for radiolabelled biomolecules. EJNMMI Radiopharm. Chem..

[33] D’Souza C. A., McBride W. J., Sharkey R. M., Todaro L. J., Goldenberg D. M. (2011). High-Yielding
Aqueous ^18^F-Labeling of Peptides via Al^18^F Chelation. Bioconjugate Chem..

[34] Dijkgraaf I., Franssen G. M., McBride W. J., D’Souza C. A., Laverman P., Smith C. J., Goldenberg D. M., Oyen W. J., Boerman O. C. (2012). PET of tumors expressing gastrin-releasing
peptide receptor with an 18F-labeled bombesin analog. J. Nucl. Med..

[35] Dijkgraaf I., Terry S. Y. A., McBride W. J., Goldenberg D. M., Laverman P., Franssen G. M., Oyen W. J. G., Boerman O. C. (2013). Imaging
integrin alpha-v-beta-3 expression in tumors with an 18F-labeled dimeric
RGD peptide. Contrast Media Mol. Imaging.

[36] Aime S., Botta M., Fasano M., Marques M. P. M., Geraldes C. F., Pubanz D., Merbach A. E. (1997). Conformational
and coordination equilibria
on DOTA complexes of lanthanide metal ions in aqueous solution studied
by 1H-NMR spectroscopy. Inorg. Chem..

[37] Yuk S. F., Sargin I., Meyer N., Krogel J. T., Beckman S. P., Cooper V. R. (2024). Putting error bars
on density functional theory. Sci. Rep..

[38] Sinnes J. P., Bauder-Wüst U., Schäfer M., Moon E. S., Kopka K., Rösch F. (2020). (68)­Ga, (44)­Sc
and (177)­Lu-labeled AAZTA(5)-PSMA-617:
synthesis, radiolabeling, stability and cell binding compared to DOTA-PSMA-617
analogues. EJNMMI Radiopharm. Chem..

[39] Nagy G., Szikra D., Trencsényi G., Fekete A., Garai I., Giani A. M., Negri R., Masciocchi N., Maiocchi A., Uggeri F. (2017). AAZTA:
An Ideal Chelating
Agent for the Development of ^44^Sc PET Imaging Agents. Angew. Chem., Int. Ed..

[40] Eiber M., Fendler W. P., Rowe S. P., Calais J., Hofman M. S., Maurer T., Schwarzenboeck S. M., Kratowchil C., Herrmann K., Giesel F. L. (2017). Prostate-Specific Membrane Antigen
Ligands for Imaging and Therapy. J. Nucl. Med..

[41] Lengacher R., Martin K. E., Śmiłowicz D., Esseln H., Lotlikar P., Grichine A., Maury O., Boros E. (2023). Targeted,
Molecular Europium­(III) Probes Enable Luminescence-Guided Surgery
and 1 Photon Post-Surgical Luminescence Microscopy of Solid Tumors. J. Am. Chem. Soc..

[42] Lengacher R., Cosby A. G., Śmiłowicz D., Boros E. (2022). Validation
of a post-radiolabeling bioconjugation strategy for radioactive rare
earth complexes with minimal structural footprint. Chem. Commun..

[43] Savisto N., Grönroos T. J., Oikonen V., Rajander J., Löyttyniemi E., Bergman J., Forsback S., Solin O., Haaparanta-Solin M. (2023). [18F]­Fluoride
uptake in various bone types and soft tissues in rat. EJNMMI Res..

[44] Segall G., Delbeke D., Stabin M. G., Even-Sapir E., Fair J., Sajdak R., Smith G. T. (2010). SNM Practice
Guideline
for Sodium ^18^F-Fluoride PET/CT Bone Scans 1.0. J. Nucl. Med..

[45] Bergmann R., Meckel M., Kubíček V., Pietzsch J., Steinbach J., Hermann P., Rösch F. (2016). (177)­Lu-labelled
macrocyclic bisphosphonates for targeting bone metastasis in cancer
treatment. EJNMMI Res..

[46] Chen Y., Pullambhatla M., Foss C. A., Byun Y., Nimmagadda S., Senthamizhchelvan S., Sgouros G., Mease R. C., Pomper M. G. (2011). 2-(3-{1-Carboxy-5-[(6-[18F]­fluoro-pyridine-3-carbonyl)-amino]-pentyl}-ureido)-pentanedioic
acid, [18F]­DCFPyL, a PSMA-based PET imaging agent for prostate cancer. Clin. Cancer Res..

[47] Stormezand G. N., Schreuder R., Brouwers A. H., Slart R., Elsinga P. H., Walenkamp A. M. E., Dierckx R., Glaudemans A., Luurtsema G. (2021). The effects of molar activity on [(18)­F]­FDOPA uptake
in patients with neuroendocrine tumors. EJNMMI
Res..

[48] Chen C. K. J., Gui X., Kappen P., Renfrew A. K., Hambley T. W. (2020). The effect
of charge on the uptake and resistance to reduction of platinum­(IV)
complexes in human serum and whole blood models. Metallomics.

[49] Scott P. J.
H., Hockley B. G., Kung H. F., Manchanda R., Zhang W., Kilbourn M. R. (2009). Studies
into radiolytic decomposition
of fluorine-18 labeled radiopharmaceuticals for positron emission
tomography. Appl. Radiat. Isot..

[50] Sowa A. R., Jackson I. M., Desmond T. J., Alicea J., Mufarreh A. J., Pham J. M., Stauff J., Winton W. P., Fawaz M. V., Henderson B. D. (2018). Futureproofing [(18)­F]­Fludeoxyglucose manufacture
at an Academic Medical Center. EJNMMI Radiopharm.
Chem..

[51] Ducharme J., Goertzen A. L., Patterson J., Demeter S. (2009). Practical Aspects of ^18^F-FDG PET When Receiving ^18^F-FDG from a Distant
Supplier. J. Nucl. Med. Technol..

[52] Mossine A. V., Brooks A. F., Ichiishi N., Makaravage K. J., Sanford M. S., Scott P. J. (2017). Development of Customized [(18)­F]­Fluoride
Elution Techniques for the Enhancement of Copper-Mediated Late-Stage
Radiofluorination. Sci. Rep..

[53] Decristoforo C., Knopp R., von Guggenberg E., Rupprich M., Dreger T., Hess A., Virgolini I., Haubner R. (2007). A fully automated synthesis
for the preparation of 68Ga-labelled peptides. Nucl. Med. Commun..

[54] Leece A. K., Rushford L. E., Stevens M., Heidari P., Collier T. L., Mahmood U. (2014). Design, construction, and testing
of an inexpensive
automated ^68^Ga-DOTATOC synthesis unit. J. Nucl. Med..

[55] Muller C., Bunka M., Haller S., Koester U., Groehn V., Bernhardt P., van der Meulen N., Tuerler A., Schibli R. (2014). Promising
prospects for 44Sc-/47Sc-based theragnostics: application of 47Sc
for radionuclide tumor therapy in mice. J. Nucl.
Med..

