# Supplementary material for: Evaluation of Bifunctional, PSMA-Targeted Triazamacrocycle-Picolinates Compatible with the 18F/44Sc/177Lu Isotope Triad
Source: J Med Chem. 2025 Dec 8;68(24):26234–45. doi: 10.1021/acs.jmedchem.5c02286 (PMC12751015; doi:10.1021/acs.jmedchem.5c02286)
Supplement: Supplementary file 1 [file jm5c02286_si_001.pdf]

Supporting Information for: Evaluation of Bifunctional, PSMA-targeted Triazamacrocycle-Picolinates Compatible with the $^{18}F/^{44}Sc/^{177}Lu$ Isotope Triad.

Owen M. Glaser,[a] Hannah Goerlach,[a] Megan V. Salek,[b] Eduardo Aluicio-Sarduy,[b] Mallory J. Gork,[a] Dariusz Śmiłowicz,[a] Edith Amason,[a] Jason C. Mixdorf,[b] Todd E. Barnhart,[b] Jonathan W. Engle[b,c] and Eszter Boros[a,] *

[a] Department of Chemistry, University of Wisconsin Madison, 1101 University Avenue, Madison, Wisconsin, 53705, United States

[b] Department of Medical Physics, University of Wisconsin-Madison, 1111 Highland Avenue, Madison, Wisconsin 53705, United States

[c] Department of Radiology, University of Wisconsin-Madison, Madison, Wisconsin 53705, United States

Corresponding author: Eszter Boros, email: eboros@wisc.edu

Table of Contents

Abbreviations 3
1 Experimental Procedures 5
1.1 General Methods 5
1.2 Ligand Synthesis 8
1.3 Coordination Complex Synthesis 16
1.3.1 Scandium Complexation 16
1.3.2 Lutetium Complexation 17
1.3.3 Scandium-Fluoride Ternary Complex Formation 18
2 Ligand and Complex Characterization 19
2.1 NMR 19
2.2 HRMS and LRMS 48
3 Thermodynamic Speciation 56
3.1 Speciation of $[ScF(mpatcn)]^-$ 56
3.2 Speciation of [ScF(mpatcn-am-Bz)] 62
4 Computational Chemistry 68
4.1 DFT Parameters 68
4.2 Bond Angle Calculations 68
4.3 DFT Coordinates 68
4.4 Bond Angle VS %RCY 72
5 Radiolabeling Data 73
5.1 General ^{18}F Radiolabeling Procedure 73
5.2 General $^{44}Sc/^{177}Lu$ Radiolabeling Procedure 73
5.3 Acid Washing of Glassware for ^{18}F Studies 73
5.4 ^{18}F In Vivo Biodistribution 73
5.5 $^{44}Sc/^{177}Lu$ In Vivo Biodistribution 73

5.6 Metabolite Analysis 74
5.7 PET/CT 74
5.8 Tabulated Biodistribution Data 78
5.9 Tumor Blood Ratio Calculations 80
5.10 HPLC Chromatographs 81
5.11 Radiolysis Stability 91
5.12 QMA Cartridge Purification of ^{18}F Conjugates 93
5.13 Displacement assay 93
6 References 94

Abbreviations

Ac	Acetate
AcN	Acetonitrile
API-ES	Atmospheric Pressure Ionization - Electrospray
Ar	Aromatic
Ar(Bz)	Aromatic Benzyl
Ar(Pa)	Aromatic Picolinic Acid
Asc	Ascorbate
Bz	Benzyl
CV	Column Volume
DCM	Dichloromethane
DFT	Density Functional Theory
DIPEA	N,N-diisopropylethylamine
DMAP	4-dimethylaminopyridine
DMEM	Dulbecco's Modified Eagle Medium
DMF	Dimethylformamide
DMSO	Dimethyl sulfoxide
ESI	Electrospray Ionization
EtOAc	Ethyl Acetate
FBS	Fetal Bovine Serum
HEPES	2-[4-(2-hydroxyethyl)piperazin-1-yl]ethanesulfonic acid
HPLC	High Performance Liquid Chromatography
HR	High Resolution
Hz	Hertz
ICP-OES	Inductively Coupled Plasma - Optical Emission Spectroscopy
IS	Internal Standard
LC	Liquid Chromatography
LR	Low Resolution
MeCN	Acetonitrile
MeOH	Methanol
MS	Mass Spectrometry
NMR	Nuclear Magnetic Resonance
PBS	Phosphate Buffered Saline
PVDF	Polyvinylidene Fluoride
QTAIM	Quantum Theory of Atoms in Molecules
RCY	Radiochemical Yield
RMSD	Root Mean Square Deviation
RT	Room Temperature
R_t	Retention time
SD	Standard Deviation

TACN	Triazacyclononane
TEA	Triethylamine
TFA	Trifluoroacetic acid
THF	Tetrahydrofuran
TLC	Thin Layer Chromatography
TOF	Time of Flight

1 Experimental Procedures

1.1 General Methods

All starting materials were purchased from Acros Organics, Alfa Aesar, Sigma Aldrich, or TCI America and used without further purification.

NMR spectra: All proton ^{1}H nuclear magnetic resonance spectra were recorded on a 400 or 500 MHz Bruker or 600 MHz Avance III Bruker spectrometers. All carbon ^{13}C {^{1}H} nuclear magnetic resonance spectra were recorded on a 101 or 125MHz Bruker or 175 MHz Avance III Bruker NMR spectrometer. Spectra were collected at 25 °C and processed using MestReNova. Chemical shifts are expressed in parts per million (ppm, δ scale) and are referenced to residual $CDCl_3$ (^{1}H: δ 7.26 ppm, ^{13}C: δ 77.1 ppm), CD_3OD (^{1}H: δ 3.31 ppm, ^{13}C: δ 49.0 ppm), and $(CD_3)_2SO$ (^{1}H: δ 2.50 ppm, ^{13}C: δ 39.5 ppm).[1] Data is presented as follows: chemical shift, multiplicity (s = singlet, d = doublet, t = triplet, q = quartet, m = multiplet, and bs = broad singlet), integration, and coupling constant in hertz (Hz).

Mass spectrometry: Low-resolution electrospray ionization (ESI) mass spectrometry and high-resolution (ESI) mass spectrometry were carried out at the UW-Madison Chemistry Paul Bender Chemical Instrumentation Center Facility, with a Thermo Q ExactiveTM Plus (Electrospray Ionization-Quadrupole-Ion Trap) mass spectrometer.

MALDI TOF analysis: 50µl of the sample (estimated ~5µg of compound) was acidified with TFA (to 0.2% final) and SPE desalted with Pierce (Thermo Scientific) 100µl capacity C18 tips according to manufacturer protocol then eluted with 25µl of 70:30:0.2 ACN:water:formic acid. 0.5µl was spotted onto Opti-TOF™ 384 well plate (Applied Biosystems, Foster City, CA) and re-crystalized with 0.5µl of matrix [10mg/ml of α-Cyano-4-Hydroxycinnamic acid in acetonitrile/H2O/TFA (70/30/0.1)]. Mass spectrum was acquired on a 4800 Matrix-Assisted Laser Desorption/Ionization-Time of Flight-Time of Flight (MALDI TOF-TOF) mass spectrometer (Applied Biosystems) scanning from 700 to 4,000Da range using 1,000 shots acquired from 20 randomized regions of the sample spot at 3,000 fixed laser intensity and 2.059 kV Detector Voltage of OptiBeam™ on-axis Nd:YAG laser with 200Hz firing rate and 3 to 7ns pulse width in MS Reflector Positive Ion Mode. External calibration with six mass reference standards was performed to validate mass accuracy. Peak mass annotation was performed with DataExplorer® software version 4.9 [build 115] (Applied Biosystems) with the following peak detection settings: % Cetroid: 50, Noise window width: 250, S/N threshold: 3, Threshold after S/N recalculation: 10, Integration Baseline settings: Valley to Baseline.

UV-vis spectra: Data was collected with a NanoDrop 1C instrument (AZY1706045) and spectra were recorded from 190 to 850 nm in a quartz cuvette with 1 cm path length.

High Performance Liquid Chromatography: Analytical HPLC analysis was carried out using a Shimadzu HPLC-20AR equipped with an autoinjector, binary gradient pump, Phenomenex Luna 5 µm C18(2) column (150 mm × 3 mm), and a UV-vis detector. RadioHPLC was carried out using Agilent 1220 Infinity II LC system equipped with a binary gradient pump, autoinjector, a Phenomenex Luna 5 µm C18(2) column (150 mm × 3 mm), UV–vis detector, and a LabLogic radio detector.

LCMS: Low resolution liquid chromatography - mass spectrometry (LC-MS) was carried out on a Phenomenex Luna 5 µm C18 column (150 mm × 3 mm, 100 Å, AXIA packed) at a flow rate of 0.8 mL/min using a single quadrupole Agilent 1200 Infinity II LC/MSD system equipped with a binary gradient pump, UV-vis detector, automatic injector, and an atmospheric pressure electrospray ionization (API-ES) source. UV absorption was recorded at 254 nm, both positive

and negative mass spectra were collected. Purity of all intermediates and final products, including radiochemical species, was determined using analytical HPLC. All conjugates and complexes were ≥ 95% pure.

Inductively Coupled Plasma- Optical Emission Spectroscopy (ICP-OES): Metal ion concentrations were determined with an Agilent 5110 ICP-OES. A 10-point standard calibration curve with respect to scandium or lutetium was used and a line of best fit was found with an R^2 of 0.999.

PET/CT Imaging: Positron emission tomography/computed tomography (PET/CT) imaging was performed using either a Siemens Inveon Hybrid MicroPET/CT Scanner (Siemens Medical Solutions USA, Inc., Knoxville, TN) or a Mediso nanoScan microPET/CT Scanner (Mediso, Budapest, Hungary). Mice were anesthetized with 4% isoflurane gas and anesthesia was maintained during scans at 2% isoflurane in oxygen. CT scans were acquired prior to PET scans for anatomical coregistration as well as attenuation correction. CT scan parameters were as follows: 220 rotation degrees, 120 rotation steps, binning factor of 4, exposure time of 250 ms, x-ray energy of 80 kVp, 1 mA current, and 105 μm resolution. PET scans were acquired with 40 million coincidence events per mouse, an energy window of 350-650 keV, and a timing window of 3.432 ns. Quantification of PET/CT images was performed in an Inveon Research Workstation and data are expressed as percent injected dose per gram of tissue (% ID/g).

SPECT/CT Imaging: Mice were inducted with 4% isoflurane gas anesthesia, placed on a heated bed with 2% maintenance and subsequently scanned on an MILabs U-SPECT6CTUHR micro-single photon emission computed tomography/computed tomography (μSPECT/CT) system (MILabs; Houten, The Netherlands). SPECT data were acquired using the General Purpose Rat/Mouse collimator (GP-RM; 1.5mm pinholes) with 4 frames in spiral scan mode using normal steps for a total of 40 minutes of effective acquisition time. SPECT data were reconstructed based on the 114 keV and 208 keV photopeaks with a 20% window of the photopeak, and background subtraction using the automatic triple energy windows (8.3 keV and 4.6 keV above/below the 20% width of the photopeak for the 208 keV and 114ke V photopeaks, respectively) into one static image with 0.4 mm isotropic voxels, SROSEM reconstruction with 128 subsets and 5 iterations, and a 1.4 mm full width half max (FWHM) smoothing filter.

Chromatography Solvent Systems:

Analytical HPLC (Method A): binary solvent system (A: water + 0.1% TFA; B: MeCN + 0.1% TFA); gradient (0–2 min: 5% B; 2–14 min: 5–95% B; 14–16 min: 95% B; 16–16.5 min: 95–5% B; 16.5–20 min 5% B); flow rate: 0.8 mL/min; column: Phenomenex Luna C18 column (5 μm, 150 mm × 3 mm, 100 Å, AXIA packed).

Analytical HPLC (Method B): binary solvent system (A: 10 mM ammonium formate pH 7.4; B: MeCN); gradient (0–2 min: 0% B; 2–14 min: 0–90% B; 14–16 min: 90% B; 16–16.5 min: 90–0% B; 16.5–20 min 0% B); flow rate: 1.0 mL/min; temperature: 50 °C; column: Restek Ultra AQ C18 column (5 μm, 250 mm × 3 mm).

Analytical HPLC (Method C): binary solvent system (A: water + 0.1% TFA; B: MeCN + 0.1% TFA); gradient (0–2 min: 0% B; 2–14 min: 0–90% B; 14–16 min: 90% B; 16–16.5 min: 90–0% B; 16.5–20 min 0% B); flow rate: 1.0 mL/min; temperature: 50 °C; column: Restek Ultra AQ C18 column (5 μm, 250 mm × 3 mm).

LCMS analysis (Method D):): binary solvent system (A: water + 0.1% FA; B: MeCN + 0.1% FA); gradient (0–3 min: 5% B; 3–10min: 5–95% B; 10–12 min: 95% B; 12–12.5 min: 95–5% B;

12.5–16 min 5% B); flow rate: 0.8 mL/min; column: Phenomenex Luna C18 column (5 µm, 150 mm × 3 mm, 100 Å, AXIA packed).

Semi-Preparative HPLC (Method E): Binary solvent system (A = 0.1% TFA in water, B = 0.1% TFA in MeCN); gradient (0-5 min, 5% B; 5-24 min, 5-95% B; 24-27 min, 95% B; 27-27.5 min, 95-5% B; 27.5-30 min, 5% B); flowrate: 10 mL/min; column: Phenomenex Luna C18 column (10 µm, 250x10 mm, 100 Å, AXIA packed).

1.2 Ligand Synthesis

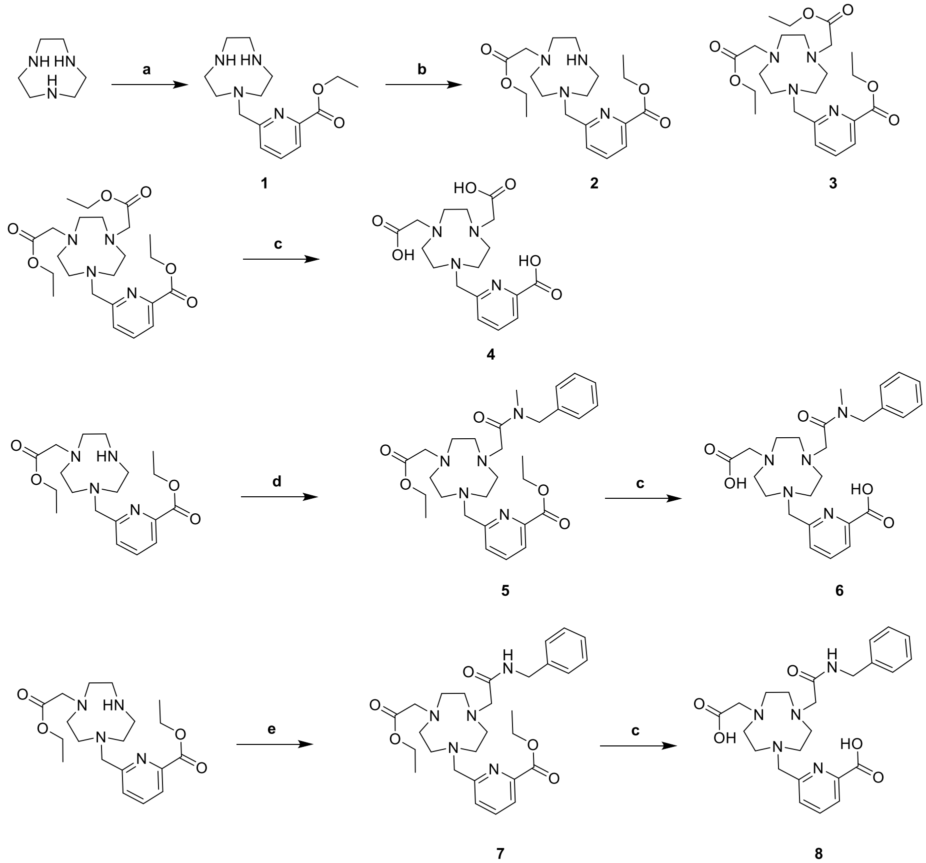

Scheme S1. Synthesis of monofunctionalized ligands discussed in this work, mpatcn, mpatcn-am-Bz, and mpatcn-am-Bz-p. a) K_2CO_3, ethyl 6-(bromomethyl)picolinate, AcN. b) K_2CO_3, ethyl 2-bromoacetate, AcN. c) 6 M HCl 100 °C. d) K_2CO_3, N-benzyl-2-bromo-N-methylacetamide, AcN. e) K_2CO_3, N-benzyl-2-bromoacetamide, AcN.

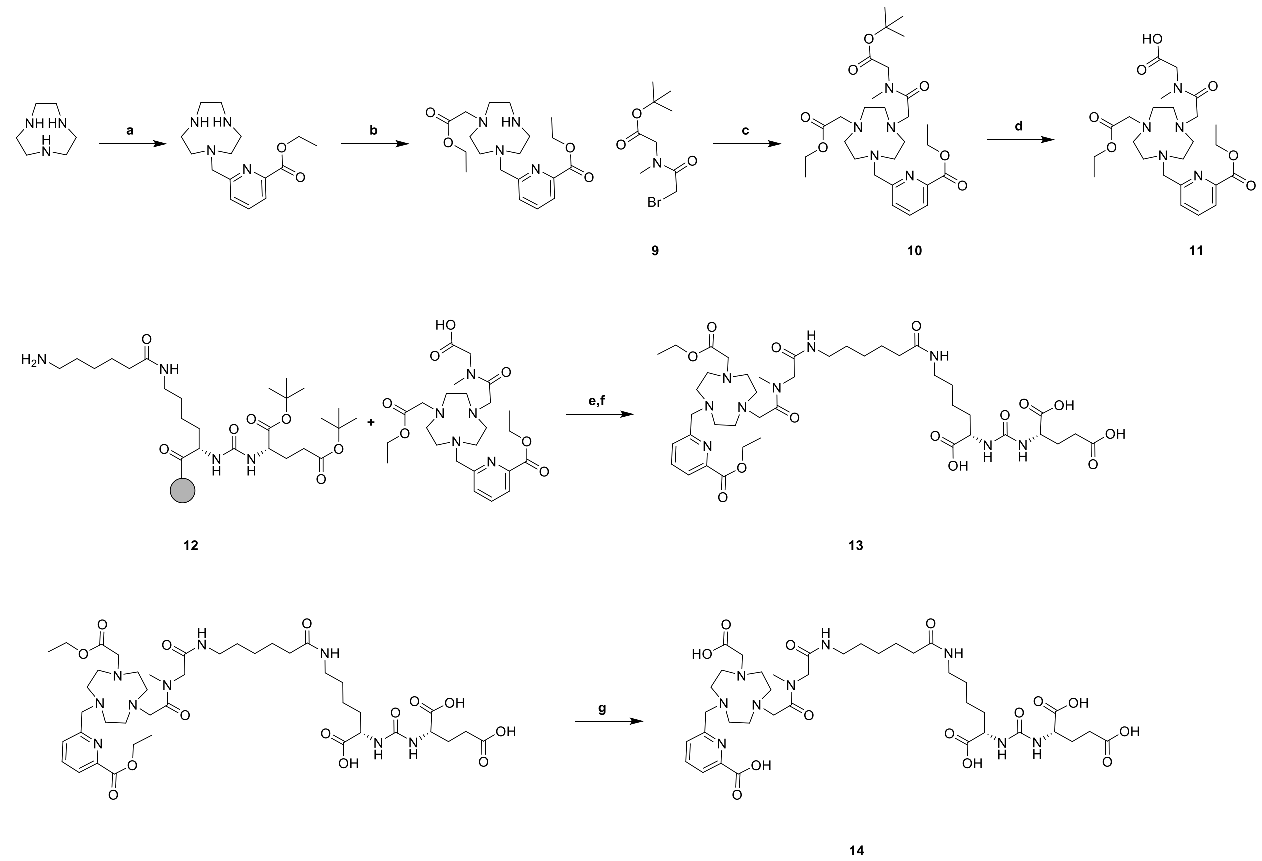

Scheme S2. Synthesis of **mpatcn-am-hex-KuE (14).** a) K_2CO_3, ethyl 6-(bromomethyl)picolinate, AcN. b) K_2CO_3, ethyl 2-bromoacetate, AcN. c) K_2CO_3, AcN. d) TFA:DCM 1:1. e) PyBOP, DIPEA, DMF. f) TFA:TIS:H_2O 95:2.5:2.5. g) 1 M KOH.

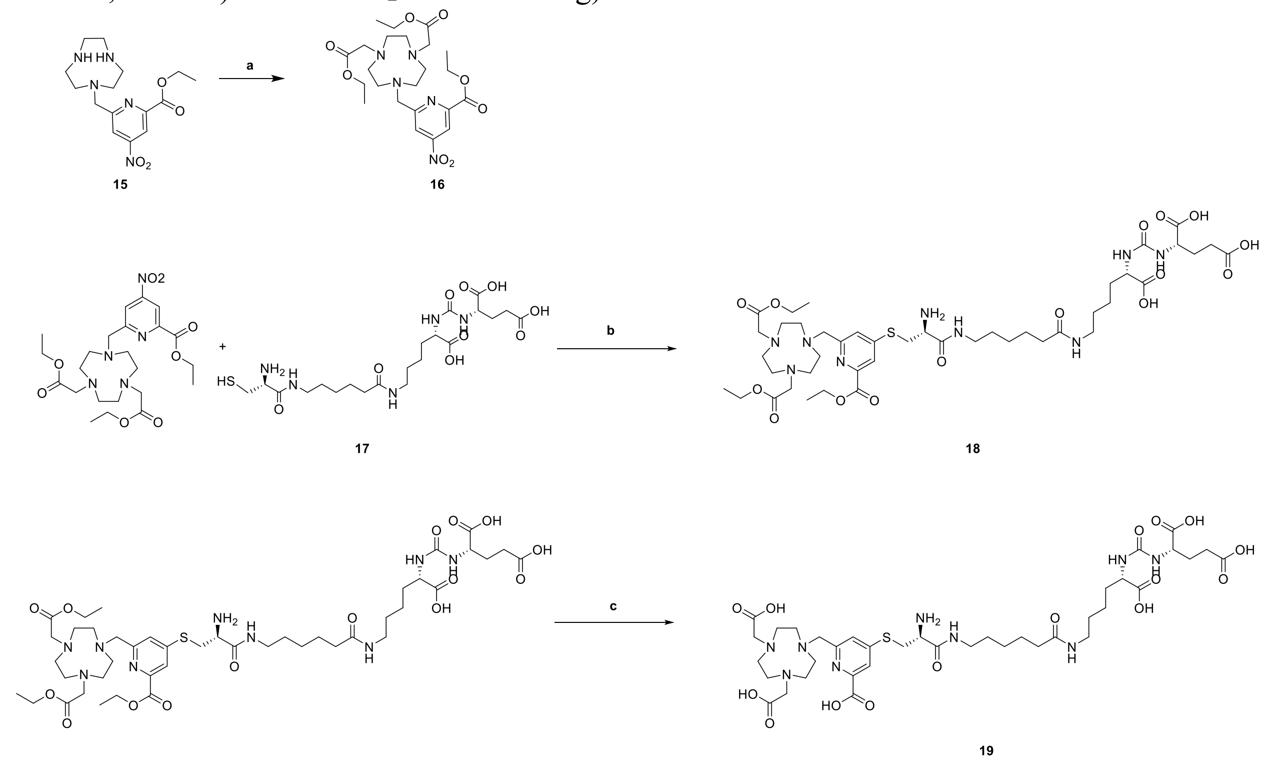

Scheme S3. Synthesis of **mpatcn-S-Cys-hex-KuE (19).** a) K_2CO_3, ethyl 2-bromoacetate, AcN. b) NH_4HCO_3:DMF 1:3, TCEP. c) 1 M KOH.

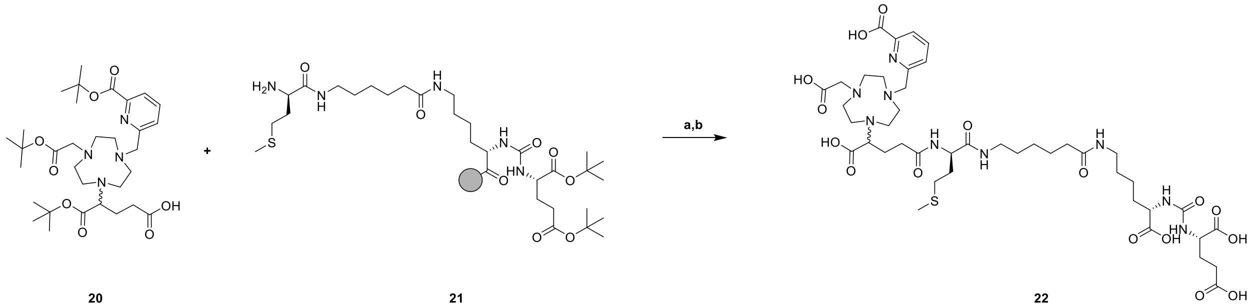

20 21 22

Scheme S4. Synthesis of **picaga-Met-hex-KuE (22).** a) PyBOP, DIPEA, DMF. b) TFA:TIS:H_2O 95:2.5:2.5.

Compounds **1-6** were synthesized according to literature.[1]

Synthesis of (7). 2 (27.7 mg, 73.2 µmol, 1 equiv.) and potassium carbonate (30.3 mg, 3.0 Eq, 220 µmol, 3 equiv.) was dissolved in 5 mL of dry AcN. N-benzyl-2-bromoacetamide (11.7 mg, 51.2 µmol, 0.7 equiv) was added. The combined solution was allowed to stir for 1 hour at rt under an N_2 atmosphere. After 1 hour, the solution was filtered, the excess solvent was removed, the solution was redissolved in 1 mL AcN and filtered to remove any trace K_2CO_3. 1 mL of water was added to the solution, and it was then purified using automatic reverse-phase combiflash (0.1% TFA H_2O:AcN gradient, 100 g column). The desired product eluted with a retention time of 10 minutes. Pure fractions were pooled and lyophilized to yield a waxy brown product (5.5 mg, 73.2 µmol, 14 % yield). ^{1}H NMR (500 MHz, MeOD): δ 8.14 (dd, *J* = 7.9, 1.1 Hz, 1H, Ar(Pa)), 8.01 (t, *J* = 7.7 Hz, 1H, Ar(Pa)), 7.75 (dd, *J* = 7.8, 1.0 Hz, 1H, Ar(Pa)), 7.38 – 7.21 (m, 6H, Ar(Bz)), 7.20 – 7.12 (m, 2H Ar(Bz)), 4.58 – 4.34 (m, 8H, CH_2), 4.13 (q, *J* = 7.1 Hz, 4H, CH_2), 3.37 (m, 2H, CH_2), 3.33 (m, 2H, TACN), 3.19 – 3.02 (m, 11H, TACN), 1.43 (t, *J* = 7.1 Hz, 3H, CH_3), 1.24 (t, *J* = 7.1 Hz, 3H, CH_3). ^{13}C NMR (126 MHz, MeOD): δ 170.82, 169.35, 168.02, 164.57, 159.68, 159.37, 159.06, 158.75, 154.27, 147.48, 138.91, 138.38, 138.03, 128.23, 128.18, 127.69, 127.23, 127.17, 126.99, 126.96, 124.75, 119.00, 116.71, 115.92, 115.57, 114.43, 112.14, 63.68, 62.26, 61.90, 60.62, 60.13, 58.59, 58.01, 54.88, 51.18, 50.89, 50.55, 50.08, 49.39, 43.15, 42.61, 27.33, 19.45, 13.18, 13.13, 13.07, 13.05. ESI-HR-MS. Calcd for $C_{28}H_{39}N_5O_5$ $[M+H]^+$: m/z 526.3024. Found 526.3025.

Synthesis of mpatcn-am-Bz-p (8). 7 (5.5 mg, 73.2 µmol, 1 equiv.) was dissolved in 1 mL of 0.1 M KOH and allowed to stir for 3 hours. Following this, excess solvents were removed to give the crude product in quantitative yield. ^{1}H NMR (500 MHz, MeOD): δ 7.94 (d, *J* = 7.7 Hz, 1H, Ar(Pa)), 7.80 (t, *J* = 7.7 Hz, 1H, Ar(Pa)), 7.40 – 7.21 (m, 9H, Ar(Pa) + Ar(Bz)), 4.45 (d, *J* = 3.1 Hz, 1H), 4.36 (s, 2H, CH_2), 3.83 (s, 2H, CH_2), 3.18 (s, 2H, CH_2), 3.09 (s, 2H, CH_2), 2.69 (t, *J* = 5.3 Hz, 2H, CH_2), 2.66 – 2.62 (m, 3H, TACN), 2.61 – 2.57 (m, 4H, TACN), 2.47 (s, 2H, TACN), 2.36 (s, 2H, TACN). ^{13}C NMR (126 MHz, MeOD): δ 177.89, 171.83, 169.02, 162.08, 161.81, 161.54, 161.26, 158.09, 138.50, 137.42, 128.19, 128.15, 128.13, 127.39, 127.16, 127.12, 126.88, 126.82, 122.26, 120.37, 118.04, 115.71, 113.38, 70.12, 62.17, 61.85, 52.96, 48.11, 48.06, 47.94, 47.77, 47.73, 47.61, 47.60, 47.56, 47.43, 47.33, 47.26, 47.09, 42.69, 42.12. ESI-HR-MS. Calcd for $C_{24}H_{31}N_5O_5$ $[M-H]^-$: m/z 468.2252. Found 468.2252.

Compound **9** was synthesized according to literature.[2]

Synthesis of ethyl 6-((4-(2-((2-(tert-butoxy)-2-oxoethyl)(methyl)amino)-2-oxoethyl)-7-(2-ethoxy-2-oxoethyl)-1,4,7-triazonan-1-yl)methyl)picolinate (10). **2** (57 mg, 0.15 mmol, 1 equiv.) was dissolved in dry AcN. K_2CO_3 (62 mg, 0.45 mmol, 3 equiv.) was added and the solution was stirred to prevent sticking to the side of the RBF. **9** (28 mg, 0.11 mmol, 0.7 equiv.) was added to the solution. The combined solution was allowed to stir for 1 hour at rt under an N_2 atmosphere. After 1 hour, the solution was filtered, the excess solvent was removed, the solution was redissolved in 1 mL AcN and filtered to remove any trace K_2CO_3. 1 mL of water was added to the solution, and it was then purified using automatic reverse-phase combiflash (0.1% TFA H_2O:AcN gradient, 100 g column). The desired product eluted with a retention time of 8.5 minutes. Pure fractions were pooled and lyophilized to yield a waxy brown product (30.1 mg, 0.15 mmol, 35% yield). ^{1}H NMR (500 MHz, MeOD): δ = 8.01 (ddd, J = 8.9, 7.7, 1.0 Hz, 1H), 7.91 (td, J = 7.8, 5.2 Hz, 1H), 7.68 (td, J = 7.4, 1.1 Hz, 1H), 4.42 – 4.33 (m, 2H), 4.29 (d, J = 4.4 Hz, 2H), 4.22 (s, 1H), 4.11 – 3.90 (m, 7H), 3.82 (s, 1H), 3.50 (s, 2H), 3.40 (s, 1H), 3.25 (s, 7H), 3.15 (t, J = 5.6 Hz, 2H), 3.14 – 2.98 (m, 7H), 2.93 (s, 2H), 2.82 (s, 1H), 1.39 (s, 1H), 1.38 – 1.30 (m, 15H), 1.14 (td, J = 7.1, 5.7 Hz, 3H). ^{13}C NMR (126 MHz, MeOD) δ = 170.8, 170.7, 169.8, 169.1, 168.4, 168.4, 168.2, 168.2, 164.7, 164.6, 160.3, 160.0, 159.7, 159.4, 155.7, 155.3, 147.7, 147.5, 138.6, 138.5, 127.4, 127.4, 124.4, 124.3, 119.4, 117.1, 114.8, 82.5, 81.8, 81.8, 78.0, 61.8, 61.7, 60.5, 60.5, 59.3, 59.1, 55.6, 54.9, 54.8, 54.7, 53.4, 51.8, 51.5, 51.1, 51.0, 50.7, 50.6, 50.4, 50.3, 50.2, 50.0, 49.2, 49.2, 48.5, 48.1, 47.9, 47.7, 40.7, 40.6, 35.8, 34.7, 33.8, 33.3, 26.9, 26.9, 26.9, 26.8, 25.3, 24.6, 13.2, 13.1, 13.1.ESI-HR-MS. Calcd for $C_{28}H_{48}N_5O_7$ [M+H]$^+$: m/z 564.3319. Found 564.3389.

Synthesis of N-(2-(4-(2-ethoxy-2-oxoethyl)-7-((6-(ethoxycarbonyl)pyridin-2-yl)methyl)-1,4,7-triazonan-1-yl)acetyl)-N-methylglycine (11). **4** (30.1 mg, 0.15 mmol) was dissolved in 5 mL of 1:1 DCM:TFA and allowed to stir for 1 hour. Excess solvent was removed to give the intermediate product in quantitative yield. ^{1}H NMR (500 MHz, MeOD): δ = 8.01 (dt, J = 7.8, 1.4 Hz, 2H), 7.92 (td, J = 7.8, 0.9 Hz, 2H), 7.69 (ddd, J = 9.0, 7.8, 1.1 Hz, 2H), 4.37 (qd, J = 7.1, 5.6 Hz, 4H), 4.30 (d, J = 3.5 Hz, 4H), 4.24 (s, 1H), 4.12 (d, J = 7.3 Hz, 1H), 4.10 – 3.95 (m, 12H), 3.86 (s, 1H), 3.56 – 3.52 (m, 3H), 3.46 (s, 1H), 3.20 – 2.95 (m, 12H), 3.05 (s, 2H), 2.94 (s, 4H), 2.88 (s, 1H), 2.84 (s, 2H), 1.33 (t, J = 7.1 Hz, 6H), 1.24 – 1.09 (m, 7H). ^{13}C NMR (126 MHz, MeOD): δ = 170.9, 170.7, 170.1, 164.8, 155.5, 147.5, 147.4, 138.6, 127.5, 127.3, 124.4, 124.3, 61.8, 61.8, 60.6, 60.5, 59.3, 59.1, 55.6, 55.1, 55.0, 54.8, 51.5, 50.9, 50.7, 50.4, 50.1, 49.7, 49.3, 49.2, 49.0, 48.7, 48.0, 40.8, 40.6, 35.6, 34.5, 34.3, 33.8, 33.3, 24.6, 13.1, 13.1. ESI-HR-MS. Calcd for $C_{24}H_{37}N_5O_7$ [M+H]$^+$: m/z 508.2693. Found 508.2764

Compound **12** was synthesized according to literature.[3]

Synthesis of protected mpatcn-am-hex-KuE (13). The resin bearing **12** was loaded onto a syringe and swelled in DCM (2 mL) and shook for one minute (5 x), then washed in DMF (5 x). **11** (15.0 mg, 29.6 µmol, 1 equiv.) was dissolved in 1 mL DMF. Following this PyBOP (15.4 mg, 29.6 µmol, 1 equiv.) and DIPEA (30.9 µL, 177 µmol, 6 equiv.) was added and the solution was allowed to stir for 10 minutes. The solution was loaded onto a syringe containing **12** (60 mg, 0.010 mmol, 3.5 equiv.) on resin. The syringe was shaken overnight, before the excess solvent was removed. The syringe was then loaded with TFA:TIS:H_2O (95:2.5:2.5) and shaken for 5 hours before the solution was eluted off the syringe. Excess solvent was removed, and the crude product was purified by semi-preparative reverse-phase HPLC (Method **E**). Pure fractions were pooled and lyophilized to yield a white powder (10.1 mg, 9.4 µmol, 32% yield). ^{1}H NMR (500 MHz, MeOD) δ: 7.89 – 7.85 (m, 1H), 7.83 – 7.76 (m, 1H), 7.71 (t, *J* = 7.1 Hz, 1H), 4.13 (ddd, *J* = 14.6, 8.0, 5.0 Hz, 2H), 4.02 (d, *J* = 2.8 Hz, 2H), 3.92 (s, 2H), 3.57 (d, *J* = 8.7 Hz, 3H), 3.39 (s, 3H), 3.35 (t, *J* = 1.7 Hz, 1H), 3.11 (s, 11H), 2.99 (s, 2H), 2.90 (s, 3H), 2.80 (s, 1H), 2.29 (dt, *J* = 9.1, 6.1 Hz, 2H), 2.12 – 1.97 (m, 3H), 1.81 (dq, *J* = 14.8, 7.9 Hz, 1H), 1.72 (dt, *J* = 13.5, 6.1 Hz, 1H), 1.54 (dp, *J* = 29.4, 7.8 Hz, 3H), 1.42 (t, *J* = 6.8 Hz, 6H), 1.33 (q, *J* = 7.4 Hz, 2H), 1.23 (d, *J* = 13.4 Hz, 3H), 1.19 (s, 2H). ESI-HR-MS. Calcd for $C_{42}H_{67}N_9O_{14}$ $[M+H]^+$: m/z 922.4807. Found 922.4866.

Synthesis of mpatcn-am-hex-KuE (14). 13 (3.0 mg, 2.8 µmol, 1 equiv.) was dissolved in 1 mL of 1 M KOH and allowed to stir overnight. Excess solvent was removed, and the crude product was purified by semi-preparative reverse-phase HPLC (Method **E**). Pure fractions were pooled and lyophilized to yield a white powder (1.0 mg, 1 µmol, 40% yield). ^{1}H NMR (500 MHz, MeOD): δ: 7.99 (t, *J* = 7.3 Hz, 1H), 7.89 (td, *J* = 7.8, 5.0 Hz, 1H), 7.77 (dd, *J* = 15.2, 7.6 Hz, 1H), 4.46 (s, 3H), 4.36 (q, *J* = 7.1 Hz, 2H), 4.23 (s, 1H), 4.19 (s, 1H), 4.10 – 4.00 (m, 3H), 3.96 (t, *J* = 7.1 Hz, 4H), 3.41 (s, 2H), 3.19 – 3.08 (m, 2H), 2.93 (d, *J* = 7.8 Hz, 6H), 2.85 (s, 1H), 2.26 (dt, *J* = 11.3, 5.9 Hz, 2H), 2.08 (q, *J* = 7.0 Hz, 3H), 2.00 (s, 1H), 1.90 – 1.79 (m, 1H), 1.70 (s, 2H), 1.51 (dt, *J* = 14.6, 6.9 Hz, 5H), 1.42 (s, 5H), 1.32 (t, *J* = 7.1 Hz, 4H), 1.24 – 1.11 (m, 14H), 0.80 (t, *J* = 6.8 Hz, 1H). ESI-HR-MS. Calcd for $C_{38}H_{59}N_9O_{14}$ $[M+H]^+$: m/z 866.4254. Found 866.4255.

Compound **15** was synthesized according to literature.[4]

Synthesis of diethyl 2,2'-(7-((6-(ethoxycarbonyl)-4-nitropyridin-2-yl)methyl)-1,4,7-triazonane-1,4-diyl)diacetate (16). 15 (144 mg, 427 µmol, 1 equiv.) and potassium carbonate (177 mg, 3.00 Eq, 1.28 mmol) were dissolve in dry acetonitrile (4 mL). Ethyl 2-bromoacetate (143 mg, 95.0 µL, 2.01 equiv., 856 µmol) was added and the mixture was stirred under N2 for 2 hours. Excess solvent was removed and the crude product was purified by semi-preparative reverse-phase HPLC. Pure fractions were pooled and lyophilized to yield a brown powder (165 mg, 323 µmol, 75.7% yield). ^{1}H NMR (500 MHz, CD_2Cl_2) δ: 12.10 (s, 1H), 8.76 (s, 1H), 8.63 – 8.58 (m, 1H), 8.36 (s, 1H), 4.52 – 4.43 (m, 2H), 4.27 (s, 2H), 4.18 – 4.05 (m, 5H), 3.80 – 3.71 (m, 1H), 3.53 (s, 4H), 3.42 (d, *J* = 10.1 Hz, 1H), 3.10 – 2.99 (m, 11H), 2.84 – 2.75 (m, 1H), 1.44 (td, *J* = 7.1, 2.6 Hz, 4H), 1.24 (t, *J* = 7.1 Hz, 6H). ^{13}C NMR (126 MHz, CD_2Cl_2) δ: 172.93, 172.87, 172.41, 167.16, 166.10, 165.96, 164.87, 164.72, 164.66, 156.85, 156.78, 156.58, 151.93, 151.66, 120.60, 119.55, 119.25, 117.64, 117.40, 117.34, 64.19, 64.09, 63.88, 63.81, 63.77, 63.31, 61.96, 61.72, 61.66, 59.65, 59.13, 59.11, 58.97, 58.28, 57.47, 57.06, 56.84, 55.59, 55.40, 55.34, 53.07, 52.15, 51.68, 51.51, 15.40, 15.38, 15.35, 15.33.ESI-HR-MS Calcd for $C_{23}H_{35}N_5O_8$ $[M+H]^+$: m/z 510.2558. Found 510.2554

Cys-KuE (17) The resin bearing **12** (200 mg, 0.16 mmol, 1 equiv.) was swelled in DCM (2 mL) and shook for one minute (5 x), then washed in DMF (5 x). Next, Fmoc-Cys(trt)-OH (374.85 mg, 0.64 mmol, 4 equiv.), PyBOP (166.5 mg, 0.32 mmol, 2 equiv.), and DIPEA (0.112 mL, 0.64 mmol, 4 equiv.) were added to the resin. The reaction shook overnight at room temperature. Following the reaction, the resin was washed with DMF (5 x). Next, the Fmoc group was removed by shaking the resin with 20% piperidine in DMF (2 mL) for 20 minutes (2 x). After, the resin was washed with DMF (5 x), DCM (5 x), and Et2O (5 x), then dried in a vacuum for 10 mins. The syringe was then loaded with TFA:TIS:H_2O (95:2.5:2.5) and shaken for 5 hours before the solution was eluted off the syringe. Excess solvent was removed, and the crude product was purified by semi-preparative reverse-phase HPLC (Method **E**). Pure fractions were pooled and lyophilized to yield a white powder. ^{1}H NMR (500 MHz, D_2O): δ 4.79 (s, 14H), 4.20 – 4.08 (m, 1H), 3.23 (tt, *J* = 11.2, 6.7 Hz, 1H), 3.14 – 3.02 (m, 1H), 2.52 – 2.43 (m, 1H), 2.27 (t, *J* = 7.2 Hz, 1H), 1.75 – 1.57 (m, 1H), 1.57 (dd, *J* = 9.4, 6.5 Hz, 1H), 1.46 – 1.30 (m, 1H). ^{13}C NMR (126 MHz, D_2O) δ 179.48, 178.73, 176.85, 167.75, 159.25, 54.73, 54.64, 54.40, 39.54, 39.15, 35.70, 31.71, 31.14, 27.97, 27.93, 27.87, 25.49, 25.06, 25.01, 22.50. ESI-HR-MS. Calcd for $C_{13}H_{37}N_5O_9S$ $[M+H]^+$: m/z 536.2312. Found 536.2406.

Synthesis of protected mpatcn-S-Cys-hex-KuE (18). 16 (2 mg, 4 μmol, 1 equiv.) was dissolved in 1 mL of a degassed 1:3 mixture of NH_4HCO_3 (100 mM, pH 7.8) and DMF. TCEP (2 mg, 6 μmol, 3 equiv.) and **17** (1 mg, 2 μmol, 0.5 equiv.) were added to the solution and allowed for 5 days. Excess solvent was removed, and the crude product was purified by semi-preparative reverse-phase HPLC (Method **E**). Pure fractions were pooled and lyophilized to yield a white powder (1.0 mg, 1 μmol, 25% yield). ^{1}H NMR (600 MHz, MeOD): δ 7.99 (d, *J* = 1.7 Hz, 1H), 7.98 (s, 1H), 7.78 (s, 1H), 7.73 (s, 1H), 4.53 (s, 2H), 4.47 (q, *J* = 7.1 Hz, 5H), 4.17 (s, 2H), 4.21 – 4.08 (m, 10H), 3.90 (d, *J* = 6.8 Hz, 1H), 3.60 – 3.52 (m, 8H), 3.50 (dd, *J* = 13.9, 6.3 Hz, 1H), 3.24 (dq, *J* = 16.2, 8.8 Hz, 1H), 3.16 (d, *J* = 6.5 Hz, 3H), 3.16 – 3.10 (m, 1H), 3.13 – 3.06 (m, 1H), 2.93 (d, *J* = 7.6 Hz, 8H), 2.42 – 2.35 (m, 1H), 2.38 – 2.30 (m, 3H), 2.25 – 2.17 (m, 1H), 2.17 (td, *J* = 7.0, 3.4 Hz, 3H), 2.12 – 2.04 (m, 1H), 1.90 (ddd, *J* = 14.5, 9.7, 4.8 Hz, 1H), 1.80 (s, 2H), 1.62 (s, 3H), 1.64 – 1.56 (m, 4H), 1.51 (s, 4H), 1.55 – 1.46 (m, 4H), 1.46 – 1.36 (m, 6H), 1.33 (d, *J* = 7.8 Hz, 1H), 1.33 – 1.23 (m, 12H), 1.24 (d, *J* = 2.9 Hz, 3H). ESI-HR-MS. Calcd for $C_{44}H_{71}N_9O_{15}S$ $[M+H]^+$: m/z 998.4790. Found 998.4826.

Synthesis of mpatcn-S-Cys-hex-KuE (19). 9 9 (2.0 mg, 2.2 μmol, 1 equiv.) was dissolved in 1 mL of 1 M KOH and allowed to stir at room temperature overnight. Excess solvent was removed, and the crude product was purified by semi-preparative reverse-phase HPLC (Method **E**). Pure fractions were pooled and lyophilized to yield a white powder (0.22 mg,250 nmol, 11% yield). Due to rapid degradation ^{1}H NMR of the pure final product was unable to be collected. ESI -LR-MS. Calcd for $C_{38}H_{57}N_9O_{15}S$ $[M+H]^+$: m/z 914.4. Found 914.2.

Compound **20** was synthesized according to literature. [5, 6]

Met-KuE (21) The resin bearing **12** (200 mg, 0.16 mmol, 1 equiv.) was swelled in DCM (2 mL) and shook for one minute (5 x), then washed in DMF (5 x). Next, Fmoc-Met-OH (237.73 mg, 0.64 mmol, 4 equiv.), PyBOP (166.5 mg, 0.32 mmol, 2 equiv.), and DIPEA (0.112 mL, 0.64 mmol, 4 equiv.) were added to the resin. The reaction shook overnight at room temperature. Following the reaction, the resin was washed with DMF (5 x). Next, the Fmoc group was removed by shaking the resin with 20% piperidine in DMF (2 mL) for 20 minutes (2 x). After, the resin was washed with DMF (5 x), DCM (5 x), and Et2O (5 x), then dried in a vacuum for 10 mins. The dry resin was then stored for use in future couplings. ^{1}H NMR (600 MHz, MeOD): δ 4.21 (dd, *J* = 8.6, 5.1 Hz, 1H), 4.16 (dd, *J* = 8.4, 4.9 Hz, 1H), 3.81 (dd, *J* = 7.1, 6.1 Hz, 1H), 3.20 – 3.09 (m, 2H), 3.07 (d, *J* = 6.9 Hz, 1H), 2.46 (ddd, *J* = 8.4, 6.8, 1.7 Hz, 2H), 2.38 – 2.24 (m, 2H), 2.12 – 1.92 (m, 7H), 1.85 – 1.70 (m, 2H), 1.60 – 1.20 (m, 11H), 1.19 (s, 2H). ^{13}C NMR (151 MHz, MeOD): δ 175.09, 174.59, 158.72, 117.81, 52.60, 52.34, 52.16, 39.09, 38.69, 35.42, 31.86, 30.68, 29.69, 28.53, 28.49, 28.46, 27.56, 26.01, 25.10, 22.57, 13.73. ESI-HR-MS. Calcd for $C_{23}H_{41}N_5O_9S$ $[M-H]^-$: m/z 562.2552. Found 562.2557.

Synthesis of picaga-Met-hex-KuE (22) 20 (24.6 mg, 39.6 µmol, 1 equiv.) was dissolved in 1 mL DMF. Following this PyBOP (61.9 mg, 119 µmol, 3 equiv.) and DIPEA (34.5 µL, 198 µmol, 5 equiv.) was added and the solution was allowed to stir for 10 minutes. The solution was loaded onto a syringe containing **21** (101 mg, 0.0594 mmol, 1.5 equiv.) on resin. The syringe was shaken overnight, before excess solvent was removed. The syringe was then loaded with TFA:TIS:H_2O (95:2.5:2.5) and shaken for 5 hours before the solution was eluted off the syringe. Excess solvent was removed, and the crude product was purified by semi-preparative reverse-phase HPLC (Method **E**). Pure fractions were pooled and lyophilized to yield a white powder (2 mg ,2 µmol, 5% yield). ^{1}H NMR (600 MHz, D_2O): δ 8.34 (d, *J* = 0.7 Hz, 2H), 7.94 (dd, *J* = 22.2, 7.6 Hz, 1H), 4.70 (s, 3H), 4.16 (d, *J* = 12.8 Hz, 2H), 3.95 (ddt, *J* = 16.4, 7.8, 4.4 Hz, 2H), 3.73 – 3.63 (m, 2H), 3.25 (d, *J* = 0.7 Hz, 2H), 3.06 (td, *J* = 6.9, 4.3 Hz, 5H), 3.02 (s, 3H), 2.90 (s, 2H), 2.73 (d, *J* = 6.3 Hz, 2H), 2.34 (t, *J* = 8.6 Hz, 3H), 2.29 – 2.22 (m, 2H), 1.79 (s, 1H), 1.47 (dd, *J* = 6.2, 3.1 Hz, 2H), 1.45 (s, 4H), 1.43 (s, 4H), 1.42 (s, 3H), 1.39 (s, 3H), 1.26 (p, *J* = 7.6 Hz, 3H), 1.19 – 1.12 (m, 3H). ^{13}C NMR (151 MHz, D_2O): δ 215.37, 170.75, 62.40, 55.05, 39.09, 30.19, 27.96, 25.42, 25.00, 24.30, 22.41, 17.65. ESI-HR-MS. Calcd for $C_{43}H_{67}N_9O_{16}S$ $[M-H]^-$: m/z 996.4426. Found 996.4336.

1.3 Coordination Complex Synthesis

1.3.1 Scandium Complexation

Synthesis of [Sc(8)] To a solution of **8** (350 µg, 0.745 µmol, 1 equiv.) in ammonium acetate (0.25 M, pH 4.5, 1 mL) was added scandium (III) chloride hexahydrate (213 µg, 0.82 µmol, 1.1 equiv.) and the reaction mixture was heated to 80 °C for 1 hour, then checked via LCMS to ensure complete complexation. The scandium complexes were isolated and analyzed as crude product (382 µg, 0.745 µmol, >99% yield). ^{1}H NMR (500 MHz, MeOD) δ: 8.20 (t, *J* = 7.7 Hz, 1H), 8.13 – 8.09 (m, 1H), 7.64 (d, *J* = 7.7 Hz, 1H), 7.37 – 7.14 (m, 7H), 6.98 – 6.91 (m, 2H), 4.54 – 4.37 (m, 2H), 4.24 – 4.03 (m, 4H), 3.54 (dtt, *J* = 27.0, 18.7, 9.3 Hz, 4H), 3.27 – 3.11 (m, 3H), 2.90 (dddd, *J* = 30.0, 12.9, 5.4, 3.0 Hz, 2H), 2.80 – 2.62 (m, 4H), 2.06 (s, 2H). ^{13}C NMR (126 MHz, MeOD) δ: 178.48, 176.96, 175.25, 161.78, 156.25, 141.38, 136.95, 128.30, 128.12, 127.56, 127.24, 127.11, 126.82, 123.91, 122.69, 118.02, 115.69, 64.71, 63.82, 63.72, 55.76, 55.32, 54.91, 53.94, 52.43, 51.72, 48.22, 48.11, 48.05, 47.94, 47.88, 47.77, 47.71, 47.60, 47.56, 47.54, 47.52, 47.51, 47.50, 47.48, 47.43, 47.26, 47.09, 43.79, 23.61, 21.47. ^{45}Sc NMR (122 MHz, MeOD) δ: 68.87. ESI-HR-MS. Calcd for $C_{24}H_{29}N_5O_5Sc$ $[M]^+$: m/z 512.1728. Found 512.1716.

Synthesis of [Sc(14)] To a solution of **14** (47 µg, 0.055 µmol, 1 equiv.) in ammonium acetate (0.25 M, pH 4.5, 1 mL) was added scandium (III) chloride hexahydrate (47 µg, 0.082 µmol, 1.5 equiv.) and the reaction mixture was heated to 80 °C for 1 hour, then checked via LCMS to ensure complete complexation. The scandium complexes were isolated and analyzed as crude product (50 µg, 0.055 µmol, >99% yield). ESI-HR-MS. Calcd for $C_{38}H_{57}N_9O_{14}Sc$ $[M]^+$: m/z 908.3579. Found 908.3579.

Synthesis of [Sc(19)] To a solution of **19** (9.1 µg, 0.01 µmol, 1 equiv.)in ammonium acetate (0.25 M, pH 4.5, 1 mL) was added scandium (III) chloride hexahydrate (1.7 µg, 0.011 µmol, 1.1 equiv.)and the reaction mixture was heated to 80 °C for 1 hour, then checked via LCMS to ensure complete complexation. The scandium complexes were isolated and analyzed as crude product (9.6 µg, 0.01 µmol, >99% yield). ESI-LR-MS. Calcd for $C_{38}H_{54}N_9O_{15}SSc$ $[M+H]^+$: m/z 956.32. Found 956.25.

Synthesis of [Sc(22)] To a solution of **22** (9.9 µg, 0.01 µmol, 1 equiv.) in ammonium acetate (0.25 M, pH 4.5, 1 mL) was added scandium (III) chloride hexahydrate (1.7 µg, 0.011 µmol, 1.1 equiv.)and the reaction mixture was heated to 80 °C for 1 hour, then checked via LCMS to ensure complete complexation. The scandium complexes were isolated and analyzed as crude product (10.4 µg, 0.01 µmol, >99% yield). ESI-LR-MS. Calcd for $C_{43}H_{64}N_9O_{16}SSc$ $[M+H]^+$: m/z 1040.375. Found 1040.397.

1.3.2 Lutetium Complexation

Synthesis of [Lu(8)] To a solution of **8** (350 µg, 0.745 µmol, 1 equiv.) in ammonium acetate (0.25 M, pH 4.5, 1 mL) was added lutetium (III) chloride hexahydrate (213 µg, 0.82 µmol, 1.1 equiv.) and the reaction mixture was heated to 80 °C for 1 hour, then checked via LCMS to ensure complete complexation. The scandium complexes were isolated and analyzed as crude product (382 µg, 0.745 µmol, >99% yield). ^{1}H NMR (500 MHz, MeOD) δ: 8.24 (dd, *J* = 9.3, 5.8 Hz, 1H), 8.16 (d, *J* = 6.7 Hz, 1H), 7.72 (d, *J* = 8.3 Hz, 1H), 7.34 (dt, *J* = 21.2, 4.6 Hz, 3H), 7.19 (dd, *J* = 7.3, 3.3 Hz, 4H), 6.96 (d, *J* = 6.6 Hz, 2H), 4.56 – 4.41 (m, 2H), 4.32 – 4.20 (m, 2H), 4.17 – 4.12 (m, 2H), 4.01 (d, *J* = 16.6 Hz, 1H), 3.66 – 3.40 (m, 5H), 3.21 – 2.68 (m, 6H). ^{13}C NMR (126 MHz, MeOD) δ: 180.70, 175.62, 157.10, 141.81, 136.95, 128.34, 128.16, 127.54, 127.27, 127.18, 124.58, 123.24, 118.00, 64.13, 63.99, 63.20, 55.17, 54.71, 54.40, 53.22, 52.15, 48.12, 48.11, 48.06, 47.95, 47.94, 47.89, 47.78, 47.77, 47.72, 47.61, 47.60, 47.44, 47.43, 47.27, 47.26, 47.10, 47.09, 43.71, 22.23. ESI-HR-MS. Calcd for $C_{24}H_{29}LuN_5O_5$ $[M]^+$: m/z 642.1576. Found 642.1563.

Synthesis of [Lu(14)] To a solution of **14** (47 µg, 0.055 µmol, 1 equiv.) in ammonium acetate (0.25 M, pH 4.5, 1 mL) was added lutetium (III) chloride hexahydrate (23 µg, 0.082 µmol, 1.5 equiv.) and the reaction mixture was heated to 80 °C for 1 hour, then checked via LCMS to ensure complete complexation. The lutetium complexes were isolated and analyzed as crude product (57 µg, 0.055 µmol, >99% yield). ESI-HR-MS Calcd for $C_{38}H_{57}LuN_9O_{14}$ $[M-2H]^-$: m/z 1036.3277. Found 1036.3311.

Synthesis of [Lu(19)] To a solution of **19** (9.1 µg, 0.01 µmol, 1 equiv.) in ammonium acetate (0.25 M, pH 4.5, 1 mL) was added lutetium (III) chloride hexahydrate (1.7 µg, 0.011 µmol, 3.1 equiv.)and the reaction mixture was heated to 80 °C for 1 hour, then checked via LCMS to ensure complete complexation. The lutetium complexes were isolated and analyzed as crude product (11 µg, 0.01 µmol, >99% yield). ESI-LR-MS. Calcd for $C_{38}H_{54}LuN_9O_{15}S$ $[M+H]^+$: m/z 1086.3. Found 1085.9.

Synthesis of [Lu(22)] To a solution of **22** (9.9 µg, 0.01 µmol, 1 equiv.) in ammonium acetate (0.25 M, pH 4.5, 1 mL) was added lutetium (III) chloride hexahydrate (1.7 µg, 0.011 µmol, 3.1 equiv.)and the reaction mixture was heated to 80 °C for 1 hour, then checked via LCMS to ensure complete complexation. The lutetium complexes were isolated and analyzed as crude product (11.7 µg, 0.01 µmol, >99% yield). ESI-LR-MS. Calcd for $C_{43}H_{64}N_9O_{16}SLu$ $[M+H]^+$: m/z 1170.368. Found 1170.348.

1.3.3 Scandium-Fluoride Ternary Complex Formation

Synthesis of [ScF(8)]To a solution of **[Sc(8)]** (350 µg, 0.745 µmol, 1 equiv.) in ammonium acetate (0.25 M, pH 4.5, 1 mL) was added fluoride (70.7 µg, 3.725 µmol, 5 equiv.) and the reaction mixture was heated to 100 °C for 1 hour, then checked via LCMS to ensure complete fluorination. The scandium-fluoride ternary complexes were lyophilised and redissolved in acetonitrile to remove excess ammonium acetate and were used without further purification (382 µg, 0.745 µmol, >99% yield.). ^{1}H NMR (500 MHz, MeOD) δ: 8.22 – 8.15 (m, 1H), 8.15 – 8.06 (m, 1H), 7.62 (d, *J* = 7.6 Hz, 1H), 7.38 – 7.13 (m, 7H), 6.89 (dt, *J* = 6.6, 1.6 Hz, 2H), 4.45 (dd, *J* = 8.6, 7.4 Hz, 2H), 4.19 – 4.11 (m, 3H), 4.08 – 4.01 (m, 4H), 3.60 – 3.40 (m, 3H), 3.32 (d, *J* = 15.3 Hz, 2H), 3.27 – 3.13 (m, 1H), 2.77 – 2.69 (m, 1H), 2.69 – 2.55 (m, 4H). ^{13}C NMR (126 MHz, MeOD) δ: 178.28, 177.28, 175.00, 161.79, 161.52, 156.23, 141.17, 136.97, 128.31, 128.12, 127.53, 127.25, 127.11, 126.82, 124.01, 122.72, 118.02, 115.69, 63.62, 63.03, 61.25, 54.90, 54.82, 53.00, 52.47, 51.41, 48.23, 48.11, 48.06, 47.94, 47.88, 47.77, 47.71, 47.60, 47.54, 47.43, 47.26, 47.09, 43.79, 21.69. ^{45}Sc NMR (122 MHz, MeOD) δ: 69.55. ^{19}F NMR (471 MHz, MeOD) δ: -4.26. ESI-HR-MS. Calcd for $C_{24}H_{29}FN_5O_5Sc$ $[M-H]^-$: m/z 530.1634. Found 530.1640.

Synthesis of [ScF(14)] To a solution of **[Sc(14)]** (50 µg, 0.055 µmol, 1 equiv.) in ammonium acetate (0.25 M, pH 4.5, 1 mL) was added fluoride (10.2 µg, 0.275 µmol, 5 equiv.) and the reaction mixture was heated to 100 °C for 1 hour, then checked via LCMS to ensure complete fluorination. The scandium-fluoride ternary complexes were lyophilised and redissolved in acetonitrile to remove excess ammonium acetate and were used without further purification (52 µg, 0.055 µmol, >99% yield.). ESI-LR-MS. Calcd for $C_{38}H_{57}FN_9O_{14}Sc$ $[M+H]^+$: m/z 928.36. Found 928.0.

Synthesis of [ScF(22)] To a solution of **[Sc(22)]** (10.4 µg, 0.01 µmol,1 equiv.) in ammonium acetate (0.25 M, pH 4.5, 1 mL) was added fluoride (0.95 µg, 0.05 µmol,5 equiv.)) and the reaction mixture was heated to 100 °C for 1 hour, then checked via LCMS to ensure complete fluorination. The scandium-fluoride ternary complexes were lyophilised and redissolved in acetonitrile to remove excess ammonium acetate and were used without further purification (yield x). ESI-HR-MS. Calcd for $C_{43}H_{64}FN_9O_{16}SSc$ $[M]^-$: m/z 1058.3735. Found 1058.3946.

2 Ligand and Complex Characterization

2.1 NMR

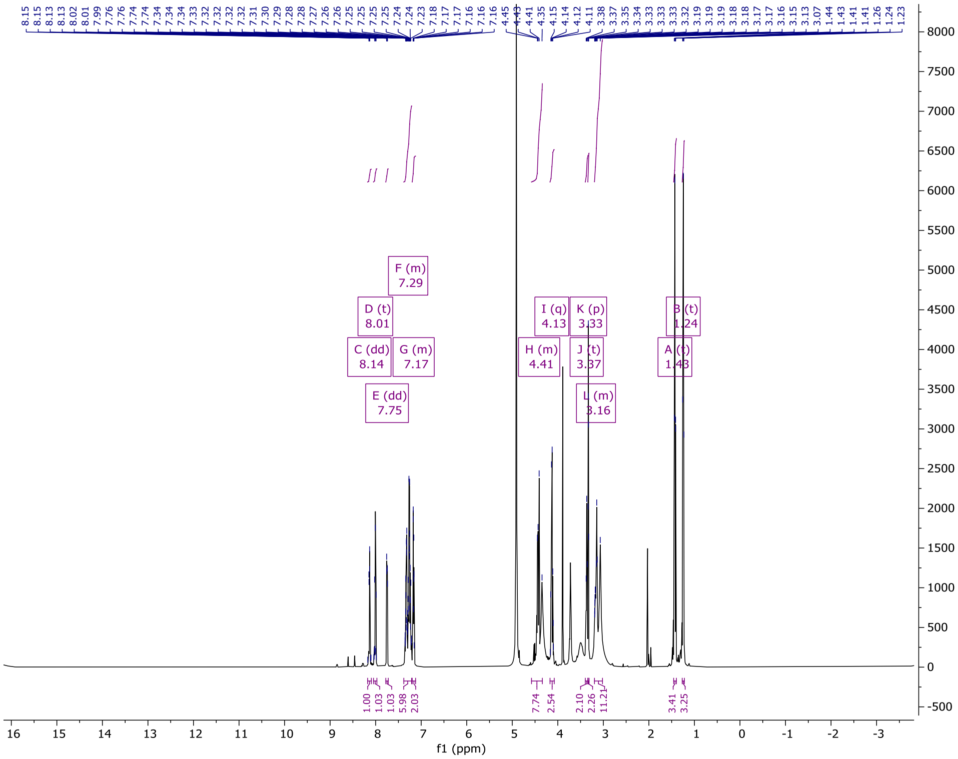

Figure S5. The ^{1}H NMR spectra of **7** in MeOD.

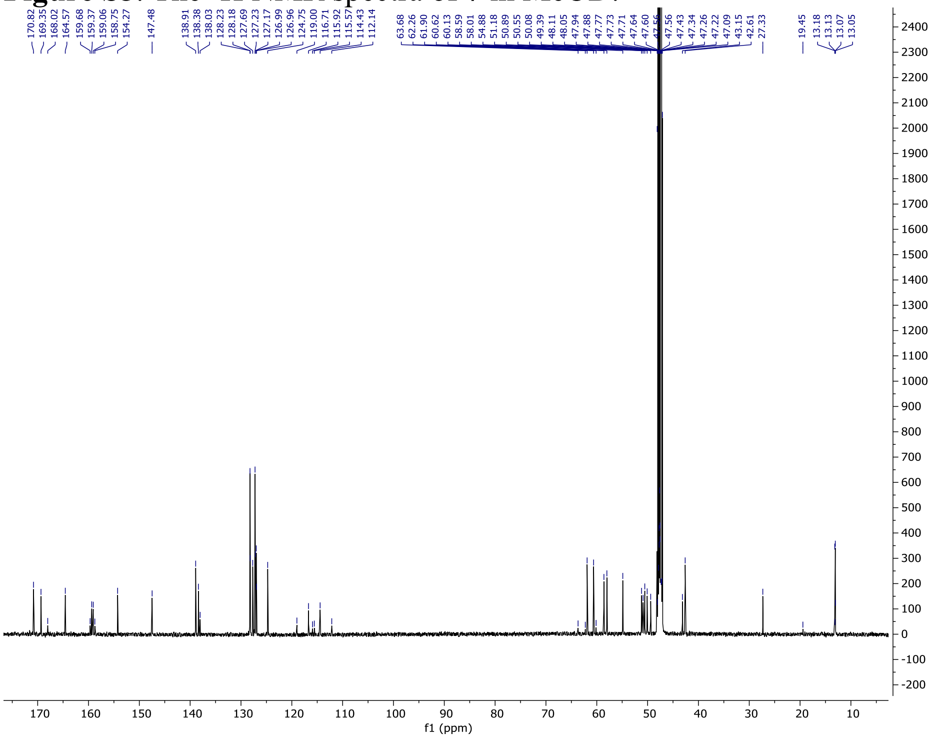

Figure S6 The $^{13}C\{^{1}H\}$ NMR spectrum of **7** in MeOD.

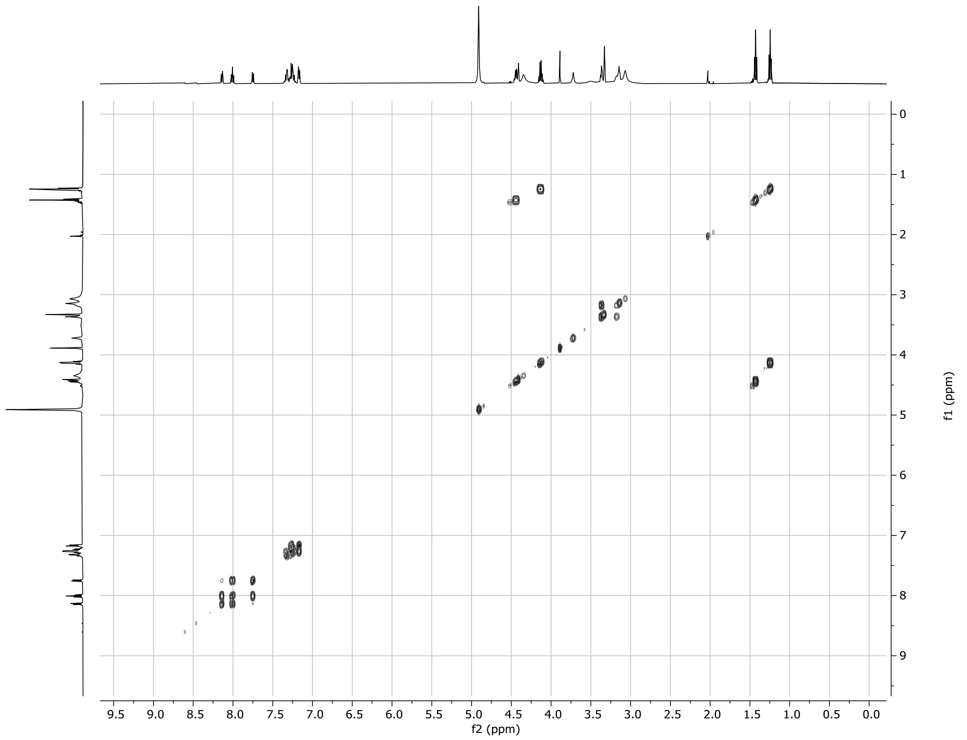

Figure S7 The ^{1}H-^{1}H COSY NMR spectrum of **7** in MeOD.

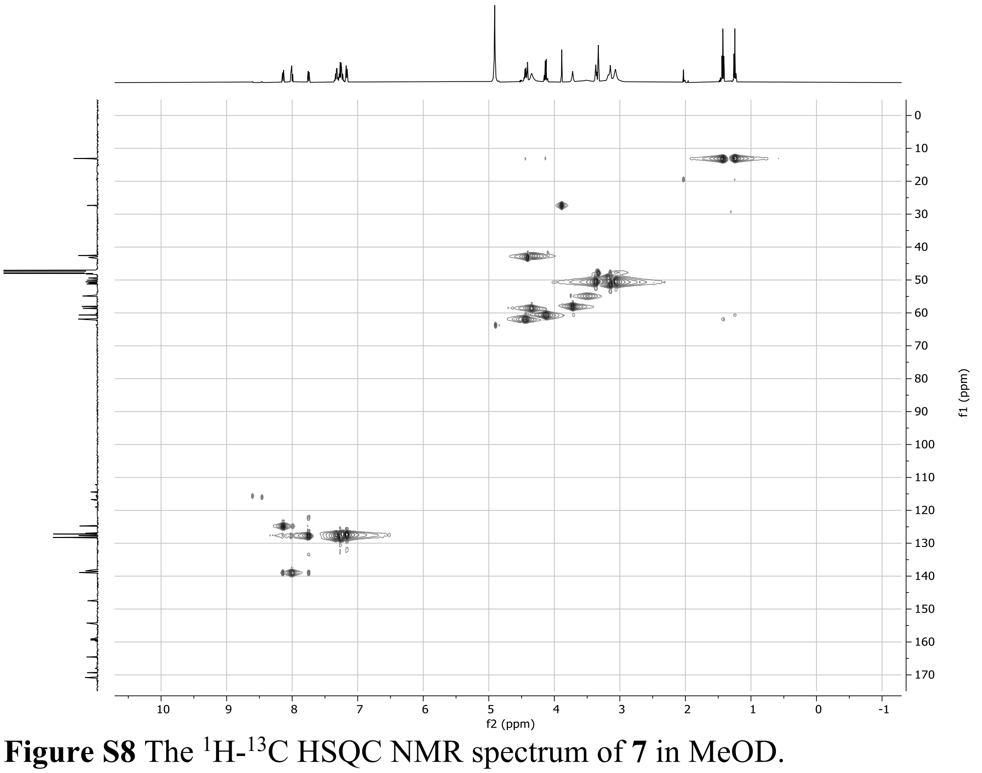

Figure S8 The ^{1}H-^{13}C HSQC NMR spectrum of **7** in MeOD.

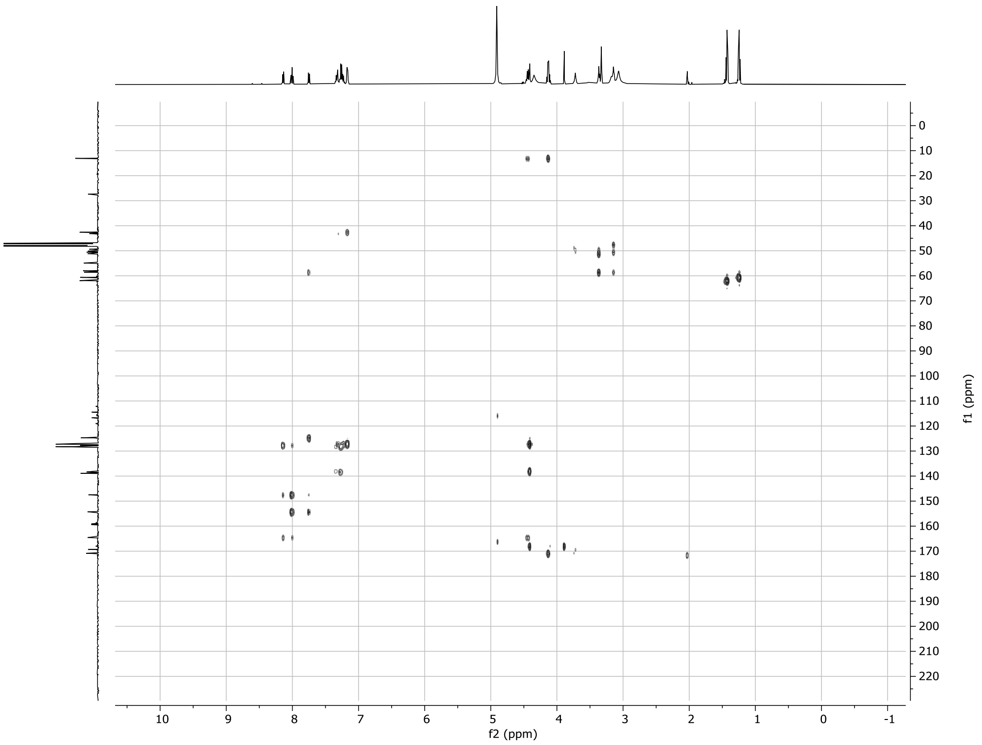

Figure S9 The ^{1}H-^{13}C HMBC NMR spectrum of **7** in MeOD.

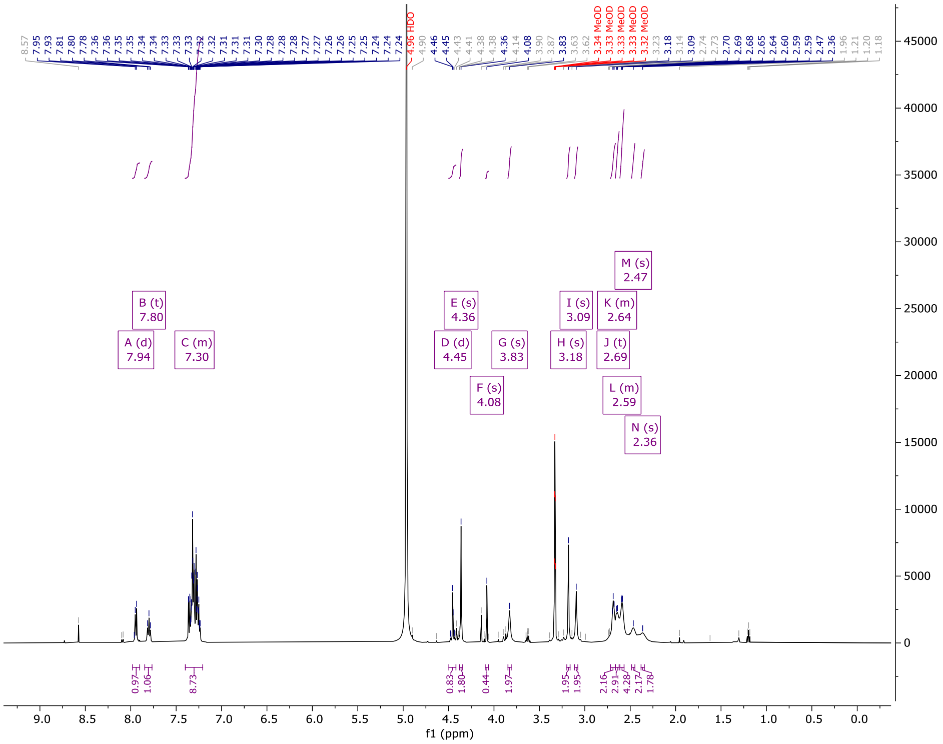

Figure S10. The ^{1}H NMR spectra of **8** in MeOD.

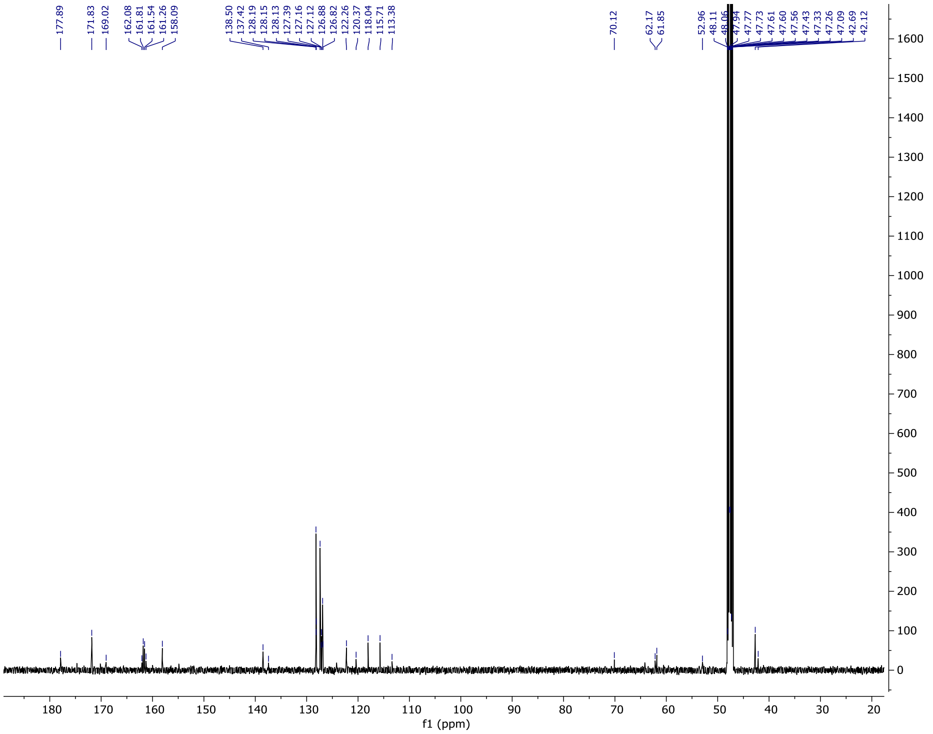

Figure S11 The $^{13}C\{^1H\}$ NMR spectrum of **8** in MeOD.

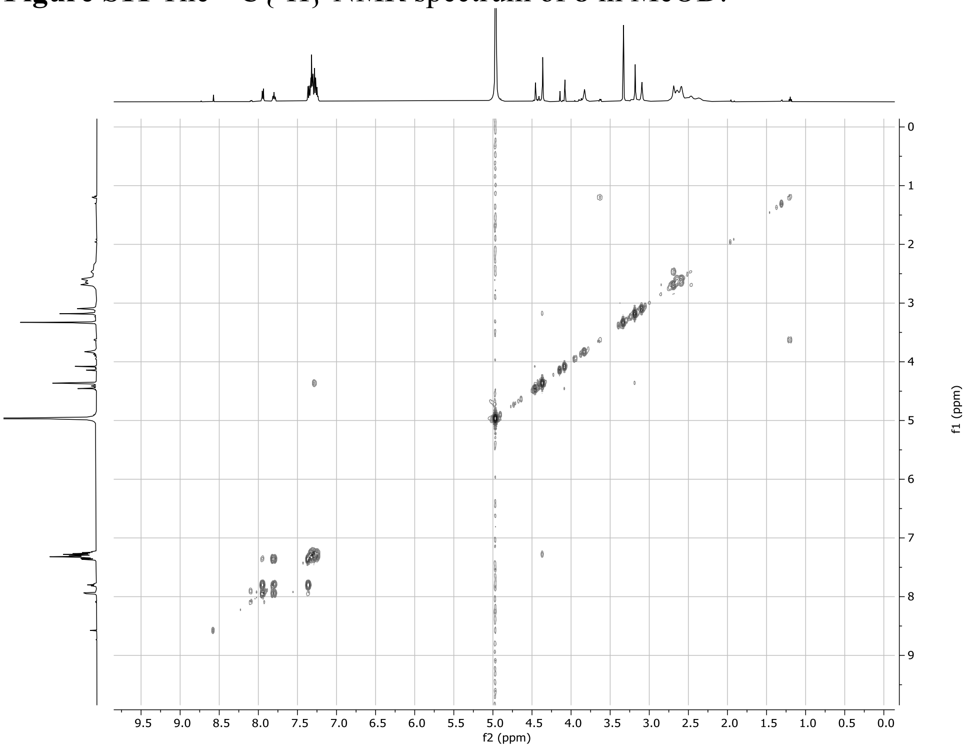

Figure S12 The ^1H-^1H COSY NMR spectrum of **8** in MeOD.

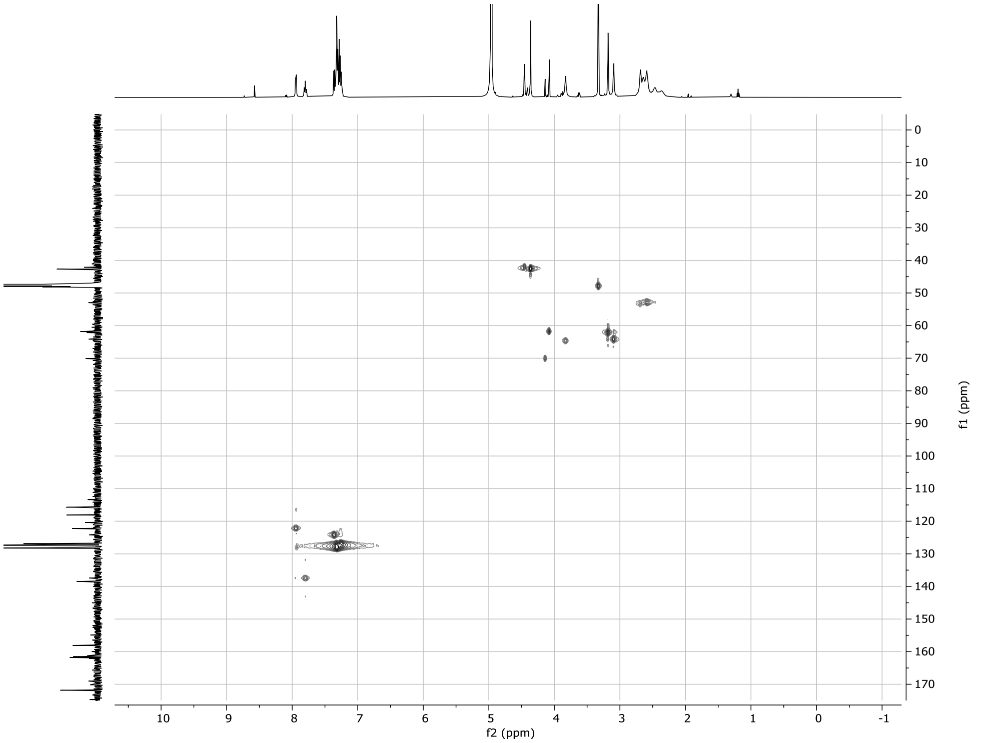

Figure S13 The ^{1}H-^{13}C HSQC NMR spectrum of **8** in MeOD.

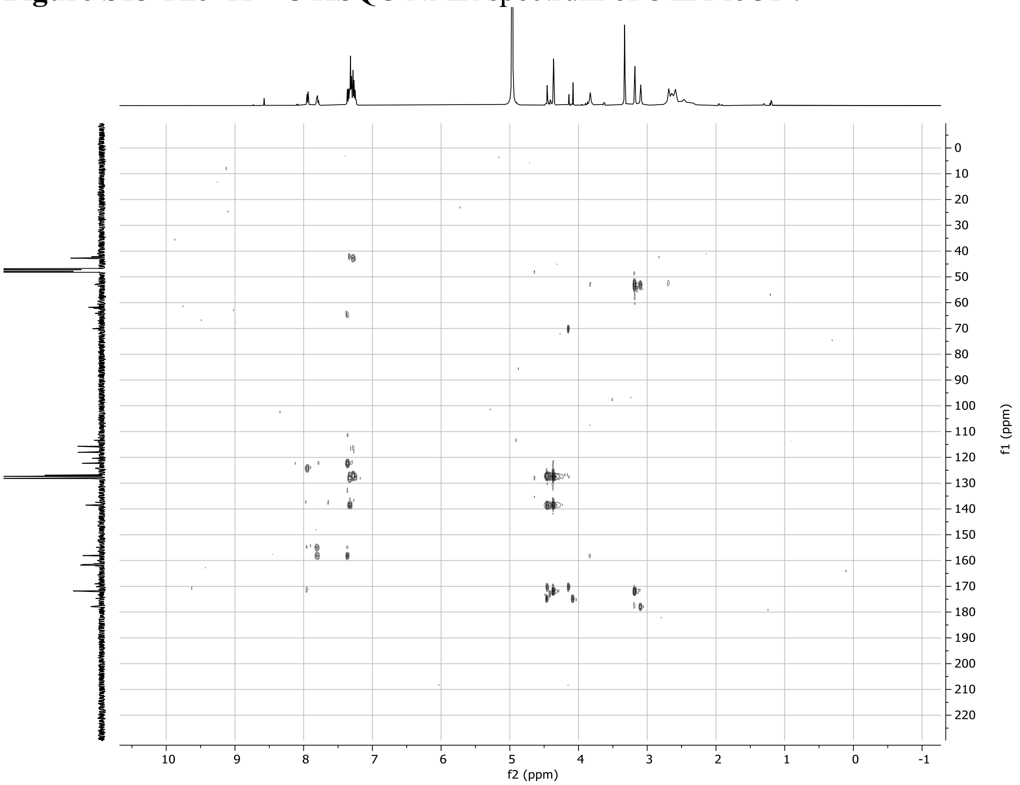

Figure S14 The ^{1}H-^{13}C HMBC NMR spectrum of **8** in MeOD.

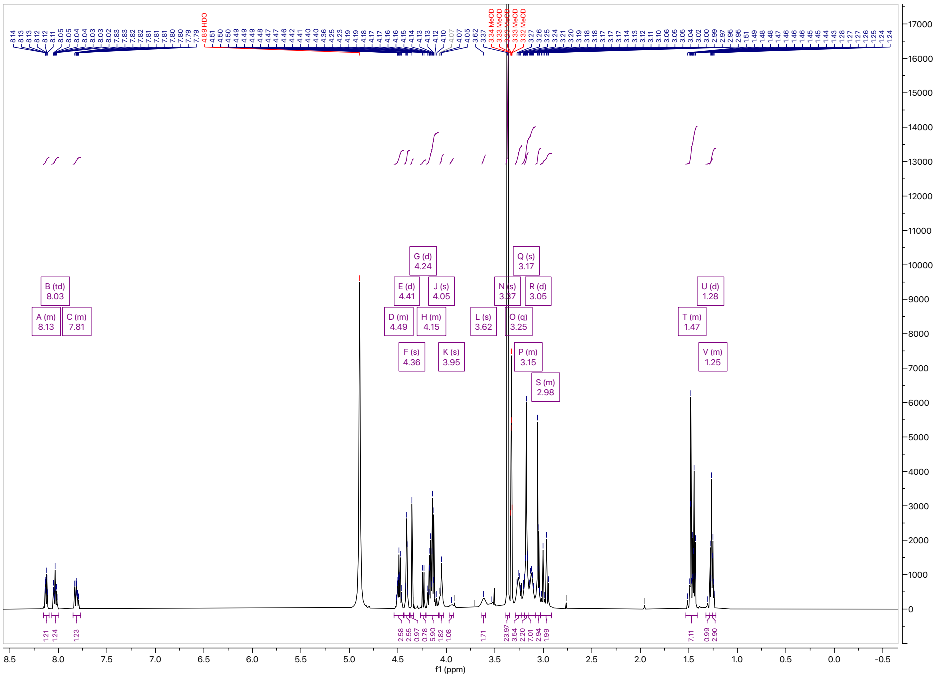

Figure S15. The ^{1}H NMR spectra of **10** in MeOD.

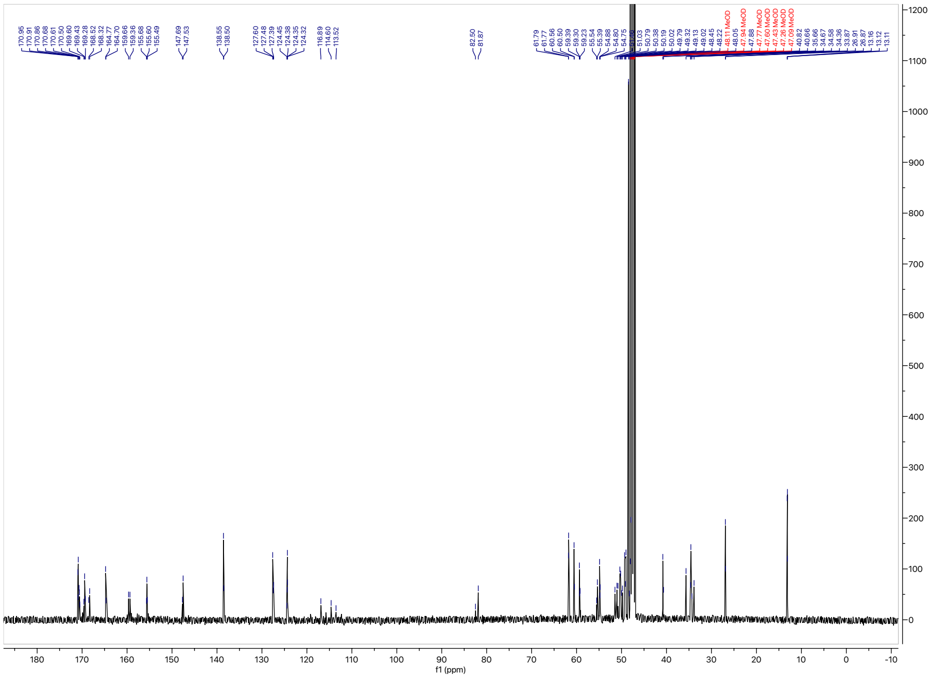

Figure S16 The ^{13}C{^{1}H} NMR spectrum of **10** in MeOD.

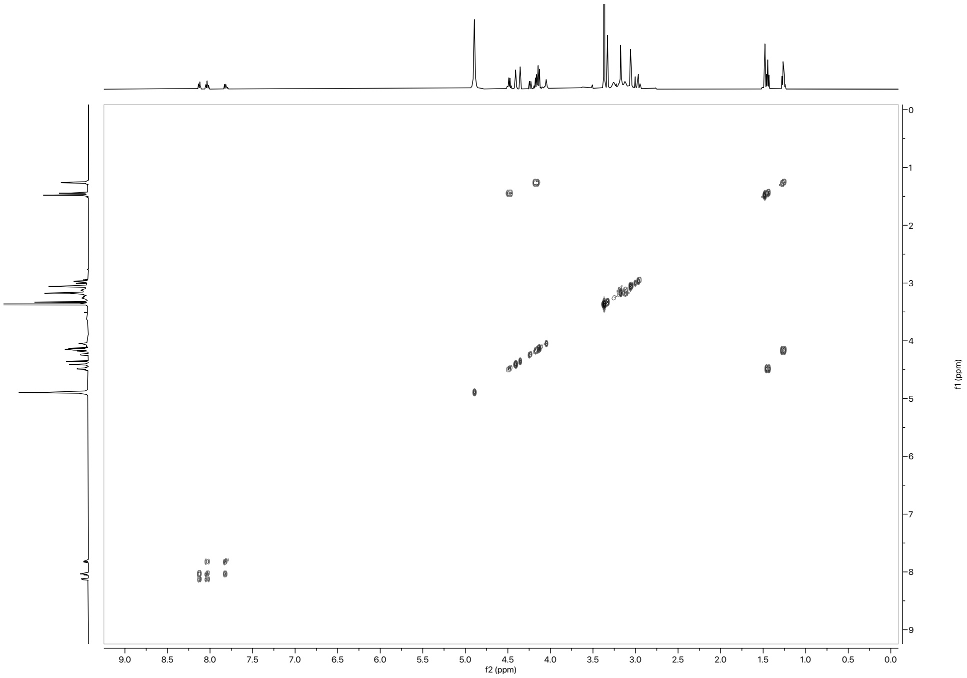

Figure S17 The ^{1}H-^{1}H COSY NMR spectrum of **10** in MeOD.

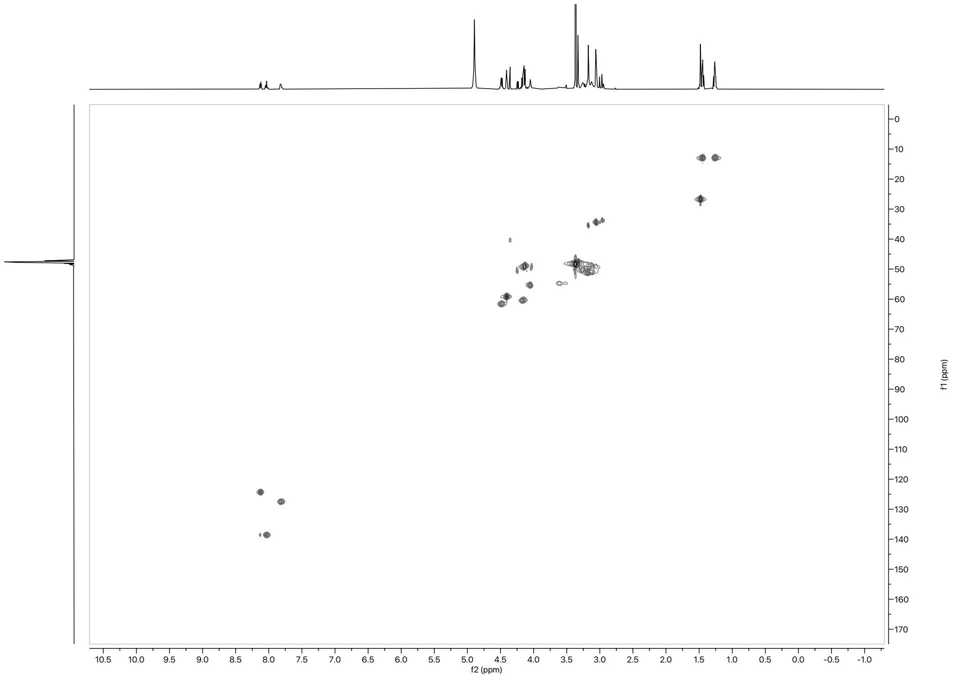

Figure S18 The ^{1}H-^{13}C HSQC NMR spectrum of **10** in MeOD.

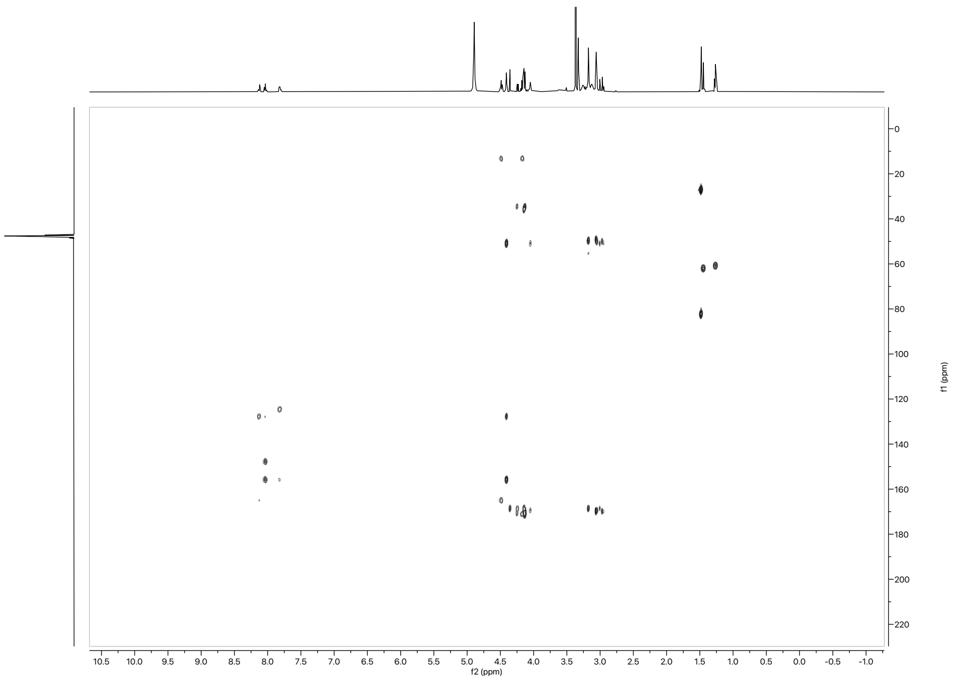

Figure S19 The ^{1}H-^{13}C HMBC NMR spectrum of **10** in MeOD.

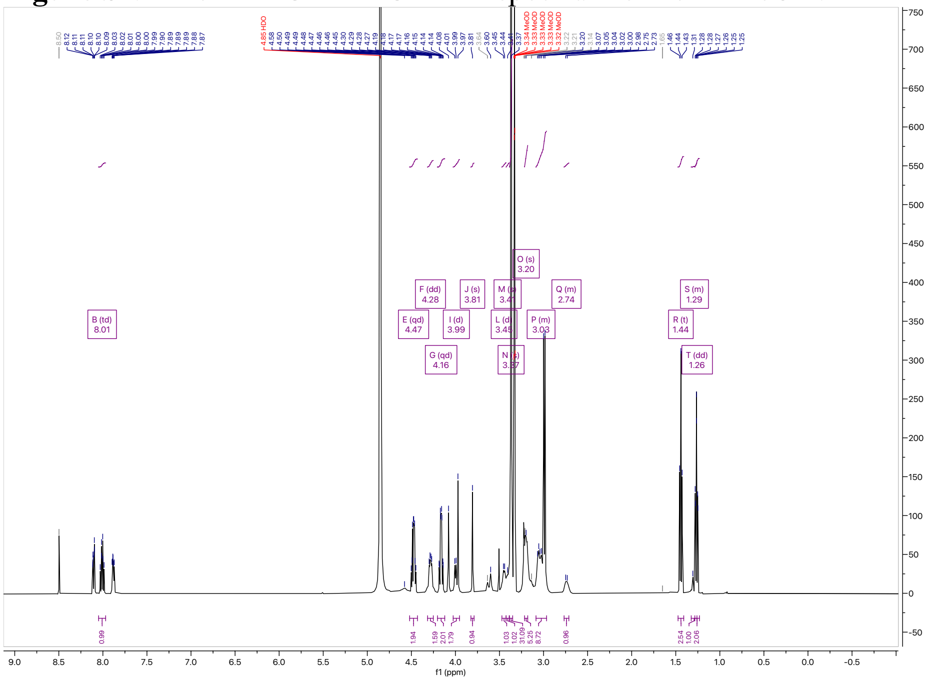

Figure S20. The ^{1}H NMR spectra of **11** in MeOD.

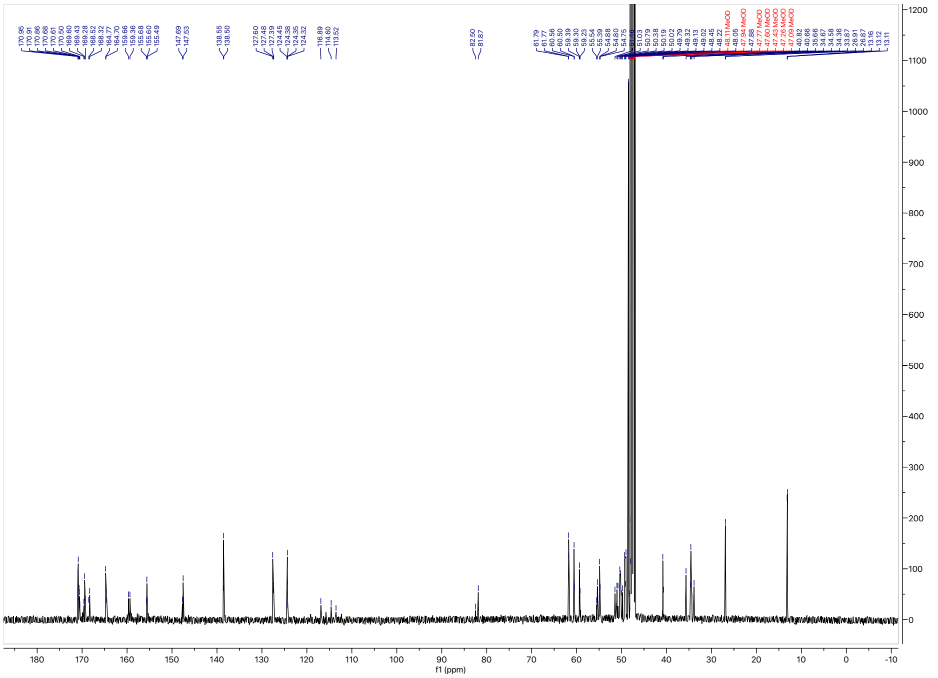

Figure S21 The $^{13}C\{^{1}H\}$ NMR spectrum of **11** in MeOD.

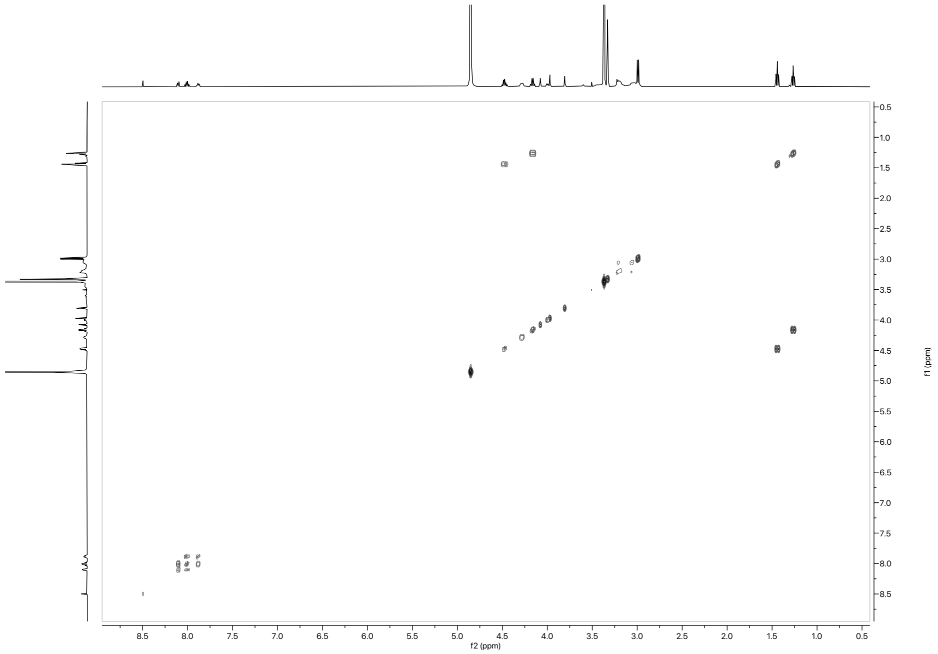

Figure S22 The ^{1}H-^{1}H COSY NMR spectrum of **11** in MeOD.

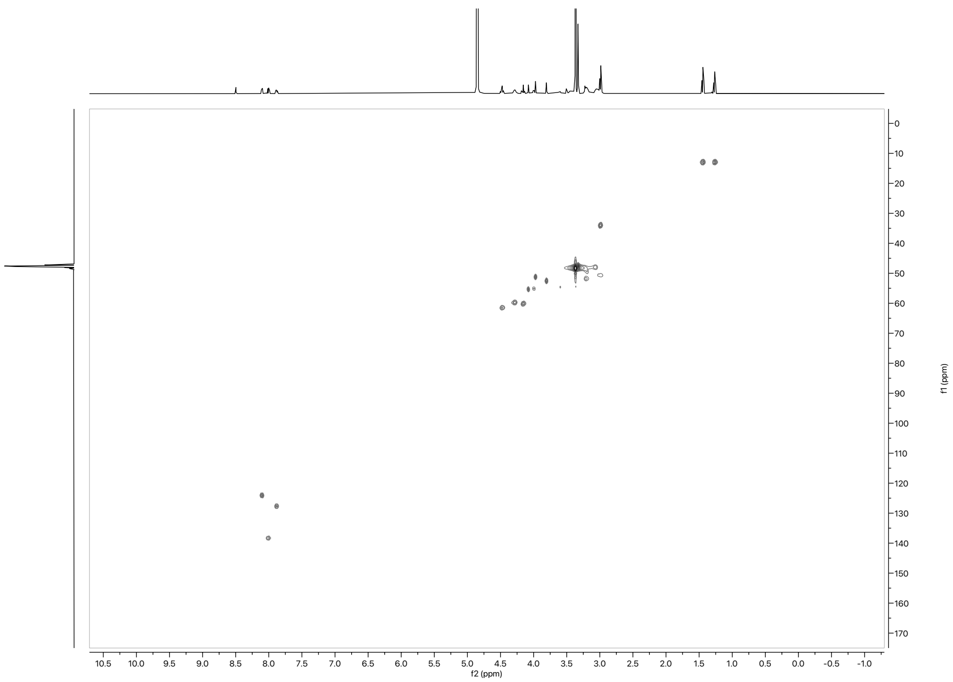

Figure S23 The ^{1}H-^{13}C HSQC NMR spectrum of **11** in MeOD.

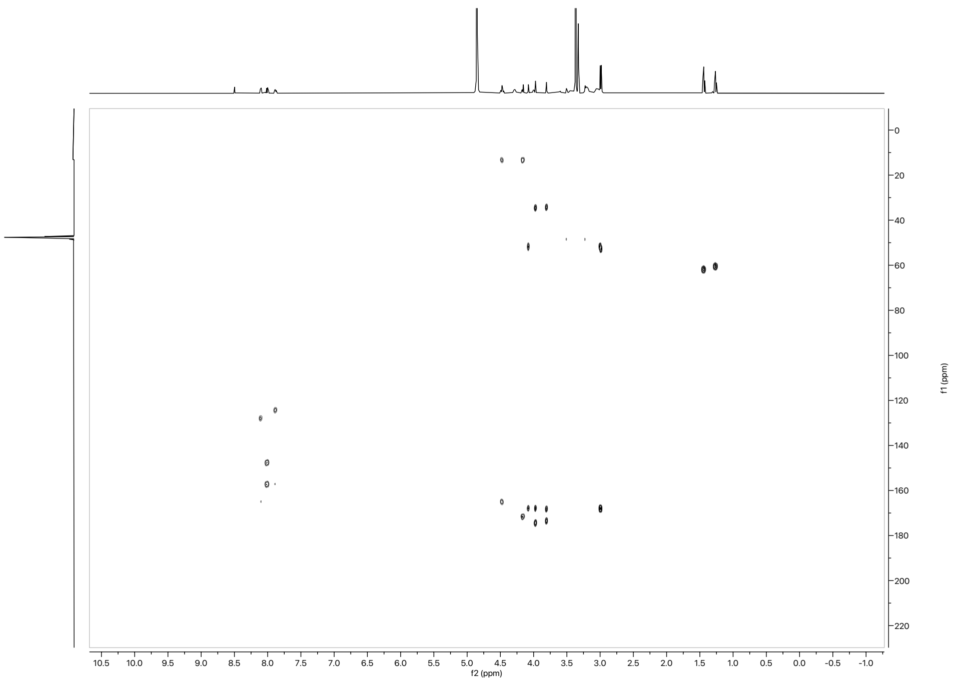

Figure S24 The ^{1}H-^{13}C HMBC NMR spectrum of **11** in MeOD.

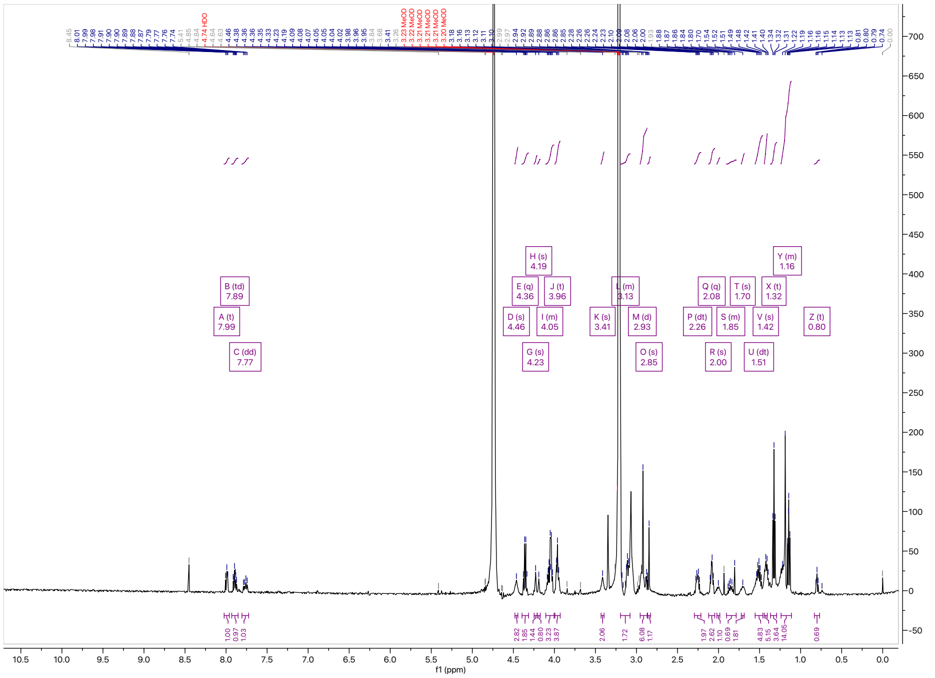

Figure S25. The ^{1}H NMR spectra of **13** in MeOD.

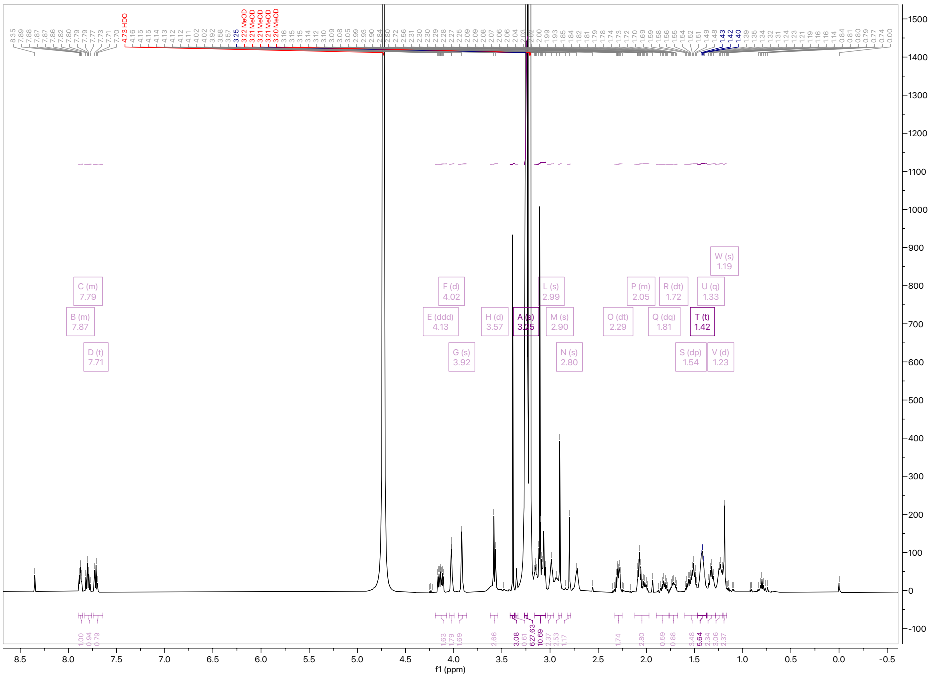

Figure S26. The ^{1}H NMR spectra of **14** in MeOD.

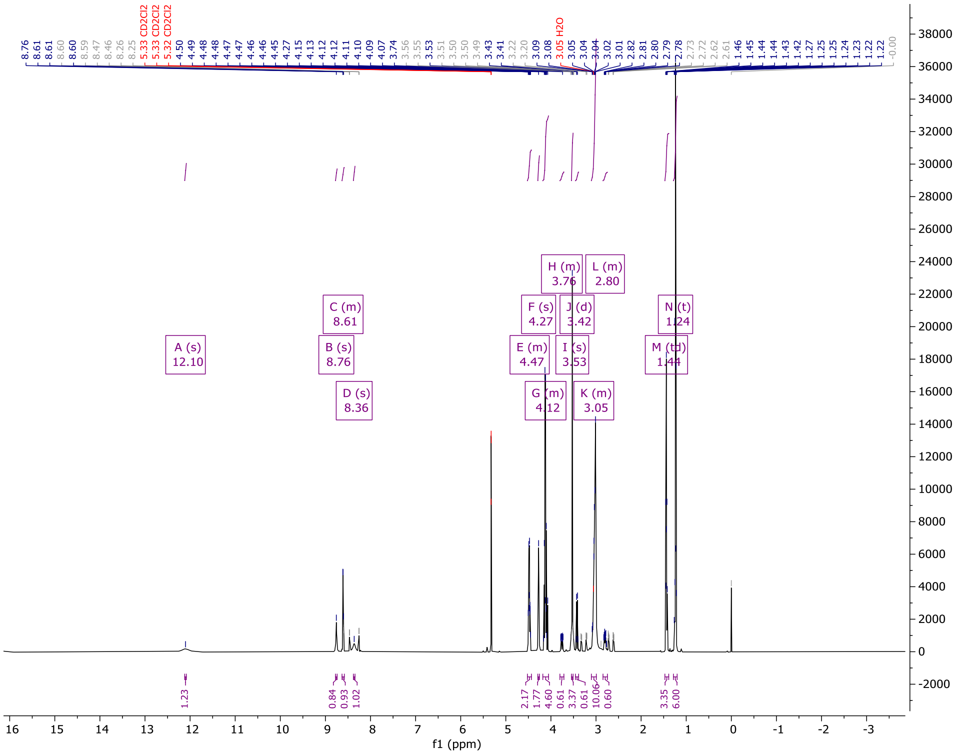

Figure S27. The ^{1}H NMR spectra of **16** in DCM-d$_2$.

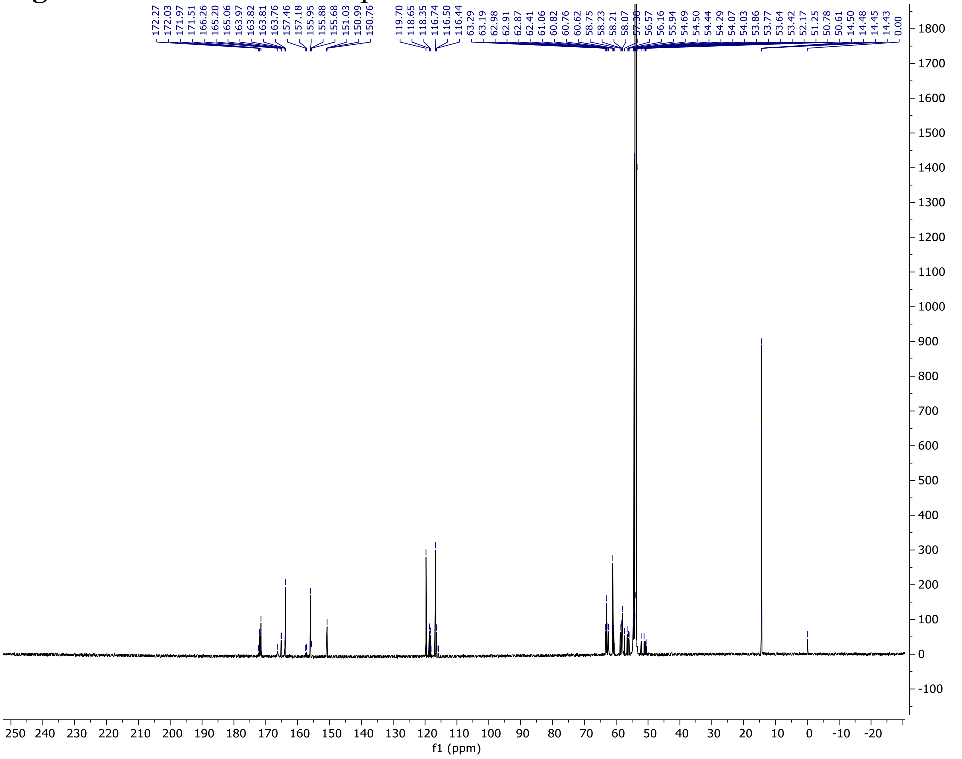

Figure S28. The $^{13}C\{^{1}H\}$ NMR spectra of **16** in DCM-d$_2$.

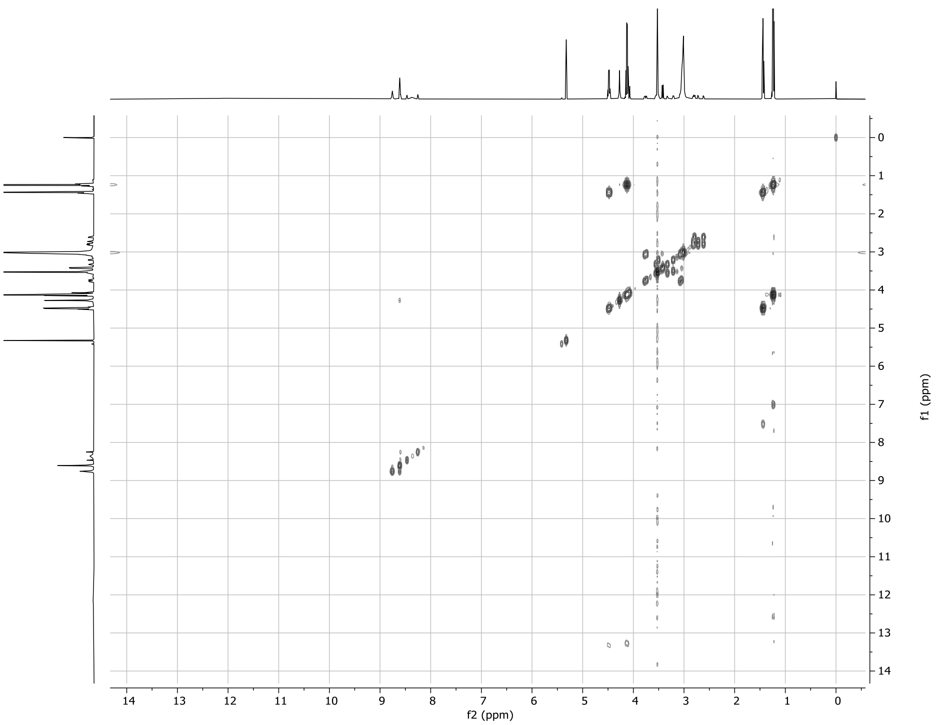

Figure S29. The ^{1}H-^{1}H COSY NMR spectrum of **16** in DCM-d_2.

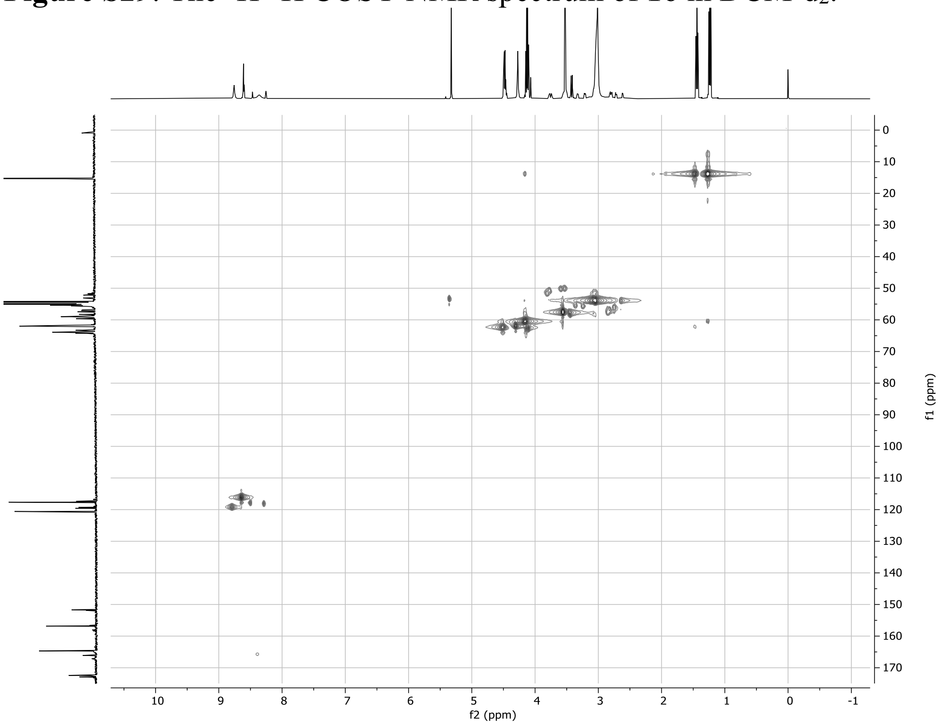

Figure S30 The ^{1}H-^{13}C HSQC NMR spectrum of **16** in DCM-d_2.

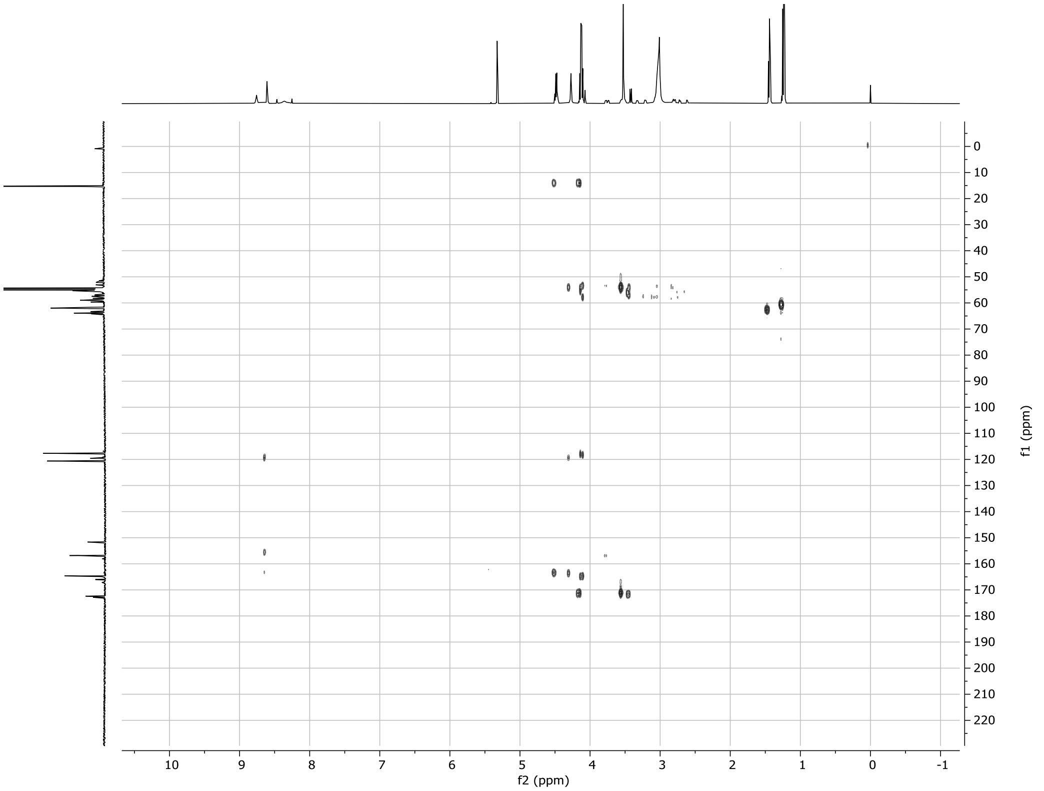

Figure S31 The ^{1}H-^{13}C HMBC NMR spectrum of **16** in DCM-d_2.

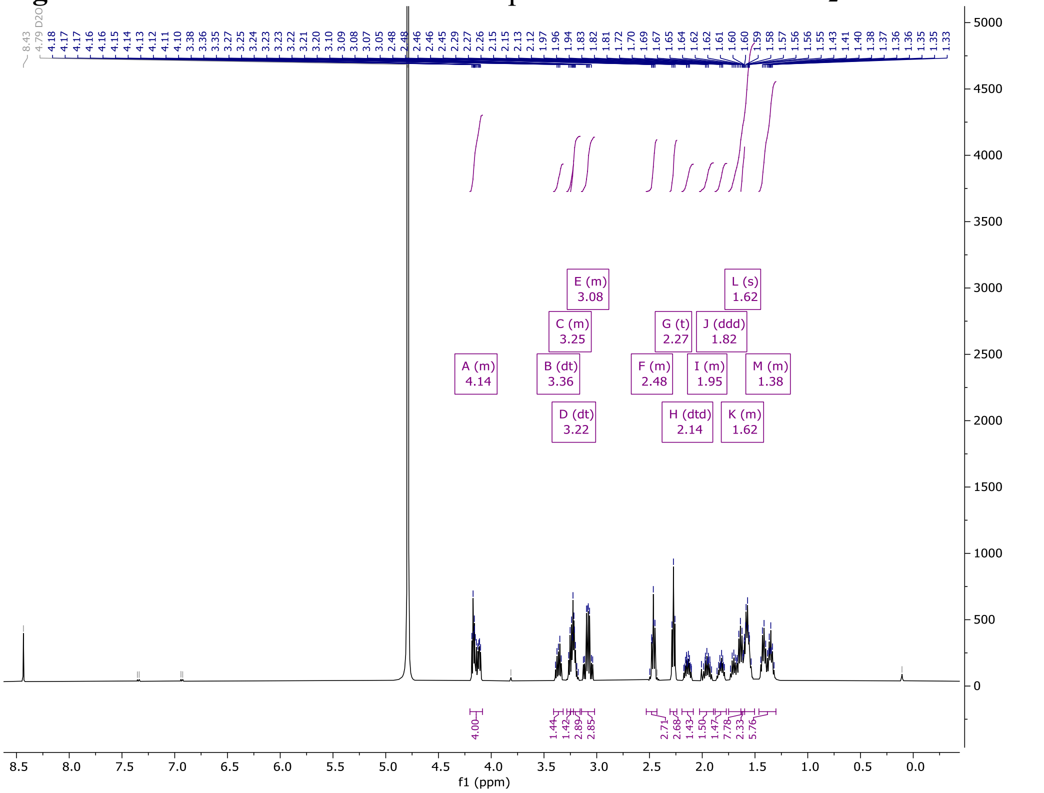

Figure S32. The ^{1}H NMR spectra of **17** in D_2O.

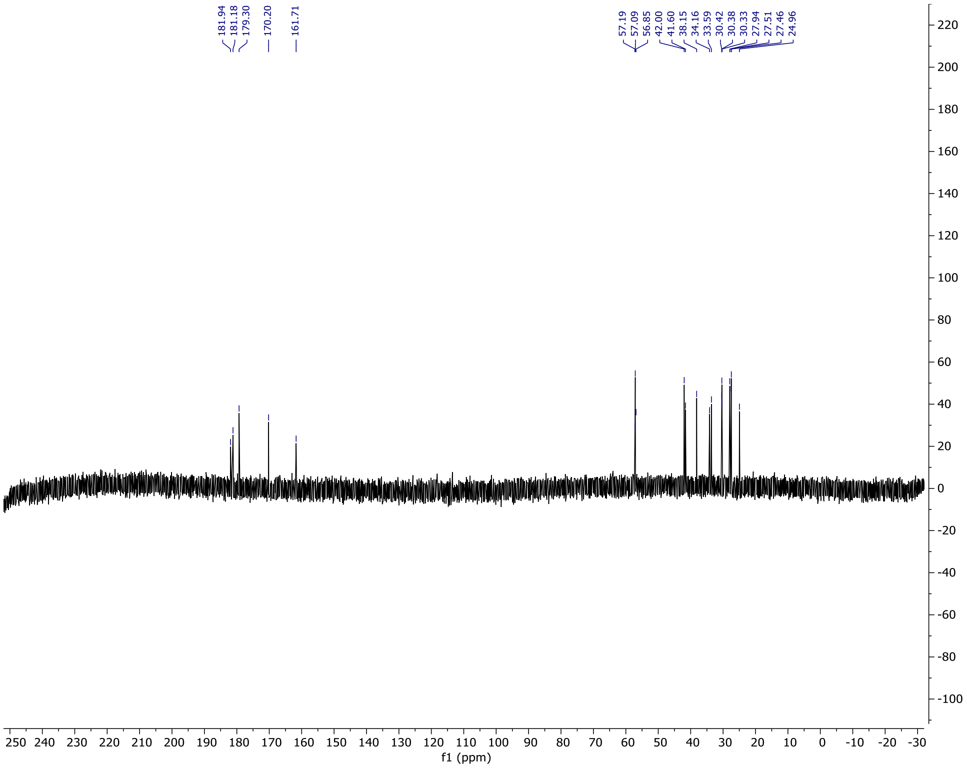

Figure S33. The 13C{^{1}H} NMR spectra of **17** in D_2O.

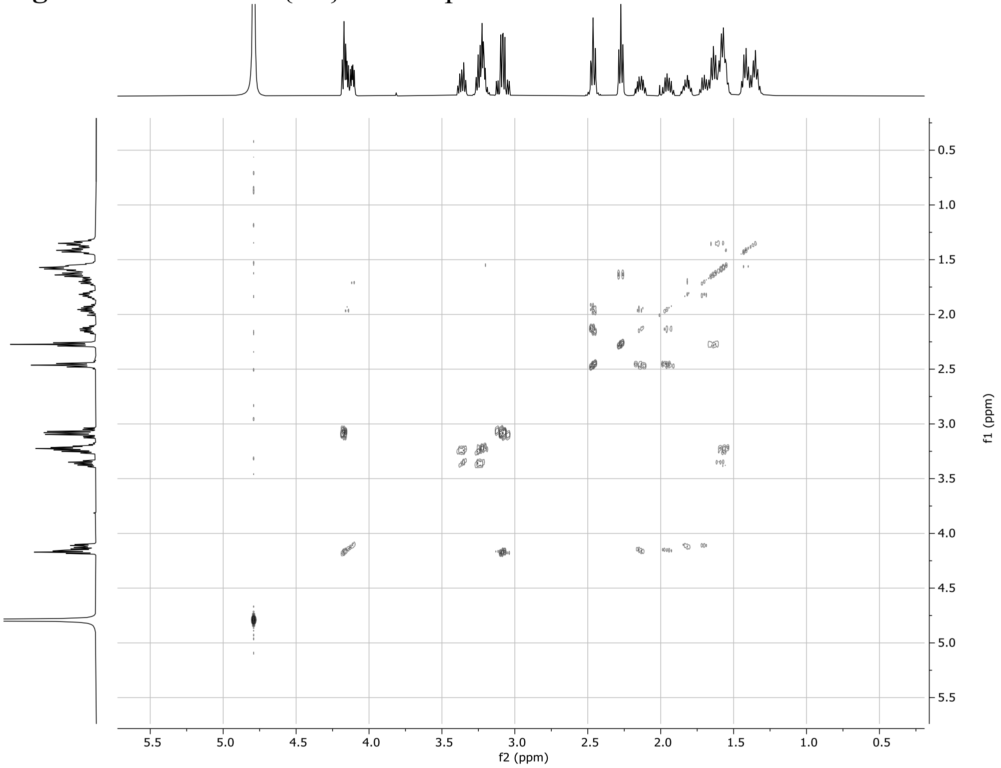

Figure S34. The ^{1}H-^{1}H COSY NMR spectrum of **17** in DCM-d_2

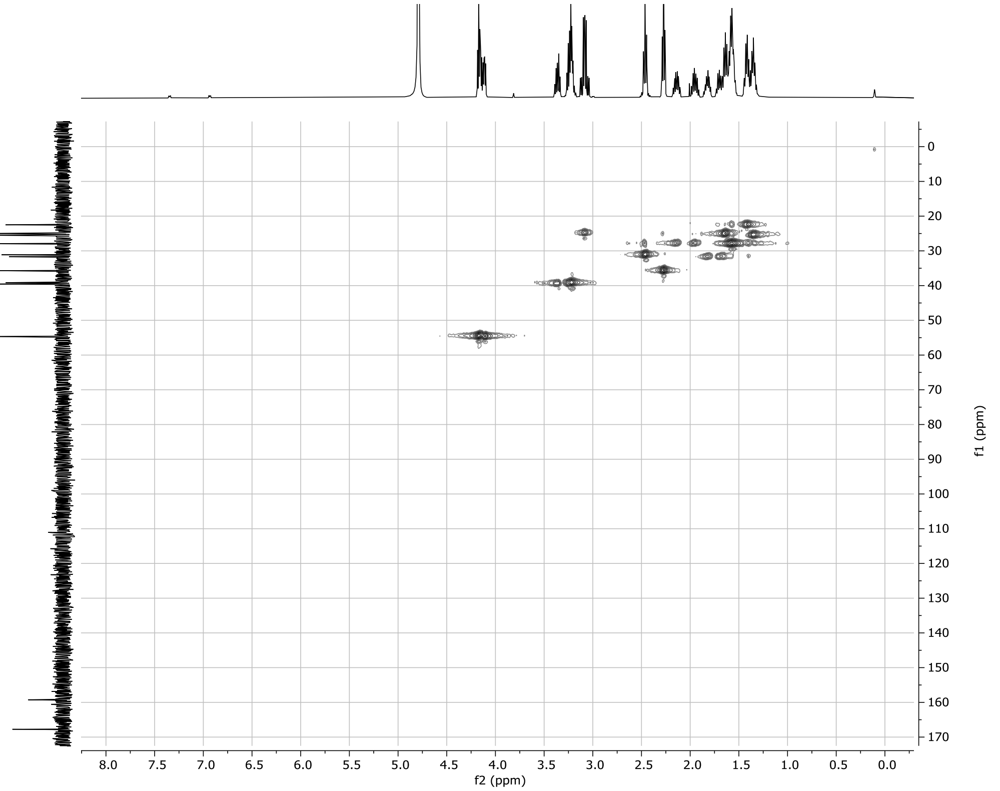

Figure S35 The ^{1}H-^{13}C HSQC NMR spectrum of **16** in DCM-d_2.

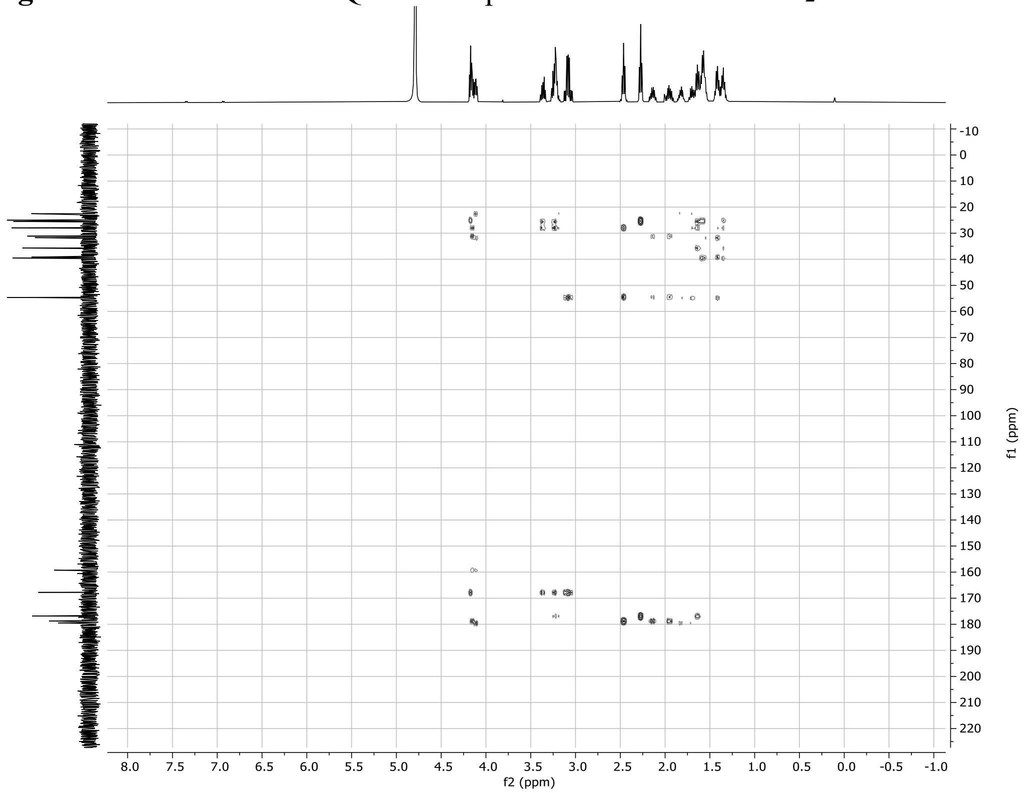

Figure S36 The ^{1}H-^{13}C HMBC NMR spectrum of **17** in DCM-d_2.

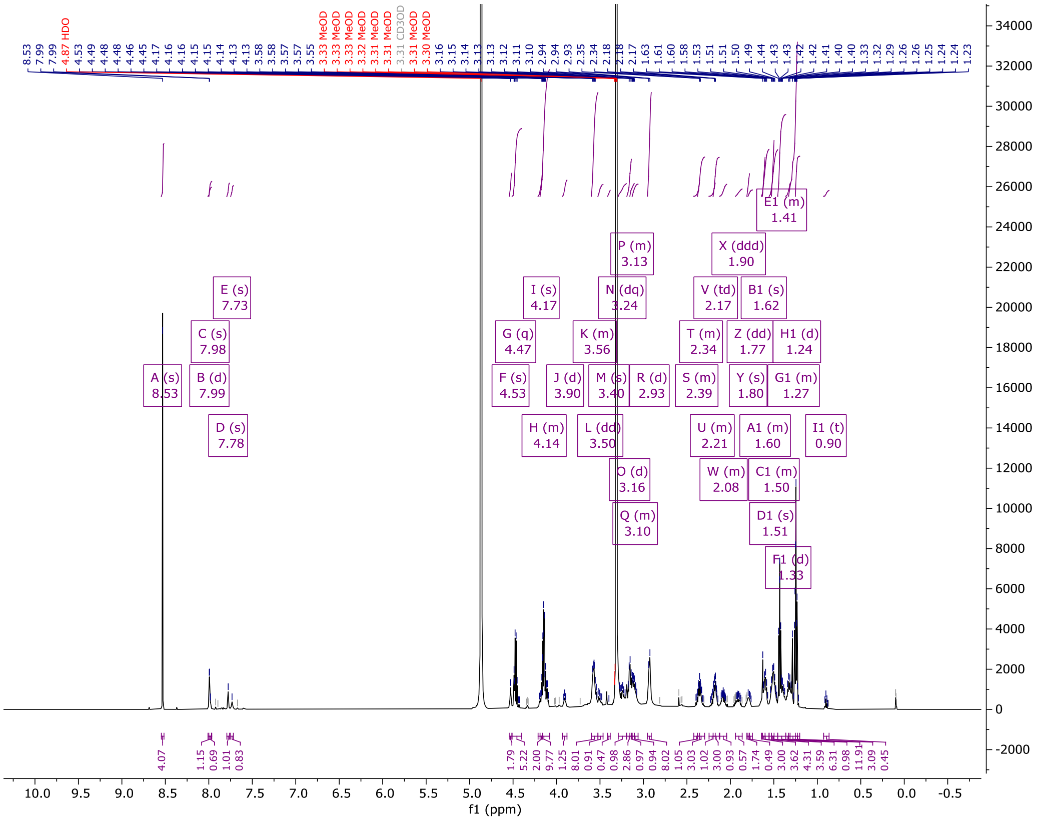

Figure S37. The ^{1}H NMR spectra of **18** in MeOD.

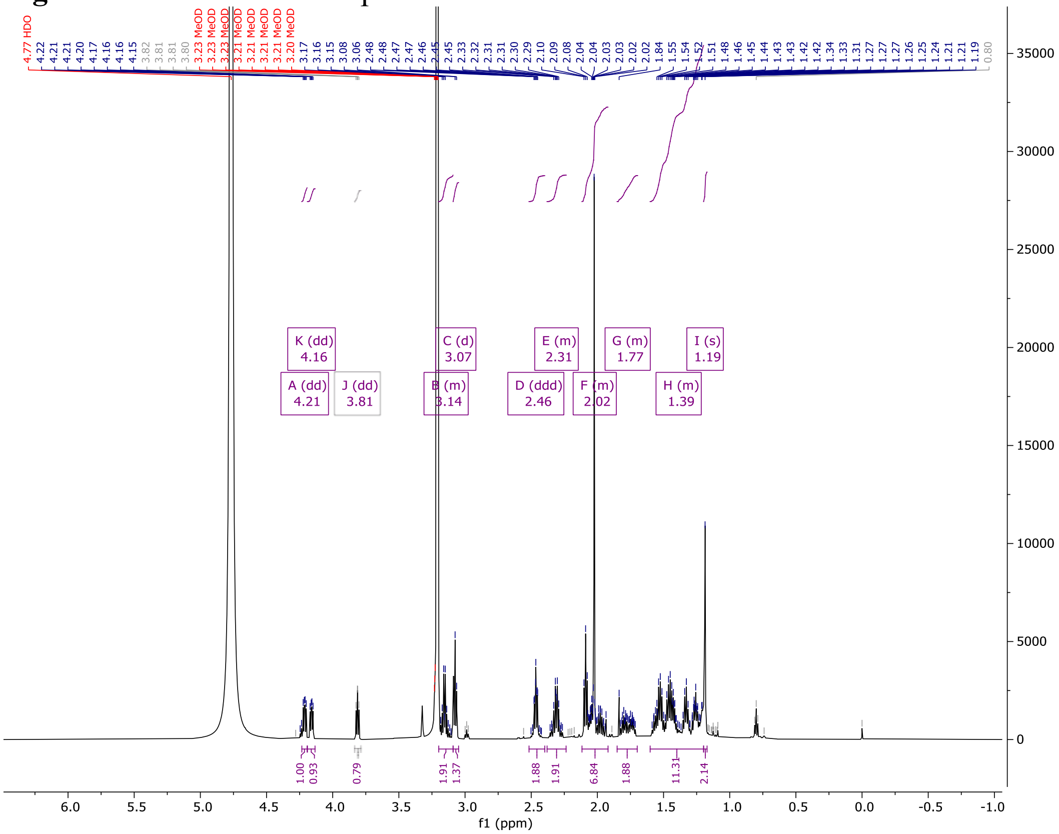

Figure S38. The ^{1}H NMR spectra of **21** in MeOD.

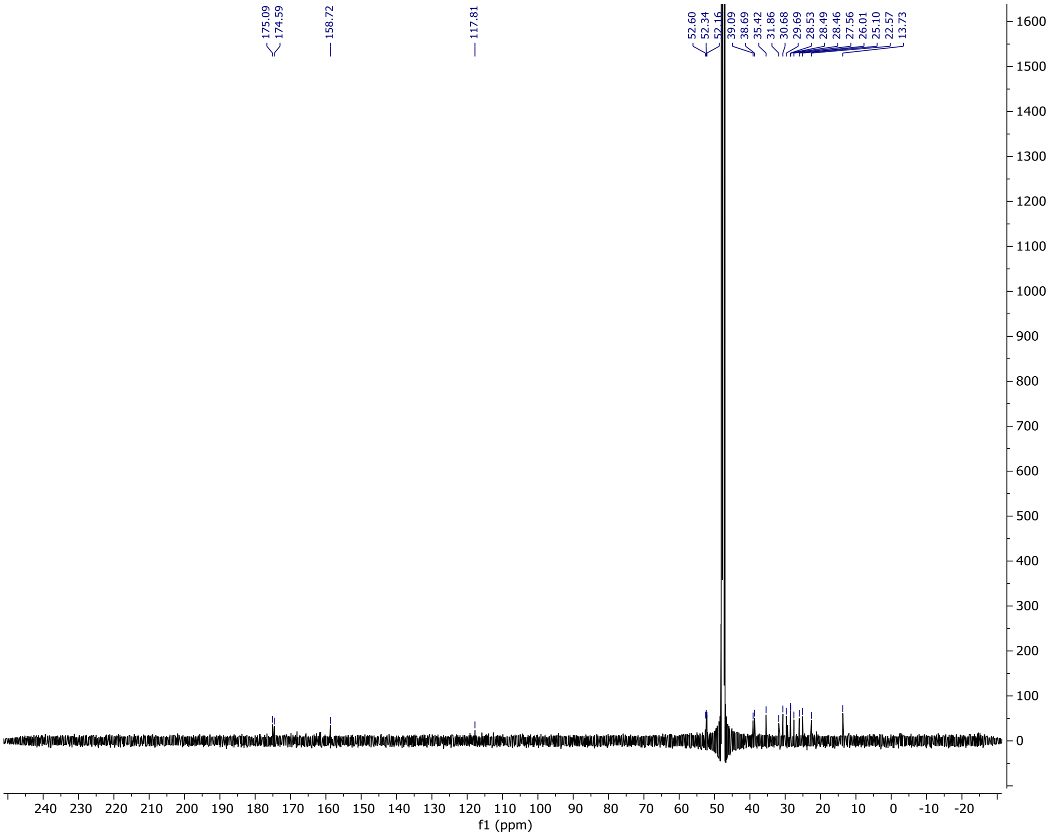

Figure S39 The $^{13}C\{^1H\}$ NMR spectrum of **21** in MeOD.

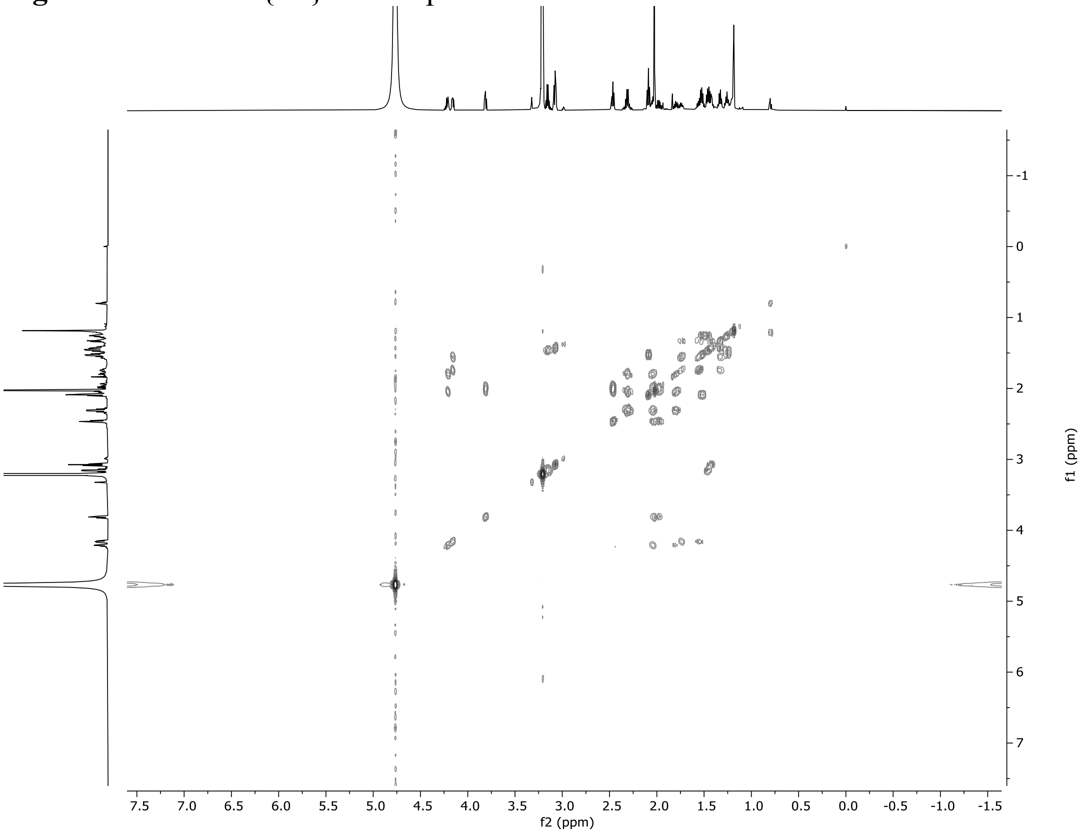

Figure S40 The ^1H-^1H COSY NMR spectrum of **21** in MeOD.

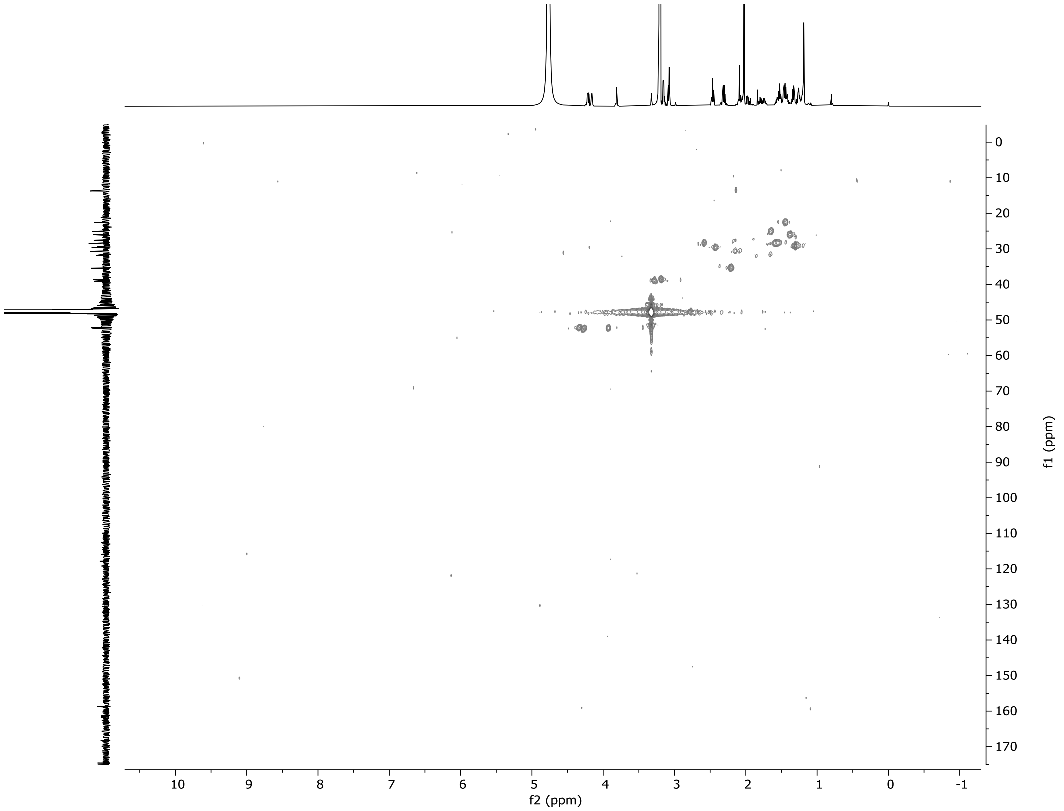

Figure S41 The ^{1}H-^{13}C HSQC NMR spectrum of **21** in MeOD.

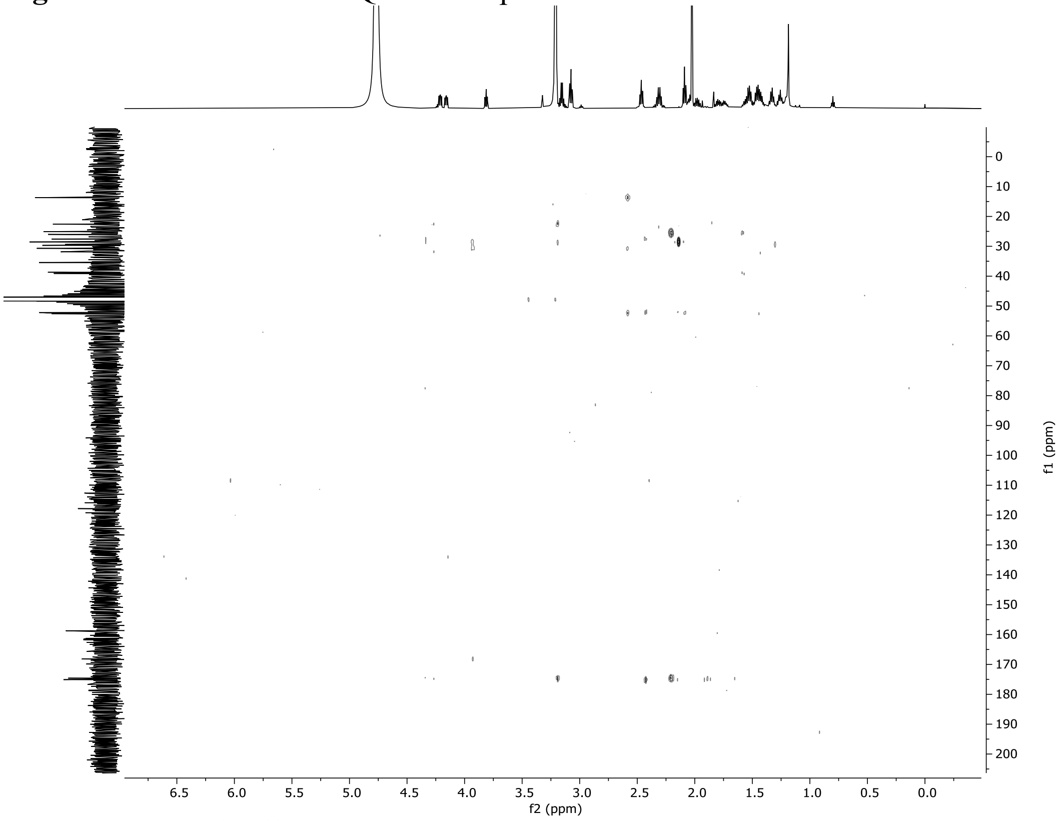

Figure S42 The ^{1}H-^{13}C HMBC NMR spectrum of **21** in MeOD.

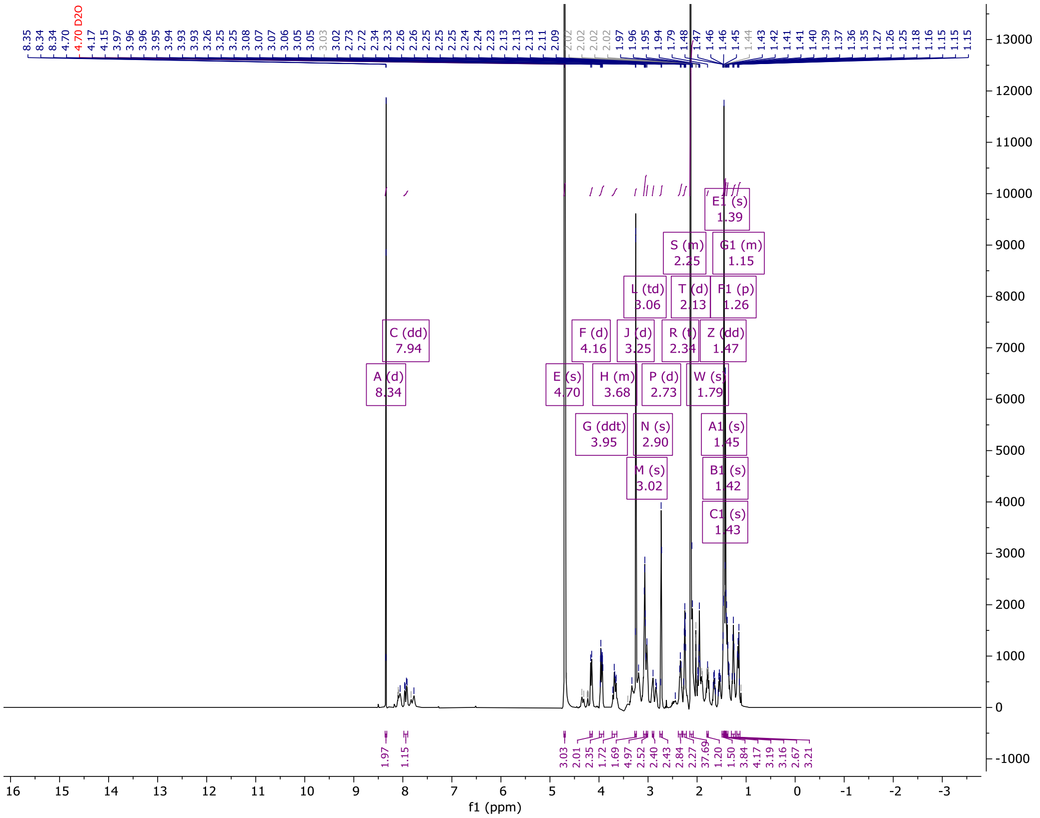

Figure S43. The ^{1}H NMR spectra of **22** in MeOD.

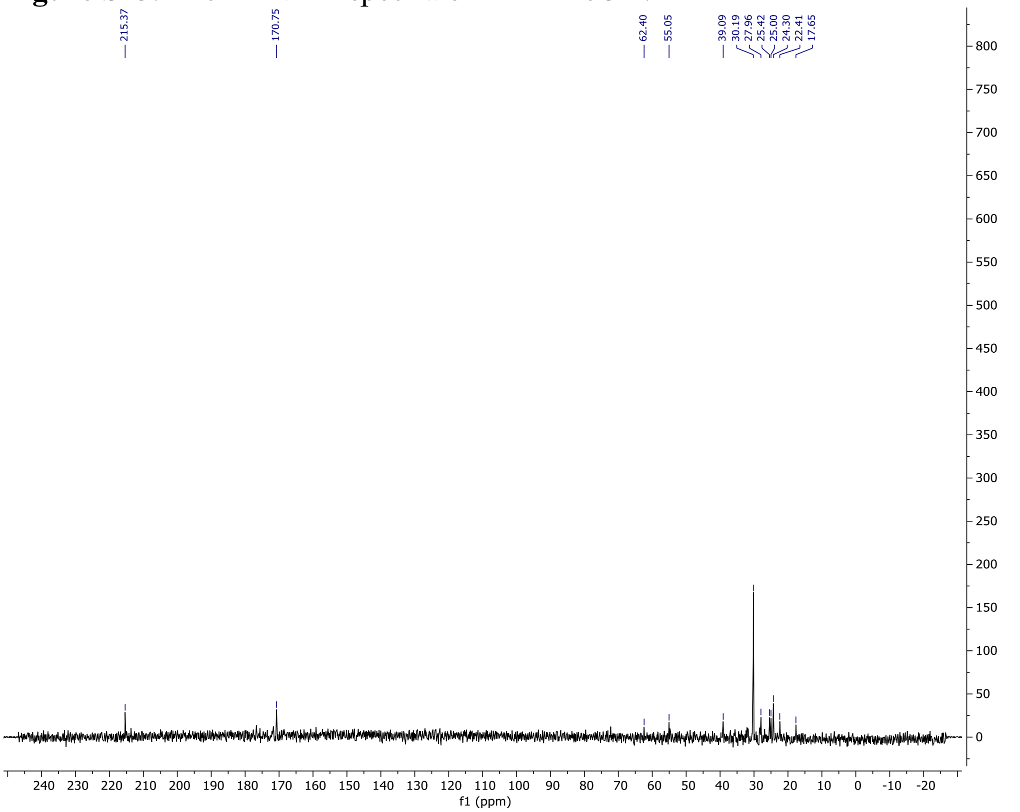

Figure S44. The 13C{^{1}H} NMR spectra of **22** in MeOD.

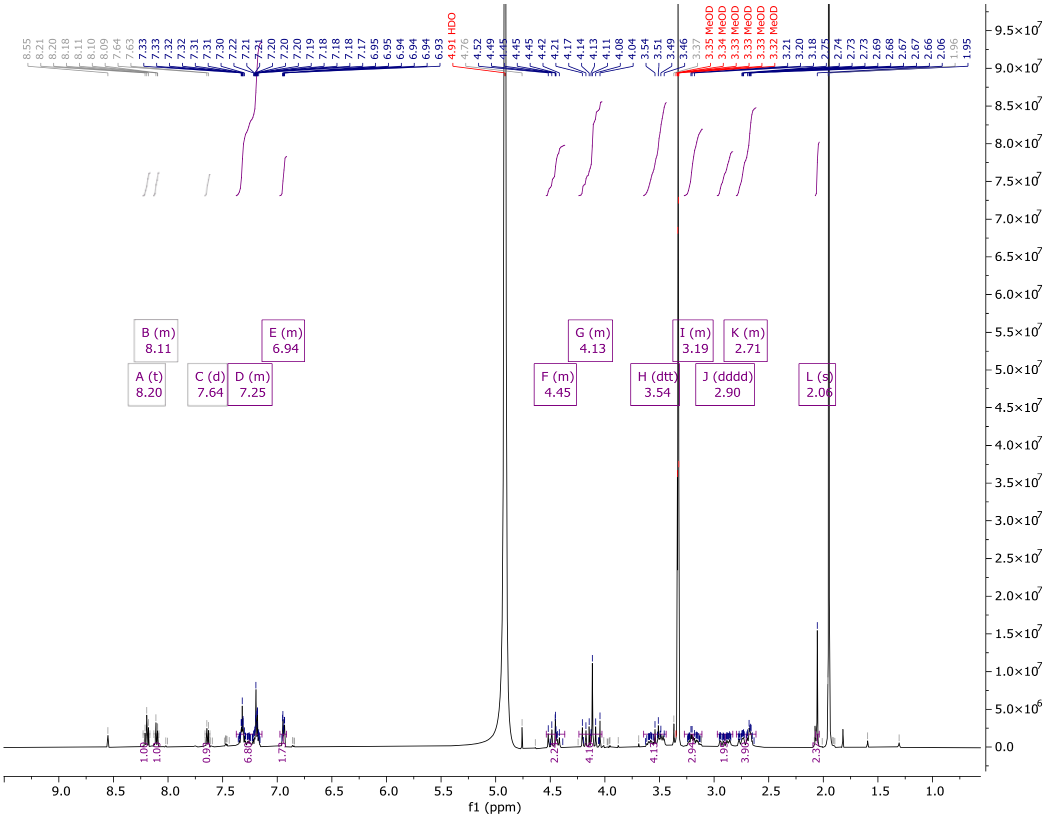

Figure S45. The ^{1}H NMR spectra of **[Sc(8)]$^{+}$** in MeOD.

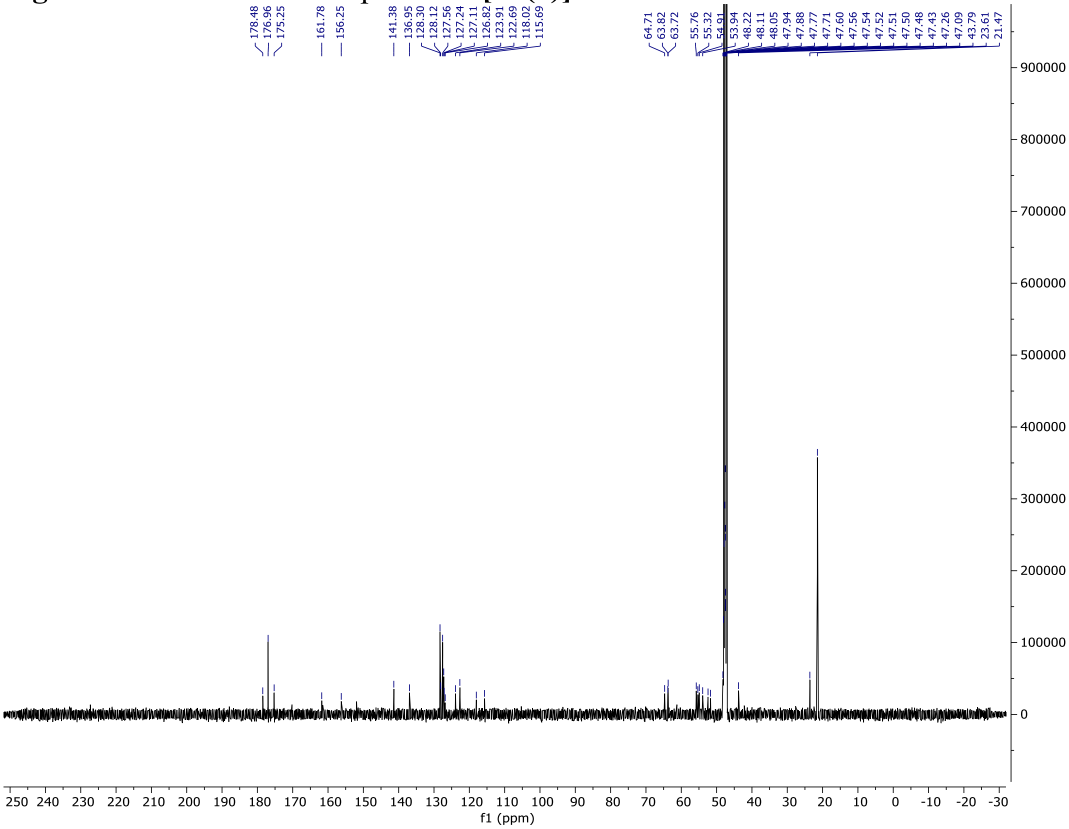

Figure S46. The $^{13}C\{^{1}H\}$ NMR spectra of **[Sc(8)]$^{+}$** in MeOD.

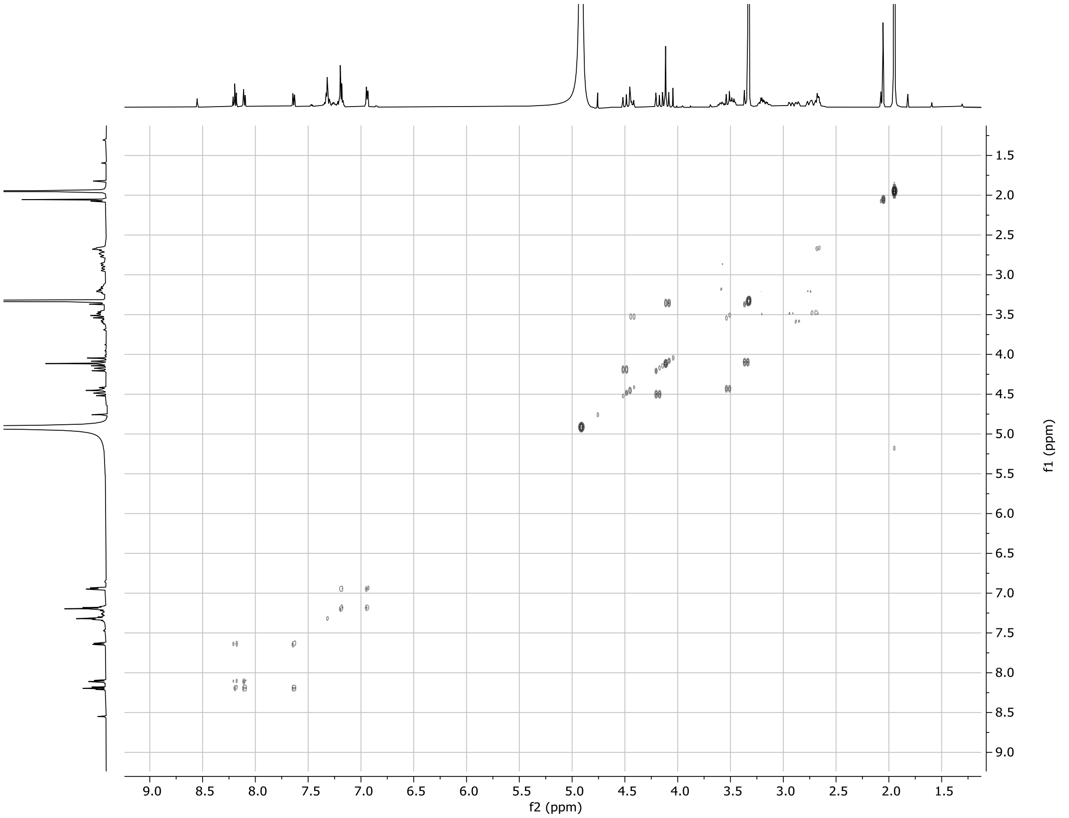

Figure S47 The ^{1}H-^{1}H COSY NMR spectrum of **[Sc(8)]**$^{+}$ in MeOD.

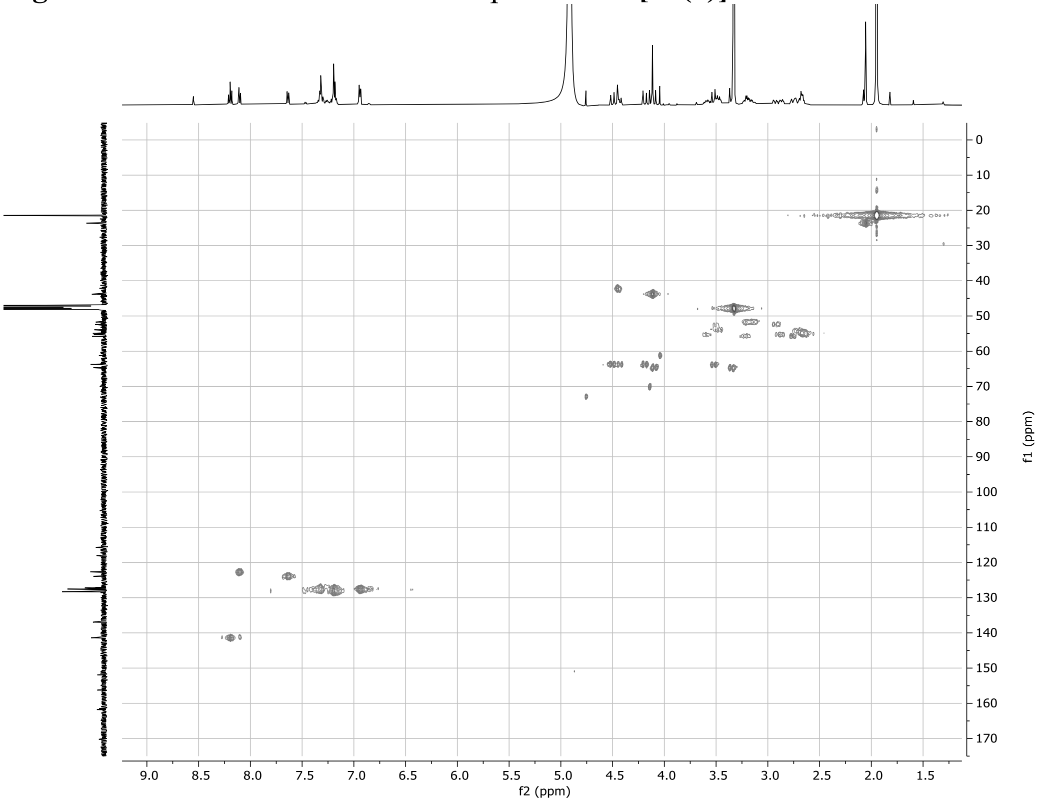

Figure S48 The ^{1}H-^{13}C HSQC NMR spectrum of **[Sc(8)]**$^{+}$ in MeOD.

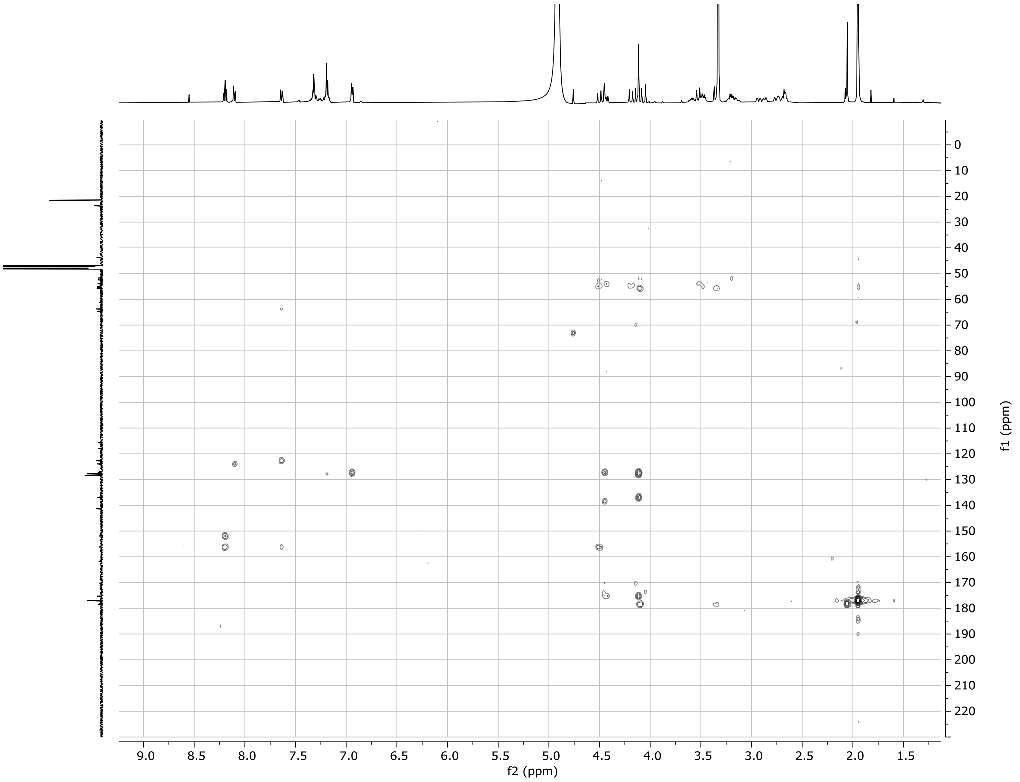

Figure S49 The ^{1}H-^{13}C HMBC NMR spectrum of **[Sc(8)]**$^{+}$ in MeOD.

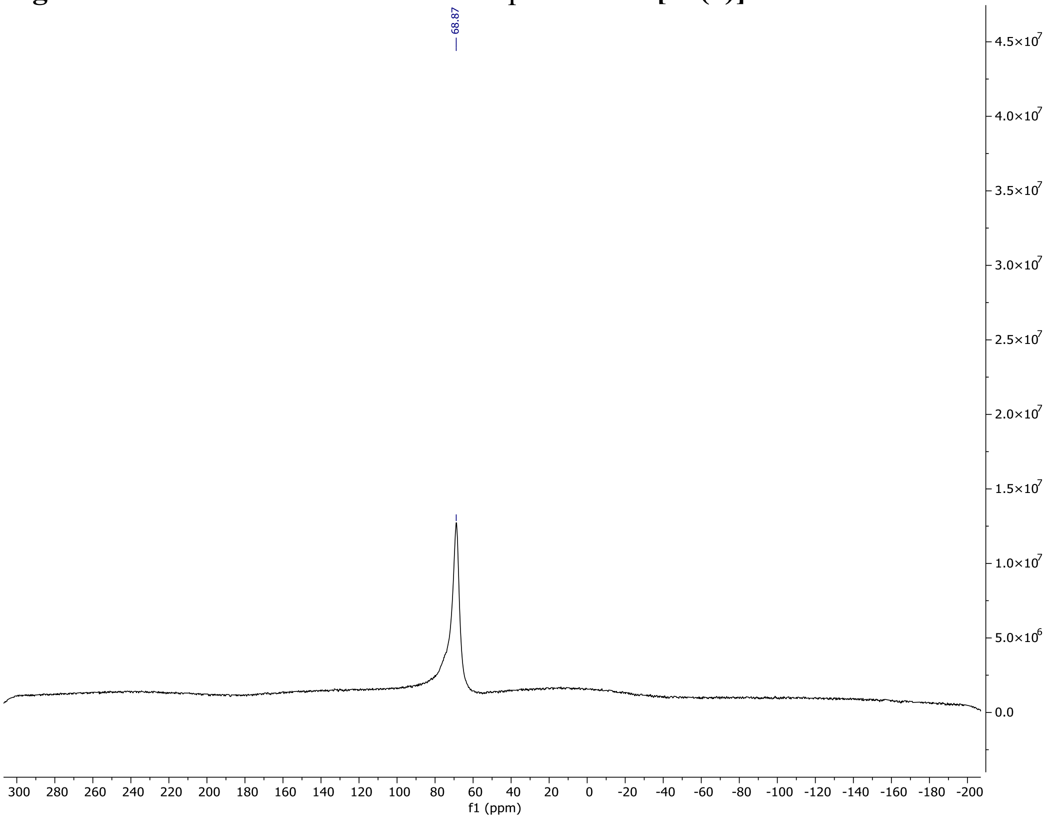

Figure S50 The ^{45}Sc NMR spectrum of **[Sc(8)]**$^{+}$ in MeOD.

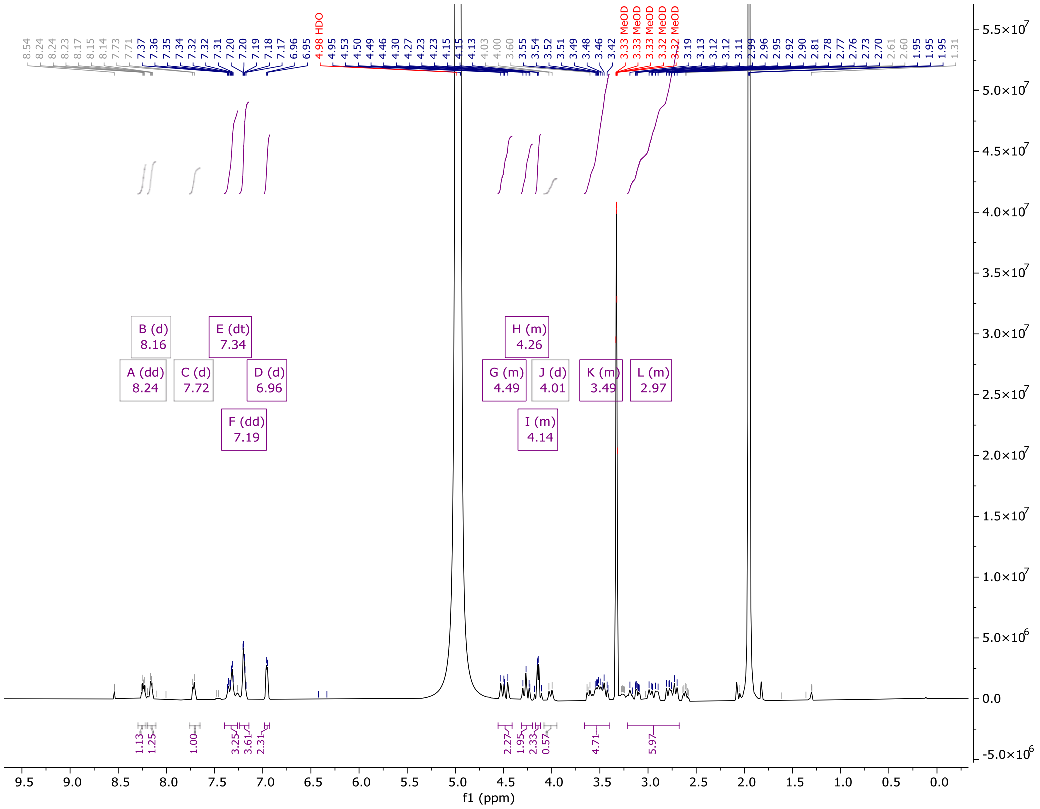

Figure S51. The ^{1}H NMR spectra of **[Lu(8)]$^{+}$** in MeOD.

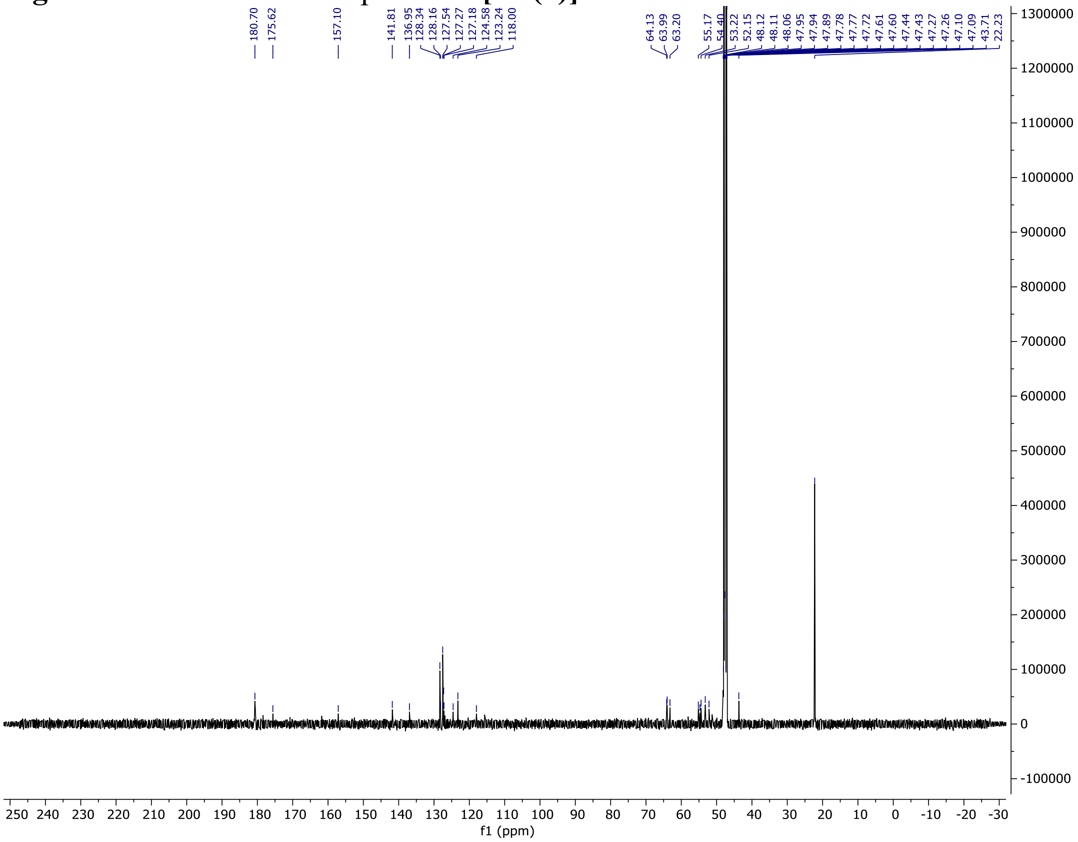

Figure S52. The $^{13}C\{^{1}H\}$ NMR spectra of **[Lu(8)]$^{+}$** in MeOD

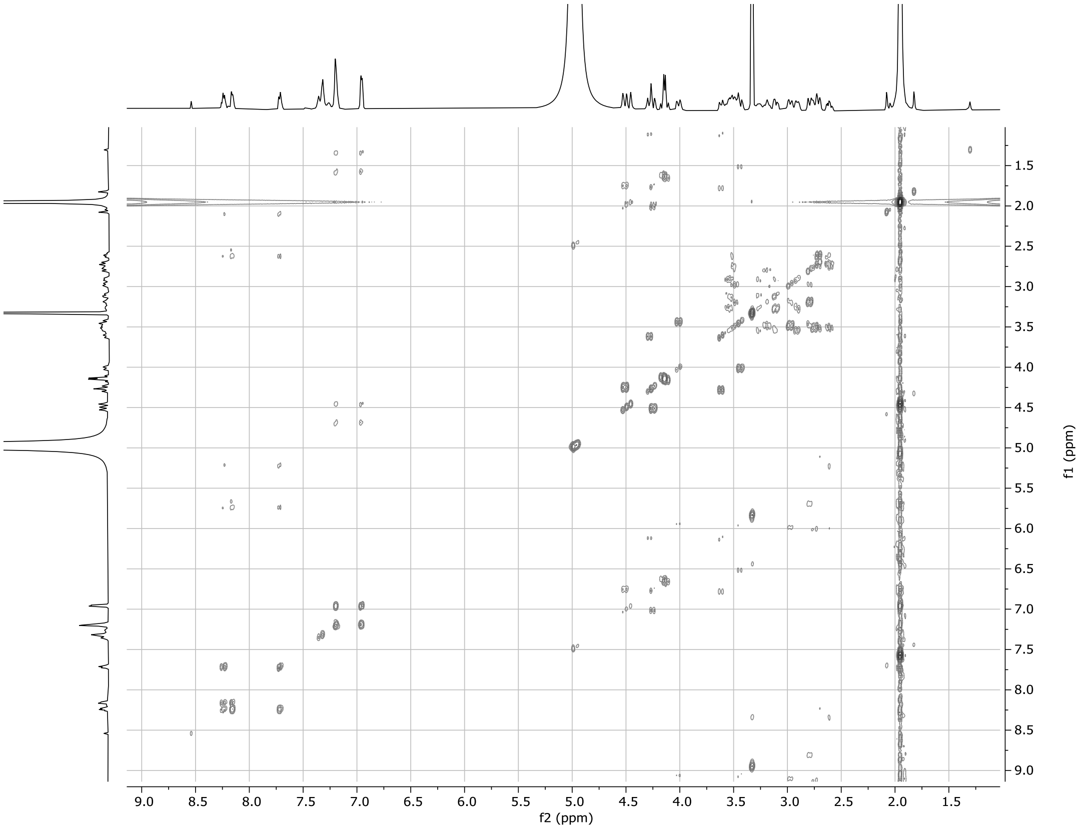

Figure S53 The ^{1}H-^{1}H COSY NMR spectrum of **[Lu(8)]**$^{+}$ in MeOD.

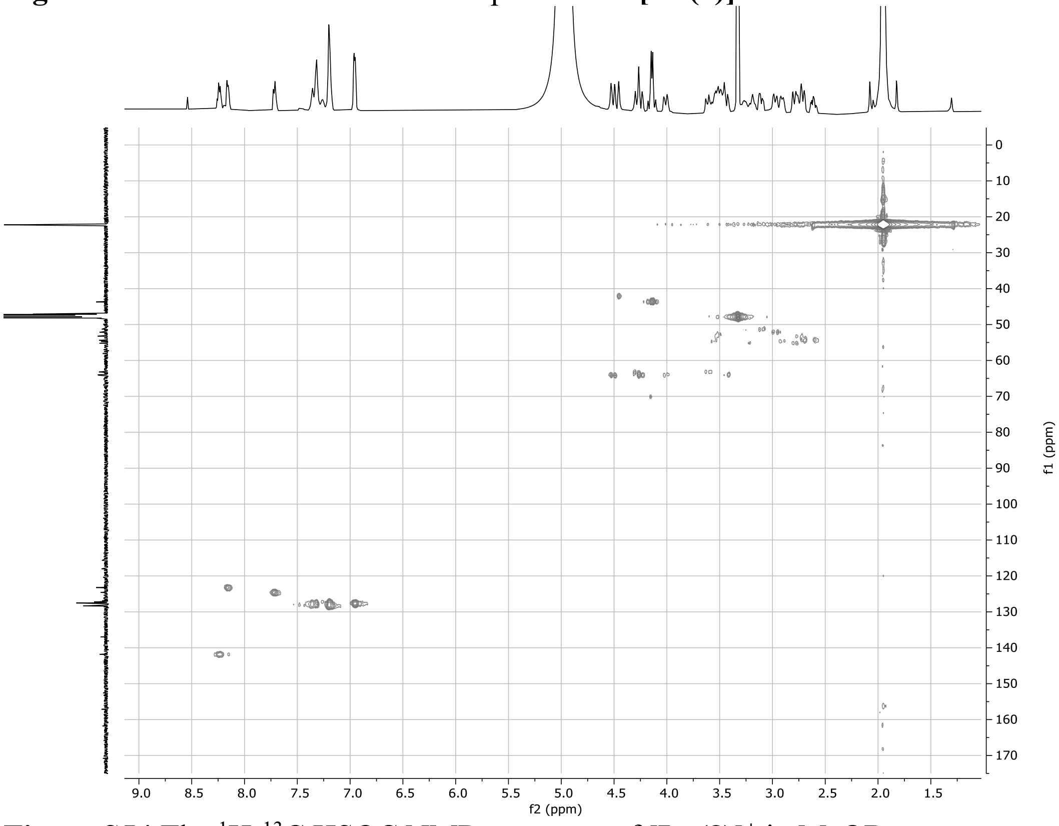

Figure S54 The ^{1}H-^{13}C HSQC NMR spectrum of **[Lu(8)]**$^{+}$ in MeOD

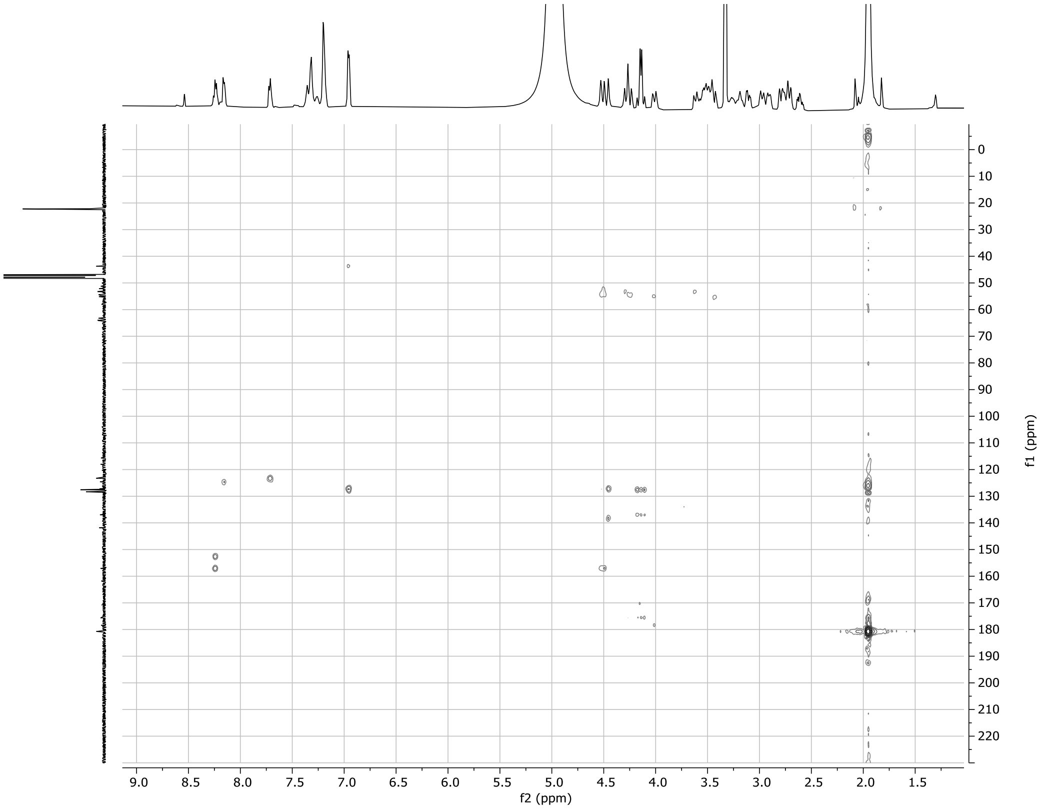

Figure S55 The ^{1}H-^{13}C HMBC NMR spectrum of **[Lu(8)]**$^+$ in MeOD

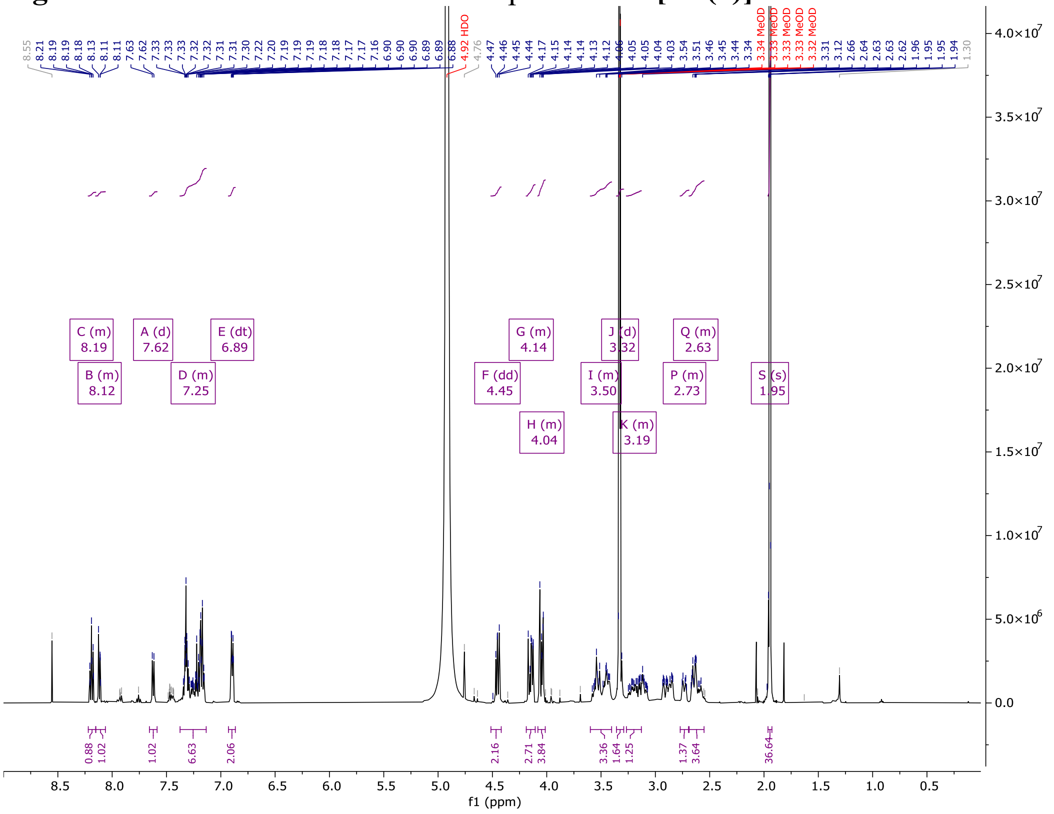

Figure S56. The ^{1}H NMR spectra of **[ScF(8)]** in MeOD.

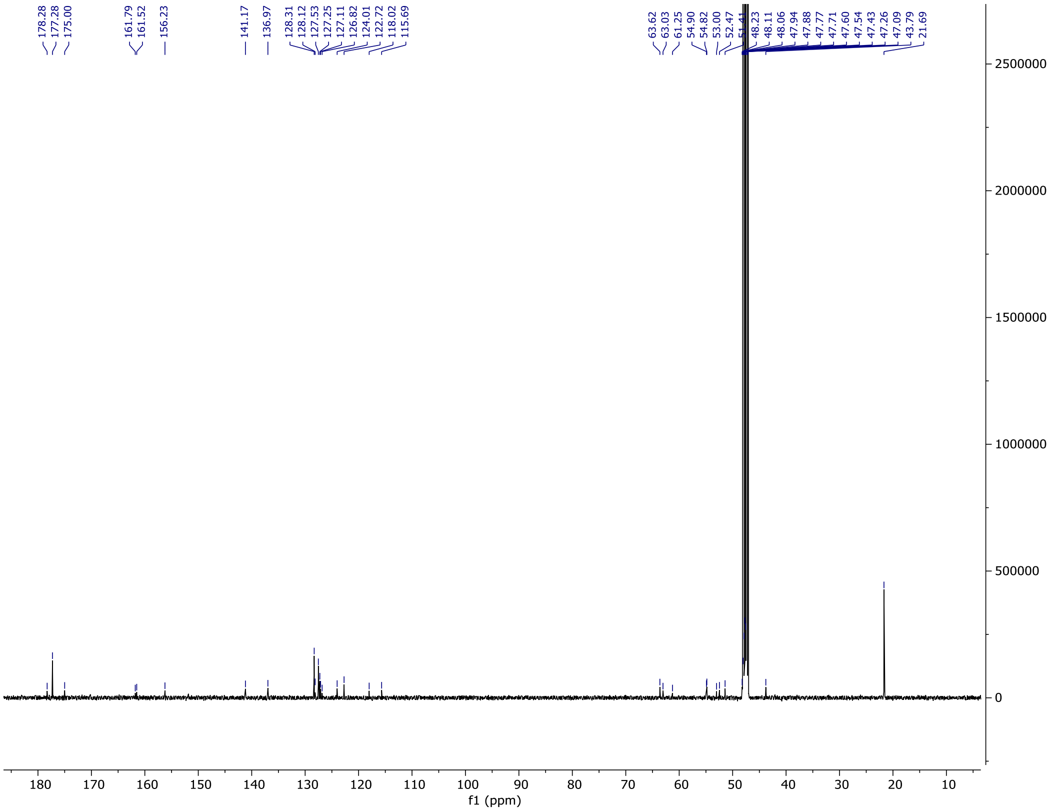

Figure S57. The $^{13}C\{^{1}H\}$ NMR spectra of **[ScF(8)]** in MeOD.

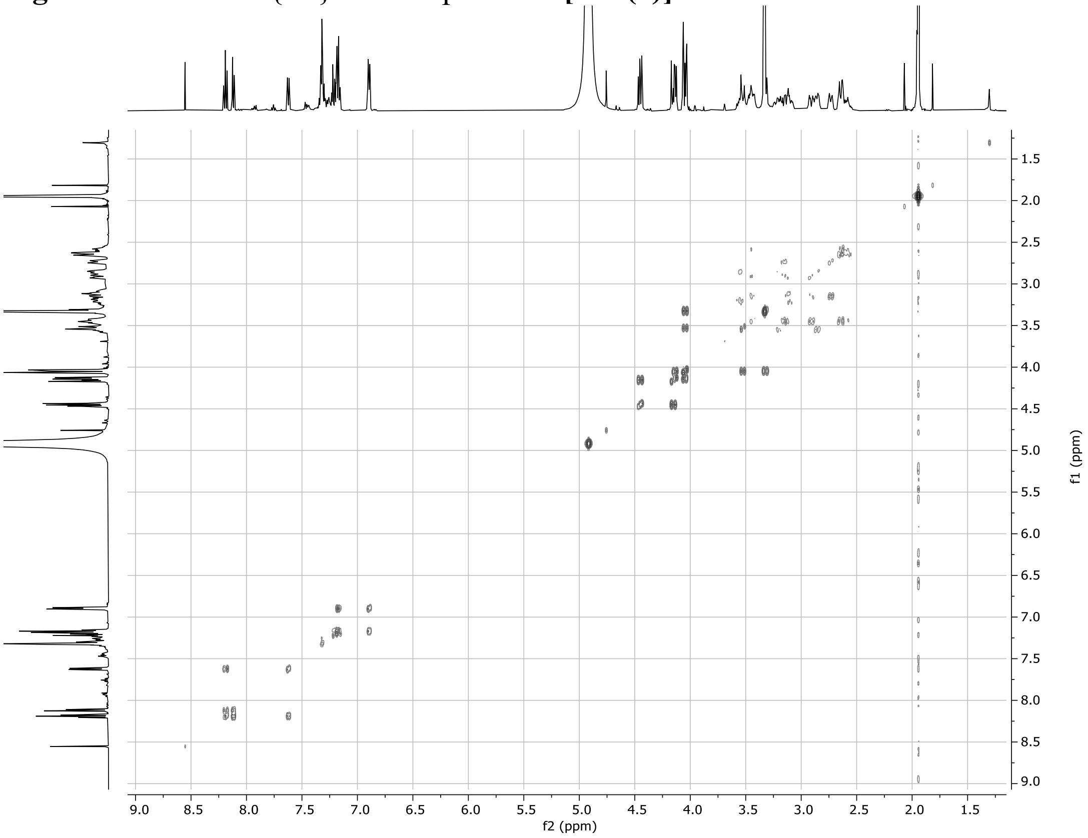

Figure S58 The ^{1}H-^{1}H COSY NMR spectrum of **[ScF(8)]** in MeOD.

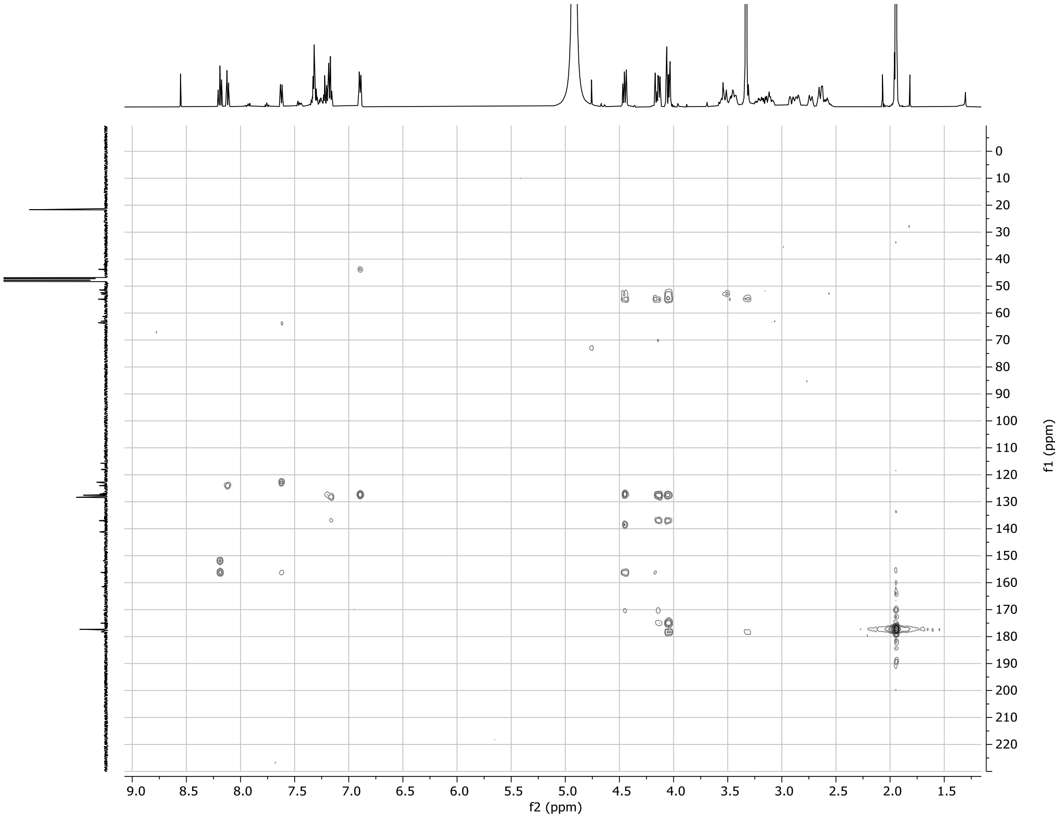

Figure S59 The ^{1}H-^{13}C HSQC NMR spectrum of **[ScF(8)]** in MeOD.

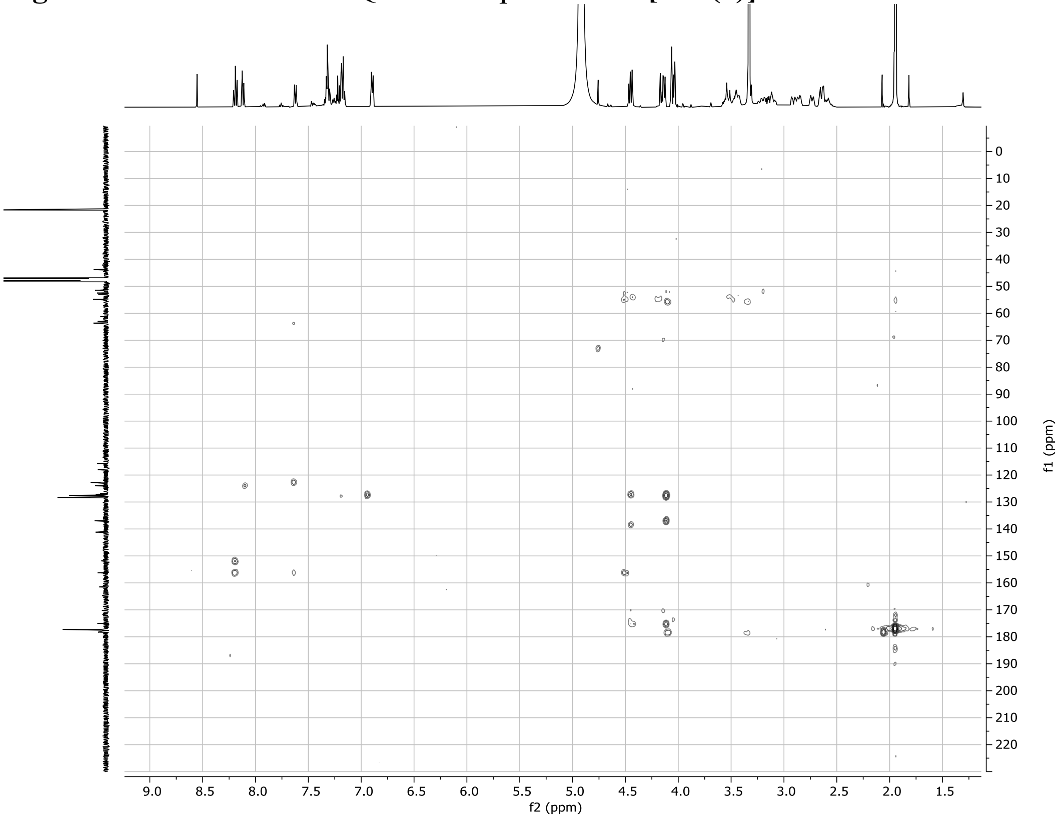

Figure S60 The ^{1}H-^{13}C HMBC NMR spectrum of **[ScF(8)]** in MeOD.

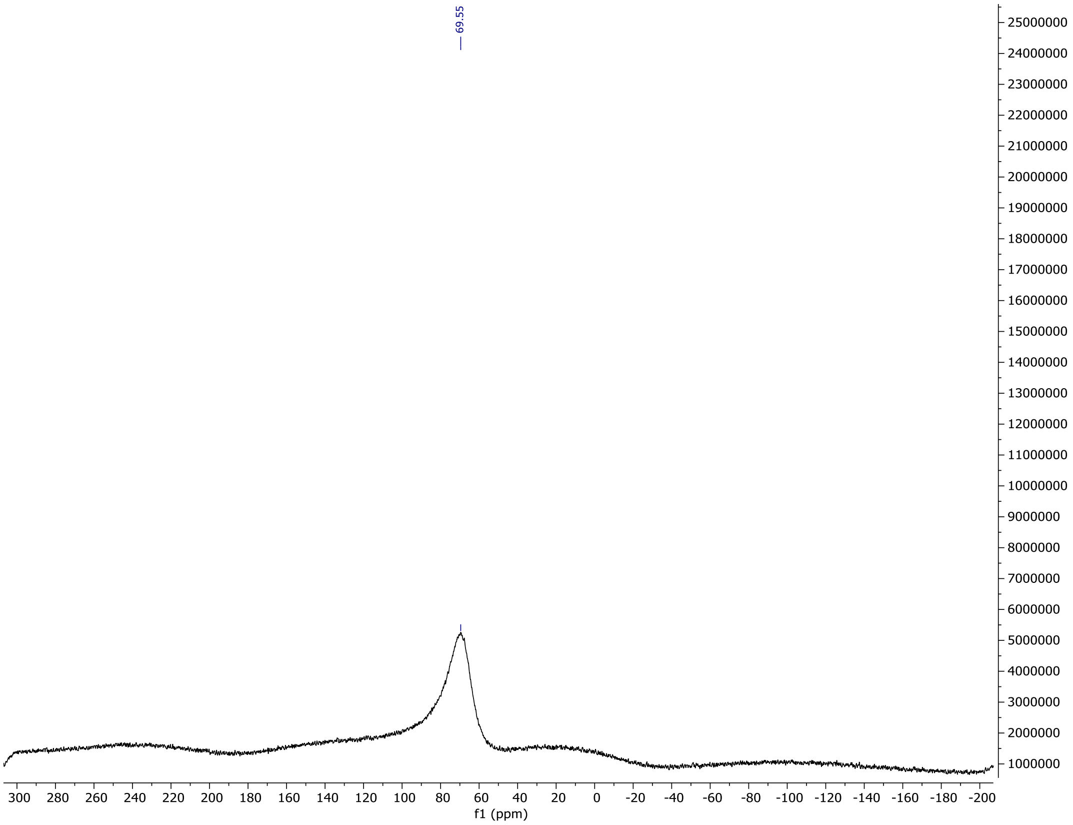

Figure S61 The ^{45}Sc NMR spectrum of **[ScF(8)]** in MeOD.

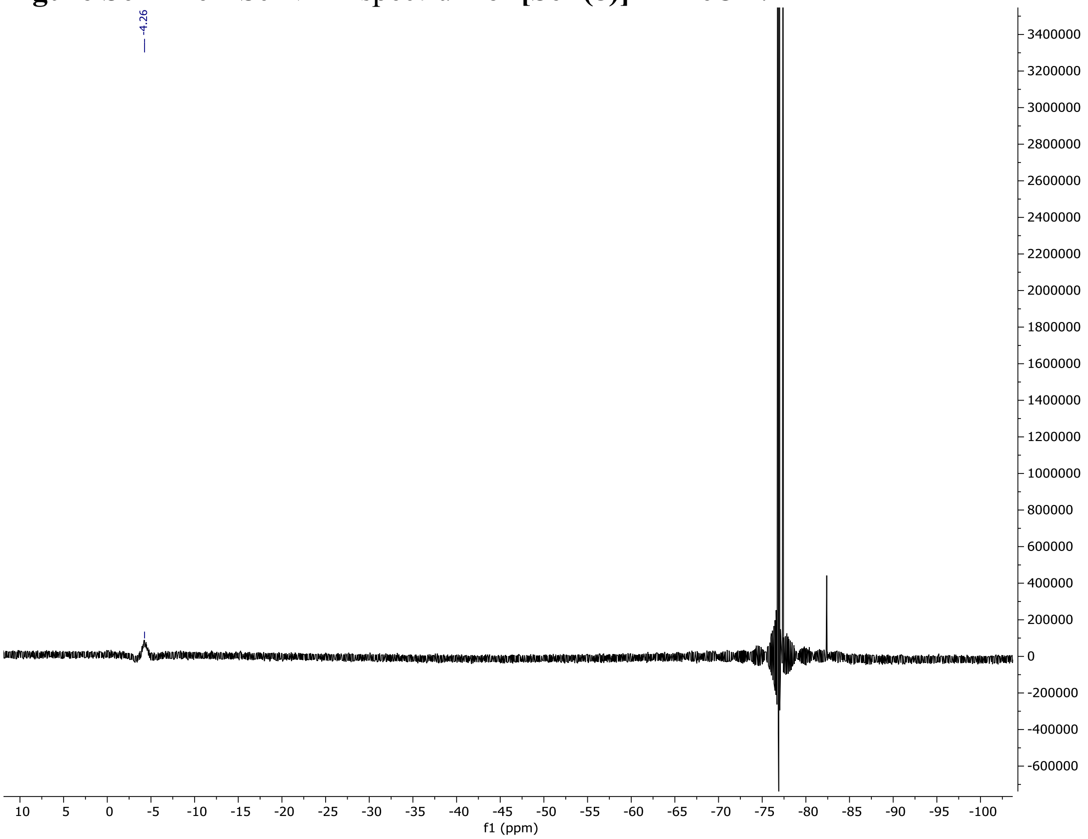

Figure S62 The ^{19}F NMR spectrum of **[ScF(8)]** in MeOD.

2.2 HRMS and LRMS

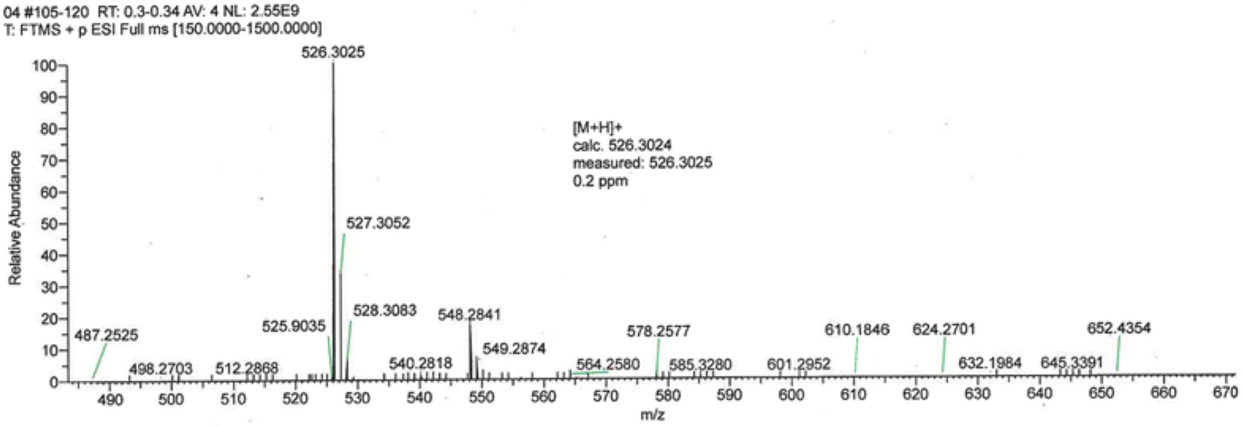

Figure S63. ESI - HRMS report for **7.**

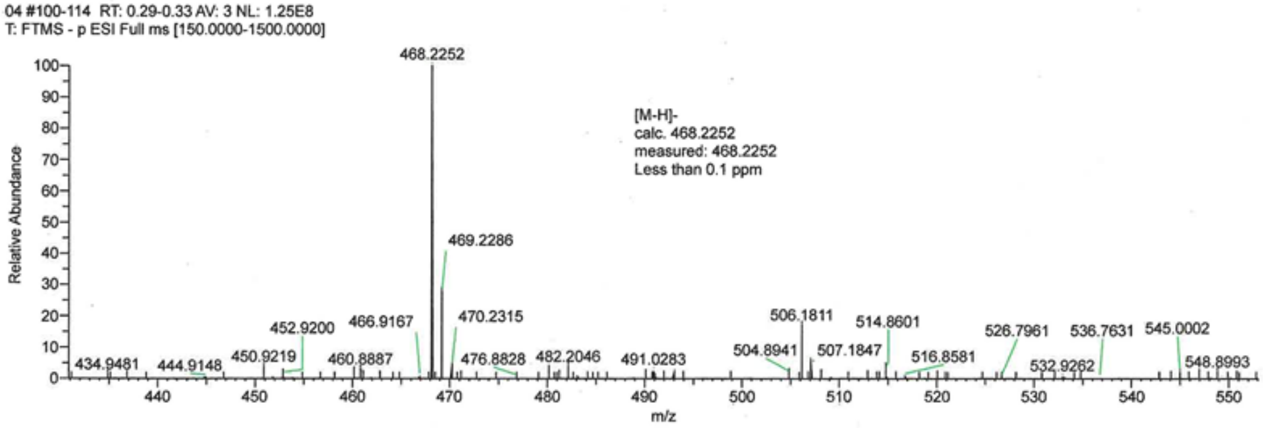

Figure S64. ESI - HRMS report for **8.**

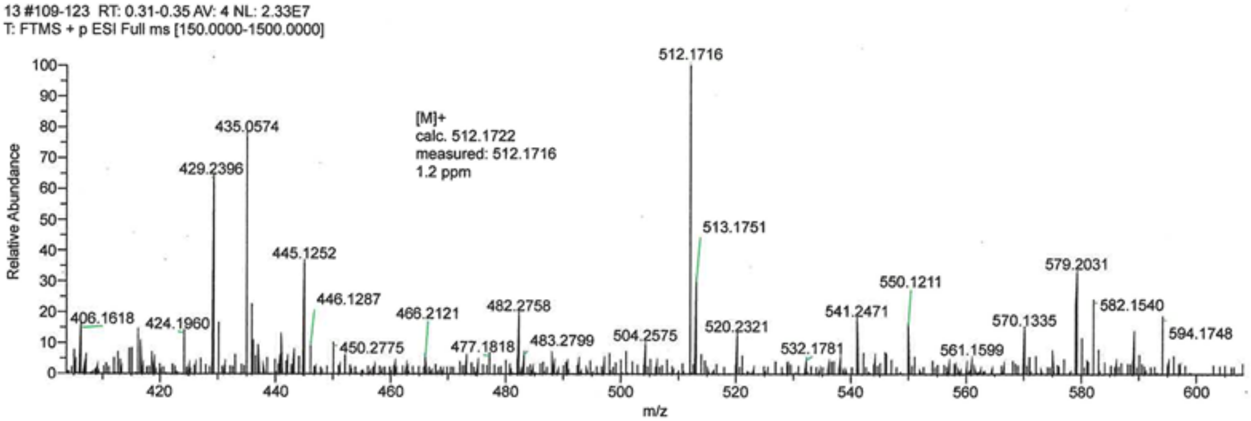

Figure S65. ESI - HRMS report for **[Sc(8)]$^+$.**

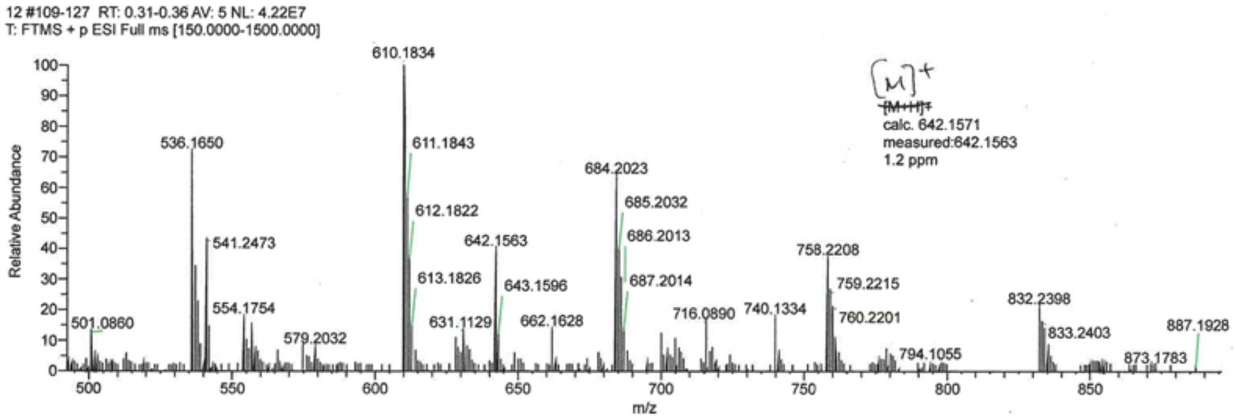

Figure S66. ESI - HRMS report for **[Lu(8)]$^+$.**

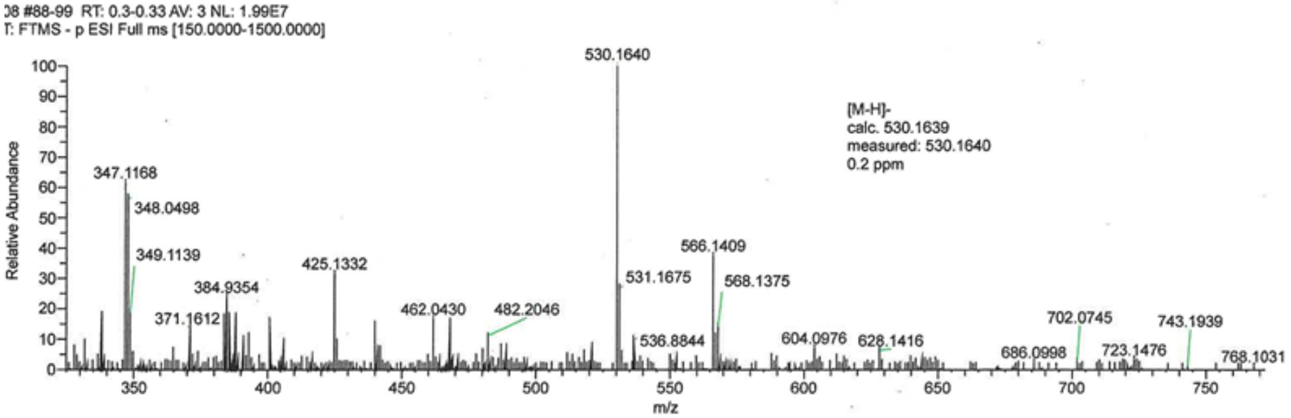

Figure S67. ESI - HRMS report for **[ScF(8)].**

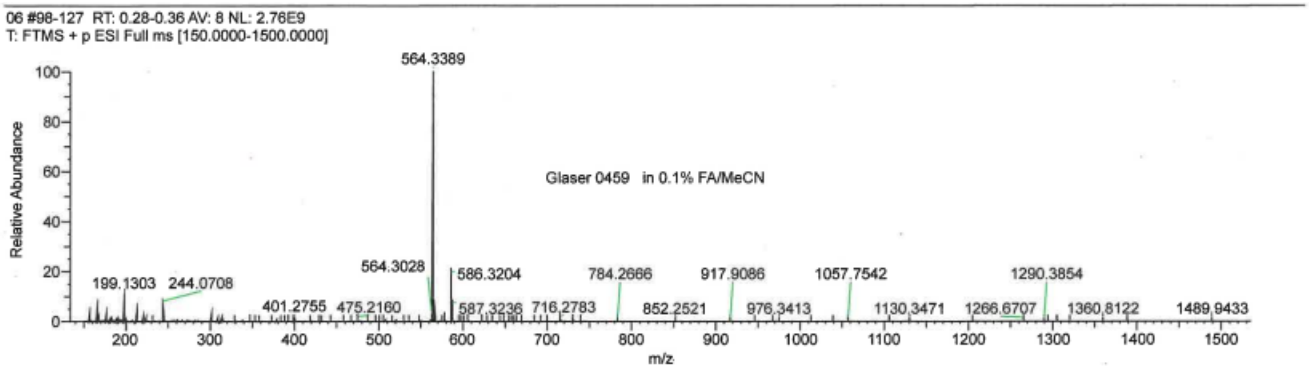

Figure S68. ESI - HRMS report for **10.**

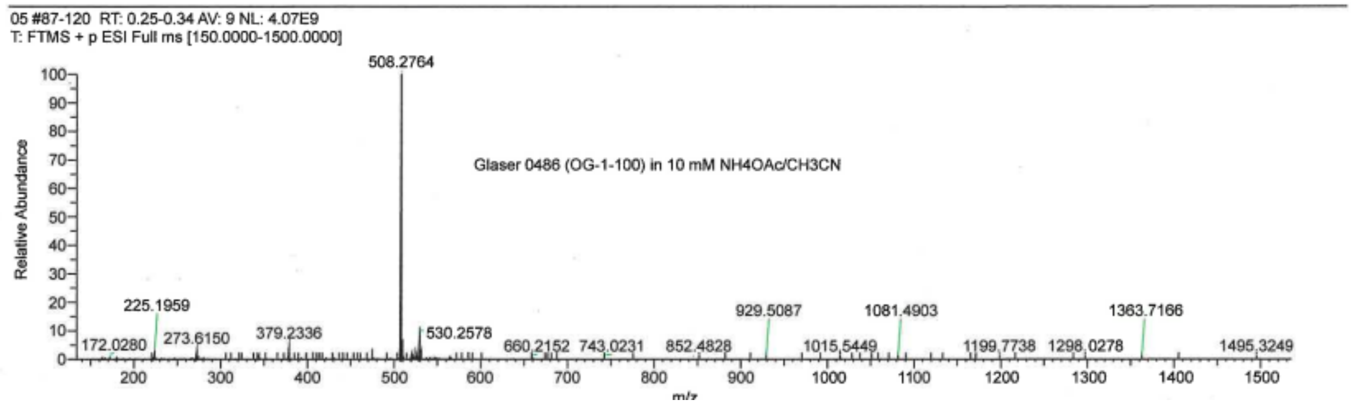

Figure S69. ESI - HRMS report for **11.**

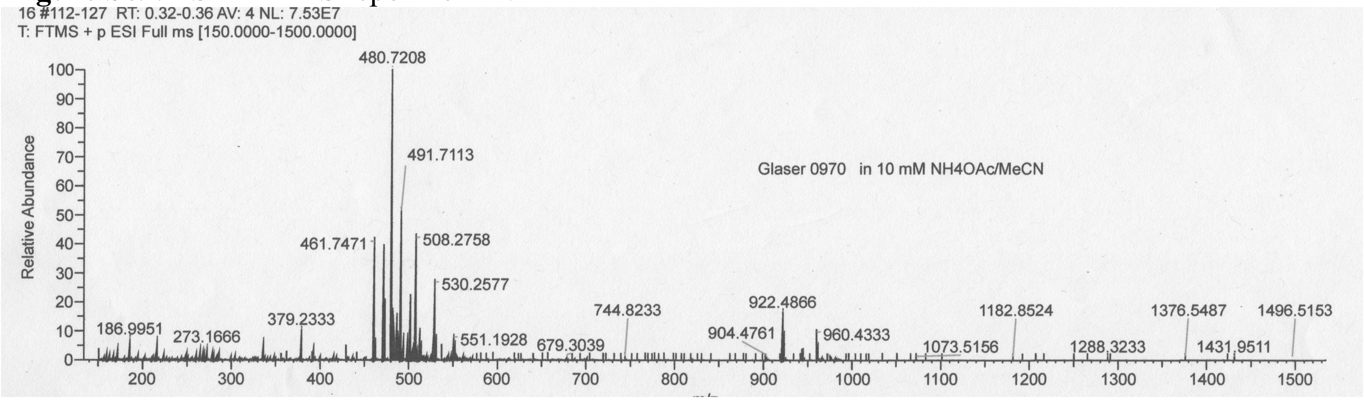

Figure S70. ESI - HRMS report for **13.**

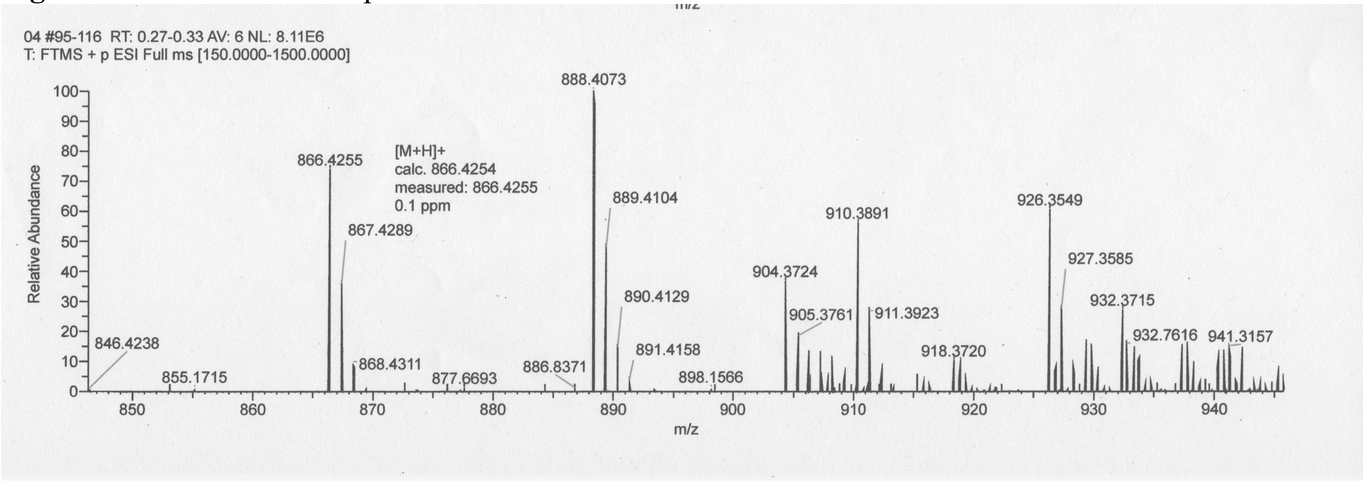

Figure S71. ESI - HRMS report for **14.**

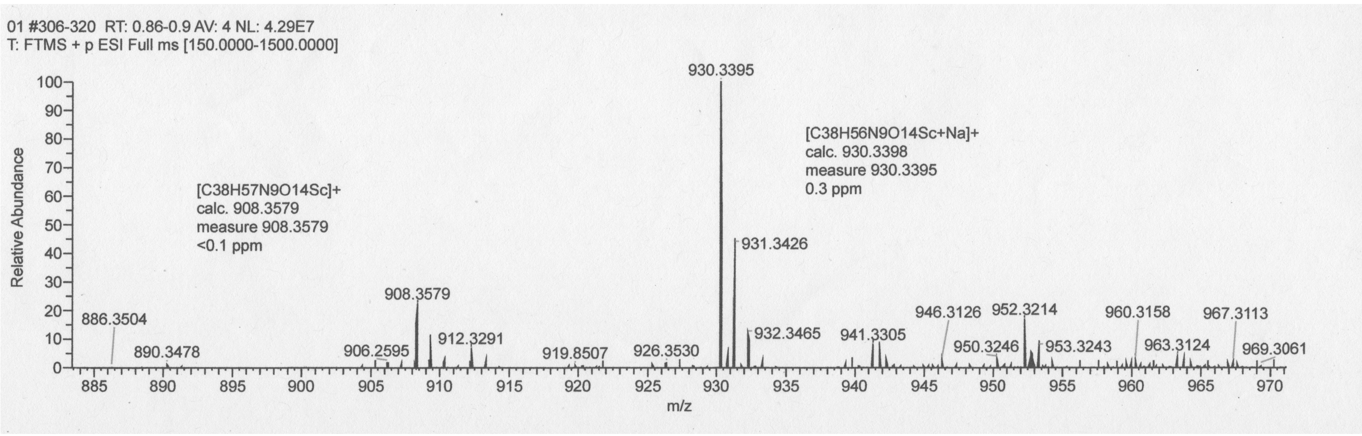

Figure S72. ESI - HRMS report for **[Sc(14)]** $^+$.

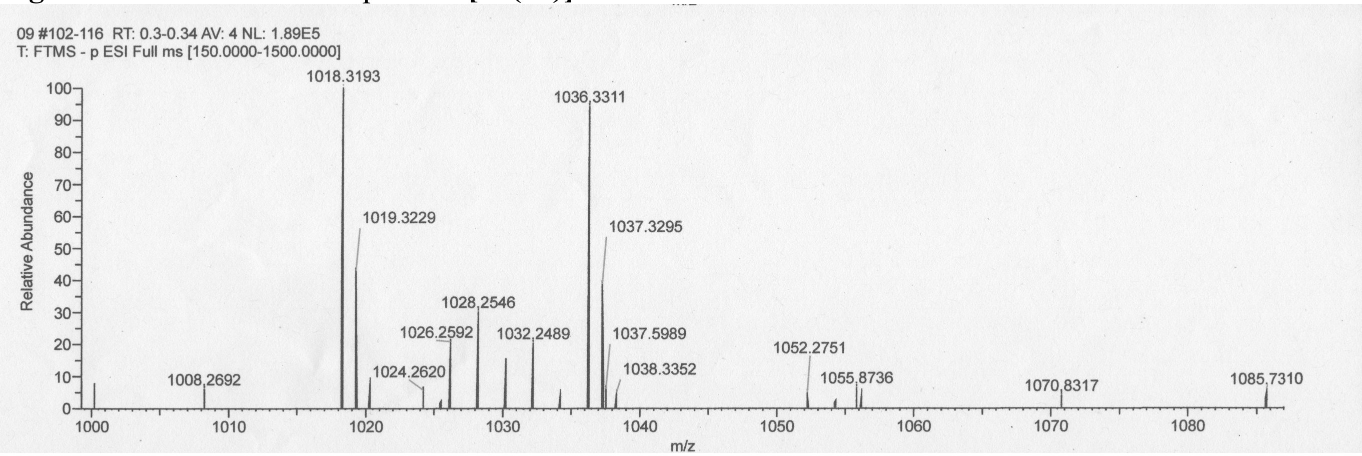

Figure S73. ESI - HRMS report for **[Lu(14)]**$^+$.

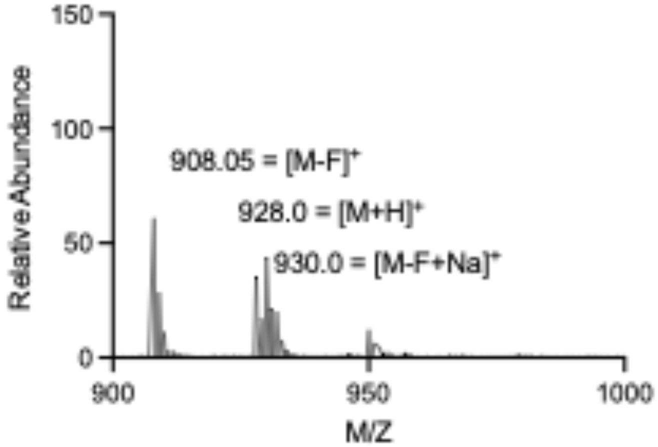

Figure S74. ESI - LRMS report for **[ScF(14)].**

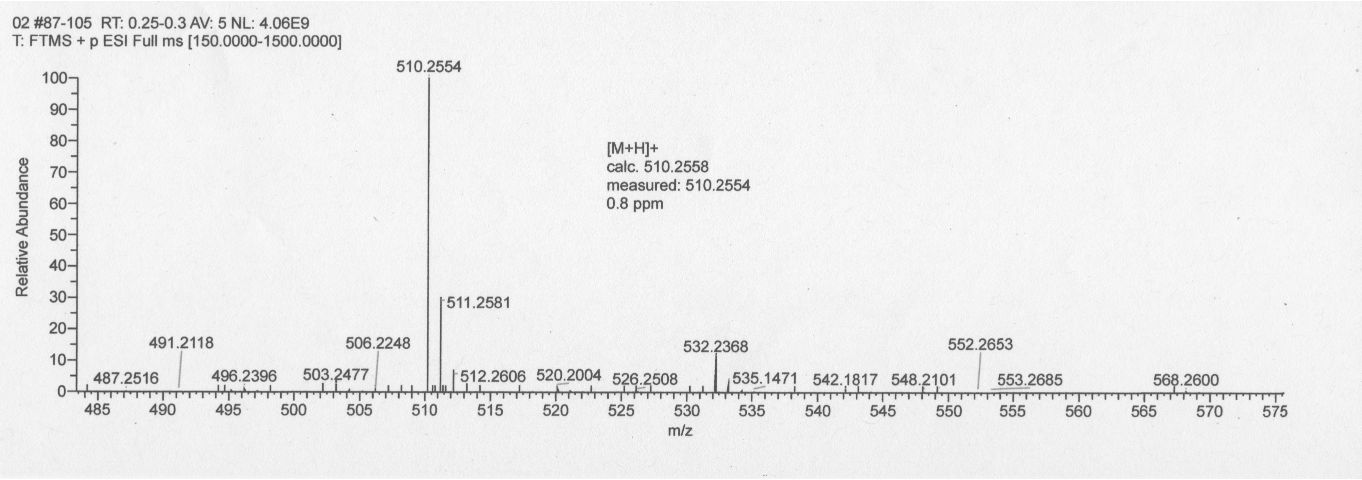

Figure S75. ESI - HRMS report for **16.**

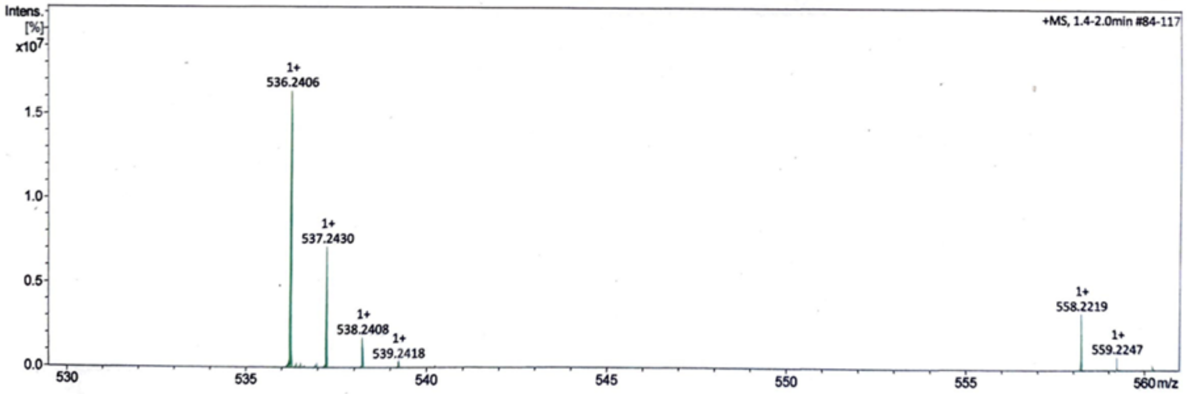

Figure S76. ESI - HRMS report for **17.**

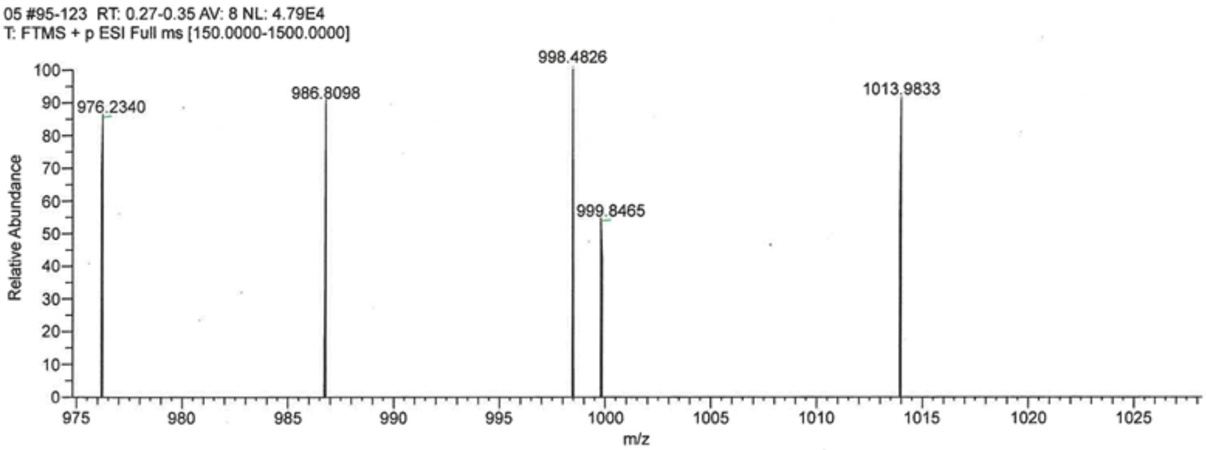

Figure S77. ESI - HRMS report for **18.**

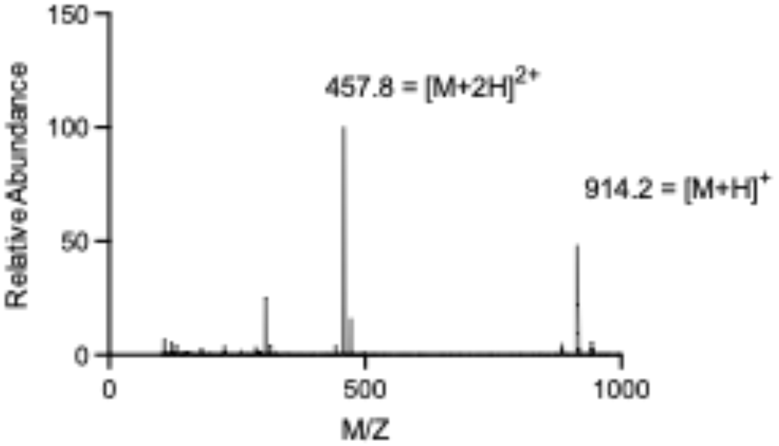

Figure S78. ESI - LRMS report for **19.**

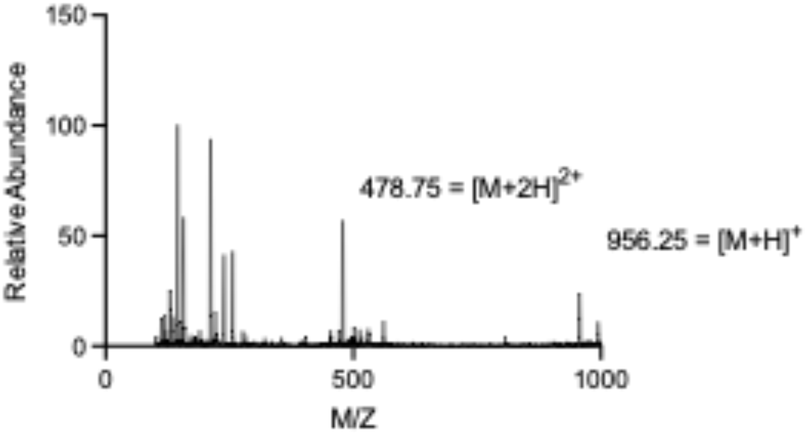

Figure S79. ESI - LRMS report for **[Sc(19)].**

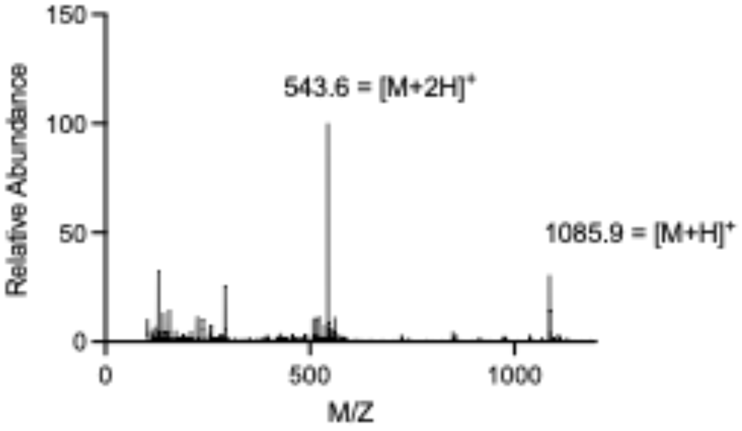

Figure S80. ESI - LRMS report for **[Lu(19)].**

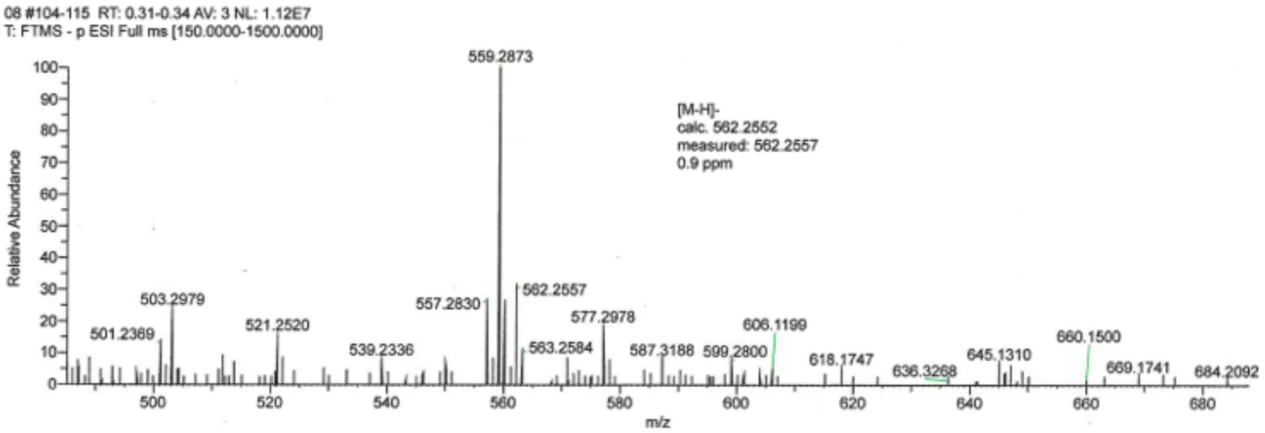

Figure S81. ESI - LRMS report for **21.**

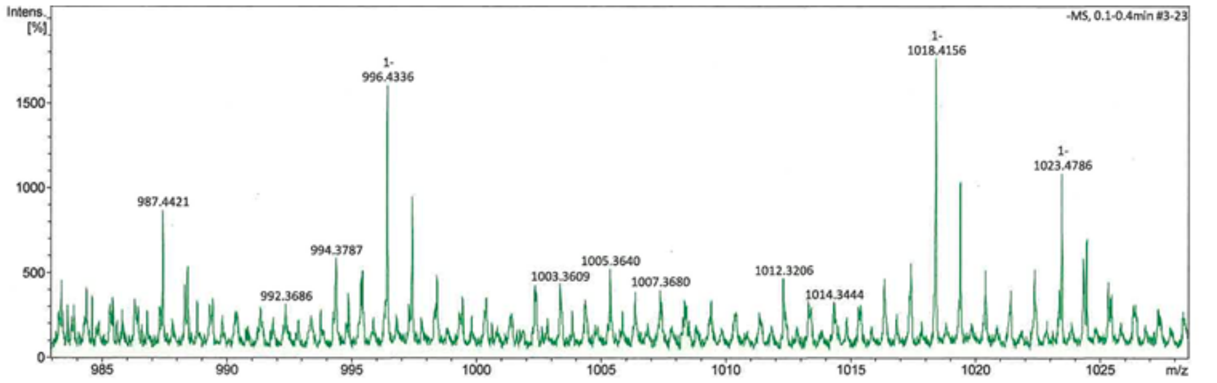

Figure S82. ESI - HRMS report for **22.**

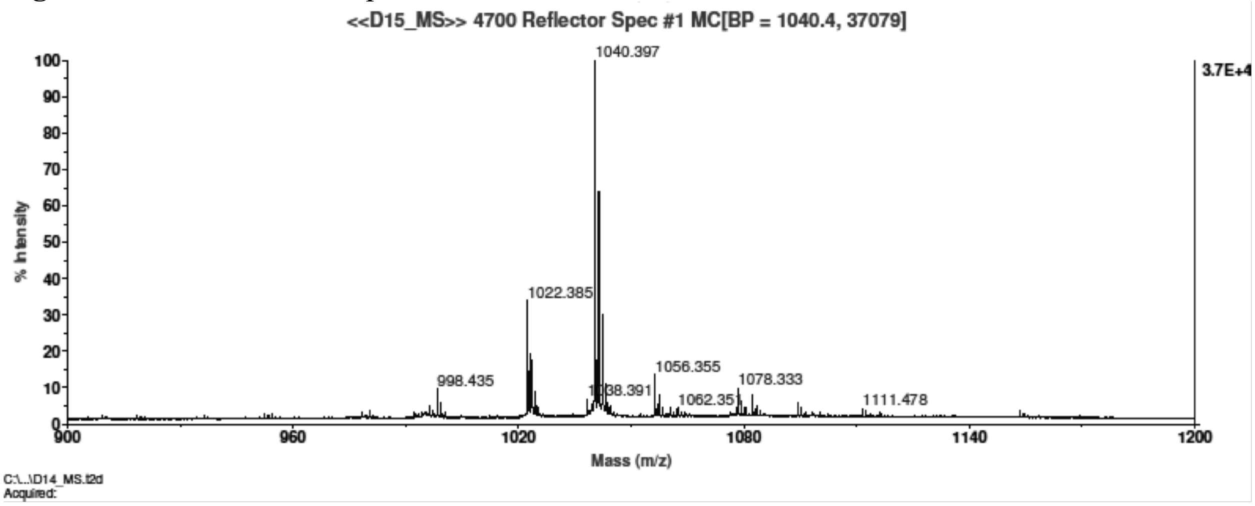

Figure S83. ESI - HRMS report for **[Sc(22)].**

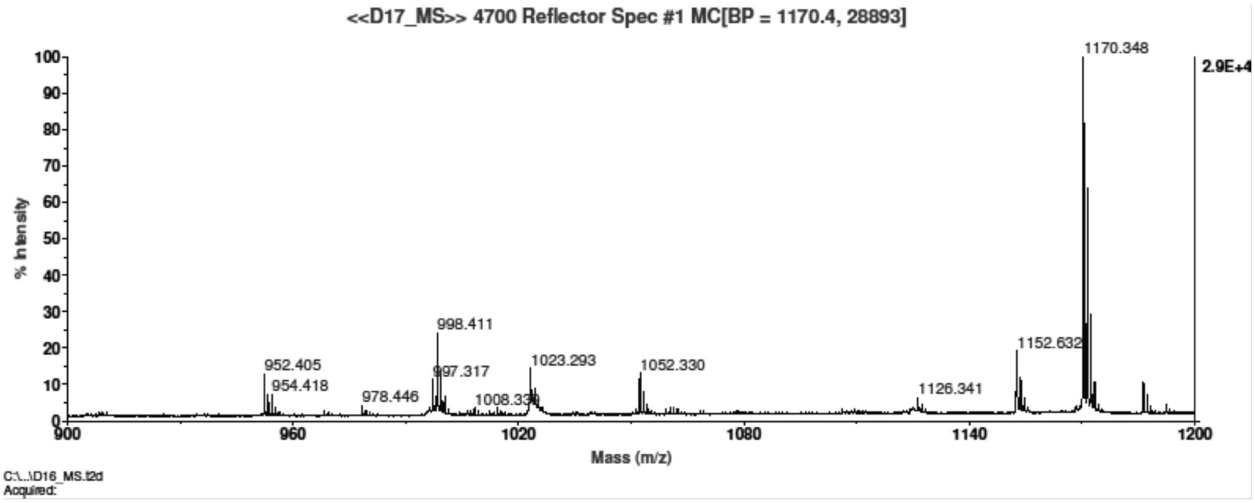

Figure S84. ESI - HRMS report for **[Lu(22)].**

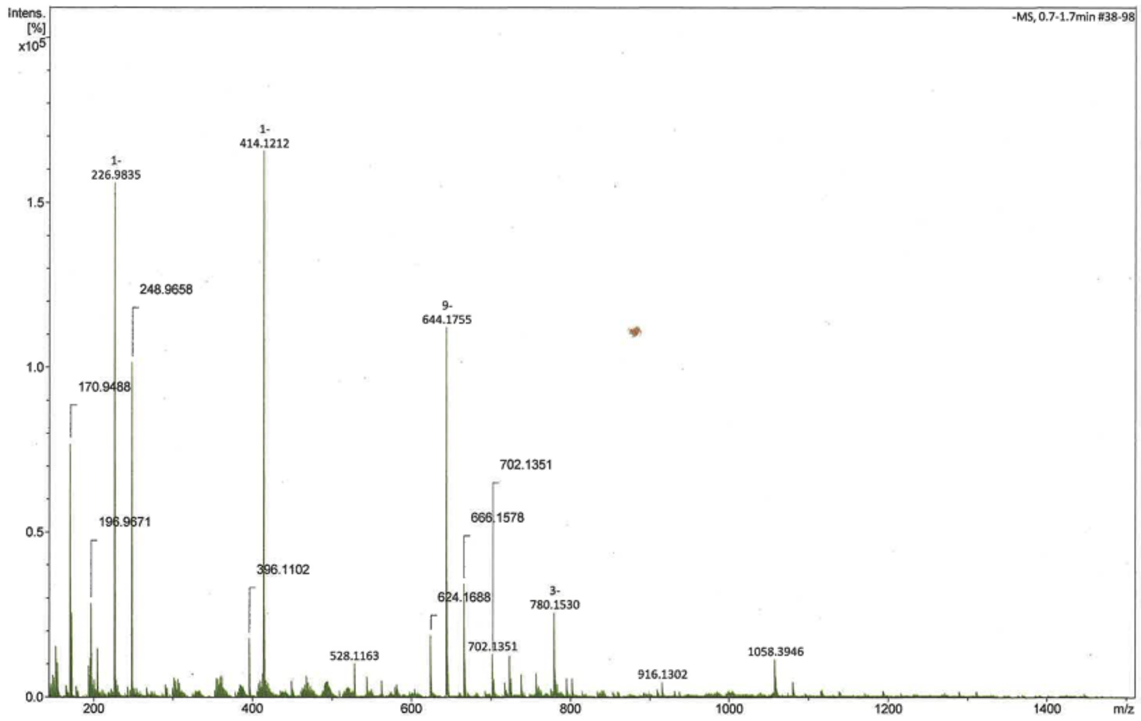

Figure S85. ESI - HRMS report for **[ScF(22)].**

3 Thermodynamic Speciation

3.1 Speciation of $[ScF(mpatcn)]^-$

mpatcn (60.34 mM stock, 207 uL, 0.0125 mmol), Sc^{3+} (18.9 mM, 661.37 uL, 0.0125 mmol), KCl (0.1888 g, 2.5 mmol), and HCl (0.2600g, 1 M, 0.25 mmol) were combined and diluted to 25.0 mL total volume to create the initial stock solution at approximately pH 2 (0.5 mM **mpatcn**, 0.5 mM Sc^{3+}, 0.1 M KCl, 0.01M HCl). Aliquots of 1 mL of the stock solution were removed, and the pH was adjusted via addition of 0.1 M KOH. Solutions were allowed to fully equilibrate over 24 hours prior to analysis. ^{1}H and ^{45}Sc NMR spectra and pH measurements were recorded for each sample. Following this F^- (.526 mM, 1 µL, 0.5 nmole) was added to each sample and ^{1}H and ^{45}Sc NMR spectra and pH measurements were rerecorded. Speciation calculations were performed using the HypNMR2008 program. Speciation plots were generated 23 ^{45}Sc NMR from pH 2-12. To enable data fitting using HypNMR for species that do not rapidly equilibrate, integrations of peaks corresponding to the Q = 0, Q = $1/F^-$, and Q = OH^- were used to generate a weighted average of the ppm shift for each spectra. This weighed average was then used for data fitting with HypNMR. Logß values for ligand protonation were previously reported.[1]

Table S1 Tabulated ^{1}H and ^{45}Sc ppm shifts for **mpatcn** species discussed in this work.

Species	^{1}H ppm Shift	^{45}Sc ppm Shift
[Sc(mpatcn)]	7.69	85.10
$[Sc(mpatcn)H_2O]$	7.64	85.10
$[ScF(mpatcn)]^-$	7.64	67.91
$[Sc(mpatcn)OH]^-$	7.62	97.6

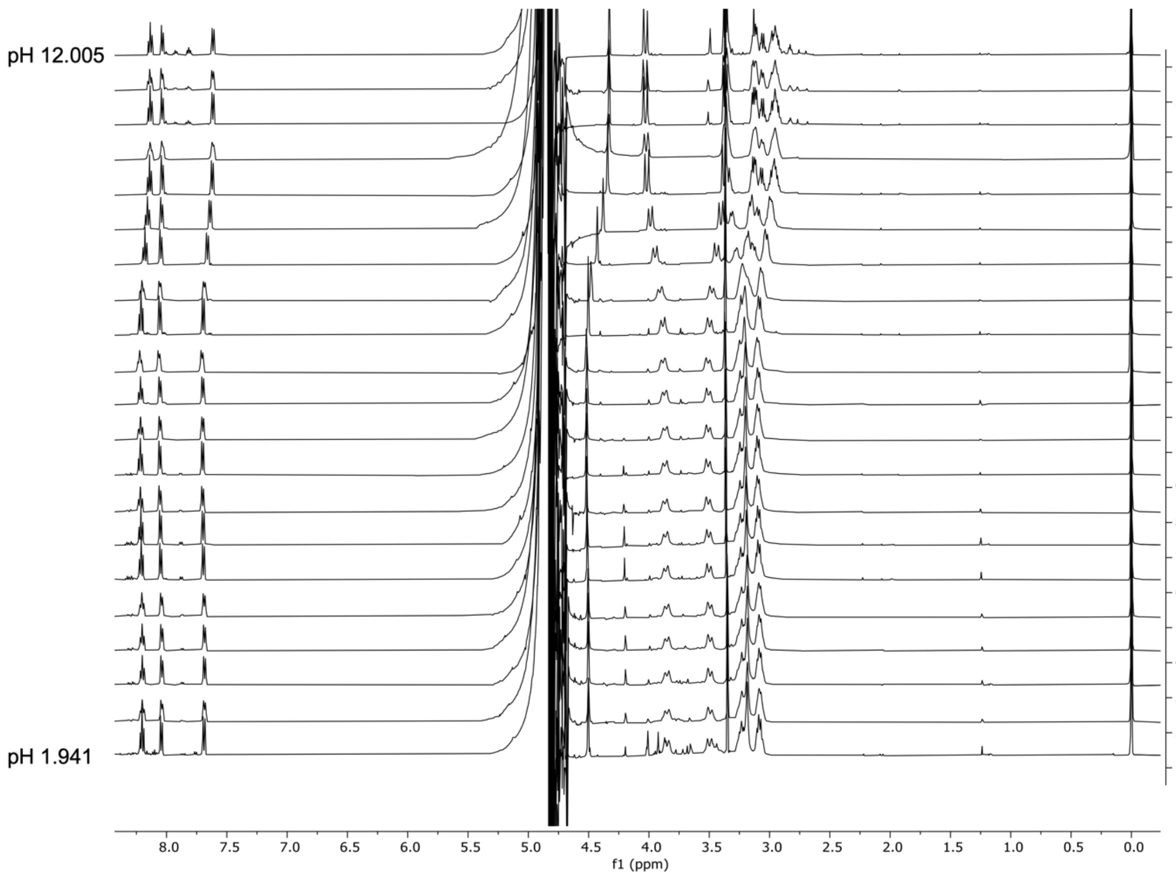

Figure S86. pH dependent ^{1}H nmr of **[Sc(mpatcn)]** that were used to calculate relevant logß values.

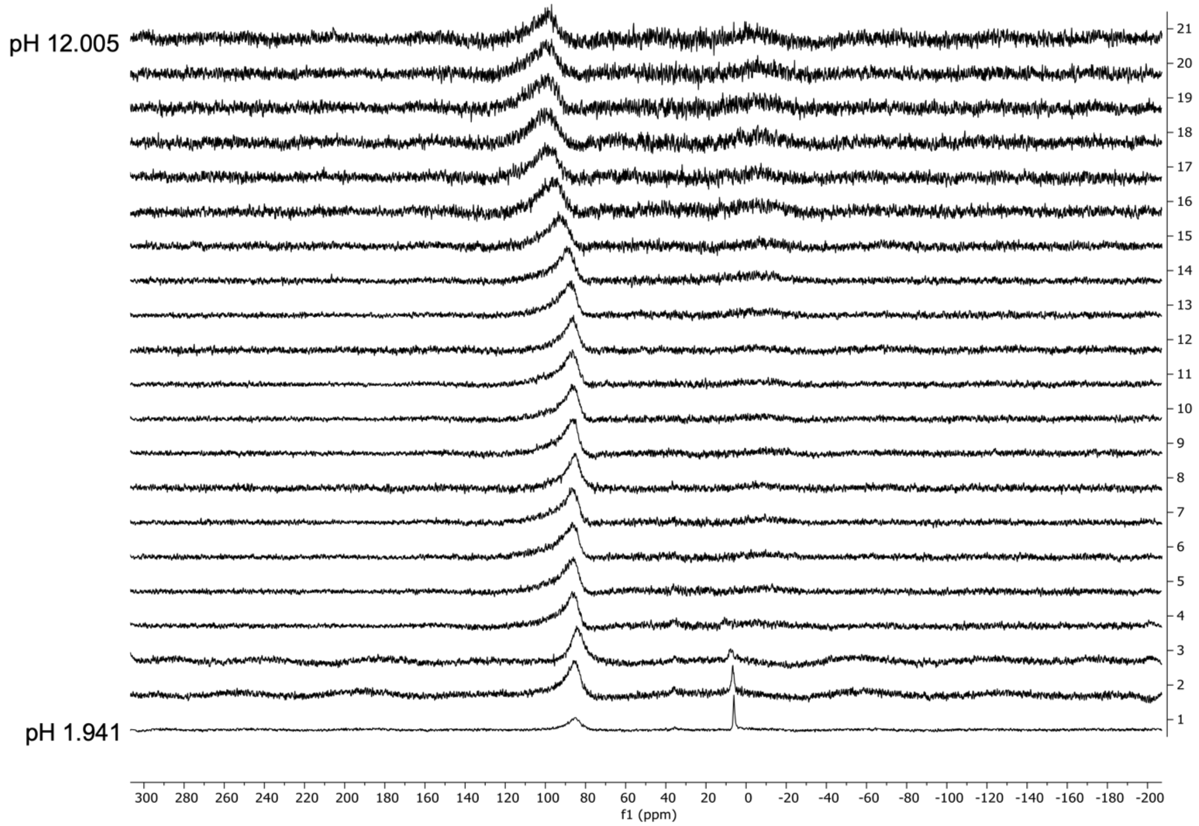

Figure S87. pH dependent ^{45}Sc nmr of [**Sc(mpatcn)]** that were used to calculate relevant logß values.

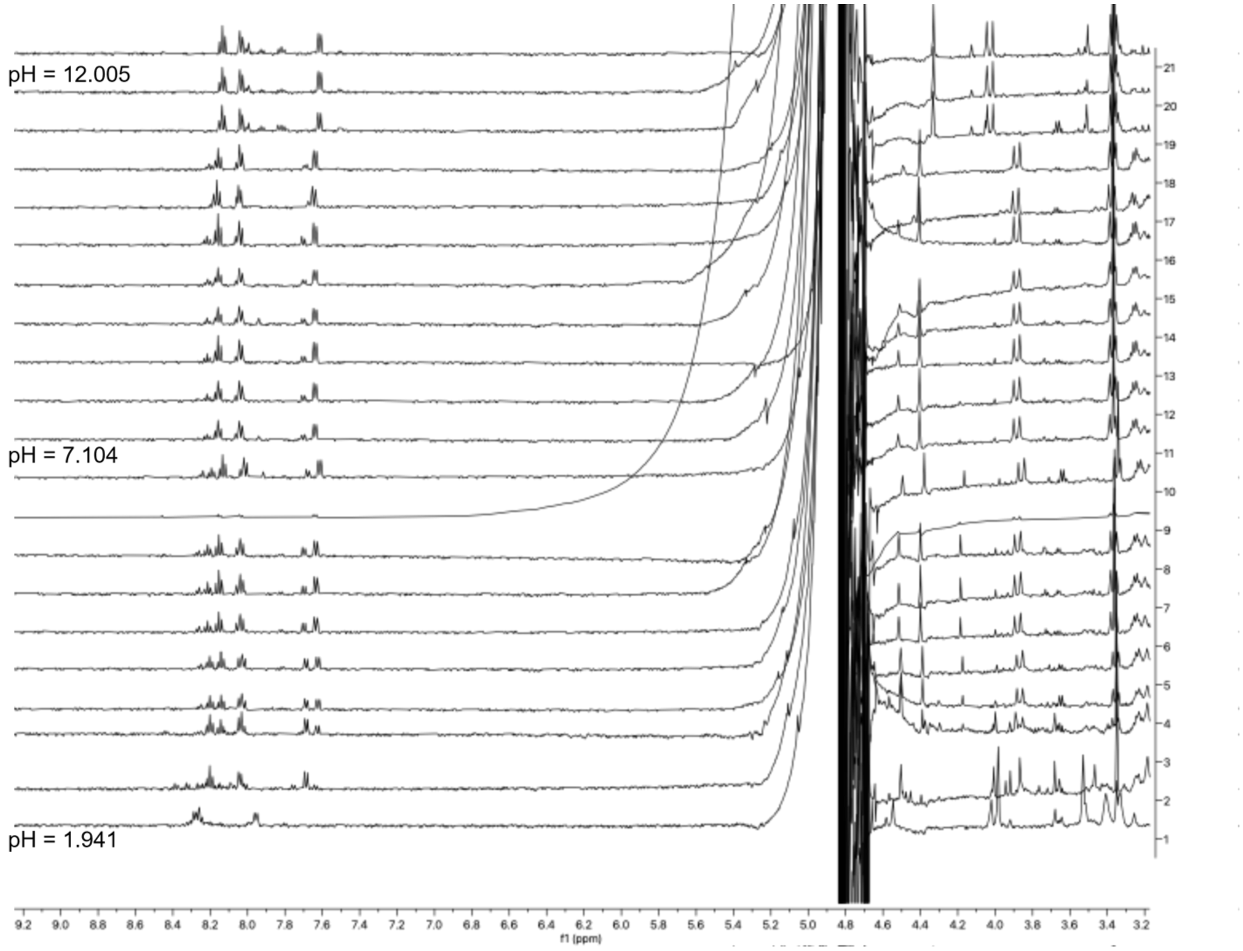

Figure S88. pH dependent ^{1}H nmr of **[ScF(mpatcn)]$^{-}$** that were used to calculate relevant logß values.

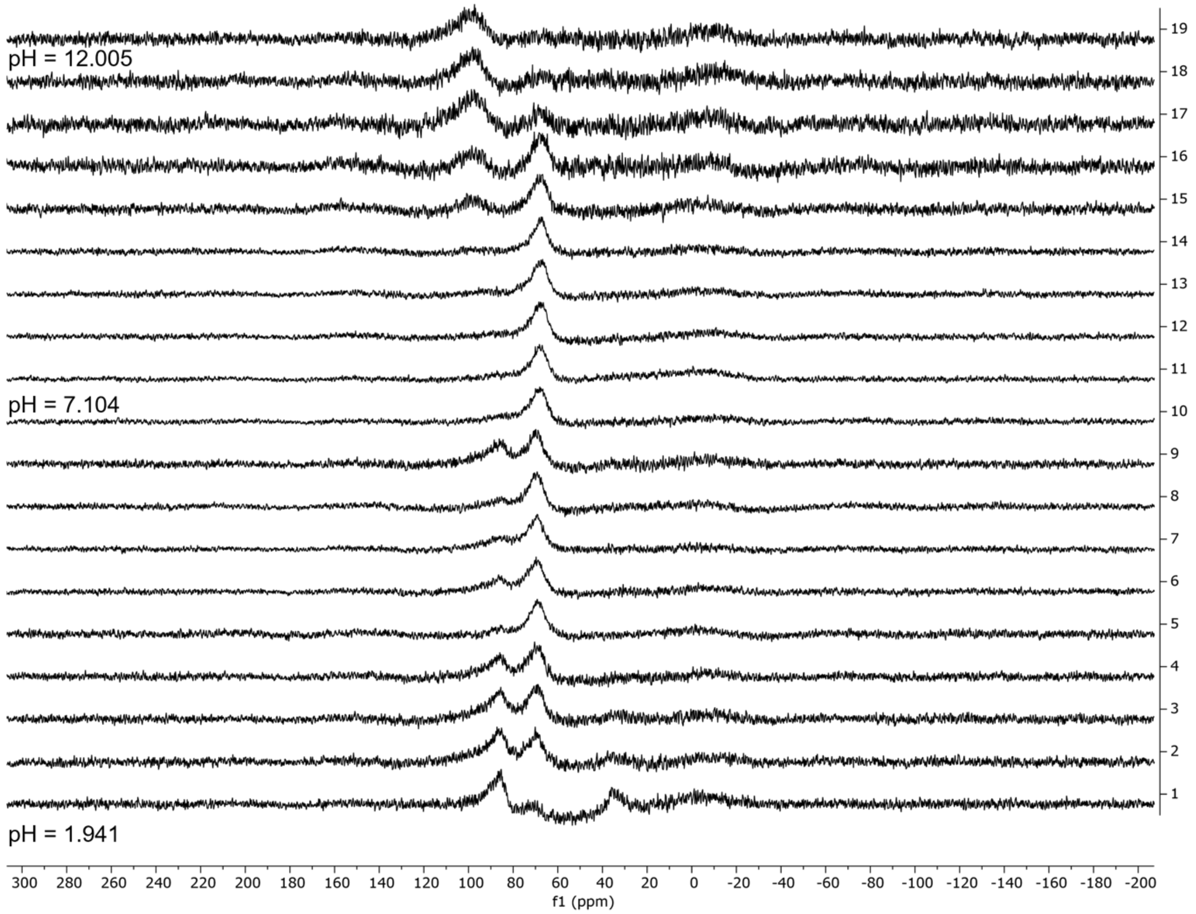

Figure S89. pH dependent ^{45}Sc nmr of **[ScF(mpatcn)]**$^{-}$ that were used to calculate relevant logß values.

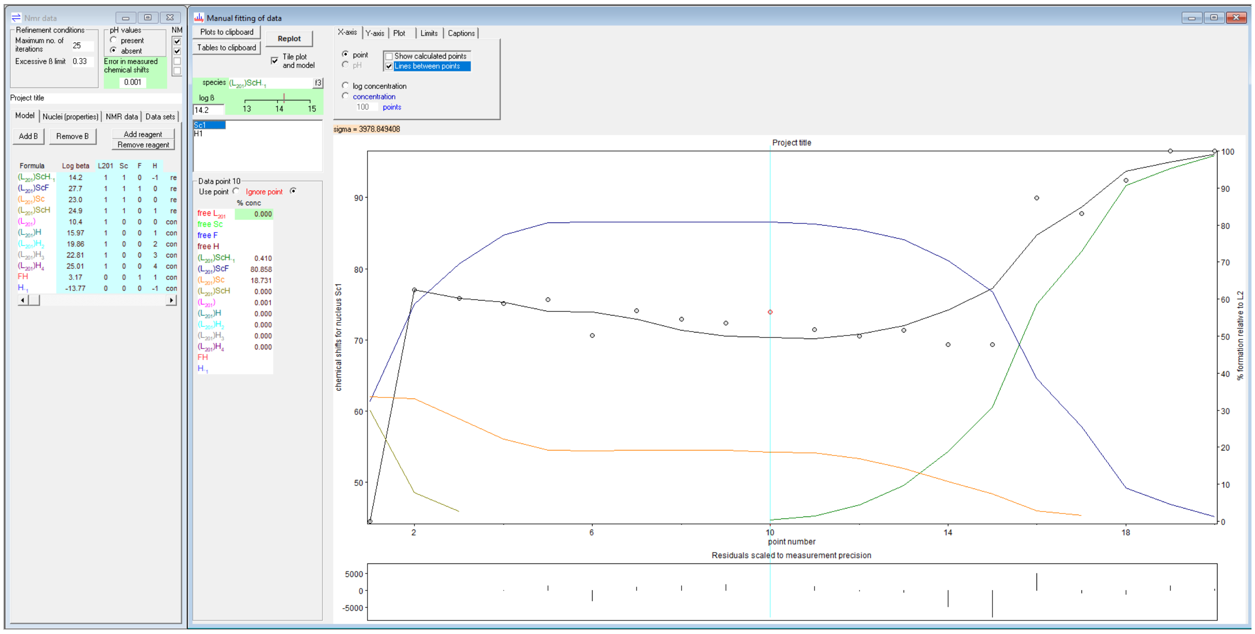

Figure S90. Representative data fit of ^{45}Sc NMR spectra of [**ScF(mpatcn)]**$^{-}$ in HYPNMR2008.

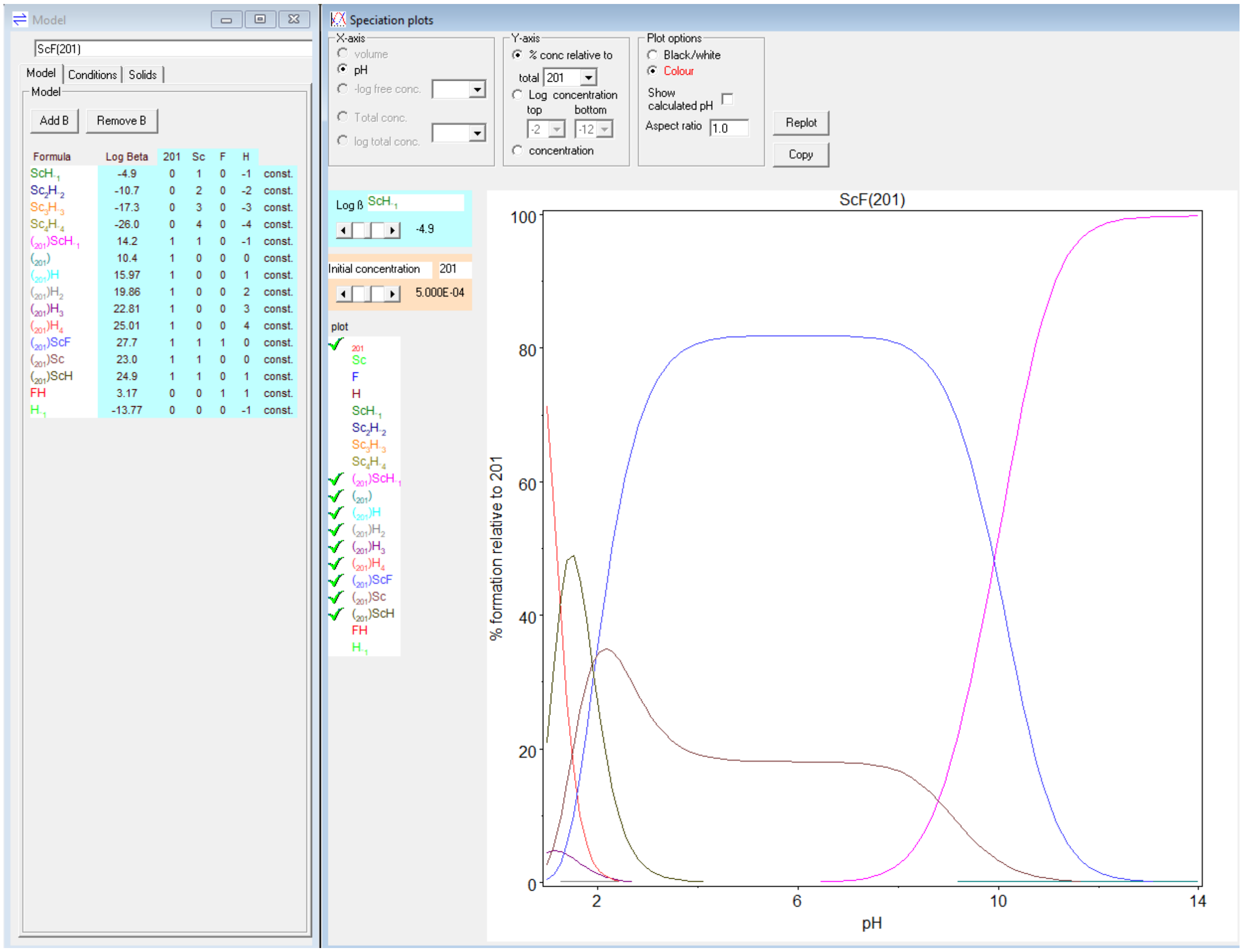

Figure S91. Data fit of [**ScF(mpatcn)**]⁻ speciation in HySS2009. Modeling concentrations are set to 0.5 mM, corresponding to the concentration at which spectra were measured.

Table S2 Tabulated Logß values **mpatcn** species discussed in this work.

Species	Logß
$[Sc(mpatcn)H]^+$	24.9
[Sc(mpatcn)]	23
$[ScF(mpatcn)]^-$	27.7
$[Sc(mpatcn)OH]^-$	14.2

3.2 Speciation of [ScF(mpatcn-am-Bz)]

mpatcn-am-Bz (8.4 mM stock, 29.7 uL, 0.0025 mmol), Sc^{3+} (37 mM, 6.7 uL, 0.0025 mmol), KCl (0.1888 g, 2.5 mmol), and HCl (0.2600g, 1 M, 0.25 mmol) were combined and diluted to 25.0 mL total volume to create the initial stock solution at approximately pH 2 (0.01 mM **mpatcn-am-Bz**, 0.01 mM Sc^{3+}, 0.1 M KCl, 0.01M HCl). Aliquots of 1 mL of the stock solution were removed, and the pH was adjusted via addition of 0.1 M KOH. Solutions were allowed to fully equilibrate over 24 hours prior to analysis. ^{1}H and ^{45}Sc NMR spectra and pH measurements were recorded for each sample. Following this F^- (.526 mM, .2 µL, 0.1 nmole) was added to each sample and ^{1}H and ^{45}Sc NMR spectra and pH measurements were rerecorded. Speciation calculations were performed using the HypNMR2008 program. Speciation plots were generated 23 ^{45}Sc NMR from pH 2-12. To enable data fitting using HypNMR for species that do not rapidly equilibrate, integrations of peaks corresponding to the Q = 0, Q = 1/F^-, and Q = OH^- were used to generate a weighted average of the ppm shift for each spectra. This weighed average was then used for data fitting with HypNMR. Logß values for ligand protonation were previously reported.[1]

Table S3 Tabulated ^{1}H ppm shifts for **mpatcn-am-Bz** species discussed in this work.

Species	^{1}H ppm Shift
[Sc(mpatcn-am-Bz)]	3.17
[Sc(mpatcn-am-Bz)H_2O]	3.15
[ScF(mpatcn-am-Bz)]$^-$	3.17
[Sc(mpatcn-am-Bz)OH]$^-$	3.14

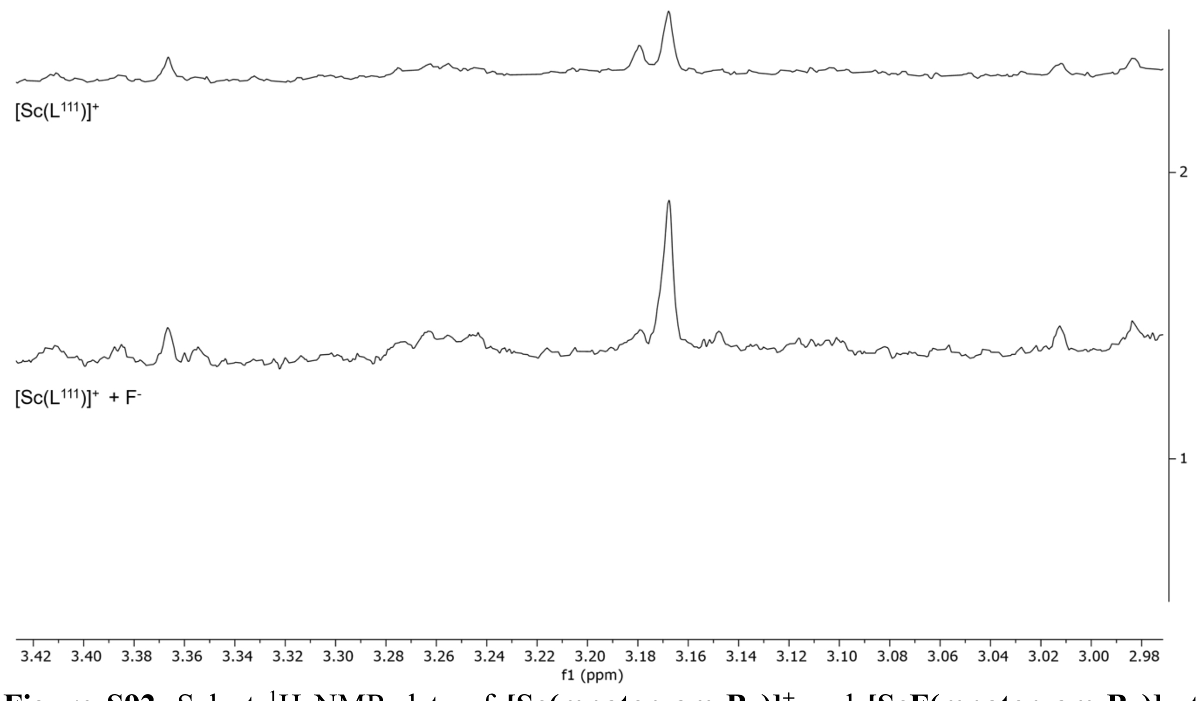

Figure S92. Select ^{1}H NMR data of **[Sc(mpatcn-am-Bz)]$^+$** and **[ScF(mpatcn-am-Bz)]** at pH 7.001. The region of the spectra containing the amide -CH_3 is highlighted to show the shift between the Sc and ScF complex.

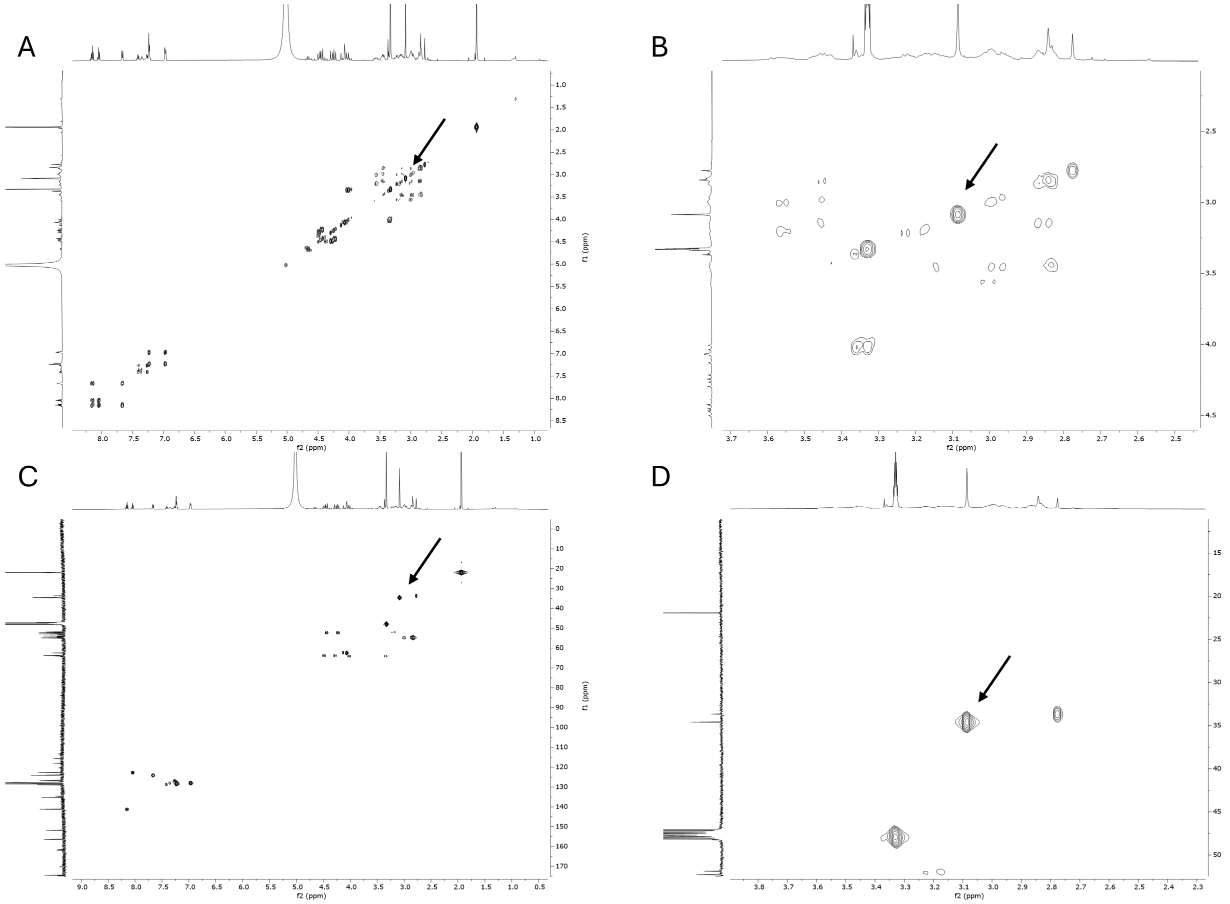

Figure S93. 2D nmr employed to confirm peak at 3.17 ppm in ^{1}H nmr spectra corresponds to the amide $-CH_3$. a) The ^{1}H-^{1}H COSY NMR spectrum of **[ScF(mpatcn-am-Bz)]**. The black arrow indicates the relevant observed correlation. b) Select region of ^{1}H-^{1}H COSY NMR spectrum corresponding to peak at 3.17 ppm. The black arrow indicates the relevant observed correlation. c) The ^{1}H-^{13}C HSQC NMR spectrum of **[ScF(mpatcn-am-Bz)]**. The black arrow indicates the relevant observed correlation. d) Select region of ^{1}H-^{13}C HSQC NMR spectrum corresponding to peak at 3.17 ppm. The black arrow indicates the relevant observed correlation.

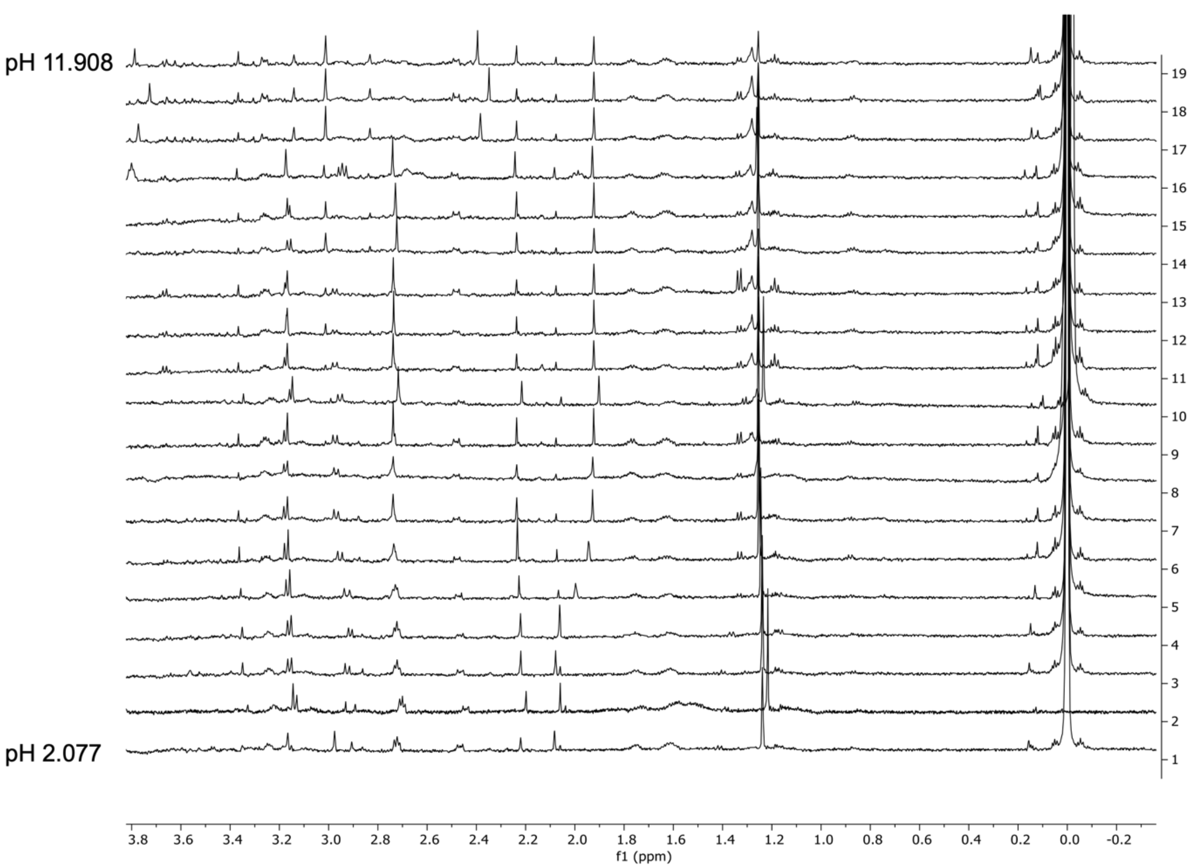

Figure S94. pH dependent ^{1}H nmr of [**Sc(mpatcn-am-Bz)**]$^{+}$ that were used to calculate relevant logß values.

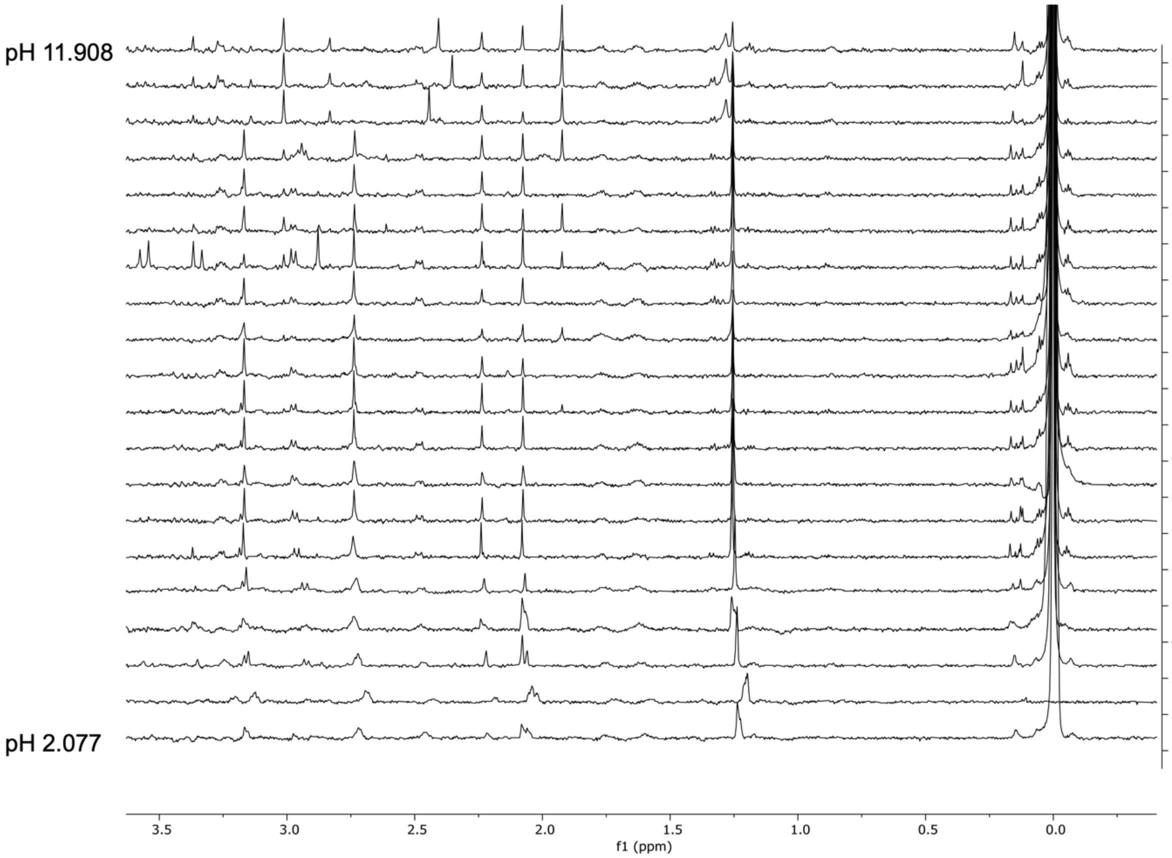

Figure S95. pH dependent ^{1}H nmr of **[ScF(mpatcn-am-Bz)]** that were used to calculate relevant logß values.

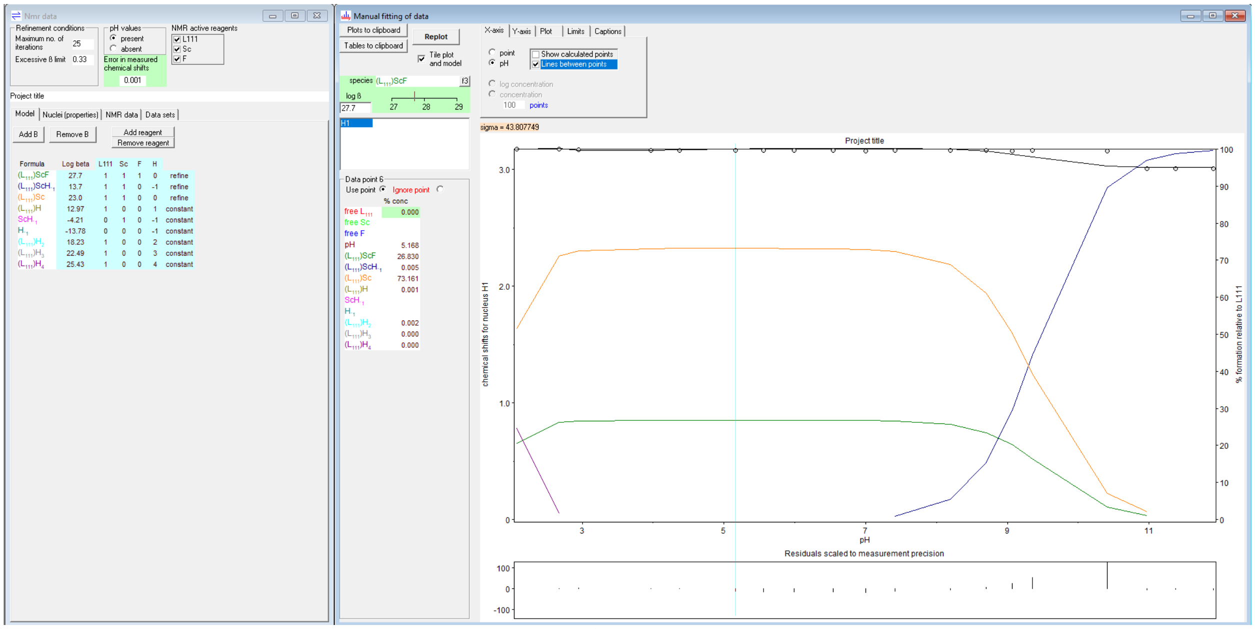

Figure S96. Representative data fit of ^{1}H NMR spectra of **[ScF(mpatcn-am-Bz)]** in HYPNMR2008.

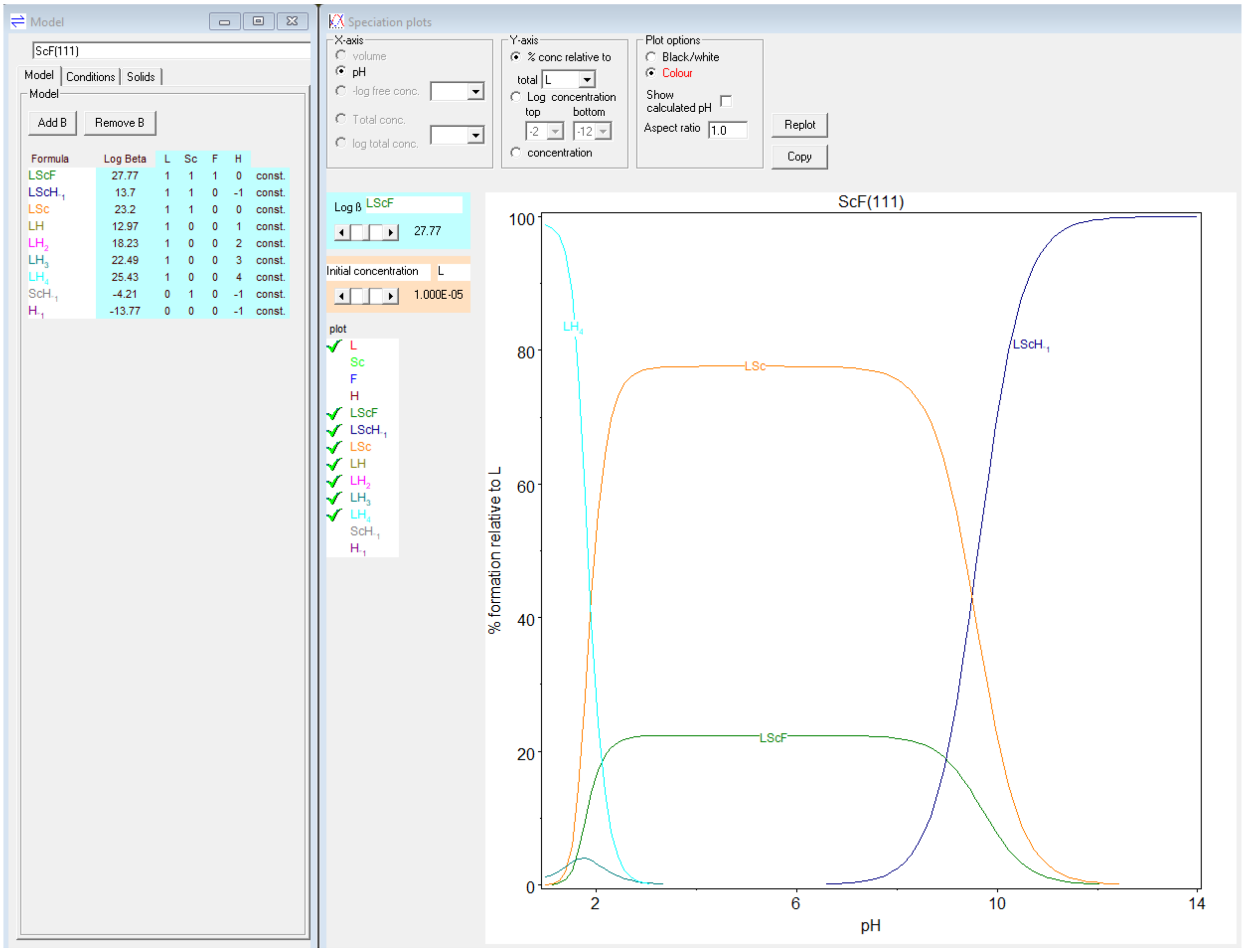

Figure S97. Data fit of **[ScF(mpatcn-am-Bz)]** speciation in HySS2009. Modeling concentrations are set to 0.01 mM, corresponding to the concentration at which spectra were measured.

Table S4 Tabulated Logß values **mpatcn-am-Bz** species discussed in this work.

Species	Logß
[Sc(mpatcn-am-Bz)]	23.2
$[ScF(mpatcn\text{-}am\text{-}Bz)]^-$	27.8
$[Sc(mpatcn\text{-}am\text{-}Bz)OH]^-$	13.7

4 Computational Chemistry

4.1 DFT Parameters

Calculations were performed using version 5.0.1 of the Orca5 software package. Computations were performed with the CAM-B3LYP functional3 with the D3(BJ) dispersion4 and the def2-TZVP5 basis set. Calculations were performed with the CPCM polarizable continuum model solvation model6–8 with water as the solvent. Geometry optimized structures as well as molecular orbitals (MOs) were modeled in PyMOL9 with all hydrogens omitted for clarity. All atom coloring is as follows: carbons are depicted as gray, oxygens as red, and nitrogens as blue. All molecular orbitals are depicted with electropositive orbitals in blue and electronegative orbitals in red with an isosurface value of 0.03. Previously modeled compounds, L^{300}, L^{030}, L^{210}, L^{120}, mpatcn, mpatcn-am-Bz, L^{021}, L2, and L3, had cone angles determined using previously published optimized structures.[1, 7] For $[Sc(AAZTA)H_2O]^-$, calculations were made using the previously published crystal structure.[8]

4.2 Bond Angle Calculations

To determine the relative openness of the water coordination site for any given ligand, the following measurements were made. XYZ coordinates of DFT optimized structures were modeled using Avogadro. Bond angles of the form ∠ABC where A is the oxygen of the inner sphere water, B is the scandium metal center, and C is a coordinate oxygen of the macrocyclic ligand were generated. All such angles were measured, and the smallest of all the angles for any given complex was used to approximate the relative openness of the water coordination site. Analysis of previously published structures indicates no significant difference in angle size between isomers, so only ΔΔΔ isomers were investigated for the set of macrocyclic ligands.

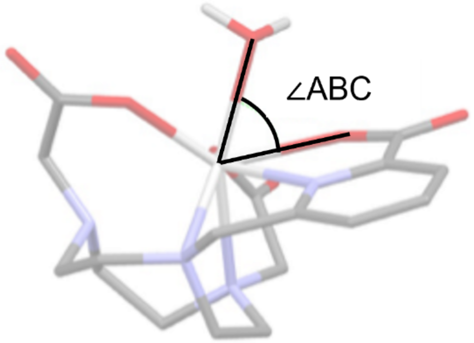

Figure S98. Representative angle measurement employed for computational analysis.

4.3 DFT Coordinates

Table S5 XYZ coordinates of $[Sc(mpatcn\text{-}am\text{-}Bz\text{-}p)H_2O]$.

Sc	0.000000	0.000000	0.000000
N	-0.451100	1.747780	1.602670
C	0.696480	2.325420	2.361460
C	2.039120	2.015020	1.716740
N	2.097100	0.577080	1.401380
C	2.195980	-0.218120	2.655820

C	1.242890	-1.398650	2.700010
N	-0.122200	-1.067160	2.219810
C	-0.852680	-0.147260	3.161060
C	-1.437060	1.106810	2.498290
H	-1.775580	1.797060	3.292470
H	-2.313480	0.849200	1.889520
H	-0.165890	0.137560	3.967340
H	-1.678990	-0.690540	3.642980
C	-0.849670	-2.350190	2.079820
C	-2.114090	-2.143260	1.307630
C	-3.292930	-2.859250	1.527310
C	-4.405830	-2.580440	0.734190
C	-4.317410	-1.580340	-0.237530
C	-3.110110	-0.905080	-0.382800
N	-2.029020	-1.197880	0.360900
C	-2.908010	0.235980	-1.344740
O	-3.830020	0.573460	-2.107050
O	-1.749510	0.784460	-1.272130
H	-5.163340	-1.307220	-0.866680
H	-5.339520	-3.124520	0.885350
H	-3.330020	-3.612360	2.314840
H	-0.205840	-3.044070	1.517330
H	-1.053620	-2.805970	3.062730
H	1.202480	-1.788730	3.733030
H	1.613340	-2.205820	2.058460
H	2.012050	0.446490	3.507050
H	3.222530	-0.597450	2.785020
C	3.194180	0.258380	0.483020
C	2.869980	-1.055580	-0.199440
O	1.649420	-1.396600	-0.260140
N	3.817220	-1.817150	-0.730130
C	5.226120	-1.431990	-0.812620
H	5.574490	-1.609800	-1.840020
H	5.364960	-0.371790	-0.583070
H	5.831580	-2.041730	-0.124760
H	4.166520	0.230860	0.997750
H	3.243260	1.029910	-0.301610
H	2.850910	2.312020	2.405980
H	2.169810	2.575340	0.785970
H	0.669260	1.937320	3.385170
H	0.574580	3.416210	2.445040
C	-1.006290	2.780810	0.711150
C	-0.118370	2.905570	-0.533380
O	-0.120010	3.944430	-1.202420
O	0.589270	1.849410	-0.815970
H	-1.099090	3.758160	1.210970

H	-2.001060	2.470120	0.365900
O	-0.035050	-0.951020	-2.106080
H	0.856460	-1.115430	-2.456220
H	-0.455580	-0.315740	-2.710690
H	3.799500	-2.689150	-0.201270

Table S6 XYZ coordinates of [Sc(picaga-OMe)H_2O].

Sc	-0.010177	-0.007273	0.016409
N	0.544996	0.919175	2.288107
C	1.927263	0.765373	2.806988
C	2.923378	0.470254	1.696441
N	2.413182	-0.644725	0.890189
C	2.437188	-1.899387	1.680229
C	1.158244	-2.711477	1.580582
N	-0.076210	-1.895399	1.674282
C	-0.269038	-1.317771	3.047217
C	-0.452840	0.206673	3.106111
H	-0.412249	0.510705	4.168794
H	-1.441856	0.484094	2.720727
H	0.586478	-1.613560	3.666681
H	-1.153726	-1.772786	3.515521
C	-1.195011	-2.791625	1.314736
C	-2.439851	-2.011721	1.029828
C	-3.727326	-2.523726	1.203191
C	-4.816068	-1.722427	0.871488
C	-4.590049	-0.422508	0.415097
C	-3.278254	0.020450	0.301583
N	-2.228971	-0.774380	0.570308
C	-2.915748	1.443687	-0.048430
O	-3.805129	2.208213	-0.472339
O	-1.697622	1.740433	0.162339
H	-5.405887	0.255357	0.172959
H	-5.832715	-2.098324	0.988916
H	-3.861705	-3.531396	1.594659
H	-0.911675	-3.331370	0.398921
H	-1.376521	-3.547246	2.097139
H	1.166377	-3.485135	2.369728
H	1.119069	-3.228441	0.617424
H	2.638420	-1.655084	2.728063
H	3.274354	-2.538515	1.354732
C	3.113677	-0.812037	-0.394734
C	2.251754	-1.677705	-1.322636
O	2.777055	-2.398865	-2.183519
O	0.980453	-1.562583	-1.145431

H	4.116563	-1.262689	-0.281284
C	3.238275	0.226614	-0.896162
H	3.910415	0.241318	2.140756
H	3.047103	1.338887	1.044050
H	1.939893	-0.038538	3.549452
H	2.233088	1.677130	3.342349
C	0.276895	2.345684	2.071200
C	0.844791	2.790058	0.718276
O	1.098827	3.983943	0.520998
O	1.001514	1.856138	-0.167778
H	0.696253	2.976604	2.870649
H	-0.803683	2.515640	2.025321
O	-0.773920	0.207039	-1.778533
H	-0.543712	1.079605	-2.132134
H	-1.142839	-0.838217	-1.642155
C	3.768134	0.086680	-1.866128
H	3.810476	0.870953	-0.260113
H	2.274082	0.665907	-1.068628
H	4.733065	-0.351233	-1.693384
C	3.891391	1.074012	-2.351261
H	3.195770	-0.556548	-2.503673
O	4.476952	1.730045	-1.699536
O	4.410628	0.945271	-3.304786
C	4.470508	2.950542	-2.038638
H	5.066080	3.514173	-1.347586
H	3.464313	3.323481	-2.027820
H	4.877678	3.054006	-3.025904

4.4 Bond Angle VS %RCY

Radiolabeling was conducted following a literature procedure.[6] To an aqueous solution of ammonium acetate (50 µL, 1 M, pH 4.8) was added an aliquot of unprocessed $[^{18}F]F^-$ stock (110-143 µL, ~1 mCi) followed by an aliquot of a $ScCl_3 \cdot 6H_2O$ stock solution (2-20 µL, 20 nmol) of known concentration as determined by ICP-OES or MP-AES. Following incubation at room temperature for 10 minutes, an aliquot of a ligand stock solution (5-20 µL, 100 nmol), of known concentration as determined UV-vis spectroscopy, was added. Total reaction volume = 200 µL. The mixtures were incubated at 80 °C for 30 min prior to radioHPLC analysis (Method B). % RCY was determined via integration of radioHPLC spectra.

Table S7 Tabulated bond angles and %RCY for ligands discussed in this work.

Ligand	Most Restrictive Angle	% RCY
L^{300}	75.6	0
L^{030}	75.1	0
L^{210}	72.1	7.9
L^{120}	77.1	14.1
mpatcn	67.4	33.3
picaga	68.2	52.65
mpatcn-am-Bz	68.7	36.7
L^{021}	69.1	19
mpatcn-am-Bz-p	68.5	0
L2	51	0
L3	57.2	66
AAZTA	74.5	0

5 Radiolabeling Data

5.1 General ^{18}F Radiolabeling Procedure

Radiolabeling was conducted following a literature procedure.[6] To an aqueous solution of ammonium acetate (50 µL, 1 M, pH 4.8) was added an aliquot of unprocessed [^{18}F]F^- stock (110-143 µL, ~1 mCi) followed by an aliquot of a $ScCl_3 \cdot 6H_2O$ stock solution (2-20 µL, 20 nmol) of known concentration as determined by ICP-OES or MP-AES. Following incubation at room temperature for 10 minutes, an aliquot of a ligand stock solution (5-20 µL, 100 nmol), of known concentration as determined UV-vis spectroscopy, was added. Total reaction volume = 200 µL. All radiolabeling were performed at 0.01 mCi/nmol. The mixtures were incubated at 80 °C for 30 min prior to radioHPLC analysis (Method B). The same procedure was used for animal studies. Following purification of the labeled compounds for animal studies, injection solutions were prepared with a decay corrected specific activity of 0.03 mCi/nmol.

5.2 General ^{44}Sc/^{177}Lu Radiolabeling Procedure

Radiolabeling was conducted following a literature procedure.[6] To an aqueous solution of ammonium acetate (10 µL, 1 M, pH 4.8) was added ligand stock solution (16-54 µL, 10 nmol), of known concentration as determined UV-vis spectroscopy, followed by an aliquot of the [^{44}Sc]$ScCl_3$ or [^{177}Lu]$LuCl_3$ stock (36-74 µL, ~1 mCi). Total reaction volume = 200 µL. All radiolabelings were performed at a specific activity of 0.1 mCi/nmol. The mixtures were incubated at 80 °C for 30 min prior to radioHPLC analysis (^{45}Sc: Method C; ^{177}Lu: Method C). The same procedure was used for animal studies.

5.3 Acid Washing of Glassware for ^{18}F Studies

Scintillation vials used to prepare stock buffer and Sc^{3+} solutions for ^{18}F radiolabeling were soaked for 24 hours in a solution of 5% HNO_3. Following this, the vials were rinsed with acetone and heated in an oven until dry. All radiolabeling was then conducted using the conditions outline in section **5.1**.

5.4 ^{18}F In Vivo Biodistribution

Following the general radiolabeling procedures outlined in section **5.1**, labeled complexes were purified via radioHPLC (Method B). Excess solvent was removed under a stream of N_2 before the ^{18}F-Sc complex was resuspended in 500 uL of 1X PBS. Following purification of the labeled compounds for animal studies, injection solutions were prepared with a decay corrected specific activity of 0.03 mCi/nmol. Doses consisted of 25-75 µCi in 100-150 uL of total volume. PET/CT imaging was performed 90 minutes p.i. and biodistribution and metabolite analysis was performed 2 hours p.i.

5.5 ^{44}Sc/^{177}Lu In Vivo Biodistribution

Following the general radiolabeling procedures outlined in section **5.2**, labeled complexes were formulated by adding 300 uL of 1X PBS to the crude radiolabeling solution. In the case where the crude labeling solution had a radiochemical purity of less than 95%, the complex was purified via radioHPLC (Method C), excess solvent was removed under a stream of N_2, and the ^{44}Sc/^{177}Lu complexes were resuspended in 500 uL of 1X PBS. Doses consisted of 100-200 µCi or 200-400 µCi, for ^{44}Sc and ^{177}Lu respectively, in 100-150 µL of total volume. Doses were formulated with

a specific activity of 0.1 mCi/nmol. PET/CT and SPECT/CT imaging was performed 90 minutes p.i. and biodistribution and metabolite analysis was performed 2 hours p.i.

5.6 Metabolite Analysis

Metabolite analysis was performed by analyzing 100 µL of mouse urine collected during biodistribution studies via radioHPLC. In cases where less than 100 µL of urine was collected, the urine was diluted with 1X PBS to a total volume of 100 µL. In cases where the total activity was below the sensitivity of the radio detector, fractions were collected every 30 seconds, activity in each fraction was quantified using a gamma counter, and the trace was reconstructed.

5.7 PET/CT

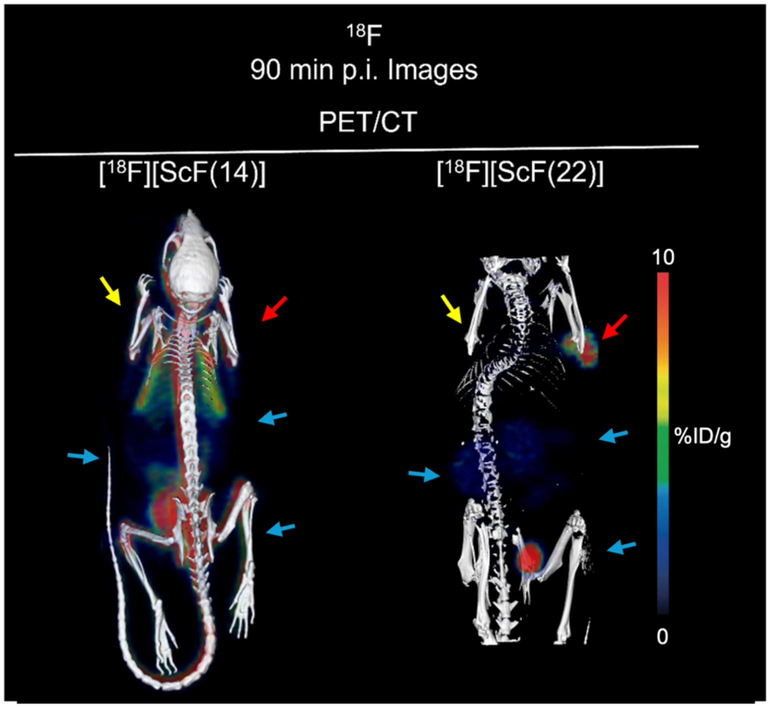

Figure S99. Representative PET/CT of **[^{18}F][ScF(14)]**, and [**^{18}F][ScF (22)]**. Imaging performed 90 minutes p.i.. PSMA expressing tumors are indicated by a red arrow, PSMA non-expressing tumors are indicated by a yellow arrow.

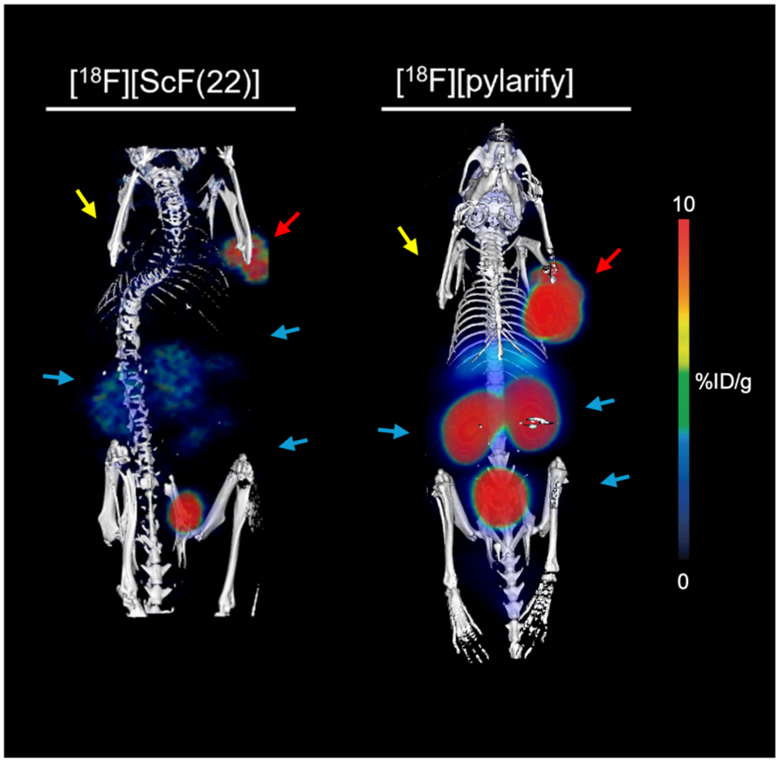

Figure S100. Representative PET/CT of [^{18}**F][ScF(22)]** and Pylarify. Imaging performed 90 minutes p.i.. PSMA expressing tumors are indicated by a red arrow, PSMA non-expressing tumors are indicated by a yellow arrow. Renal clearance pathways, kidneys and bladder, are indicated by a blue arrow.

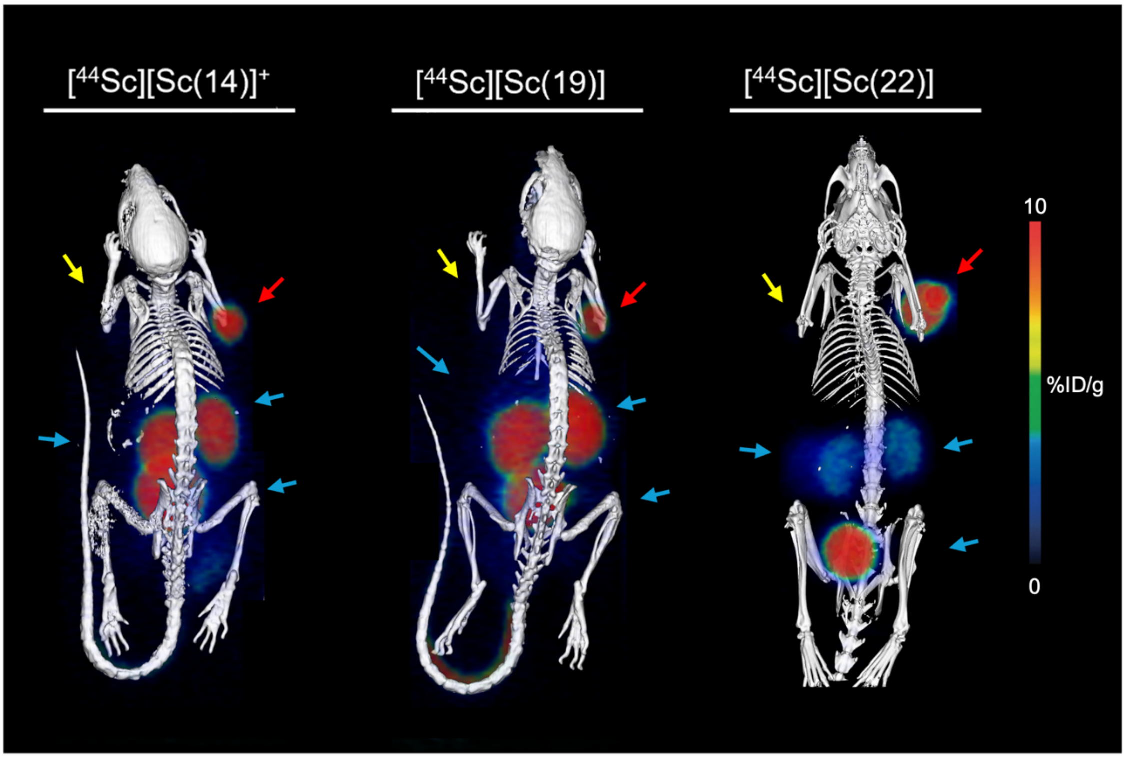

Figure S101. Representative PET/CT of **[^{44}Sc][Sc(14)]**$^{+}$, **[^{44}Sc][Sc(19)]**, and **[^{44}Sc][Sc(22)]**. Imaging performed 90 minutes p.i.. PSMA expressing tumors are indicated by a red arrow, PSMA non-expressing tumors are indicated by a yellow arrow. Renal clearance pathways, kidneys and bladder, are indicated by a blue arrow.

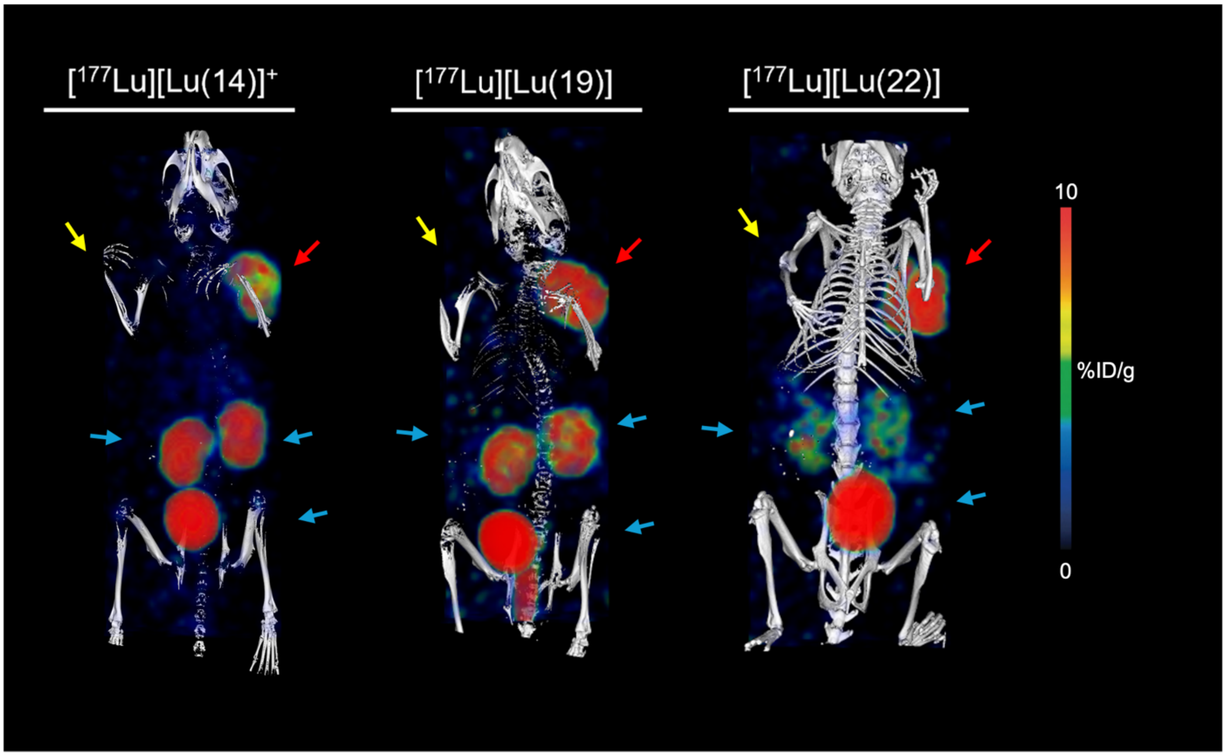

Figure S102. Representative PET/CT of **[^{177}Lu][Lu(14)]$^{+}$, [^{177}Lu][Lu(19)]**, and **[^{177}Lu][Lu(22)]**. Imaging performed 90 minutes p.i.. PSMA expressing tumors are indicated by a red arrow, PSMA non-expressing tumors are indicated by a yellow arrow. Renal clearance pathways, kidneys and bladder, are indicated by a blue arrow.

5.8 Tabulated Biodistribution Data

Table S8. Biodistributions of [**^{18}F][ScF(14)]**, and **[^{18}F][ScF(22)]$^{-}$** HEPES as measured by % ID/g in nude mice (n = 4) at 2 hour post-injection (mean % ID/g ± standard deviation). Specific activities of injected compounds were 0.03 mCi/nmol for **[^{18}F][ScF(22)]$^{-}$**. Due to formulation instability an exact specific activity of [**^{18}F][ScF(14)]** at the time of injection was not able to be determined.

	**[^{18}F][ScF(14)]**	**[^{18}F][ScF(22)]$^{-}$** (n = 6)
Blood	0.78 ± 0.25	0.07 ± 0.04
Heart	1.46 ± 0.46	0.06 ± 0.04
Lungs	1.15 ± 0.30	0.08 ± 0.03
Liver	0.74 ± 0.18	0.14 ± 0.08
Spleen	0.99 ± 0.18	0.17 ± 0.06
Kidneys	3.18 ± 1.04	1.36 ± 0.58
Stomach	0.68 ± 0.22	0.03 ± 0.01
Small Intestine	0.98 ± 0.69	0.11 ± 0.08
Large Intestine	10.02 ± 8.60	0.25 ± 0.33
Muscle	1.33 ± 0.55	0.04 ± 0.01
Bone	48.92 ± 27.11	0.41 ± 0.14
Brain	0.70 ± 0.25	0.02 ± 0.01
PSMA +	2.99 ± 0.34	4.20 ± 0.92
PSMA -	2.89 ± 3.02	0.22 ± 0.13
Tail	27.70 ± 11.74	0.37 ± 0.47

Table S9. Biodistributions of **[^{44}Sc][Sc(14)]$^{+}$**, **[^{44}Sc][Sc(19)]**, and **[^{44}Sc][Sc(22)]** as measured by % ID/g in nude mice (n = 4) at 2 hour post-injection (mean % ID/g ± standard deviation). Specific activities of injected compounds were 0.1 mCi/nmol.

	**[^{44}Sc][Sc(14)]**	**[^{44}Sc][Sc(19)]**	**[^{44}Sc][Sc(22)]**
Blood	0.17 ± 0.05	0.70 ± 0.19	0.12 ± 0.03
Heart	0.10 ± 0.03	0.37 ± 0.04	0.07 ± 0.02
Lungs	0.24 ± 0.09	0.76 ± 0.05	0.20 ± 0.04
Liver	0.13 ± 0.03	0.45 ± 0.16	0.17 ± 0.06
Spleen	0.92 ± 0.20	2.04 ± 0.79	0.48 ± 0.27
Kidneys	17.25 ± 4.66	54.87 ± 12.71	4.99 ± 0.78
Stomach	0.11 ± 0.03	0.28 ± 0.03	0.77 ± 0.69
Small Intestine	0.17 ± 0.15	0.24 ± 0.41	0.41 ± 0.14
Large Intestine	0.26 ± 0.32	0.58 ± 0.02	0.62 ± 0.64
Muscle	0.19 ± 0.15	0.20 ± 1.44	0.38 ± 0.56
Bone	0.05 ± 0.02	0.45 ± 0.18	0.48 ± 0.28
Brain	0.02 ± 0.02	0.23 ± 0.20	0.07 ± 0.04
PSMA +	7.28 ± 0.65	10.22 ± 4.76	16.20 ± 2.82
PSMA -	0.28 ± 0.10	0.36 ± 0.11	0.44 ± 0.13
Tail	0.32 ± 0.22	1.23 ± 1.40	0.22 ± 0.09

Table S10. Biodistributions of **[^{177}Lu][Lu(14)]$^+$, [^{177}Lu][Lu(19)]**, and **[^{177}Lu][Lu(22** as measured by % ID/g in nude mice (n = 4) at 2 hour post-injection (mean % ID/g ± standard deviation). Specific activities of injected compounds were 0.1 mCi/nmol.

	**[^{177}Lu][Lu(14)]$^+$**	**[^{177}Lu][Lu(19)]**	**[^{177}Lu][Lu(22)]**
Blood	0.07 ± 0.01	0.21 ± 0.014	0.10 ± 0.04
Heart	0.07 ± 0.04	0.15 ± 0.08	0.06 ± 0.01
Lungs	0.14 ± 0.05	0.18 ± 0.15	0.30 ± 0.15
Liver	0.09 ± 0.01	0.34 ± 0.07	0.26 ± 0.22
Spleen	0.56 ± 0.09	0.95 ± 0.74	0.60 ± 0.32
Kidneys	10.88 ± 1.47	16.79 ± 7.31	6.66 ± 1.34
Stomach	0.04 ± 0.01	0.45 ± 0.35	0.35 ± 0.24
Small Intestine	0.07 ± 0.02	0.33 ± 0.46	1.27 ± 1.36
Large Intestine	0.32 ± 0.29	0.44 ± 0.14	0.49 ± 0.72
Muscle	0.05 ± 0.03	0.79 ± 1.44	0.13 ± 0.12
Bone	0.09 ± 0.02	0.50 ± 0.72	0.09 ± 0.05
Brain	0.02 ± 0	0.06 ± 0.02	0.02 ± 0.01
PSMA +	9.11 ± 1.88	11.58 ± 5.25	17.27 ± 2.94
PSMA -	0.19 ± 0.04	0.20 ± 0.15	0.49 ± 0.11
Tail	0.24 ± 0.06	1.92 ± 1.25	0.31 ± 0.13

Table S11. Biodistributions of **Pluvicto, Illucix,** and **Pylarify** as measured by % ID/g in nude mice at 2 hour post-injection (mean % ID/g ± standard deviation). Pluvicto data is reproduced from literature.[5] Illucix data is reproduced from literature.[9] Pylarify data is reproduced from literature.[10]

	**Pluvicto**	**Illucix**	**Pylarify**
Blood	0.95 ± 0.46	0.12 ± 0.03	0.10 ± 0.04
Heart	0.27 ± 0.11	0.07 ± 0.02	0.06 ± 0.01
Liver	0.29 ± 0.12	0.17 ± 0.06	0.26 ± 0.22
Spleen	0.27 ± 0.12	0.48 ± 0.27	0.60 ± 0.32
Kidneys	5.12 ± 1.69	4.99 ± 0.78	6.66 ± 1.34
Small Intestine	0.28 ± 0.13	0.41 ± 0.14	1.27 ± 1.36
Muscle	0.14 ± 0.05	0.38 ± 0.56	0.13 ± 0.12
Bone	0.21 ± 0.09	0.48 ± 0.28	0.09 ± 0.05
PSMA +	11.78 ± 1.27	16.20 ± 2.82	17.27 ± 2.94
PSMA -	0.48 ± 0.18	0.44 ± 0.13	0.49 ± 0.11

5.9 Tumor Blood Ratio Calculations

Tumor to blood ratio (TBR) calculations were performed using the following equation.

Equation S1: $\mathrm{TBR} = \frac{\%\mathrm{ID/g_{tumor}}}{\%\mathrm{ID/g_{blood}}}$

Error propagation (δTBR) calculations were performed using the following equation.

Equation S2: $\delta\mathrm{TBR} = \mathrm{TBR} * \sqrt{(\frac{\%\mathrm{ID/g_{tumor}}}{\delta\%\mathrm{ID/g_{tumor}}})^2 + (\frac{\%\mathrm{ID/g_{blood}}}{\delta\%\mathrm{ID/g_{blood}}})^2}$

Data used to calculate these values is shown in section **5.8**.

5.10 HPLC Chromatographs

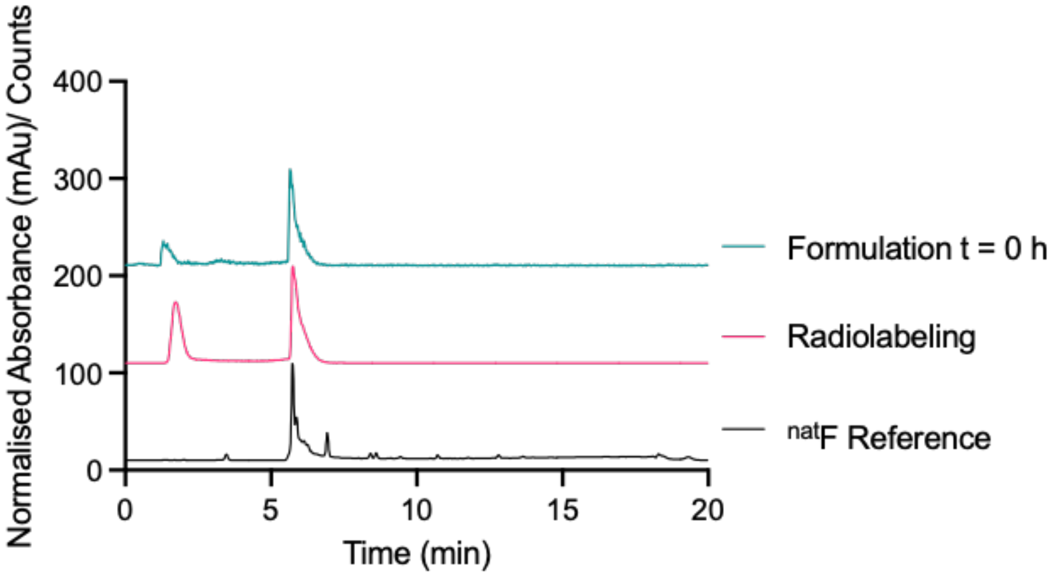

Figure S103. The RP-HPLC (Method B) analyses of **[natF][ScF(14)]**, **[18F][ScF(14)]** radiolabeling solution, and formulation at t = 0h.

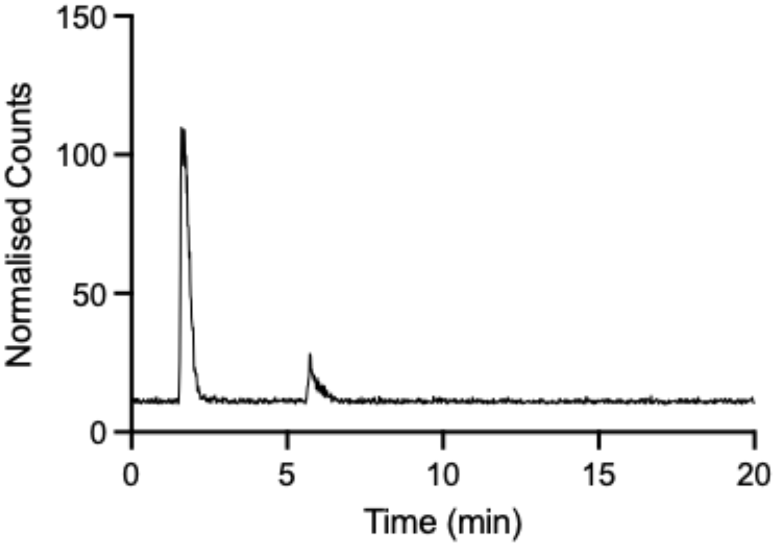

Figure S104. The RP-HPLC (Method B) analyses of **[18F][ScF(14)]** metabolites collected 2 h p.i..

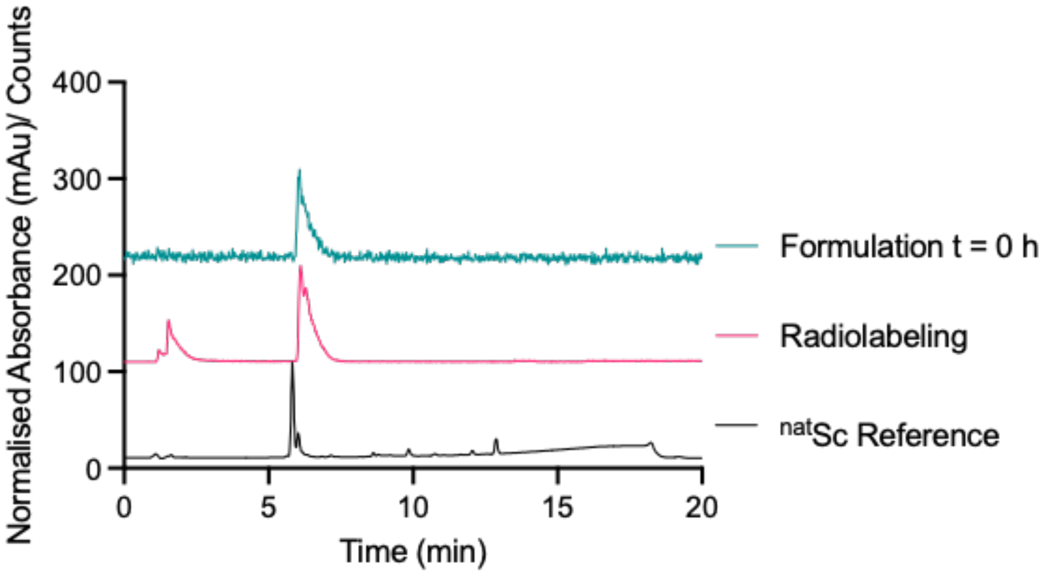

Figure S105. The RP-HPLC (Method C) analyses of **[^{nat}Sc][Sc(14)]$^{+}$**, **[^{44}Sc][Sc(14)]$^{+}$** radiolabeling solution, and formulation at t = 0h.

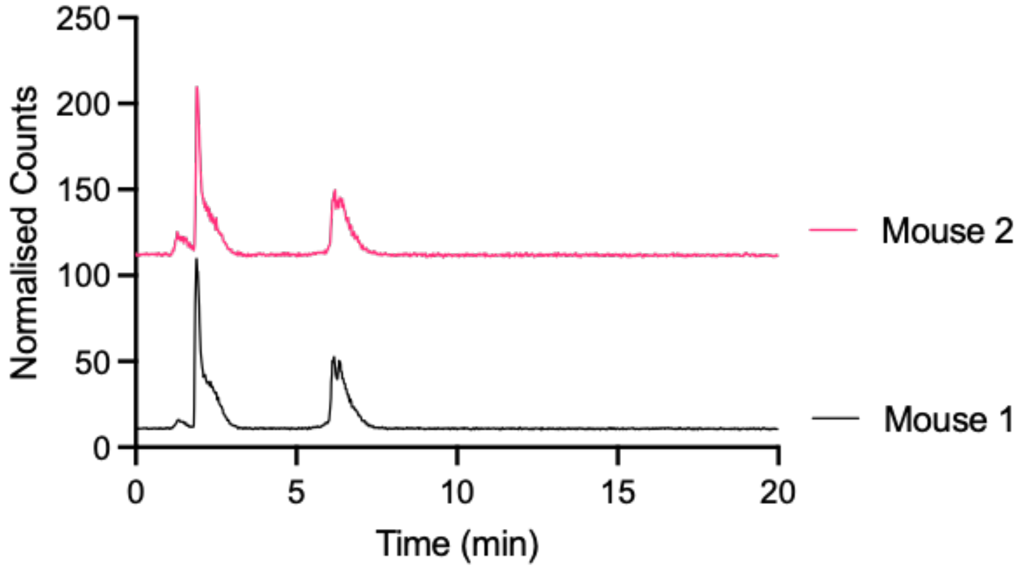

Figure S106. The RP-HPLC (Method C) analyses **[^{44}Sc][Sc(14)]$^{+}$** metabolites collected 2 h p.i..

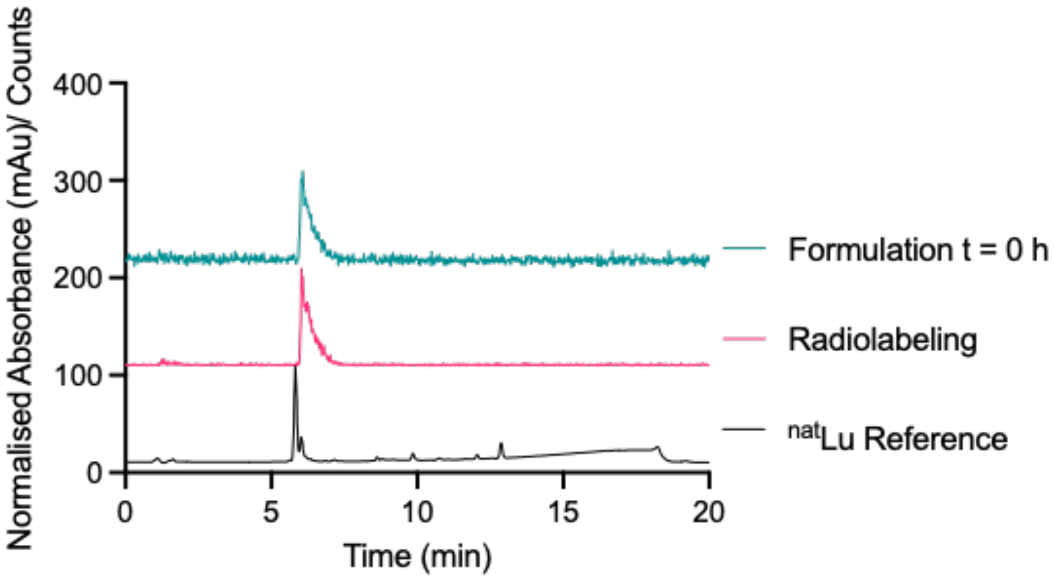

Figure S107. The RP-HPLC (Method C) analyses of **[^{nat}Lu][Lu(14)]$^+$**, **[^{177}Lu][Lu(14)]$^+$** radiolabeling solution, formulation at t = 0h.

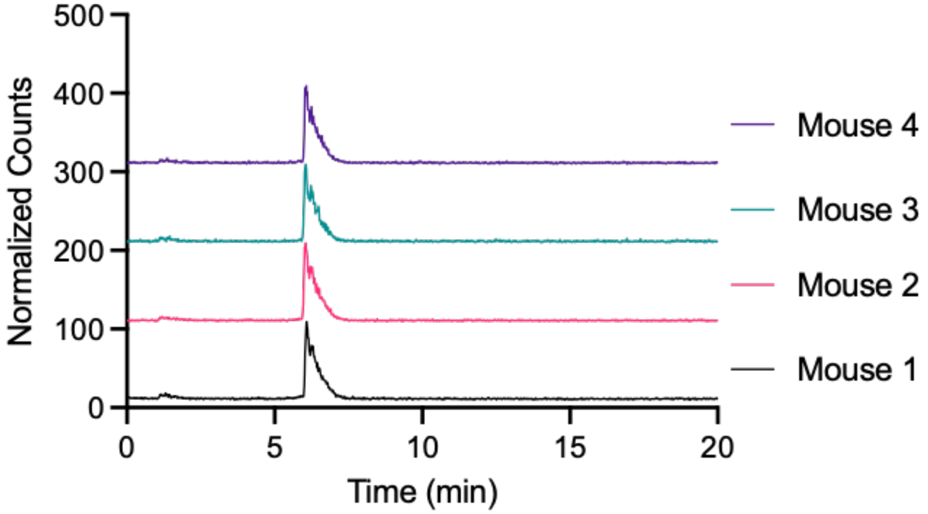

Figure S108. The RP-HPLC (Method C) analyses **[^{177}Lu][Lu(14)]$^+$** metabolites collected 2 h p.i..

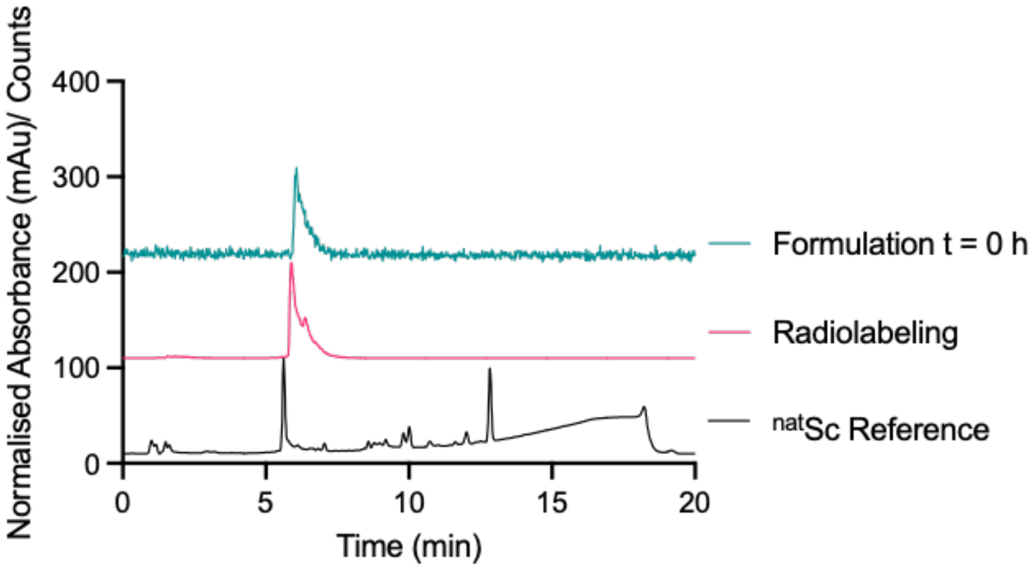

Figure S109. The RP-HPLC (Method C) analyses of **[^{nat}Sc][Sc(19)]**, **[^{44}Sc][Sc(19)]** radiolabeling solution, and formulation at t = 0h.

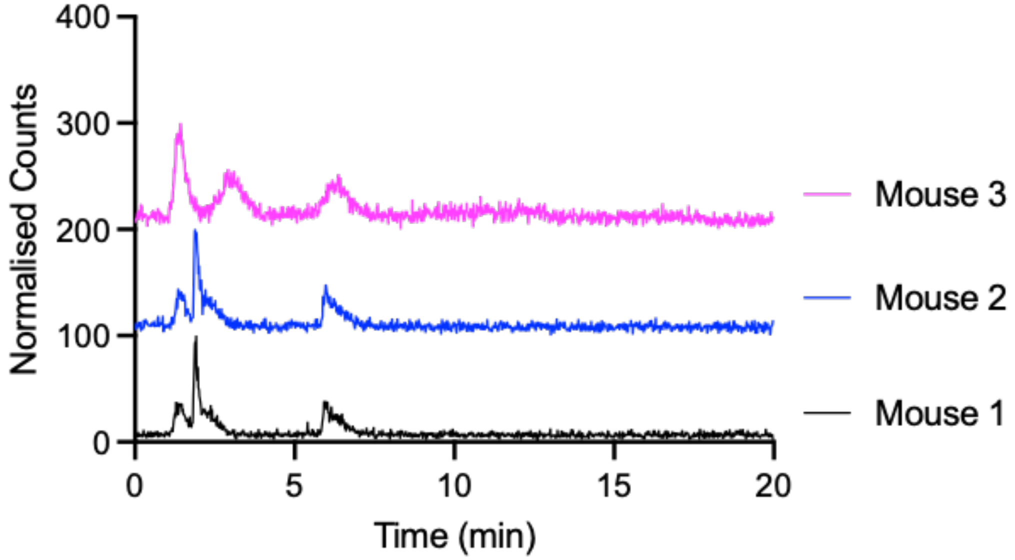

Figure S110. The RP-HPLC (Method C) analyses **[^{44}Sc][Sc(19)]** metabolites collected 2 h p.i..

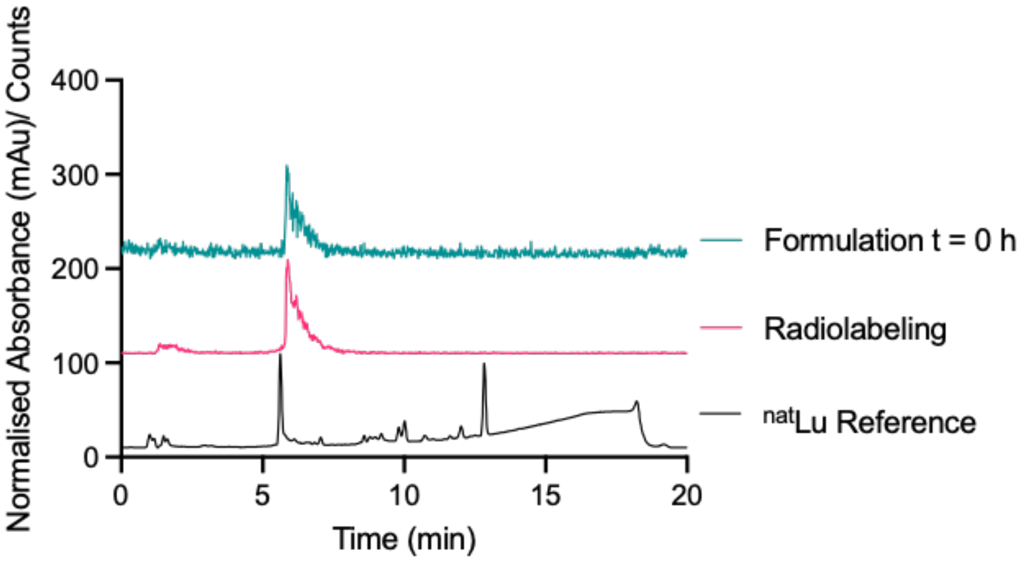

Figure S111. The RP-HPLC (Method C) analyses of **[natLu][Lu(19)]**, **[^{177}Lu][Lu(19)]** radiolabeling solution, formulation at t = 0h.

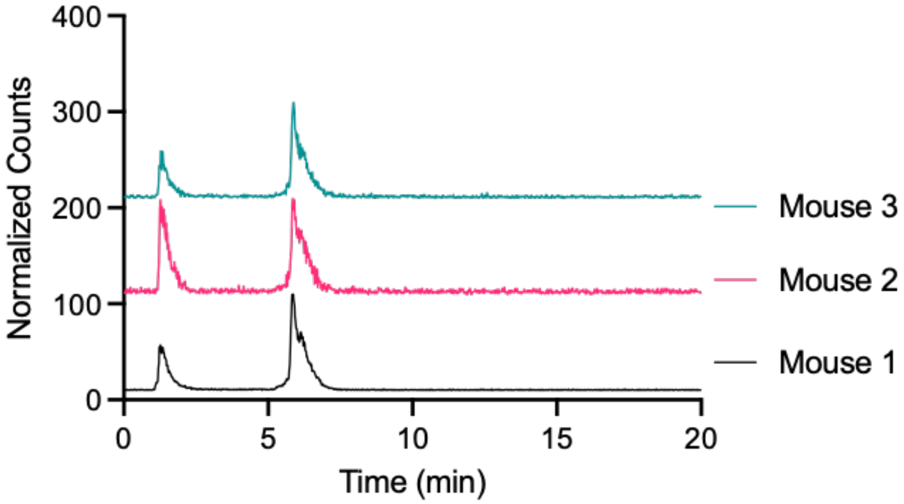

Figure S112. The RP-HPLC (Method C) analyses **[^{177}Lu][Lu(19)]** metabolites collected 2 h p.i..

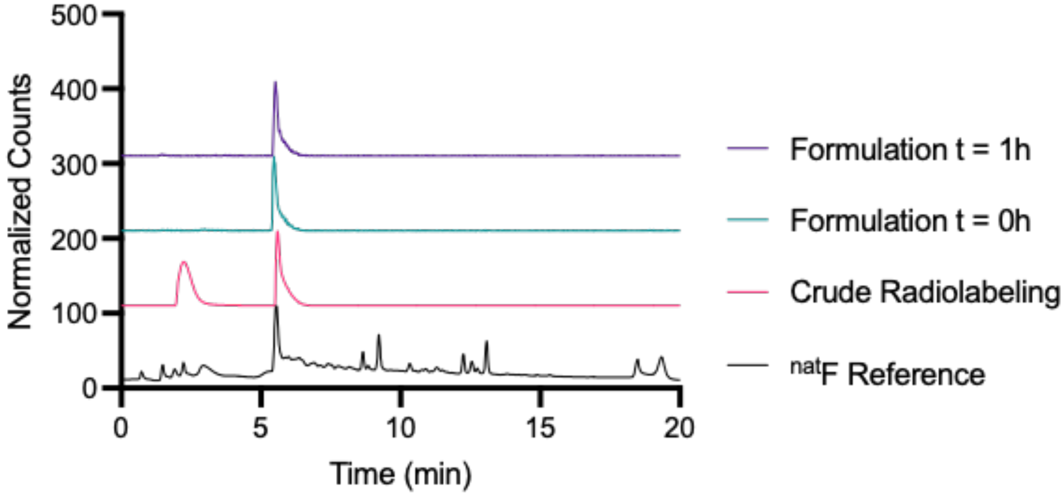

Figure S113. The RP-HPLC (Method B) analyses of **[^{nat}F][ScF(22)]$^{-}$**, **[^{18}F][ScF(22)]$^{-}$** radiolabeling solution, and formulation at t = 0, and 1h.

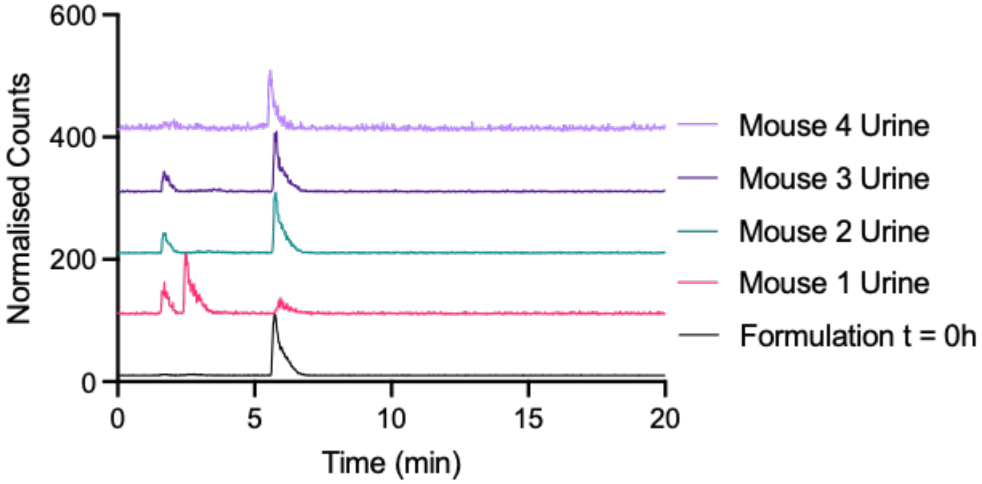

Figure S114. The RP-HPLC (Method B) analyses of **[^{18}F][ScF(22)]$^{-}$** metabolites collected 2 h p.i..

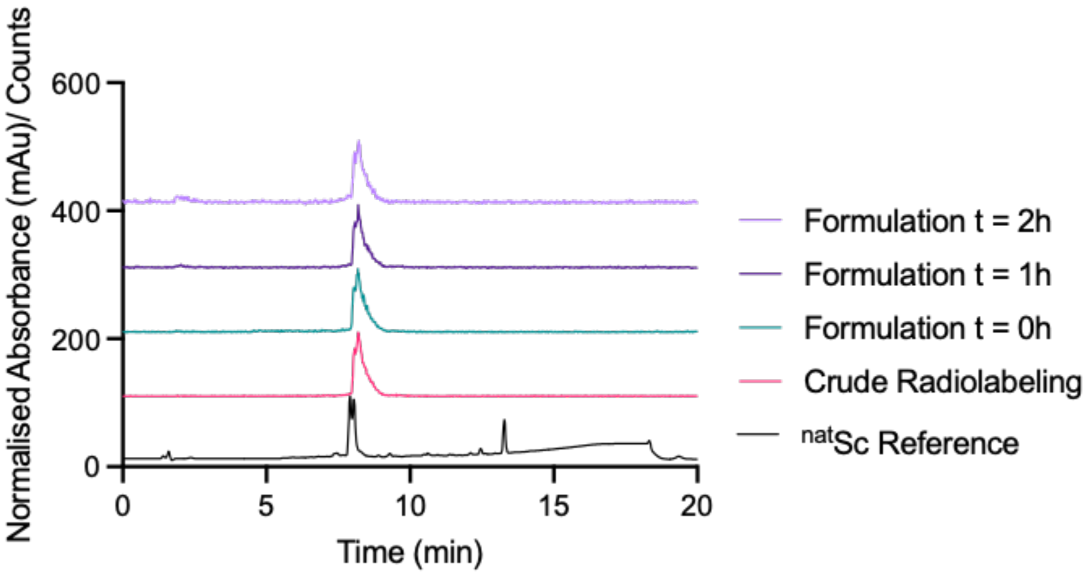

Figure S115. The RP-HPLC (Method C) analyses of **[natSc][Sc(22)]**, **[44Sc][Sc(22)]** radiolabeling solution, and formulation in PBS at t = 0, 1 and 2 h.

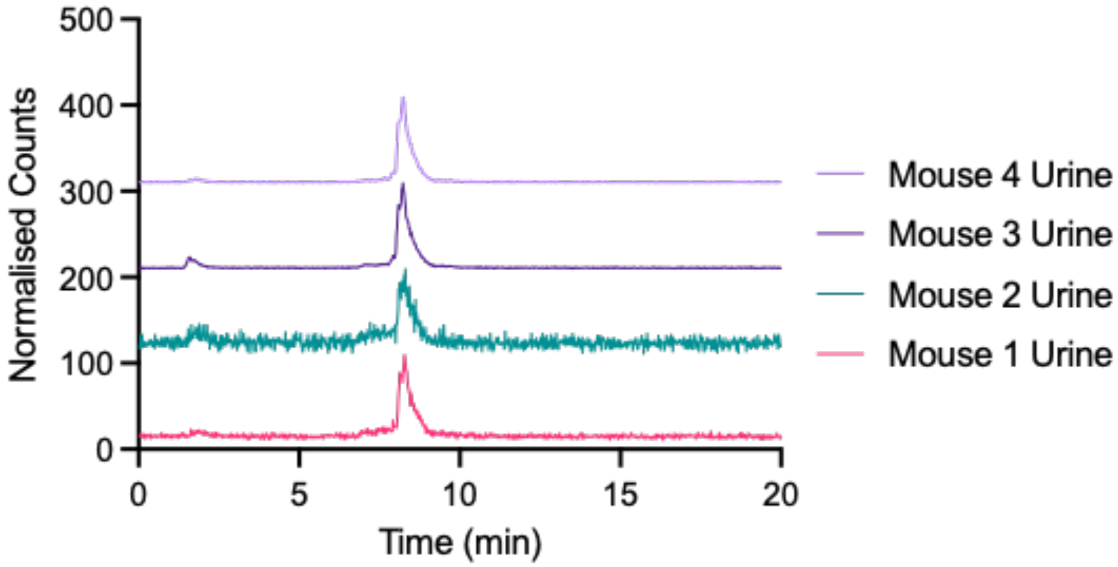

Figure S116. The RP-HPLC (Method C) analyses **[44Sc][Sc(22)]** metabolites collected 2 h p.i..

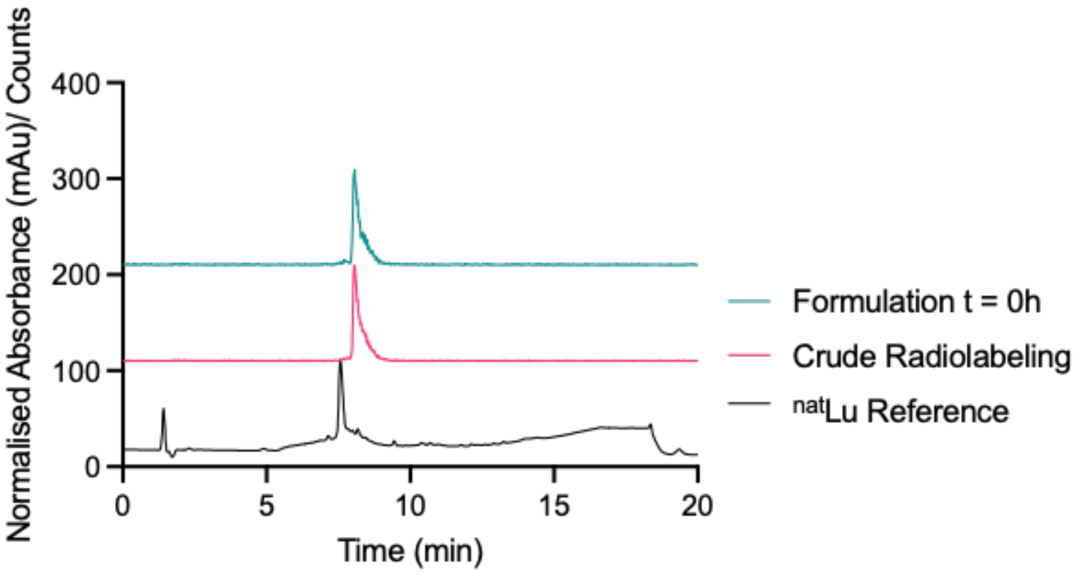

Figure S117. The RP-HPLC (Method C) analyses of **[natLu][Lu(22)]**, **[177Lu][Lu(22)]** radiolabeling solution, and formulation in PBS at t = 0 h.

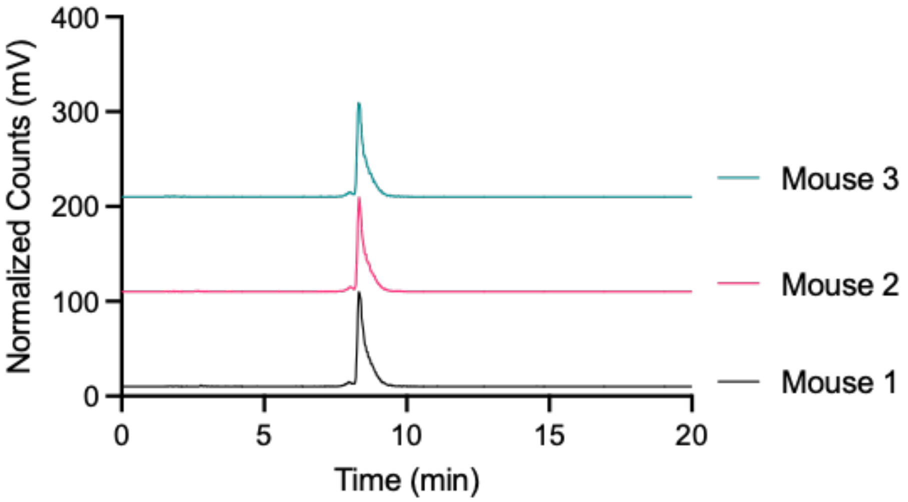

Figure S118. The RP-HPLC (Method B) analyses **[177Lu][Lu(22)]** metabolites collected 2 h p.i..

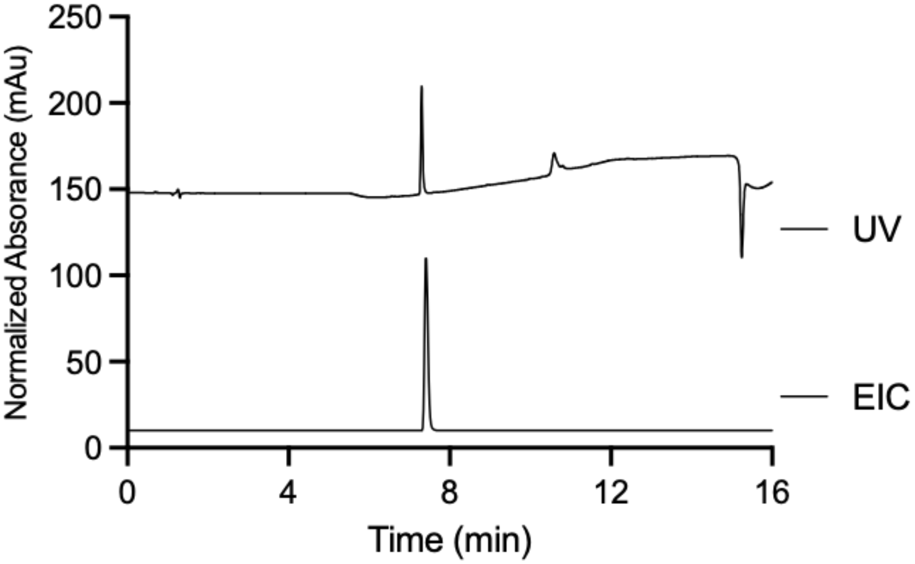

Figure S119. The UV (254 nm) and extracted ion (m/z = 865-867) LCMS (Method D) chromatograms of **14**.

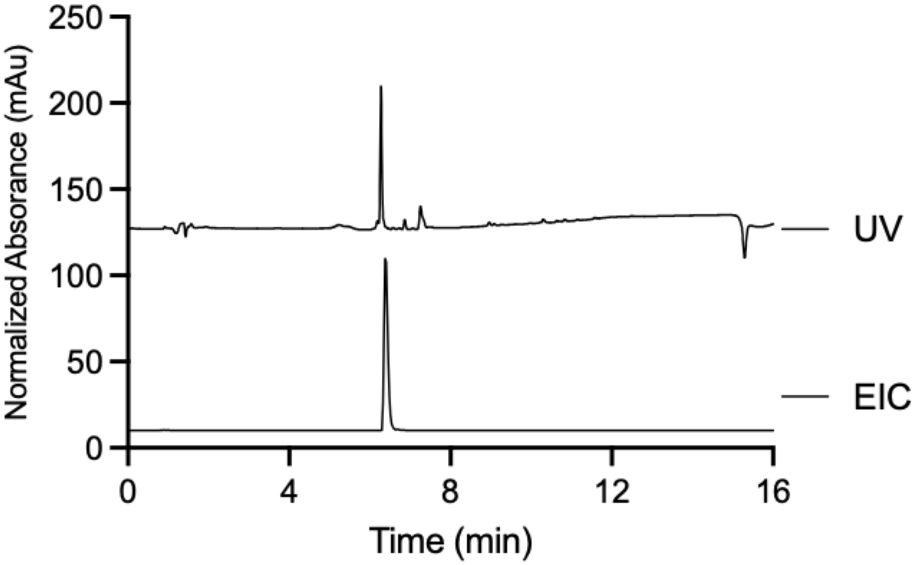

Figure S120. The UV (254 nm) and extracted ion (m/z = 913-915) LCMS (Method D) chromatograms of **19**.

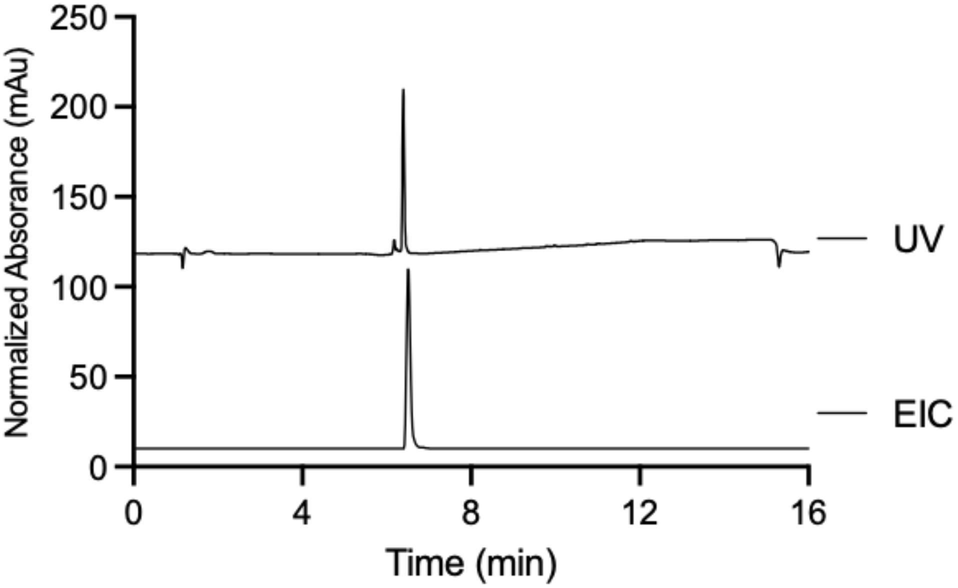

Figure S121. The UV (254 nm) and extracted ion (m/z = 997-999) LCMS (Method D) chromatograms of **22**.

Table S12. Tabulated radiofluorination yields of mpatcn at pH 4.5 and pH 7.4 (n=1).

Ligand	pH 4.5	pH 7.4
mpatcn	57%	65%

5.11 Radiolysis Stability

Radiolabeling was conducted following a literature procedure.[6] To an aqueous solution of ammonium acetate (10 µL, 1 M, pH 4.8) was added ligand stock solution (16-54 µL, 10 nmol), of known concentration as determined UV-vis spectroscopy, followed by an aliquot of [^{177}Lu]LuCl$_3$ stock (36-74 µL, ~1 mCi). Total reaction volume = 200 µL. The mixtures were incubated at 80 °C for 30 min prior to radioHPLC analysis (^{177}Lu: Method C). Following this, the radiolabeling solution was formulated in 1X PBS and 100 mM Ascorbate in 1X PBS. Aliquots of each formulation were analyzed via radioHPLC every 2 hours.

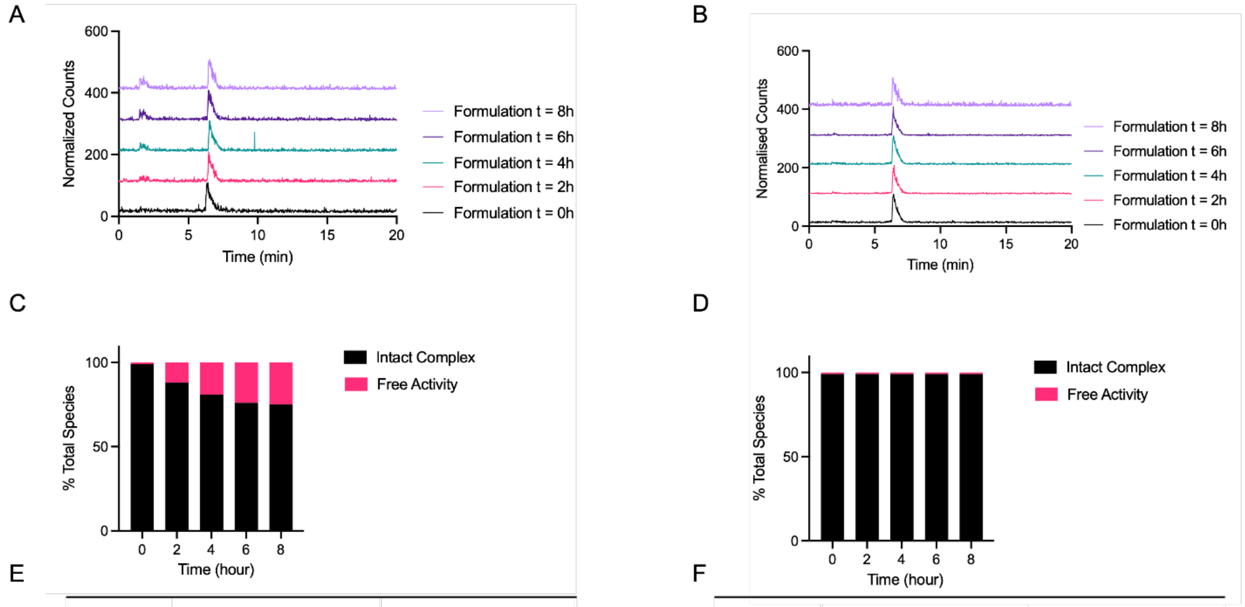

E

Time (h)	% Intact Complex	% Free Activity
0	99	1
2	88	12
4	81	19
6	76	24
8	75	25

F

Time (h)	% Intact Complex	% Free Activity
0	99	1
2	95.6	4.4
4	94.4	5.6
6	94.5	5.5
8	94.4	5.6

Figure S122. A) The RP-HPLC (Method C) analyses **[^{177}Lu][Lu(19)]** formulation in the absence of ascorbic acid. B) The RP-HPLC (Method C) analyses **[^{177}Lu][Lu(19)]** formulation in the presence of ascorbic acid. C) Quantification of the RP-HPLC (Method C) analyses **[^{177}Lu][Lu(19)]** formulation in the absence of ascorbic acid. D) Quantification of The RP-HPLC (Method C) analyses **[^{177}Lu][Lu(19)]** formulation in the presence of ascorbic acid. E) Tabulated values used for the quantification of **[^{177}Lu][Lu(19)]** degradation in the absence of ascorbic acid. F) Tabulated values used for the quantification of **[^{177}Lu][Lu(19)]** degradation in the presence of ascorbic acid.

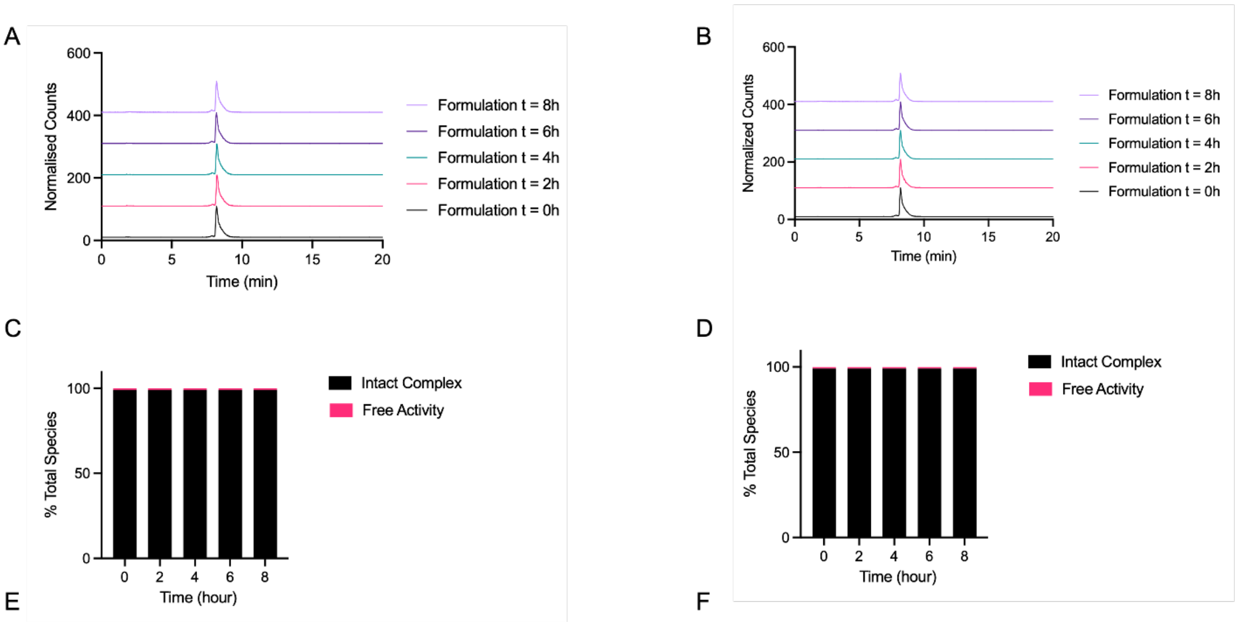

E

Time (h)	% Intact Complex	% Free Activity
0	>99	1
2	>99	1
4	>99	1
6	>99	1
8	>99	1

F

Time (h)	% Intact Complex	% Free Activity
0	>99	1
2	>99	1
4	>99	1
6	>99	1
8	>99	1

Figure S123. A) The RP-HPLC (Method C) analyses **[^{177}Lu][Lu(22)]** formulation in the absence of ascorbic acid. B) The RP-HPLC (Method C) analyses **[^{177}Lu][Lu(22)]** formulation in the presence of ascorbic acid. C) Quantification of the RP-HPLC (Method C) analyses **[^{177}Lu][Lu(22)]** formulation in the absence of ascorbic acid. D) Quantification of The RP-HPLC (Method C) analyses **[^{177}Lu][Lu(22)]** formulation in the presence of ascorbic acid. E) Tabulated values used for the quantification of **[^{177}Lu][Lu(22)]** degradation in the absence of ascorbic acid. F) Tabulated values used for the quantification of **[^{177}Lu][Lu(22)]** degradation in the presence of ascorbic acid.

5.12 QMA Cartridge Purification of ^{18}F Conjugates

A QMA cartridge was first primed using 10 mL of DI water. Following the general radiolabeling procedures outlined in section **5.1**, the crude radiolabeling solution of [^{18}F][ScF(picaga-Met-hex-KuE)], 200 µL, was loaded onto the QMA cartridge. Using 2 mL of 50 mM [K(Kryptofix2.2.2)]$^{+}$, free ^{18}F was eluted as [^{18}F][KF(Kryptofix2.2.2)] with fractions being separated into 100 µL aliquots.. Following this the **[^{18}F][ScF(22)]** was eluted using 500 µL 1 M NH_4OAc (pH 4.8) with fractions being separated into 100 µL aliquots.

5.13 Displacement assay

Stock solutions of **[^{18}F][ScF(22)], [^{177}Lu][Lu(22)],** and Pluvicto in H_2O were prepared and concentrations were determined by ICP (Agilent 5110 ICP-OES, Danbury, CT). A 6-point standard curve with respect to scandium or lutetium was used (R2 of 0.9998). Stock solutions were serially diluted 10x, resulting in stocks with concentrations as follows: 1 mM–10 pM **[^{18}F][ScF(22)],** 1 mM–10 pM **[^{177}Lu][Lu(22)]**, and 0.1 mM – 0.1 pM Pluvicto.

[^{18}F][Pylarify] (DCFPyL) was procured from SOFIE (Cleaveland, OH). 10 µL containing 110 µCi was used for each measurement.

Equation S3: $K_i = \frac{IC_{50}}{1+\frac{[S]}{K_m}}$

The Ki values were calculated using equation 1 below,[5] where the K_i value of Pluvicto was given as 6.5 ±0.8 nM.[11]

The K_i was determined by nonlinear regression analysis using GraphPad Prism software.

6 References

(1) Kelderman, C. A. A.; Glaser, O. M.; Whetter, J. N.; Aluicio-Sarduy, E.; Mixdorf, J. C.; Sanders, K. M.; Guzei, I. A.; Barnhart, T. E.; Engle, J. W.; Boros, E. Charting the coordinative landscape of the 18F–Sc/44Sc/177Lu triad with the tri-aza-cyclononane (tacn) scaffold. *Chemical Science* **2024**, *15* (43), 17927-17936.
(2) Berger, M. High Relaxivity Gadolinium Chelate Compounds For Use In Magnetic Resonance Imaging. United States of America 2018.
(3) Marlin, A.; Tran, P. N.; Dierolf, M.; DeLuca, M.; Joaqui Joaqui, M. A.; Glaser, O. M.; Koller, A. J.; Alucio-Sarduy, E.; Gork, M.; Śmiłowicz, D.; et al. Evaluation of PSMA-Targeted TREN-CAM Conjugates for Targeted Imaging of Cancer with 68Ga(III) and 45Ti(IV). *Bioconjugate Chemistry* **2025**, *36* (4), 859-866.
(4) Lengacher, R.; Martin, K. E.; Śmiłowicz, D.; Esseln, H.; Lotlikar, P.; Grichine, A.; Maury, O.; Boros, E. Targeted, Molecular Europium(III) Probes Enable Luminescence-Guided Surgery and 1 Photon Post-Surgical Luminescence Microscopy of Solid Tumors. *Journal of the American Chemical Society* **2023**, *145* (44), 24358-24366.
(5) Vaughn, B. A.; Ahn, S. H.; Aluicio-Sarduy, E.; Devaraj, J.; Olson, A. P.; Engle, J.; Boros, E. Chelation with a twist: a bifunctional chelator to enable room temperature radiolabeling and targeted PET imaging with scandium-44. *Chemical Science* **2020**, *11* (2), 333-342.
(6) Whetter, J. N.; Vaughn, B. A.; Koller, A. J.; Boros, E. An Unusual Pair: Facile Formation and In Vivo Validation of Robust Sc–18F Ternary Complexes for Molecular Imaging. *Angewandte Chemie International Edition* **2022**, *61* (7), e202114203.
(7) Whetter, J. N.; Śmiłowicz, D.; Becker, K. V.; Aluicio-Sarduy, E.; Kelderman, C. A. A.; Koller, A. J.; Glaser, O. M.; Marlin, A.; Ahn, S. H.; Kretowicz, M. N.; et al. Phosphonate-Based Aza-Macrocycle Ligands for Low-Temperature, Stable Chelation of Medicinally Relevant Rare Earth Radiometals and Radiofluorination. *Journal of the American Chemical Society* **2024**, *146* (48), 33121-33129.
(8) Nagy, G.; Szikra, D.; Trencsényi, G.; Fekete, A.; Garai, I.; Giani, A. M.; Negri, R.; Masciocchi, N.; Maiocchi, A.; Uggeri, F.; et al. AAZTA: An Ideal Chelating Agent for the Development of 44Sc PET Imaging Agents. *Angewandte Chemie International Edition* **2017**, *56* (8), 2118-2122.
(9) Umbricht, C. A.; Benešová, M.; Schmid, R. M.; Türler, A.; Schibli, R.; van der Meulen, N. P.; Müller, C. 44Sc-PSMA-617 for radiotheragnostics in tandem with 177Lu-PSMA-617—preclinical investigations in comparison with 68Ga-PSMA-11 and 68Ga-PSMA-617. *EJNMMI Research* **2017**, *7* (1), 9. DOI: 10.1186/s13550-017-0257-4.
(10) Chen, Y.; Pullambhatla M Fau - Foss, C. A.; Foss Ca Fau - Byun, Y.; Byun Y Fau - Nimmagadda, S.; Nimmagadda S Fau - Senthamizhchelvan, S.; Senthamizhchelvan S Fau - Sgouros, G.; Sgouros G Fau - Mease, R. C.; Mease Rc Fau - Pomper, M. G.; Pomper, M. G. 2-(3-{1-Carboxy-5-[(6-[18F]fluoro-pyridine-3-carbonyl)-amino]-pentyl}-ureido)-pentanedioic acid, [18F]DCFPyL, a PSMA-based PET imaging agent for prostate cancer. (1557-3265 (Electronic)).
(11) Śmiłowicz, D.; Schlyer, D.; Boros, E.; Meimetis, L. Evaluation of a Radio-IMmunoStimulant (RIMS) in a Syngeneic Model of Murine Prostate Cancer and ImmunoPET Analysis of T-cell Distribution. *Molecular Pharmaceutics* **2022**, *19* (9), 3217-3227.
